# A 3,4-*trans*-Fused Cyclic Protecting Group Facilitates α-Selective Catalytic Synthesis of 2-Deoxyglycosides**

**DOI:** 10.1002/anie.201403543

**Published:** 2014-06-20

**Authors:** Edward I Balmond, David Benito-Alifonso, Diane M Coe, Roger W Alder, Eoghan M McGarrigle, M Carmen Galan

**Affiliations:** School of Chemistry, University of Bristol, Cantock's CloseBristol BS8 1TS (UK); Centre for Synthesis and Chemical Biology, UCD School of Chemistry & Chemical Biology, University College DublinBelfield, Dublin 4 (Ireland); GlaxoSmithKline Medicines Research CentreGunnels Wood Road, Stevenage SG1 2NY (UK)

**Keywords:** conformation analysis, homogeneous catalysis, glycosides, protecting groups, synthetic methods

## Abstract

A practical approach has been developed to convert glucals and rhamnals into disaccharides or glycoconjugates with high α-selectivity and yields (77–97 %) using a *trans*-fused cyclic 3,4-O-disiloxane protecting group and TsOH⋅H_2_O (1 mol %) as a catalyst. Control of the anomeric selectivity arises from conformational locking of the intermediate oxacarbenium cation. Glucals outperform rhamnals because the C6 side-chain conformation augments the selectivity.

Chiral acetals are ubiquitous in many natural products ranging from spiroketal polyketides to complex oligosaccharides with a wide range of biological activities.[1] Deoxyhexoses are an important class of glycans which occur widely in natural products such as antibiotics and anticancer agents.[1b, 2] Thus, it is not surprising that much research has been devoted to the development of efficient and stereoselective methodologies which can give access to this important class of chiral acetals.[3] Herein we report a direct, practical, and stereoselective synthesis of 2-deoxyglucosides and l-rhamnosides using catalytic amounts of 4-toluenesulfonic acid monohydrate (TsOH⋅H_2_O). We show that conformational constraints induced by careful choice of protecting groups can help bias the outcome of the reaction and thus achieve high α-stereocontrol. Moreover, the importance of side-chain conformation is highlighted by increased selectivities with glucal as compared to rhamnal substrates.

The stereoselective formation of glycosidic linkages is one of the most challenging aspects of modern oligosaccharide chemistry.[4] With 2-deoxyglycosides, the lack of a C2 substituent, which can direct the coupling, further complicates matters and mixtures of anomers tend to be produced.[3j, 5] Despite the development of many indirect and direct methods, the stereoselective synthesis of deoxyglucosides remains particularly difficult.

As part of our ongoing interest in developing stereoselective glycosylation methods, we decided to focus our attention on the synthesis of deoxyglycosides. Recently, our team reported a mild organocatalytic method for the synthesis of 2-deoxygalactosides in excellent yields and α-selectivity[6] (Scheme 1 a). Although the thiourea **1** worked well with galactals, reactions with glucals such as **5** were slower, less stereoselective, and Ferrier rearrangement side-products (**7**)[7] were observed (Scheme 1 b). These results were not completely unexpected as glycosylation reactions with glucal derivatives tend to furnish poor selectivity and reactivity, which is normally attributed to the lack of the axial C4 OH substituent on the ring, thus leading to the attack of the nucleophile from both faces of the ring.[3j]

**Scheme 1:**
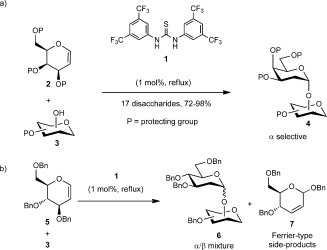
Thiourea-catalyzed synthesis of deoxyglycosides. a) Galactal series.[5] b) Glucal series.

It is generally agreed that acid-catalyzed direct nucleophilic substitution on a glycal is likely to proceed via oxacarbenium ion intermediates.[3j] It has been shown that substituted tetrahydropyran oxacarbenium ions generally adopt half-chair conformations (Scheme 2).[8] Two half-chair conformers are possible for these glucal intermediates and their respective stabilities are determined by steric effects as well as the electronic nature of the substituents.[8], [9] Nucleophilic addition to each conformer gives different diastereomeric products.[8], [10]

**Scheme 2:**
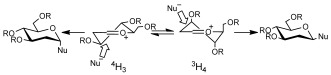
Diastereomeric half-chair conformers of glucal oxacarbenium ions and preferred nucleophilic attack to yield the corresponding glycosides.

Protecting groups can influence the reactivity of a glycosyl donor[11] and the conformer equilibrium by influencing thermodynamic factors.[12] For example, bulky silyl protecting groups *trans*-vicinal to each other in a carbohydrate moiety have been used to achieve higher reactivity, and in some cases selectivity, by way of inducing conformational constraints which favor molecules in axial-rich conformations.[12c, 13] Cyclic protecting groups can also influence the reactivity and stereoselectivity of glycosylation reactions.[14] The effect of 4,6-*O*-benzylidene acetals on the stereo-outcome of glycosylations has been extensively studied by Crich and co-workers[14a in the synthesis of mannopyranosides, and 3,5-*O*-di-*tert*-butylsilane and disiloxane acetals have been found to favor the formation of β-arabinofuranosides.[15]

We hypothesized that protecting-group-induced conformational constraints on the charged glucal-derived oxacarbenium ion could be used to influence the stereoselectivity of the glycosylation. To that end, we set out to explore the reactivity and stereoselectivity of a range of differentially protected glucals using the bench-stable protic acid TsOH⋅H_2_O as an economical, easy-to-handle, and more reactive glycosylation promoter, relative to the previously used **1**,[16] to efficiently activate the less reactive glucal donors.[3j] Thus, glucals containing benzyl and bulky silyl ethers (**5 a**–**e**), as well as derivatives with cyclic protecting groups such as 4,6-*O*-silyl and benzylidene acetal groups (**5 f**–**h**) and 3,4-*O*-siloxane derivatives (**5 i**,**j**) were synthesized and reacted with **3 a**, as the model glycoside acceptor, in CH_2_Cl_2_ (Table 1).

**Table 1:** Reaction of glycals with the model acceptor 3 a.[a] 
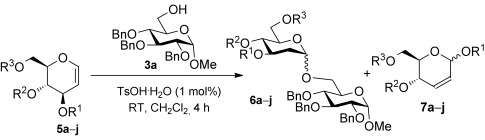

Entry	R^1^	R^2^	R^3^	Yield [%][b]	Yield [%][b]			
				6 a–j	α/β[b]	7 a–j	α/β[b]	
1	**5 a**	Bn	Bn	Bn	ca. 74	6:1	ca. 11	7:1
2	**5 b**	TBS	TBS	TBS	ca. 60	5:1	34	4:1
3	**5 c**	TBS	Bn	TBS	ca. 60	15:1	20	4:1
4	**5 d**	TBS	Ac	TBS	ca. 64[d]	15:1	2	4:1
5	**5 e**	TIPS	Allyl	TIPS	80[c]	15:1	4[c]	n.d.
6	**5 f**	TIPS	Si(*t*Bu)_2_	ca. 34	3:1	n.o.	–	
7	**5 g**	TIPS	CHPh	ca. 39	3:1	n.o.	–	
8	**5 h**	O[Si(*i*Pr)_2_]_2_	Bn	76[c]	α	n.o.	–	
9	**5 i**	O[Si(*i*Pr)_2_]_2_	TIPS	85[c]	α	n.o.	–	
10	**5 j**	O[Si(Me)_2_]_2_	TIPS	ca. 61	α	n.o.	–	

[a] Used 1.2–1.5 equiv of **5 a**–**j**.

[b] Measured by ^1^H NMR spectroscopy unless stated otherwise.

[c] Yield of isolated product.

[d] Reaction run for 8 h. n.d.=not determined, n.o.=not observed, TBS=*tert*-butyldimethylsilyl, TIPS=triisopropylsilyl.

Initial reactions with the perbenzylated glucal **5 a** and acceptor **3 a** using 1 mol % of TsOH⋅H_2_O at room temperature for 4 hours afforded an inseparable mixture of the disaccharide **6 a** (ca. 74 %, 6:1 α/β), the rearranged product **7 a** (ca. 11 %), and hydrolyzed starting material (Table 1, entry 1). When the persilylated glucal **5 b** was subjected to the same reaction conditions, the disaccharide **6 b** was formed in yields of over 60 % with similar α-selectivity as before, alongside 34 % of the rearranged **7 b** with a preference for the α-product (entry 2). Reactions with the disilylated derivatives **5 c**–**e**, which bear a benzyl or allyl ether, or an acetate ester at C4, afforded the disaccharides **6 c**–**e** in good yield (60–80 %) and with a strong preference for the α-glycosides. In these instances, the rearranged products (**7 c**-**e**) were also observed albeit in small amounts (entries 3–5). These results show that bulky silyl ether protecting groups can influence the stereo-outcome of the glycosylation with glucals, as previously shown for other glycoside donors.[12c, 13] Further increases in α-selectivity are obtained by switching R^2^ to a small group (entries 3–5). We speculate that this effect results from a shift in the conformer equilibrium in the absence of vicinal bulky groups,[13a] but we cannot rule out a change in the relative reactivity of the conformers.

Encouraged by these results, we decided to explore the effect of cyclic protecting groups. Reactions using the 4,6-*O*-linked **5 f** and **5 g** were slow and the disaccharides **6 f** and **6 g**, respectively, were produced in low yields (ca. 34–39 %) along with hydrolyzed starting materials (Table 1, entries 6 and 7). However no rearranged products were observed. This result was not completely unexpected as it has been shown[17] that conformational restraints imposed by these type of *trans*-fused bicyclic systems can disfavor the formation of oxacarbenium ions, and thus such substrates tend to be less reactive than the noncyclic derivatives.

Excitingly, reaction of the 3,4-*O*-tetraisopropyldisiloxane derivatives **5 h** and **5 i** under the same reaction conditions as before, afforded the disaccharides **6 h** (76 %) and **6 i** (85 %), respectively, and exclusively as the α glycoside (Table 1, entries 8 and 9). Complete α-selectivity was also observed with the formation of **6 j** when the less bulky 3,4-*O*-tetramethyldisiloxane **5 j**[18] was used as the glycoside donor, thus suggesting that the high α-selectivity of the reaction should be attributed to conformational constraints induced by the cyclic nature of the protecting group rather than to the steric bulk of the isopropyl substituents.

To determine the scope of the methodology, the glucal derivative **5 i** was used as the glycosyl donor and reacted with a range of differentially protected glycoside acceptors **3 b**–**h** (Table 2). In all cases, the reactions proceeded in excellent yields and with high α-selectivity. For instance, the thioglucoside acceptor **3 b** bearing a primary alcohol and benzoyl protecting groups gave an isolated product yield of 80 % and complete α-selectivity (entry 1). Similarly, reactions with secondary alcohols at different C-positions around the pyran ring, such as in the benzylidene-acetal-protected glucoside **3 c** or N-Troc-protected glucosamine **3 d**, also afforded disaccharides in excellent yields (81 %) and α-selectivity (entries 2, 3). The reaction was also applicable to the preparation of glycosyl amino acids and other glycoconjugates, thus reactions involving Boc- or Fmoc-protected serine (**3 e**,**f**) or Boc-threonine (**3 g**) yielded the desired products with complete α-selectivity and high yields (80–89 %). In the case of cholesterol **3 h**, the reaction proceeded with a yield of 82 % and in this instance a 20:1 α/β ratio was observed.

**Table 2:** Scope of acceptor in the glycosylation of the conformationally constrained glucal 5 i.[a] 
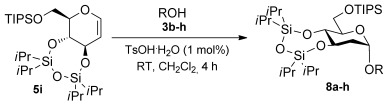

Entry	ROH	Yield [%][a]	α/β[b]	
1	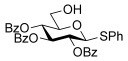	**3 b**[c]	80	α
2	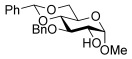	**3 c**[d]	81	α
3	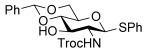	**3 d^[^**^e]^	81	α
4	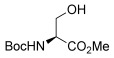	**3 e**	80	α
5	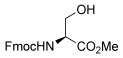	**3 f**	89	α
6	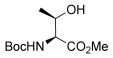	**3 g**	86	α
7	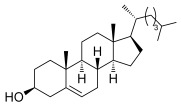	**3 h**[d]	82	20:1

[a] Yield of isolated product.

[b] Determined by ^1^H NMR spectroscopy.

[c] Reaction run for 16 h.

[d] Reaction run for 5 h.

[e] 2 mol % TsOH⋅H_2_O with respect to **3 d** (0.05 m). Boc=*tert*-butoxycarbonyl, Fmoc=9-fluorenylmethoxycarbonyl, Troc=trichlorethoxycarbonyl.

2,6-Dideoxyglycosides are also an important class of compounds and their stereoselective synthesis is further complicated by the lack of oxygen substituents at C6.[3b] Thus, we decided to explore the effect of the 3,4-*O*-siloxane protecting group strategy on the couplings of the l-rhamnal **9** with a series of alcohols, namely, **3 a**, **3 d**, **3 f**, and **3 h** (Table 3). These glycosylation reactions, using 1 % TsOH⋅H_2_O in CH_2_Cl_2_, proceeded in excellent yields (81–95 %) and with a high preference for the α-glycoside products in most instances, with the exception of **3 h** which afforded the glycoside **10 h** in a 7:2 α/β ratio. Glycosylation of the l-rhamnal **10**, which lacks the cyclic protecting group, with **3 a** (entry 5) yielded the disaccharide **12** with a lower α/β (4:1) ratio than that obtained with the *trans*-fused cyclic protecting group (entry 1), thus further highlighting the effect of the constraint on the l-rhamnal glycosylation stereo-outcome.

**Table 3:** Scope of acceptor in the glycosylation of conformationally constrained the l-rhamnal 9 and the non-cyclic protected 10.[a] 
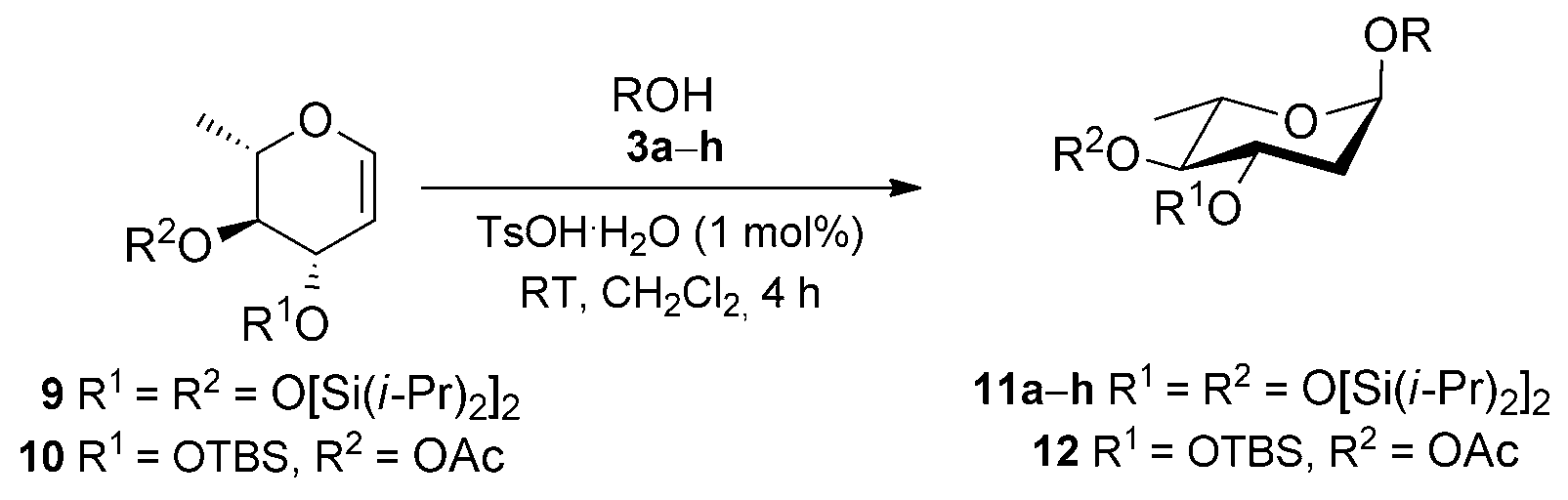

Entry	Donor	ROH	Yield [%][a]	α/β[b]
1	**9**	**3 a**	81	9:1
2	**9**	**3 d**[c]	84	α
3	**9**	**3 f**	95	10:1
4	**9**	**3 h**	95	7:2[d]
5	**10**	**3 a**	57	4:1

[a] Yield of the isolated product.

[b] Determined by ^1^H NMR spectroscopy after isolation.

[c] 2 mol % TsOH⋅H_2_O with respect to **3 d** (0.05 m).

[d] Ratio of each isomer after isolation.

To probe the mechanism, the deuterated glucal **13** was subjected to the reaction conditions using **3 a** as the glycoside acceptor, and **14 a** and **14 b** were isolated in a 4:1 ratio, whereby there is a preference for the newly formed bonds to be *cis* to each other (Scheme 3). The glycosides α-**8 d**, α-**11 d**, and β-**11 h** were re-subjected to the reaction conditions for 4 hours. Slow anomerization was only observed in the case of β-**11 h** (α/β 1:1.7 after 4 h). In monitoring the reaction of **5 i** to form **6 i** over time, no β-anomer was detected. These results suggest that upon formation of the oxacarbenium ion, the attack of the nucleophile is α-selective.

**Scheme 3:**
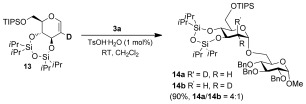
Mechanistic investigation.

DFT calculations were used to try to understand our observations.[19] It was found that the glucal oxacarbenium ion is held in an almost perfect half-chair (^4^H_3_) conformation by being *trans*-fused to the cyclic protecting group. The ^3^H_4_ conformation is simply unattainable because of the requirement for ring fusion of the protecting group through two axial bonds. The results of attack from the α- and β-faces on the half-chair oxacarbenium ion are different, as shown in Figure 1 a. Attack of the alcohol to give the major α product proceeds via a chairlike transition state (TS), but formation of the β product[20] requires a higher energy twist-boat-like TS,[21] therefore high α-selectivity results in most cases. The energy difference between chair and twist-boat 2-alkoxytetrahydropyrans is about 20 kJ mol^−1^, so α-attack will be favored by some fraction of this energy difference, depending on how early or late the transition state is. Although we could not locate true transition states using DFT calculations,[19] energy profiles for α- and β-attack of ROH suggest that, between 2 and 1.6 Å, α-attack is favored by 10–15 kJ mol^−1^, which we believe could account for the α-selectivity. This effect could also account for the α-selectivity in the rhamnal series.

**Figure 1:**
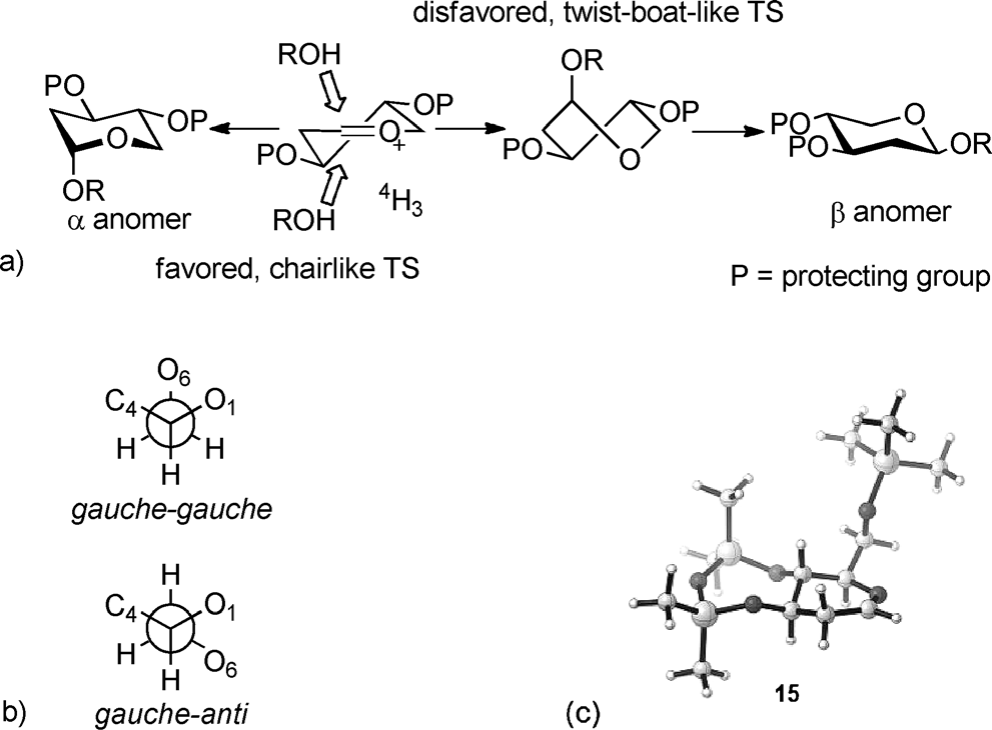
a) Chair and twist-boat products formed by axial α and β attack on a half-chair oxacarbenium ion. b) Staggered conformations about the exocyclic C5=C6 bond. The stereochemical relationship (*gauche* or *anti*) between O6 and O1 and C4 is described. c) Favored *gauche-gauche* conformation for the glucal oxacarbenium ion (15) with the O-C-CH_2_-OR torsion angle at −64°.

DFT calculations also enable a rationalization of the higher selectivity observed for glucals compared to rhamnals. Surprisingly, the glucal oxacarbenium ion prefers a conformation in which the C6 OSiR_3_ group adopts a *gauche-gauche* conformation with respect to the endocyclic oxygen atom and C4 (Figure 1 b), which orients the C6=O bond approximately parallel to the pseudoaxial substituents (Figure 1 c). This is 11 kJ mol^−1^ more stable than the *gauche-anti* conformation.[22] Thus, approach of a nucleophile from the top (β) face will be hindered and may also suffer from dipole repulsion. Hence, attack occurs on the α-face and excellent selectivities are observed with the 3,4-*O*-siloxane-protected glucals, but less so with the rhamnals which lack the C6 substituent.[23] This represents a further example of how subtle conformational effects can influence the outcome of reactions involving oxacarbenium ions.[12c, 21]

In conclusion, we have described a practical, stereoselective, and efficient direct glycosylation method for glucals and rhamnals, that is widely applicable to a range of nucleophile acceptors using the commercially available TsOH⋅H_2_O at room temperature. The reaction proceeds with excellent yields and high selectivity for the α anomer and is tolerant of most common alcohol protecting groups, that is, benzyl and silyl ethers, benzoyl esters and acetals, and carbamate amino protecting groups (Troc, Fmoc, and Boc). Moreover, we exemplify the generality of the approach in the stereoselective synthesis of a series of disaccharides, glycosyl amino acids and other glycoconjugates. We note that high α-selectivity has been achieved in reactions via an oxacarbenium ion intermediate, with a constrained conformation arising from the use of the *trans*-fused 3,4-disiloxane cyclic protecting group. In the case of glucal substrates the stereochemical outcome is further augmented by the conformational preference of the C6 side-chain. It should be noted that, in other cases, the two effects described above could act in opposition rather than supplementing each other as they do for the glucal derivatives with non-cyclic protecting groups. Our results further demonstrate the importance of protecting groups, not only in terms of electronics, but also in the effect that they have on the conformation of putative reaction intermediates and how such effects can be exploited to achieve the desired stereocontrol.

## Experimental Section

The glycoside acceptor (ca. 0.1 mmol) and glucal (1.2–1.5 equiv) were weighed into a round-bottom flask and placed under vacuum for 1 h. Then the flask was filled with N_2_, followed by the addition of a stock solution (1 mL, 1 mol %) of TsOH⋅H_2_O in anhydrous CH_2_Cl_2_. The solutions were then stirred at room temperature under N_2_ until the reaction was determined to be complete by TLC analysis of the crude material. The reactions were quenched with Et_3_N (60 μL), concentrated in vacuo, and purified by column chromatography.
