# Supplementary material for: A 3,4-trans-Fused Cyclic Protecting Group Facilitates α-Selective Catalytic Synthesis of 2-Deoxyglycosides
Source: Angew Chem Int Ed Engl. 2014 Jun 20;53(31):8190–4. doi: 10.1002/anie.201403543 (PMC4499252; doi:10.1002/anie.201403543)

Supporting Information

© Wiley-VCH 2014

69451 Weinheim, Germany

A 3,4-*trans*-Fused Cyclic Protecting Group Facilitates α-Selective Catalytic Synthesis of 2-Deoxyglycosides**

Edward I. Balmond, David Benito-Alifonso, Diane M. Coe, Roger W. Alder, Eoghan M. McGarrigle, and M. Carmen Galan**

anie_201403543_sm_miscellaneous_information.pdf

Electronic Supplementary Information

Table of Contents

Experimental Procedures....2

Glucal Synthesis....3

Acceptor Synthesis....21

Glycosylation Reactions....30

Glucal Scope with Acceptor **3a**....30

Acceptor Scope with Glucal **5i**....43

Acceptor Scope with Rhamnal **9** and **10**....50

Deuterated Experimental....56

References....57

Spectra....59

Experimental Procedures

General. Chemicals were purchased and used without further purification. Anhydrous solvents were obtained by passage through a column of anhydrous alumina using equipment from Anhydrous Engineering (University of Bristol) based on the Grubbs' design. Reactions requiring anhydrous conditions were performed under N_2; glassware and needles were either flame dried immediately prior to use or placed in an oven (150 °C) for at least 2 h and allowed to cool either in desiccators or under reduced pressure; liquid reagents, solutions or solvents were added *via* syringe through rubber septa. Reactions were monitored by TLC on Kieselgel 60 F254 (Merck). Detection was by examination under UV light (254 nm) and by charring with 10% H_2SO_4 in EtOH, vanillin and ninhydrin solutions where appropriate. Flash column chromatography was performed using silica gel [Merck, 230−400 mesh (40−63 μm)]. Extracts were concentrated *in vacuo* using both a rotary evaporator (bath temperatures up to 40 °C) at a pressure of either 15 mmHg (diaphragm pump) or 0.1 mmHg (oil pump), as appropriate, and a high vacuum line at room temperature. ^{1}H and ^{13}C NMR spectra were measured in the solvent stated at 400 or 500 MHz and 101 or 126 MHz respectively. ^{1}H and ^{13}C NMR chemical shifts are quoted in parts per million (ppm) and referenced to the residual solvent peak ($CDCl_3$: ^{1}H = 7.26 ppm and ^{13}C = 77.2 ppm, d_6-DMSO: ^{1}H = 2.50 ppm and ^{13}C = 39.5 ppm), coupling constants (*J*) are given in Hertz (Hz). Multiplicities are abbreviated as: br (broad), s (singlet), d (doublet), t (triplet), q (quartet)and m (multiplet) or combinations thereof. Assignments were made, where necessary, with the aid of COSY, HSQC and HMBC NMR experiments. For α/β mixtures only peaks that can clearly be assigned have been reported. The units of the specific rotation, (deg·mL)/(g·dm), are implicit and are not included with the reported value. Concentration *c* is given in g/100 mL.

Glucal Synthesis

1,2-Dideoxy-3,4,6-tri-O-benzyl-2-deoxy-D-arabino-1-hexenopyranose (5a)

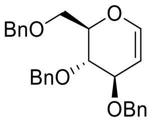

D-Glucal **S1** (2.098 g, 14.37 mmol), which had been pre-dried under vacuum for 1 h, was dissolved in anhydrous DMF (100 mL) and cooled to 0 °C. NaH (60% in mineral oil, 3.527 g, 88.18 mmol) was added and the solution was then stirred at room temperature for 30 min, after which the mixture was cooled to 0 °C and BnBr (10.0 mL, 84.1 mmol) was added dropwise. The solution was then stirred at room temperature for 18 h at which time the reaction was quenched with MeOH (5 mL) and the solution concentrated *in vacuo.* The obtained residue was dissolved in CH_2Cl_2 (100 mL) and washed with H_2O (100 mL). The aqueous phase was washed with CH_2Cl_2 (2 x 50 mL), the organic layers were combined, washed with brine (50 mL), dried over $MgSO_4$ and concentrated *in vacuo*. The residue was purified by column chromatography (95:5 to 9:1, Petrol ether (40-60):EtOAc) to afford glucal **5a** as a white solid (5.829 g, 97%). ^{1}H NMR (400 MHz; $CDCl_3$) δ: 7.40-7.24 (15H, m, Ph), 6.45 (1H, dd, *J* = 6.1, 1.1 Hz, H-1), 4.90 (1H, dd, *J* = 6.2, 2.7 Hz, H-2), 4.86 (1H, d, *J* = 11.3 Hz, OC*H*HPh), 4.67 (1H, d, *J* = 11.3 Hz, OCH*H*Ph), 4.66 (1H, d, *J* = 11.7 Hz, OC*H*HPh), 4.63 (1H, d, *J* = 12.0 Hz, OC*H*HPh), 4.59 (2H, app d, *J* = 12.0 Hz, 2 x OCH*H*Ph), 4.27-4.21 (1H, m, H-3), 4.09 (1H, ddd, *J* = 8.4, 5.1, 2.9 Hz, H-5), 3.89 (1H, dd, *J* = 8.6, 6.2 Hz, H-4), 3.84 (1H, dd, *J* = 10.7, 5.0 Hz, H-6a), 3.79 (1H, dd, *J* = 10.7, 2.9 Hz, H-6b); ^{13}C NMR (101 MHz; $CDCl_3$) δ: 144.8 (C-1), 138.5 (4° C), 138.3 (4° C), 138.1 (4° C), 128.53 (CH), 128.51 (CH), 128.49 (CH), 128.0 (CH), 127.91 (CH), 127.86 (CH), 127.85 (CH), 127.77 (CH), 127.76 (CH), 100.1 (C-2), 76.9 (C-5), 75.9 (C-3), 74.5 (C-4), 73.9 (O*C*H_2Ph), 73.6 (O*C*H_2Ph), 70.6 (O*C*H_2Ph), 68.6 (C-6). Proton and carbon NMR were consistent with literature data, apart from C-3 and C-5 which have been reassigned on the basis of the HSQC NMR spectrum.[1]

1,2-Dideoxy-3,4,6-tri-*O-tert*-butyldimethylsilyl-D-arabino-1-hexenopyranose (5b)

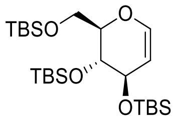

Following the reported methodology,[2] D-glucal **S1** (496 mg, 3.39 mmol) and imidazole (1.456 g, 21.39 mmol), which had been pre-dried under vacuum for 1 h, were dissolved in anhydrous DMF (25 mL) under a N_2 atmosphere. TBSCl (1.856 g, 12.31 mmol) was added and the mixture was heated to 60 °C, and stirred at this temperature for 18 h at which point the mixture was concentrated *in vacuo*. The crude residue was dissolved in CH_2Cl_2 (25 mL) and washed with H_2O (25 mL), the aqueous phase was washed with CH_2Cl_2 (10 mL). The combined organic phases were dried over Na_2SO_4, filtered and concentrated *in vacuo*. Purification by column chromatography (99:1 to 97:3, Hexane:EtOAc) afforded glucal **5b** as an oil (1.515 g, 91%). ^{1}H NMR (400 MHz; $CDCl_3$) δ: 6.32 (1H, dd, J = 6.3, 0.5 Hz, H-1), 4.69 (1H, ddd, J = 6.2, 4.4, 1.2 Hz, H-2), 3.99 (1H, dtd, J = 7.4, 3.7, 1.5 Hz, H-5), 3.93 (1H, dd, J = 11.1, 7.4 Hz, H-6a), 3.90-3.87 (1H, m, H-3), 3.79 (1H, td, J = 3.6, 1.2 Hz, H-4), 3.76 (1H, dd, J = 11.1, 3.4 Hz, H-6b), 0.90 (9H, s, $SiC(CH_3)_3$), 0.888 (9H, s, $SiC(CH_3)_3$), 0.886 (9H, s, $SiC(CH_3)_3$), 0.10 (6H, s, 2 x $SiCH_3$), 0.079 (3H, s, $SiCH_3$), 0.075 (3H, s, $SiCH_3$), 0.06 (3H, s, $SiCH_3$), 0.05 (3H, s, $SiCH_3$); ^{13}C NMR (101 MHz; $CDCl_3$) δ: 143.1 (C-1), 101.5 (C-2), 80.2 (C-5), 70.4 (C-4), 66.9 (C-3), 61.9 (C-6), 26.1 ($SiC(\mathit{C}H_3)_3$), 26.02 ($SiC(\mathit{C}H_3)_3$), 26.01 ($SiC(\mathit{C}H_3)_3$), 18.6 ($Si\mathit{C}(CH_3)_3$), 18.24 ($Si\mathit{C}(CH_3)_3$), 18.16 ($Si\mathit{C}(CH_3)_3$), -4.1 ($SiCH_3$), -4.16 ($SiCH_3$), -4.24 ($SiCH_3$), -4.6 ($SiCH_3$), -5.0 ($SiCH_3$), -5.1 ($SiCH_3$). Proton and carbon NMR were consistent with literature data.[3]

1,2-Dideoxy-3,6-di-*O-tert*-butyldimethylsilyl-2-deoxy-D-arabino-1-hexenopyranose (S2)

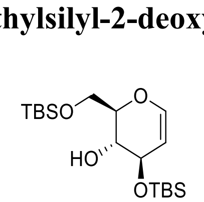

Following the reported synthesis,[4] D-glucal **S1** (513 g, 3.51 mmol) and imidazole (1.226 g, 18.01 mmol) which had been pre-dried under vacuum for 1 h, were dissolved in anhydrous DMF (18 mL) and cooled to 0 °C. TBSCl (1.132 g, 7.511 mmol) was added and the solution was stirred at 0 °C for 4 h. The reaction was concentrated *in vacuo* and dissolved in CH_2Cl_2 (25 mL) and washed with H_2O (25 mL). The aqueous phase was extracted with CH_2Cl_2 (10

mL) and the combined organic phases were dried over Na_2SO_4, filtered and concentrated *in vacuo*. Following purification by column chromatography (95:5, Hexane:EtOAc) glucal **S2** obtained as a white solid (1.091 g, 83%). ^{1}H NMR (500 MHz; $CDCl_3$) δ: 6.27 (1H, dd, *J* = 6.1, 1.5 Hz, H-1), 4.62 (1H, dd, *J* = 6.1, 2.4 Hz, H-2), 4.26-4.21 (1H, m, H-3), 3.97 (1H, dd, *J* = 11.3, 4.9 Hz, H-6a), 3.88 (1H, dd, *J* = 11.3, 3.8 Hz, H-6b), 3.85-3.80 (1H, m, H-5), 3.78-3.73 (1H, m, H-4), 2.51 (1H, d, *J* = 3.6 Hz, OH), 0.913 (9H, s, $SiC(CH_3)_3$), 0.908 (9H, s, $SiC(CH_3)_3$), 0.122 (3H, s, $SiCH_3$), 0.119 (3H, s, $SiCH_3$), 0.090 (3H, s, $SiCH_3$), 0.087 (3H, s, $SiCH_3$); ^{13}C NMR (126 MHz; $CDCl_3$) δ: 143.5 (C-1), 103.6 (C-2), 78.0 (C-5), 71.3 (C-4), 70.1 (C-3), 63.4 (C-6), 26.1 (SiC(*C*H$_3$)$_3$), 26.0 (SiC(*C*H$_3$)$_3$), 18.6 (Si*C*(CH$_3$)$_3$), 18.3 (Si*C*(CH$_3$)$_3$), -4.28 ($SiCH_3$), -4.33 ($SiCH_3$), -5.15 ($SiCH_3$), -5.22 ($SiCH_3$). Proton and carbon NMR were consistent with literature data.[5]

4-*O*-Benzyl-1,2-dideoxy-3,6-di-*O*-*tert*-butyldimethylsilyl-2-deoxy-D-arabino-1-hexenopyranose (5c)

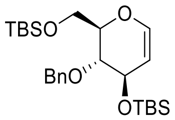

Glucal **S2** (1.109 g, 2.96 mmol) which had been pre-dried under vacuum for 1 h, was dissolved in anhydrous THF (18 mL) and cooled to 0 °C. NaH (60% in mineral oil, 236 mg, 5.90 mmol) was added and the mixture stirred at room temperature for 30 min. The mixture was cooled back to 0 °C followed by the addition of benzyl bromide (0.54 mL, 4.5 mmol) and the reaction was allowed to warm to room temperature and stir for 16 h. The reaction was quenched by the addition of MeOH (1 mL). The mixture was diluted with CH_2Cl_2 (30 mL) and washed with H_2O (25 mL), the organic layer was dried over Na_2SO_4 and concentrated *in vacuo*. The residue was purified by column chromatography (98:2 to 97:3, Hexane:EtOAc) to afford glucal **5c** as an oil (1.133 g, 82%). ^{1}H NMR (400 MHz; $CDCl_3$) δ: 7.37-7.26 (5H, m, Ph), 6.31 (1H, dd, *J* = 6.1, 1.4 Hz, H-1), 4.82 (1H, d, *J* = 11.2 Hz, OC*H*HPh), 4.72 (1H, d, *J* = 11.2 Hz, OCH*H*Ph), 4.63 (1H, dd, *J* = 6.1, 2.8 Hz, H-2), 4.36-4.32 (1H, m, H-3), 3.96 (1H, dd, *J* = 11.2, 4.6 Hz, H-6a), 3.93-3.87 (1H, m, H-5), 3.83 (1H, dd, *J* = 11.2, 2.2 Hz, H-6b), 3.64 (1H, dd, *J* = 8.4, 6.1 Hz, H-4), 0.91 (9H, s, $SiC(CH_3)_3$), 0.90 (9H, s, $SiC(CH_3)_3$), 0.10 (3H, s, $SiCH_3$), 0.09 (3H, s, $SiCH_3$), 0.06 (6H, s, 2 x $SiCH_3$); ^{13}C NMR (101 MHz; $CDCl_3$) δ: 143.6 (C-1), 138.6 (4° C), 128.5 (CH), 128.0 (CH), 127.8 (CH), 103.4 (C-2), 78.3 (C-5), 76.7

(C-4), 74.1 (OCH_2Ph), 69.1 (C-3), 62.1 (C-6), 26.1 (SiC(CH_3)$_3$), 26.0 (SiC(CH_3)$_3$), 18.6 (Si*C*(CH_3)$_3$), 18.2 (Si*C*(CH_3)$_3$), -4.3 ($SiCH_3$), -4.4 ($SiCH_3$), -5.0 ($SiCH_3$), -5.2 ($SiCH_3$). Proton and carbon NMR were consistent with literature data.[6]

4-*O*-Acetyl-1,2-dideoxy-3,6-di-*O*-*tert*-butyldimethylsilyl-2-deoxy-D-arabino-1-hexenopyranose (5d)

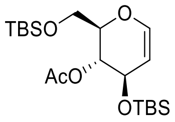

Glucal **S2** (1.091 g, 2.912 mmol) was dissolved in a solution of pyridine (4 mL) and Ac_2O (2 mL). The mixture was stirred at room temperature for 16 h at which point it was diluted with CH_2Cl_2 (25 mL). The solution was washed with 2 M HCl (25 mL), $NaHCO_3$ $_{(sat.\ aq.)}$ (25 mL), dried over Na_2SO_4, filtered and concentrated *in vacuo*. The residue was purified by column chromatography (95:5, Hexane:EtOAc) to afford glucal **5d** as an oil (1.175 g, 97%). ^{1}H NMR (400 MHz; $CDCl_3$) δ: 6.35 (1H, dd, *J* = 6.2, 0.7 Hz, H-1), 4.99 (1H, app t, *J* = 5.2 Hz, H-4), 4.72 (1H, ddd, *J* = 6.2, 3.7, 0.4 Hz, H-2), 4.14-4.10 (1H, m, H-3), 4.10-4.05 (1H, m, H-5), 3.91 (1H, dd, *J* = 11.6, 7.2 Hz, H-6a), 3.74 (1H, dd, *J* = 11.6, 3.2 Hz, H-6b), 2.08 (3H, s, (C=O)CH_3), 0.89 (9H, s, SiC(CH_3)$_3$), 0.87 (9H, s, SiC(CH_3)$_3$), 0.08 (6H, s, 2 x $SiCH_3$), 0.054 (3H, s, $SiCH_3$), 0.048 (3H, s, $SiCH_3$); ^{13}C NMR (101 MHz; $CDCl_3$) δ: 169.8 (C=O), 143.4 (C-1), 102.3 (C-2), 77.5 (C-5), 71.1 (C-4), 64.9 (C-3), 62.2 (C-6), 26.1 (SiC(CH_3)$_3$), 25.8 (SiC(CH_3)$_3$), 21.2 ((C=O)CH_3), 18.55 (Si*C*(CH_3)$_3$), 18.1 (Si*C*(CH_3)$_3$), -4.5 ($SiCH_3$), -4.8 ($SiCH_3$), -5.1 ($SiCH_3$), -5.2 ($SiCH_3$). Proton and carbon NMR were consistent with literature data.[4, 7]

1,2-Dideoxy-3,6-di-*O*-triisopropylsilyl-2-deoxy-D-arabino-1-hexenopyranose (S3)

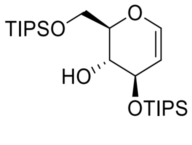

Following the reported synthesis,[8] D-glucal **S1** (2.064 g, 14.12 mmol) and imidazole (3.850 g, 56.55 mmol) which had been pre-dried under vacuum for 1 h, were dissolved in anhydrous DMF (28 mL) and cooled to 0 °C. TIPSCl (9.5 mL, 44 mmol) was added and the solution

was allowed to warm to room temperature. The reaction was stirred for 18 h at which point it was quenched by the addition of MeOH (2 mL) and concentrated *in vacuo*. The residue was dissolved in CH_2Cl_2 (50 mL) and washed with H_2O (50 mL). The aqueous phase was extracted with CH_2Cl_2 (2 x 25 mL) and the combined organic phases were dried over Na_2SO_4, filtered and concentrated *in vacuo*. Following purification by column chromatography (99:1 to 95:5, Petrol ether (40-60):EtOAc) glucal **S3** obtained as an oil (5.399 g, 85%). ^1H NMR (400 MHz; $CDCl_3$) δ: 6.29 (1H, dd, *J* = 6.1, 1.4 Hz, H-1), 4.71 (1H, dd, *J* = 6.2, 2.7 Hz, H-2), 4.33-4.28 (1H, m, H-3), 4.08 (1H, dd, *J* = 10.8, 5.1 Hz, H-6a), 3.94 (1H, dd, *J* = 10.8, 3.7 Hz, H-6b), 3.93-3.88 (1H, m, H-5), 3.83 (1H, ddd, *J* = 7.9, 5.9, 4.2 Hz, H-4), 2.53 (1H, d, *J* = 4.2 Hz, OH), 1.16-0.99 (42H, m, 2 x $OSi(C\mathit{H}(C\mathit{H}_3)_2)_3$); ^{13}C NMR (101 MHz; $CDCl_3$) δ: 143.3 (C-1), 103.5 (C-2), 78.4 (C-5), 71.7 (C-4), 69.4 (C-3), 63.6 (C-6), 18.22 ($OSi(CH(\mathit{C}H_3)_2)_3$), 18.21 ($OSi(CH(\mathit{C}H_3)_2)_3$), 18.1 ($OSi(CH(\mathit{C}H_3)_2)_3$), 12.6 ($OSi(\mathit{C}H(CH_3)_2)_3$), 12.1 ($OSi(\mathit{C}H(CH_3)_2)_3$). Proton and carbon NMR were consistent with literature data.[9]

4-*O*-Allyl-3,6-di-*O*-triisopropylsilyl-1,2-dideoxy-2-deoxy-D-arabino-1-hexenopyranose (5e)

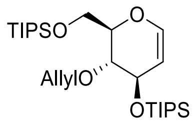

Glucal **S3** (5.399 g, 11.77 mmol) which had been pre-dried under vacuum for 1 h, was dissolved in anhydrous DMF (50 mL) and cooled to 0 °C. Allyl bromide (1.8 mL, 21 mmol) was added followed by NaH (60% in mineral oil, 820 mg, 20.5 mmol) and the reaction was allowed to warm to room temperature and stir for 16 h. The reaction was quenched by the addition of MeOH (5 mL) and concentrated *in vacuo*. The obtained residue was dissolved in CH_2Cl_2 (50 mL) and washed with H_2O (50 mL). The aqueous phase was extracted with CH_2Cl_2 (2 x 25 mL), the organic layers were combined, dried over Na_2SO_4 and concentrated *in vacuo*. The residue was purified by column chromatography (8:2 to 6:4, Petrol ether (40-60):PhMe) to afford glucal **5e** as an oil (5.341 g, 95%). ^1H NMR (400 MHz; $CDCl_3$) δ: 6.31 (1H, dd, *J* = 6.2, 1.1 Hz, H-1), 5.92 (1H, ddt, *J* = 17.3, 10.4, 5.7 Hz, $C\mathit{H}$=CH_2), 5.26 (1H, dq, *J* = 17.3, 1.5 Hz, CH=C*H*H), 5.16 (1H, dq, *J* = 10.3, 1.5 Hz, CH=CH*H*), 4.73 (1H, ddd, *J* = 6.2, 3.4, 0.6 Hz, H-2), 4.31 (1H, dtd, *J* = 4.7, 3.6, 1.0 Hz, H-3), 4.28-4.17 (2H, m,

$CH_2CH=CH_2$), 4.06 (1H, dd, *J* = 11.0, 5.4 Hz, H-6a), 4.03-3.98 (1H, m, H-5), 3.96 (1H, dd, *J* = 10.9, 2.3 Hz, H-6b), 3.63-3.58 (1H, m, H-4), 1.16-0.99 (42H, m, 2 x OSi(C*H*(C*H*$_3$)$_2$)$_3$); ^{13}C NMR (101 MHz; $CDCl_3$) δ: 143.4 (C-1), 135.0 (*C*H=CH_2), 117.2 (CH=*C*H_2), 102.8 (C-2), 78.2 (C-5), 76.6 (C-4), 72.3 (*C*H_2CH=CH_2), 67.4 (C-3), 62.2 (C-6), 18.3 (Si(CH(*C*H_3)$_2$)$_3$), 18.2 (Si(CH(*C*H_3)$_2$)$_3$), 18.12 (Si(CH(*C*H_3)$_2$)$_3$), 18.11 (Si(CH(*C*H_3)$_2$)$_3$), 12.6 (Si(*C*H(CH_3)$_2$)$_3$), 12.2 (Si(*C*H(CH_3)$_2$)$_3$). ESI-HRMS for $C_{27}H_{54}NaO_4Si_2^+$ $(M+Na)^+$ calculated: 521.3453; found: 521.3450. $[\alpha]_D^{21}$ = -4 (*c* 1.0, $CHCl_3$).

4,6-*O*-[Bis(*tert*-butyl)silylene]-1,2-dideoxy-D-arabino-1-hexenopyranose (S4)

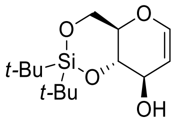

Following reported methodology,[10] D-glucal **S1** (2.89 g, 19.8 mmol) which had been pre-dried under vacuum in a flame-dried flask for 1 h, was dissolved in anhydrous DMF (60 mL) under a N_2 atmosphere. The reaction mixture was cooled to -45 °C and a solution of (*t*Bu)$_2$Si(OTf)$_2$ (4.2 mL, 12.9 mmol) in anhydrous CH_2Cl_2 (16 mL) was added dropwise over 40 min. The reaction was left stirring at -45 °C for 45 min after which distilled pyridine (3.2 mL) was added and the solution was allowed to warm to 0 °C. After 30 min at 0 °C the reaction was quenched with $NaHCO_{3\ (sat.\ aq.)}$ (15 mL) and then diluted with a solution of hexane/EtOAc (5:1, 450 mL). The organic layer was washed with brine (1 x 80 mL, 4 x 40 mL), dried over $MgSO_4$, filtered and concentrated to give glucal **S4** was white solid (3.51 g, 95%). ^{1}H NMR (400 MHz; $CDCl_3$) δ: 6.26 (1H, ddd, *J* = 6.1, 1.9, 0.5 Hz, H-1), 4.76 (1H, dd, *J* = 6.1, 1.9 Hz, H-2), 4.30 (1H, m, H-3), 4.18 (1H, dd, *J* = 10.3, 5.0 Hz, H-6a), 3.96 (1H, t, *J* = 10.2 Hz, H-6b), 3.92 (1H, dd, *J* = 10.2, 7.2 Hz, H-4), 3.84 (1H, td, *J* = 10.2, 4.8 Hz, H-5), 2.40 (1H, d, *J* = 3.3 Hz, OH), 1.07 (9H, s, C(CH_3)$_3$), 0.99 (9H, s, C(CH_3)$_3$); ^{13}C NMR (101 MHz; $CDCl_3$) δ: 143.8 (C-1), 103.1 (C-2), 77.5 (C-4), 72.4 (C-5), 70.4 (C-3), 65.9 (C-6), 27.6 (C(*C*H_3)$_3$), 27.1 (C(*C*H_3)$_3$), 22.9 (*C*(CH_3)$_3$), 20.0 (*C*(CH_3)$_3$). Proton and carbon NMR were consistent with literature data.[11]

4,6-*O*-[Bis(*tert*-butyl)silylene]-1,2-dideoxy-3-*O*-triisopropylsilyl-D-arabino-1-hexenopyranose (5f)

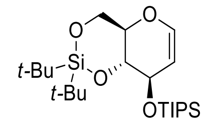

Following the reported synthesis,[12] glucal **S4** (1.525 g, 5.324 mmol) and imidazole (735 mg, 10.8 mmol) which had been pre-dried under vacuum in a flame-dried flask for 1 h, were dissolved in anhydrous DMF (60 mL) under a N_2 atmosphere. TIPSCl (2.3 mL, 11 mmol) was added and the reaction mixture was heated at 45 °C for 18 h, at which point the solution was concentrated *in vacuo*. The residue was dissolved in CH_2Cl_2 (100 mL) and washed with H_2O (80 mL), the aqueous phase was washed with CH_2Cl_2 (2 x 40 mL), the organic phases were combined and washed with brine (80 mL), dried over $MgSO_4$, filtered and concentrated *in vacuo*. Following purification by column chromatography (99:1 Hexane:EtOAc) glucal **5f** was obtained as a solid (2.092 g, 89%). ^{1}H NMR (400 MHz; $CDCl_3$) δ: 6.23 (1H, ddd, *J* = 6.1, 1.7, 0.5 Hz, H-1), 4.67 (1H, d, *J* = 6.1, 2.0 Hz, H-2), 4.42 (1H, dt, *J* = 6.9, 1.8 Hz, H-3), 4.15 (1H, dd, *J* = 10.2, 5.0 Hz, H-6a), 4.01 (1H, dd, *J* = 10.3, 6.9 Hz, H-4), 3.95 (1H, t, *J* = 10.4, Hz, H-6b), 3.83 (1H, td, *J* = 10.3, 5.0 Hz, H-5), 1.15-1.08 (21H, m, 3 x Si(C*H*(C*H*$_3$)$_2$), 1.06 (9H, s, C(CH_3)$_3$), 0.99 (9H, s, C(CH_3)$_3$); ^{13}C NMR (101 MHz; $CDCl_3$) δ: 142.8 (C-1), 105.5 (C-2), 77.7 (C-4), 72.9 (C-5), 71.0 (C-3), 66.2 (C-6), 27.6 (C(*C*H$_3$)$_3$), 27.1 (C(*C*H$_3$)$_3$), 22.9 (*C*(CH_3)$_3$), 20.0 (*C*(CH_3)$_3$), 18.30 (Si(CH(*C*H$_3$)(CH_3)), 18.28 (Si(CH(CH_3)(*C*H$_3$)), 12.6 (Si(*C*H(CH_3)$_2$). Proton and carbon NMR were consistent with literature data.[13]

Methyl 4,6-*O*-benzylidene-α-D-glucopyranoside (S6)

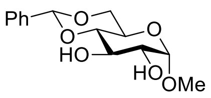

Following the reported methodology,[14] methyl α-D-glucopyranoside **S5** (10.02 g, 51.6 mmol), which had been pre-dried under vacuum in a flame-dried flask for 1 h, was dissolved in anhydrous MeCN (200 mL) under a N_2 atmosphere, Cu(OTf)$_2$ (1.10 g, 3.05 mmol) was added followed by benzaldehyde dimethyl acetal (9.5 mL, 63 mmol) and the solution sonicated under N_2 for 1.5 h and quenched by addition of Et_3N (2 mL). The mixture was concentrated *in vacuo*, following purification by column chromatography (95:5, CH_2Cl_2:MeOH) product

S6 was obtained as a white solid (13.9 g, 95%). ^{1}H NMR (400 MHz; $CDCl_3$) δ: 7.53-7.46 (2H, m, Ph), 7.41-7.33 (3H, m, Ph), 5.53 (1H, s, $PhCHO_2$), 4.80 (1H, d, J = 3.9 Hz, H-1), 4.30 (1H, dd, J = 9.7, 4.3 Hz, H-6a), 3.93 (1H, td, J = 9.2, 1.9 Hz, H-3), 3.78-3.71 (2H, m, H-5 and H-6b), 3.64 (1H, td, J = 9.4, 3.9 Hz, H-2), 3.43 (1H, t, J = 9.2 Hz, H-4), 3.47 (3H, s, OCH_3), 2.67 (1H, d, J = 2.0 Hz, OH), 2.24 (1H, d, J = 9.6 Hz, OH); ^{13}C NMR (101 MHz; $CDCl_3$) δ: 137.6 (4° C), 129.4 (CH), 128.5 (CH), 126.5 (CH), 102.1 (Ph*C*HO_2), 99.9 (C-1), 81.1 (C-4), 73.0 (C-2), 71.9 (C-3), 69.1 (C-6), 62.5 (C-5), 55.7 (OCH_3). Proton and carbon NMR were consistent with literature data.[15]

Methyl 4,6-*O*-benzylidene-2-*O*-trifluoromethanesulfonyl-α-D-glucopyranoside (S7)

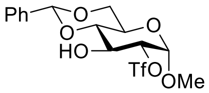

Following the reported synthesis,[16] distilled pyridine (10.5 mL, 130 mmol) was added to anhydrous CH_2Cl_2 (180 mL), cooled to -30 °C followed by the addition of triflic anhydride (6.0 mL, 36 mmol), the mixture was stirred under N_2. Glucopyranoside **S6** (9.295 g, 32.93 mmol) was dissolved in anhydrous CH_2Cl_2 (420 mL) and added to the reaction mixture which was stirred for 3 h. The reaction was quenched by dropwise addition of H_2O (12 mL) and washed with H_2O (2 x 300 mL). The organic phase was dried over $MgSO_4$, filtered, concentrated *in vacuo* and purified by column chromatography (98:2 to 7:3, PhMe:EtOAc) to afford glucoside **S7** as a white solid (9.891 g, 72%). ^{1}H NMR (400 MHz; $CDCl_3$) δ: 7.54-7.34 (5H, m, Ph), 5.54 (1H, s, PhC*H*O_2), 4.97 (1H, d, J = 3.7 Hz, H-1), 4.70 (1H, dd, J = 9.4, 3.7 Hz, H-2), 4.32 (1H, dd, J = 10.1, 4.8 Hz, H-6a), 4.30-4.23 (1H, m, H-3), 3.87 (1H, td, J = 9.9, 4.6 Hz, H-5), 3.75 (1H, t, J = 10.3 Hz, H-6b), 3.54 (1H, t, J = 9.4 Hz, H-4), 3.48 (3H, s, OCH_3), 2.66 (1H, d, J = 3.4 Hz, OH); ^{13}C NMR (101 MHz; $CDCl_3$) δ: 136.7 (4° C), 129.6 (CH), 128.6 (CH), 126.4 (CH), 118.6 (q, J = 318 Hz, CF_3), 102.3 (PhH*C*O_2), 97.7 (C-1), 84.2 (C-2), 81.2 (C-4), 68.8 (C-6), 68.3 (C-3), 62.1 (C-5), 56.0 (OCH_3); ^{19}F NMR (377 MHz; $CDCl_3$) δ: -74.53. Proton and carbon NMR were consistent with literature data.[16]

Methyl 4,6-*O*-benzylidene-2-deoxy-2-iodo-α-D-mannopyranoside (S8)

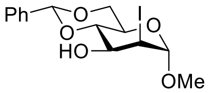

Following the reported synthesis,[16] glycosyl triflate **S7** (1.236 g, 2.983 mmol) and tetrabutylammonium iodide (3.542 g, 9.589 mmol) were added to a flask and purged with N_2 three times. Anhydrous PhMe (40 mL) was added and the mixture was heated at 80 °C for 4 h, after which the solution was concentrated *in vacuo*, diluted with Et_2O (70 mL), washed with $Na_2S_2O_{4\,(sat.\,aq.)}$ (50 mL), H_2O (2 x 50 mL), dried over Na_2SO_4, filtered and concentrated *in vacuo*. Following purification by column chromatography (9:1, Petrol ether (40-60):EtOAc) glycosyl iodide **S8** was obtained as a white solid (1.057 g, 90%). ^{1}H NMR (500 MHz; $CDCl_3$) δ: 7.51-7.46 (2H, m, Ph), 7.41-7.34 (3H, m, Ph), 5.60 (1H, s, PhC*H*O_2), 5.12 (1H, s, H-1), 4.52 (1H, dd, *J* = 4.3, 0.9 Hz, H-2), 4.28 (1H, dd, *J* = 9.5, 3.7 Hz, H-6a), 3.98-3.84 (3H, m, H-4, H-5, H-6b), 3.40 (3H, s, OCH_3), 3.34 (1H, dd, *J* = 8.5, 4.3 Hz, H-3), 2.58 (1H, br s, OH); ^{13}C NMR (126 MHz; $CDCl_3$) δ: 137.2 (4° C), 129.5 (CH), 128.5 (CH), 126.4 (CH), 103.9 (C-1), 102.4 (PhH*C*O_2), 81.5, 68.8 (C-6), 66.2 (C-3), 64.3, 55.4 (OCH_3), 36.2 (C-2). Proton and carbon NMR were consistent with literature data.[16]

4,6-*O*-Benzylidene-1,2-dideoxy-D-arabino-1-hexenopyranose (S9)

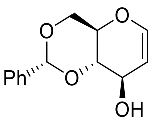

Following the reported synthesis,[16] glycosyl iodide **S8** (1.057 g, 2.695 mmol) was added to a flask and purged with N_2 three times, and dissolved in anhydrous CH_2Cl_2 (15 mL). Zinc (1.850 g, 28.29 mmol) and hydrogen hexachloroplatinate (IV) hexahydrate (11 mg, 0.021 mmol) were then added followed by AcOH (5 mL) and the mixture was stirred for 1 h. The mixture was diluted with CH_2Cl_2 (15 mL), sonicated for 5 min and filtered through Celite®. The solution was washed with $NaHCO_{3\,(sat.\,aq.)}$ (50 mL), dried over Na_2SO_4, filtered and concentrated *in vacuo*. Following purification by column chromatography (9:1 to 7:3, Petrol ether (40-60):EtOAc) glucal **S9** was obtained as a white solid (381 mg, 59%). ^{1}H NMR (500 MHz; $CDCl_3$) δ: 7.54-7.49 (2H, m, Ph), 7.42-7.33 (3H, m, Ph), 6.35 (1H, ddd, *J* = 6.1, 1.8, 0.6 Hz, H-1), 5.61 (1H, s, PhC*H*O_2), 4.78 (1H, dd, *J* = 6.2, 2.0 Hz, H-2), 4.54-4.46 (1H, m, H-3), 4.38 (1H, dd, *J* = 10.4, 5.0 Hz, H-6a), 3.93 (1H, td, *J* = 10.2, 5.1 Hz, H-5), 3.83 (1H, t,

J = 10.4 Hz, H-6b), 3.81 (1H, dd, J = 10.1, 7.6 Hz, H-4), 2.25 (1H, br s, OH); ^{13}C NMR (126 MHz; $CDCl_3$) δ: 144.3 (C-1), 137.2 (4° C), 129.5 (CH), 128.5 (CH), 126.4 (CH), 103.7 (C-2), 102.0 (PhH*C*O_2), 80.9 (C-4), 68.5 (C-5 and C-6), 66.8 (C-3). Proton and carbon NMR were consistent with literature data apart from H-4 and H-6b which have been reassigned on the basis of the HSQC NMR spectrum.[16]

4,6-*O*-Benzylidene-1,2-dideoxy-3-triisopropylsilyl-D-arabino-1-hexenopyranose (5g)

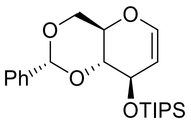

Following the reported synthesis,[17] glucal **S9** (502 mg, 2.14 mmol) and imidazole (245 mg, 3.60 mmol) were added to a flask and purged with N_2 three times, and dissolved in anhydrous DMF (4 mL). TIPSCl (0.80 mL, 3.7 mmol) was added dropwise to the solution was allowed to stir at room temperature for 16 h, the reaction was quenched with H_2O (0.5 mL). The solution was concentrated *in vacuo* and the residue obtained was dissolved in CH_2Cl_2 (10 mL), washed with H_2O (15 mL), dried over Na_2SO_4, filtered and concentrated *in vacuo*. Following purification by column chromatography (99:1 to 97:3, Hexane:EtOAc) glucal **5g** was obtained as a white solid (767 mg, 91%). ^{1}H NMR (400 MHz; $CDCl_3$) δ: 7.53-7.44 (2H, m, Ph), 7.40-7.30 (3H, m, Ph), 6.30 (1H, dd, J = 6.2, 1.6 Hz, H-1), 5.60 (1H, s, PhC*H*O_2), 4.73 (1H, dd, J = 6.2, 2.0 Hz, H-2), 4.62 (1H, dt, J = 7.0, 1.6 Hz, H-3), 4.35 (1H, dd, J = 10.3, 4.7 Hz, H-6a), 3.94-3.77 (3H, m, H-4, H-5, H-6b), 1.18-0.95 (21H, m, $OSi(CH(CH_3)_2)_3$); ^{13}C NMR (101 MHz; $CDCl_3$) δ: 143.3 (C-1), 137.4 (4° C), 129.0 (CH), 128.2 (CH), 126.3 (CH), 105.8 (C-2), 101.7 (PhH*C*O_2), 81.1 (C-4), 68.9 (C-5), 68.7 (C-6), 67.4 (C-3), 18.2 ($OSi(CH(CH_3)(CH_3))$), 18.1 ($OSi(CH(CH_3)(CH_3))$), 12.5 ($OSi(CH(CH_3)_2)_3$). Proton and carbon NMR were consistent with literature data.[17]

6-*O*-Benzyl-1,2-dideoxy-3,4-*O*-(1,1,3,3-tetraisopropyldisiloxane-1,3-diyl)-D-arabino-1-hexenopyranose (5h)

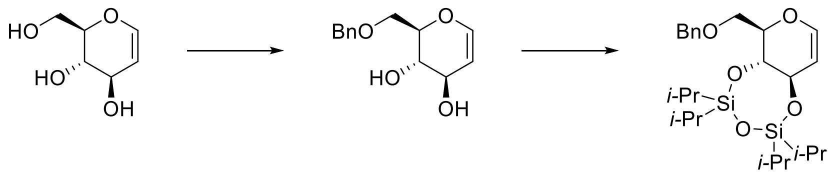

S1 S10 5h

D-Glucal **S1** (2.065 g, 14.13 mmol) which had been pre-dried under vacuum for 1 h was dissolved in anhydrous DMF (40 mL) and cooled to -40 °C. A 1 M THF solution of LiHMDS (15 mL, 15 mmol) was added dropwise, followed by the dropwise addition BnBr (1.6 mL, 13 mmol). The solution was then allowed to warm to 0 °C over a period of 6 h. $NH_4Cl_{\text{(sat. aq.)}}$ (10 mL) and EtOAc (50 mL) were added and the phases separated, the aqueous phase was extracted with EtOAc (3 x 15 mL). The combined organic phase were washed with brine (50 mL), dried over Na_2SO_4, filtered and concentrated *in vacuo*. Following purification by column chromatography (1:1 Hexane:EtOAc) glucal **S10** was obtained as a solid (989 mg, 30%). ^{1}H NMR (400 MHz; $CDCl_3$) δ: 7.40-7.27 (5H, m, Ph), 6.34 (1H, dd, *J* = 6.0, 1.8 Hz), 4.73 (1H, dd, *J* = 6.1, 2.2 Hz, H-2), 4.65 (1H, d, *J* = 12.1 Hz, OC*H*HPh), 4.56 (1H, d, *J* = 12.1 Hz, OCH*H*Ph), 4.24 (1H, dt, *J* = 7.0, 2.0 Hz, H-3), 3.91 (1H, dt, *J* = 9.7, 4.1 Hz, H-5), 3.83 (1H, dd, *J* = 10.7, 4.2 Hz, H-6a), 3.788 (1H, dd, *J* = 10.7, 4.0 Hz, H-6b), 3.786 (1H, dd, *J* = 9.7, 7.1 Hz, H-4), 2.91 (1H, br m, OH), 2.35 (1H, br m, OH). Proton NMR was consistent with literature data.[18] Glucal **S10** (973 mg, 4.12 mmol) and imidazole (2.310 g, 33.93 mmol), that had been pre-dried under vacuum for 1 h, were dissolved in anhydrous DMF (40 mL) and cooled to 0 °C. Dichloro-1,1,3,3-tetraisopropyldisiloxane (1.4 mL, 4.4 mmol) was added dropwise and the solution was then allowed to warm to room temperature and stirred for 5 h. The reaction was quenched with H_2O (2 mL) and concentrated *in vacuo*. The crude residue was dissolved in CH_2Cl_2 (40 mL) washed with H_2O (40 mL), the aqueous phase was back extracted with CH_2Cl_2 (2 x 20 mL) and the combined organic phases were dried over Na_2SO_4, filtered and concentrated *in vacuo*. Following purification by column chromatography (98:2 to 96:3 Hexane:EtOAc) glucal **5h** was obtained as an oil (1.644 g, 83%). ^{1}H NMR (400 MHz; $CDCl_3$) δ: 7.37-7.24 (5H, m, Ph), 6.36 (1H, dd, *J* = 6.0, 1.8 Hz, H-1), 4.66 (1H, ddd, *J* = 6.0, 2.0, 0.6 Hz, H-2), 4.65 (1H, d, *J* = 12.1 Hz, PhC*H*HO), 4.56 (1H, d, *J* = 12.1 Hz, PhCH*H*O), 4.41 (1H, dtd, *J* = 6.3, 1.9, 0.9 Hz, H-3), 3.95-3.83 (3H, m, H-4, H-5, H-6a), 3.72 (1H, dd, *J* = 10.5, 5.3 Hz, H-6b), 1.16-0.86 (28H, m, 4 x

Si(C*H*(C*H*$_3$)$_2$); ^{13}C NMR (101 MHz; CDCl$_3$) δ: 144.0 (C-1), 138.1 (4° C), 128.5 (CH), 127.9 (CH), 127.7 (CH), 103.6 (C-2), 78.1 (CH), 73.7 (Ph*C*H$_2$O), 72.8 (CH), 72.6 (CH), 69.3 (C-6), 17.5 (Si(CH(*C*H$_3$)(CH$_3$)), 17.47 (Si(CH(*C*H$_3$)(CH$_3$)), 17.45 (Si(CH(*C*H$_3$)(CH$_3$)), 17.38 (Si(CH(*C*H$_3$)(CH$_3$)), 17.37 (Si(CH(*C*H$_3$)(CH$_3$)), 17.31 (Si(CH(*C*H$_3$)(CH$_3$)), 13.19 (Si(*C*H(CH$_3$)$_2$), 13.02 (Si(*C*H(CH$_3$)$_2$), 12.61 (Si(*C*H(CH$_3$)$_2$), 12.47 (Si(*C*H(CH$_3$)$_2$). MALDI-HRMS for $C_{25}H_{42}NaO_5Si_2^+$ (M+Na)$^+$ calculated: 501.2463; found: 501.2465. $[\alpha]_D^{21}$ = +15 (*c* 1.0, CHCl$_3$).

1,2-Dideoxy-6-*O*-triisopropylsilyl-D-arabino-1-hexenopyranose (S11)

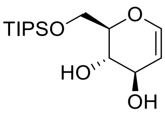

D-Glucal **S1** (2.511 g, 17.18 mmol) and imidazole (2.35 g, 34.5 mmol) which had been pre-dried under vacuum in a flame-dried flask for 1 h, were dissolved in anhydrous DMF (40 mL) under a N_2 atmosphere and the solution was cooled to 0 °C. TIPSCl (4.7 mL, 22 mmol) was added dropwise, the reaction was allowed to warm to room temperature and stir for 18 h. The reaction was concentrated *in vacuo* and the residue was dissolved in CH_2Cl_2 (100 mL), washed with H_2O (100 mL), the aqueous phase was then washed with CH_2Cl_2 (2 x 50 mL) and the combined organic phases were washed with brine (100 mL), dried over $MgSO_4$, filtered and concentrated *in vacuo*. Following purification by column chromatography (95:5 to 7:3 Petrol ether (40-60):EtOAc) glucal **S11** was obtained as a white solid (3.839 g, 74%). ^{1}H NMR (400 MHz; CDCl$_3$) δ: 6.30 (1H, dd, *J* = 6.1, 1.8 Hz, H-1), 4.73 (1H, ddd, *J* = 6.1, 2.2 Hz, H-2), 4.31-4.25 (1H, m, H-3), 4.11-4.06 (1H, m, H6-a), 4.01-3.95 (1H, m, H6-b), 3.86-3.79 (2H, m, H-4, H-5), 3.35 (1H, d, *J* = 2.1 Hz, OH), 2.38 (1H, d, *J* = 5.4 Hz, OH), 1.19-1.04 (21H, m, 3 x Si(C*H*(C*H*$_3$)$_2$); ^{13}C NMR (101 MHz; CDCl$_3$) δ: 144.2 (C-1), 102.6 (C-2), 76.2, 73.3 (C-4, C-5), 69.5 (C-3), 64.9 (C-6), 18.03 (Si(CH(*C*H$_3$)(CH$_3$)), 18.01 (Si(CH(*C*H$_3$)(CH$_3$)), 11.9 (Si(*C*H(CH$_3$)$_2$). Proton and carbon NMR were consistent with literature data.[3]

1,2-Dideoxy-3,4-*O*-(1,1,3,3-tetraisopropyldisiloxane-1,3-diyl)-6-*O*-triisopropylsilyl-D-arabino-1-hexenopyranose (5i)

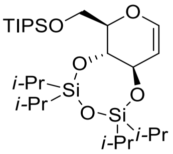

Glucal **S11** (1.02 g, 3.37 mmol) and imidazole (437 mg, 6.42 mmol) which had been pre-dried under vacuum in a flame-dried flask for 1 h, were dissolved in distilled pyridine (30 mL) under a N_2 atmosphere and the solution was cooled to 0 °C. 1,3-Dichloro-1,1,3,3-tetraisopropyldisiloxane (1.6 mL, 5.00 mmol) was added dropwise and the solution was then allowed to warm to room temperature and stirred for 18 h. The reaction was quenched with H_2O (30 mL), extracted with EtOAc (60 mL). The organic layer was washed with brine (20 mL), dried over $MgSO_4$, filtered and concentrated *in vacuo*. Following purification by column chromatography (99.5:0.5 to 98:2 Petrol ether (40-60):EtOAc) glucal **5i** was obtained as an oil (1.76 g, 96%). ^{1}H NMR (400 MHz; $CDCl_3$) δ: 6.34 (1H, dd, J = 6.0, 1.7 Hz, H-1), 4.62 (1H, dd, J = 6.0, 2.0 Hz, H-2), 4.41 (1H, dt, J = 6.8, 1.9 Hz, H-3), 4.12 (1H, dd, J = 11.1, 1.9 Hz, H-6a), 3.92 (1H, dd, J = 11.1, 5.5 Hz, H-6b), 3.86 (1H, dd, J = 10.2, 6.8 Hz, H-4), 3.77 (1H, ddd, J = 10.0, 5.5, 1.8 Hz, H-5), 1.16-0.91 (49H, m, 7 x Si(C*H*(C*H*$_3$)$_2$); ^{13}C NMR (101 MHz; $CDCl_3$) δ: 144.1 (C-1), 103.0 (C-2), 79.6 (C-5), 72.9 (C-3), 72.4 (C-4), 63.1 (C-6), 18.13 (Si(CH(*C*H$_3$)(CH$_3$)), 18.09 (Si(CH(*C*H$_3$)(CH$_3$)), 17.8 (Si(CH(*C*H$_3$)(CH$_3$)), 17.62 (Si(CH(*C*H$_3$)(CH$_3$)), 17.56 (Si(CH(*C*H$_3$)(CH$_3$)), 17.48 (Si(CH(*C*H$_3$)(CH$_3$)), 17.47 (Si(CH(*C*H$_3$)(CH$_3$)), 17.41 (Si(CH(*C*H$_3$)(CH$_3$)), 17.36 (Si(CH(*C*H$_3$)(CH$_3$)), 17.31 (Si(CH(*C*H$_3$)(CH$_3$)), 13.25 (Si(*C*H(CH$_3$)$_2$), 13.0 (Si(*C*H(CH$_3$)$_2$), 12.7 (Si(*C*H(CH$_3$)$_2$), 12.5 (Si(*C*H(CH$_3$)$_2$), 12.2 (Si(*C*H(CH$_3$)$_2$). ESI-HRMS for $C_{27}H_{56}NaO_5Si_3^+$ (M+Na)$^+$ calculated: 567.3328; found: 567.3333. $[\alpha]_D^{21}$ = +22 (*c* 1.0, $CHCl_3$).

1,2-Dideoxy-3,4-*O*-(1,1,3,3-tetramethyldisiloxane-1,3-diyl)-6-*O*-triisopropylsilyl-D-arabino-1-hexenopyranose (5j)

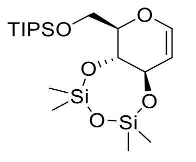

6-*O*-Triisopropylsilyl-D-glucal **S11** (700 mg, 2.31 mmol) and imidazole (1.556 g, 22.86 mmol) which had been pre-dried under vacuum in a flame-dried flask for 1 h, were dissolved in DMF (80 mL) under a N_2 atmosphere and the solution was cooled to 0 °C. 1,3-Dichloro-1,1,3,3-tetramethyldisiloxane (470 μL, 2.40 mmol) was added dropwise and the solution was then allowed to warm to room temperature and stirred for 3 h. The reaction was quenched with H_2O (1 mL) and concentrated *in vacuo*. The crude residue was dissolved in CH_2Cl_2 (25 mL) washed with H_2O (25 mL), the aqueous phase was back extracted with CH_2Cl_2 (2 x 10 mL) and the combined organic phases were dried over Na_2SO_4, filtered and concentrated *in vacuo*. Following purification by column chromatography (95:5 Hexane:EtOAc) the glucal **5j** was obtained as an oil (516 mg, 52%). ^{1}H NMR (400 MHz; $CDCl_3$) δ: 6.34 (1H, dd, J = 6.0, 1.9 Hz, H-1), 4.61 (1H, dd, J = 6.0, 1.9 Hz, H-2), 4.44 (1H, dt, J = 7.2, 1.9 Hz, H-3), 4.02-3.91 (3H, m, H-4, H-6a, H-6b), 3.78 (1H, dt, J = 10.1, 2.8 Hz, H-5), 1.17-0.96 (21H, m, Si(C*H*(C*H*$_3$)$_2$), 0.203 (3H, s, $SiCH_3$), 0.195 (3H, s, $SiCH_3$), 0.18 (3H, s, $SiCH_3$), 0.15 (3H, s, $SiCH_3$); ^{13}C NMR (101 MHz; $CDCl_3$) δ: 144.5 (C-1), 102.6 (C-2), 79.1 (CH), 72.6 (CH), 71.3 (CH), 62.2 (C-6), 18.2 (Si(CH(*C*H$_3$)(CH$_3$)), 18.1 (Si(CH(*C*H$_3$)(CH$_3$)), 12.2 (Si(*C*H(CH$_3$)$_2$), -0.8 ($SiCH_3$), -1.0 ($SiCH_3$), -2.00 ($SiCH_3$), -2.1 ($SiCH_3$). ESI-HRMS for $C_{19}H_{40}NaO_5Si_3^+$ $(M+Na)^+$ calculated: 455.2076; found: 455.2078.

3,4-*O*-(1,1,3,3-Tetraisopropyldisiloxane-1,3-diyl)-1,2,6-trideoxy-L-arabino-1-hexenopyranose (9)

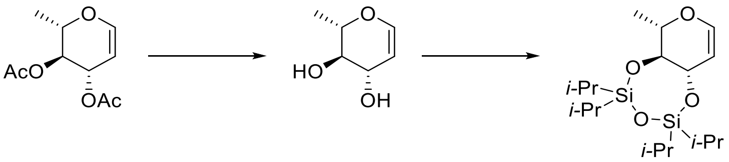

S12 **S13** **9**

3,4-*O*-Di-acetyl-L-rhamnal **S12** (520 mg, 2.43 mmol) was dissolved in a solution of MeOH (8 mL), H_2O (1 mL) and Et_3N (1 mL) and stirred for 18 h after which the solvents were removed to afford L-rhamnal **S13** as a solid. L-Rhamnal **S13** and imidazole (332 mg, 4.88 mmol) which had been pre-dried under vacuum for 1 h, were dissolved in distilled pyridine (20 mL) under a N_2 atmosphere and the solution was cooled to 0 °C. 1,3-Dichloro-1,1,3,3-tetraisopropyldisiloxane (1.2 mL, 3.75 mmol) was added dropwise and the solution was then allowed to warm to room temperature and stirred for 18 h. The reaction was quenched with H_2O (30 mL), extracted with EtOAc (50 mL). The organic layer was washed with brine (20 mL), dried over $MgSO_4$, filtered and concentrated *in vacuo*. Following purification by column chromatography (1:0 to 96:4 Hexane:EtOAc) rhamnal **9** was obtained as a white solid (846 mg, 96%). ^{1}H NMR (400 MHz; $CDCl_3$) δ: 6.29 (1H, ddd, *J* = 6.1, 1.8, 0.5 Hz, H-1), 4.65 (1H, dd *J* = 6.0, 2.1 Hz, H-2), 4.36 (1H, dt, *J* = 7.0, 1.9 Hz, H-3), 3.82 (1H, dqd, *J* = 9.9, 6.3, 0.5 Hz, H-5), 3.58 (1H, dd, *J* = 9.9, 7.0 Hz, H-4), 1.36 (3H, d, *J* = 6.3 Hz, H-6), 1.12-0.91 (28H, 4 x Si(C*H*(C*H*$_3$)$_2$); ^{13}C NMR (101 MHz; $CDCl_3$) δ: 144.2 (C-1), 103.9 (C-2), 77.4 (C-4), 75.4 (C-5), 72.8 (C-3), 17.80, 17.76, 17.57, 17.55, 17.47, 17.46, 17.41, 17.40, 17.39 (C-6, 8 x Si(CH(*C*H$_3$)(CH$_3$)), 13.2 (Si(*C*H(CH$_3$)$_2$), 13.1 (Si(*C*H(CH$_3$)$_2$), 12.6 (Si(*C*H(CH$_3$)$_2$), 12.5 (Si(*C*H(CH$_3$)$_2$). ESI-HRMS for $C_{18}H_{36}NaO_4Si_2^+$ $(M+Na)^+$ calculated: 372.2044; found: 372.2045. $[\alpha]_D^{21}$ = -5 (*c* 1.0, $CHCl_3$).

4-*O*-Acetyl-3-*O*-*tert*-butyldimethylsilyl-1,2,6-trideoxy-L-arabino-1-hexenopyranose (10)

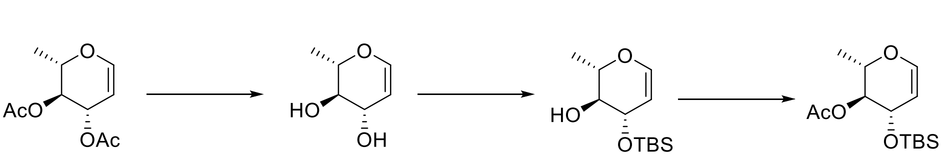

S12 **S13** **S13b** **10**

3,4-*O*-Di-acetyl-L-rhamnal **S12** (1.056 g, 4.930 mmol) was dissolved in a solution of MeOH (8 mL), H_2O (1 mL) and Et_3N (1 mL) and stirred for 18 h after which the solvents were removed

to yield L-rhamnal **S13**. L-Rhamnal **S13** and imidazole (744 mg, 10.9 mmol) which had been pre-dried under vacuum for 1 h, were dissolved in anhydrous DMF (10 mL), cooled to -20 °C followed by the addition of TBSCl (890 mg, 5.90 mmol) and stirred at this temperature for 30 min, and then stirred at 0 °C for 5 h. The mixture was concentrated *in vacuo*, the obtained residue was dissolved in CH_2Cl_2 (30 mL), washed with H_2O (30 mL), dried over Na_2SO_4, filtered and concentrated *in vacuo*. The residue was dissolved in Et_2O (30 mL) and filtered through silica and concentrated *in vacuo*. Without further purification, the dried residue containing **S13b** was dissolved in a solution of pyridine (6 mL) and Ac_2O (3 mL) and stirred at room temperature for 18 h before being diluted with CH_2Cl_2 (25 mL). The solution was washed with 2 M HCl (25 mL), $NaHCO_{3\ (sat.\ aq.)}$ (25 mL), dried over Na_2SO_4, filtered and concentrated *in vacuo*. The residue was purified by column chromatography (98:2 to 96:4, Hexane:EtOAc) to afford **10** as an oil (988 mg, 70%). ^{1}H NMR (400 MHz; $CDCl_3$) δ: 6.30 (1H, dd, J = 6.1, 1.4 Hz, H-1), 4.91 (1H, dd, J = 8.1, 6.1 Hz, H-4), 4.67 (1H, dd, J = 6.2, 2.8 Hz, H-2), 4.25 (1H, dddd, J = 6.1, 2.8, 1.5, 0.6 Hz, H-3), 4.05-3.97 (1H, m, H-5), 2.10 (3H, s, (C=O)CH_3), 1.29 (3H, d, J = 6.5 Hz, H-6), 0.87 (6H, s, $SiC(CH_3)_3$), 0.08 (3H, s, $SiCH_3$), 0.06 (3H, s, $SiCH_3$); ^{13}C NMR (101 MHz; $CDCl_3$) δ: 170.0 (C=O), 143.6 (C-1), 103.4 (C-2), 75.1 (C-4), 72.8 (C-5), 66.8 (C-3), 25.8 ($SiC(\mathit{C}H_3)_3$), 21.2 ((C=O)$\mathit{C}H_3$), 18.1 ($Si\mathit{C}(CH_3)_3$), 16.9 (C-6), -4.4 ($SiCH_3$), -4.7 ($SiCH_3$). Proton NMR was consistent with literature data. (Kopper, S.; Springer, D.; Thiem, J., *J. Carbohydr. Chem.* **1994**, *13*, 1065-1070.)

1,2-Dideoxy-[2-^{2}H]-3,4,6-tri-*O*-acetyl-D-arabino-1-hexenopyranose (S16)

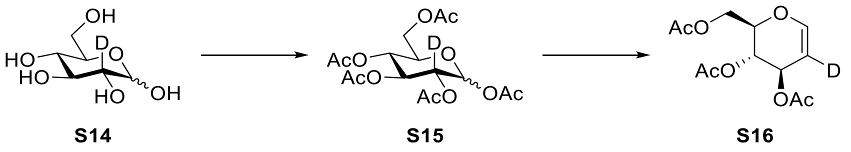

[2-^{2}H]-D-Glucose **S14** (1.005 g, 5.548 mmol) was dissolved in a solution of pyridine (12 mL), Ac_2O (6 mL), DMAP (60 mg, 0.49 mmol) was added and the solution was stirred for 18 h. The mixture was then diluted with CH_2Cl_2 (100 mL), washed with 1 M HCl (2 x 50 mL), $NaHCO_3$ $_{(sat.\ aq.)}$ (2 x 50 mL), brine (100 mL), dried over $MgSO_4$ filtered and concentrated *in vacuo* by azeotroping with PhMe to yield deuterated glucose **S15** as a solid (2.147 g, 99%), which was used without further purification on the next step: α:β = 1:0.33. ^{1}H NMR (500 MHz; $CDCl_3$) **α-anomer**: δ: 6.32 (1H, s, H-1), 5.46 (1H, d, *J* = 9.5 Hz, H-3), 5.14 (1H, dd, *J* = 10.2, 9.6 Hz, H-4), 4.31-4.23 (1H, m, H-6a), 4.14-4.06 (2H, m, H-5, H-6b), 2.18 (3H, s, CH_3), 2.09 (3H, s, CH_3), 2.04 (3H, s, CH_3), 2.02 (3H, s, CH_3), 2.01 (3H, s, CH_3); ^{13}C NMR (126 MHz; $CDCl_3$) δ: 170.5 (C=O), 170.26 (C=O), 170.25 (C=O), 170.0 (C=O), 169.0 (C=O), 89.8 (C-1), 68.9 (C-5), 67.6, 67.4 (C-3 and C-4), 61.4 (C-6), 21.0 (CH_3), 20.80 (CH_3), 20.78 (CH_3), 20.75 (CH_3), 20.7 (CH_3). ESI-HRMS for $C_{16}H_{21}DNaO_{11}^+$ $(M+Na)^+$ calculated: 414.1117; found: 414.1109. Following the reported methodology,[19] deuterated glucose **S15** (1.002 g, 2.560 mmol) was dissolved in anhydrous CH_2Cl_2 (8 mL) under N_2, the solution was cooled to 0 °C and 33% HBr/AcOH (3.6 mL) was added dropwise. After complete addition the solution was allowed to warm to room temperature and stir for 1 h, after which the solution was added to ice H_2O (20 mL) and extracted with EtOAc (80 mL) and washed with $NaHCO_3$ $_{(sat.\ aq.)}$ (2 x 30 mL), H_2O (60 mL), brine (60 mL) and dried over $MgSO_4$. The solution was filtered and concentrated *in vacuo* to 4 mL and added to a solution of zinc dust (1.046 g, 16.00 mmol), NaOAc (1.000 g, 12.20 mmol), $CuSO_4$ (27 mg, 0.17 mmol) in 60% $AcOH/H_2O$ (4 mL). After 3 h of stirring at room temperature the reaction mixture was filtered through Celite® which was rinsed with EtOAc (20 mL). The filtrate was diluted with EtOAc (60 mL), washed with $NaHCO_3$ $_{(sat.\ aq.)}$ (60 mL), H_2O (60 mL), brine (60 mL), dried over $MgSO_4$, filtered and concentrated *in vacuo*. Following purification by column chromatography (8:2 to 6:4, Hexane:Et_2O) deuterated glucal **S16** was obtained as a syrup (502 mg, 72%). ^{1}H NMR (500 MHz; $CDCl_3$) δ: 6.46 (1H, d, *J* = 1.3 Hz, H-1), 5.33 (1H, m, H-3), 5.22 (1H, dd, *J* = 7.6, 5.7 Hz, H-4), 4.39 (1H, dd, *J* = 12.1, 5.8 Hz, H-6a), 4.25 (1H, m, H-5), 4.19 (1H, dd, *J* = 12.1, 3.1 Hz, H-6b), 2.09 (3H, s, CH_3), 2.07 (3H, s, CH_3), 2.04 (3H, s,

CH_3); ^{13}C NMR (126 MHz; $CDCl_3$) δ: 170.7 (C=O), 170.5 (C=O), 169.7 (C=O), 145.7 (C-1), 74.1 (C-5), 67.5 (C-3), 67.3 (C-4), 61.5 (C-6), 21.1 (CH_3), 21.0 (CH_3), 20.9 (CH_3). ESI-HRMS for $C_{16}H_{21}DNaO_{11}^+$ $(M+Na)^+$ calculated: 296.0851; found: 296.0846. $[\alpha]_D^{23}$ = -18 (*c* 1.0, $CHCl_3$).

[2-^{2}H]-1,2-Dideoxy-3,4-*O*-(1,1,3,3-tetraisopropyldisiloxane-1,3-diyl)-6-*O*-triisopropylsilyl-D-arabino-1-hexenopyranose (13)

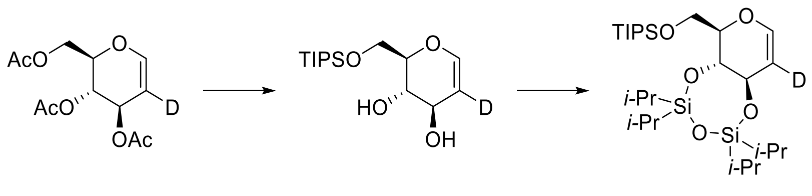

Deuterated glucal **S16** (502 mg, 1.84 mmol) was dissolved in MeOH/Et_3N/H_2O (8:1:1, 10 mL) and stirred for 18 h. The reaction mixture was concentrated *in vacuo*, imidazole (257 mg, 3.77 mmol) was added and then placed under vacuum for 1 h. Anhydrous DMF (5 mL) was added under a N_2 atmosphere and the solution was cooled to 0 °C, followed by the dropwise addition of TIPSCl (0.50 mL, 2.3 mmol), the reaction was allowed to warm to room temperature and stir for 18 h. The reaction was concentrated *in vacuo* and the residue was dissolved in CH_2Cl_2 (25 mL), washed with H_2O (25 mL), the aqueous phase was then washed with CH_2Cl_2 (2 x 10 mL) and the combined organic phases were dried over $MgSO_4$, filtered and concentrated *in vacuo*. Following purification by column chromatography (95:5 to 7:3 Hexane:EtOAc) deuterated glucal **S17** was obtained as a syrup (383 mg, 69%). ^{1}H NMR (400 MHz; $CDCl_3$) δ: 6.30 (1H, br d, J = 1.7 Hz, H-1), 4.31-4.24 (1H, m, H-3), 4.12-4.06 (1H, m, H6-a), 4.01-3.95 (1H, m, H6-b), 3.87-3.79 (2H, m, H-4, H-5), 3.38 (1H, br s, OH), 2.43 (1H, br s, OH), 1.20-1.02 (21H, m, 3 x Si(C*H*(C*H*$_3$)$_2$); ^{13}C NMR (101 MHz, $CDCl_3$) δ: 144.1 (C-1), 76.3, 73.2 (C-4, C-5), 69.4 (C-3), 64.9 (C-6), 18.0 (Si(CH(*C*H$_3$)(CH$_3$)), 18.0 (Si(CH(*C*H$_3$)(CH$_3$)), 11.9 (Si(*C*H(CH$_3$)$_2$). MALDI-HRMS for $C_{15}H_{29}DNaO_4Si^+$ $(M+Na)^+$ calculated: 326.1868; found: 326.1871. Deuterated glucal **S17** (383 mg, 1.26 mmol) and imidazole (713 mg, 10.5 mmol), that had been pre-dried under vacuum for 1 h, were dissolved in anhydrous DMF (10 mL) and cooled to 0 °C. Dichloro-1,1,3,3-tetraisopropyldisiloxane (0.43 mL, 1.3 mmol) was added dropwise and the solution was then allowed to warm to room temperature and stirred for 3 h. The reaction was quenched with

H_2O (0.5 mL) and concentrated *in vacuo*. The crude residue was dissolved in CH_2Cl_2 (25 mL) washed with H_2O (25 mL), the aqueous phase was back extracted with CH_2Cl_2 (2 x 10 mL) and the combined organic phases were dried over Na_2SO_4, filtered and concentrated *in vacuo*. Following purification by column chromatography (99.5:0.5 to 98:2 Hexane:EtOAc) deuterated glucal **13** was obtained as an oil (557 mg, 81%). ^{1}H NMR (400 MHz; $CDCl_3$) δ: 6.34 (1H, d, *J* = 1.8 Hz, H-1), 4.41 (1H, dd, *J* = 6.8, 1.9 Hz, H-3), 4.12 (1H, dd, *J* = 11.1, 1.9 Hz, H-6a), 3.92 (1H, dd, *J* = 11.1, 5.5 Hz, H-6b), 3.86 (1H, dd, *J* = 10.2, 6.8 Hz, H-4), 3.77 (1H, ddd, *J* = 10.2, 5.5, 1.9 Hz, H-5), 1.17-0.88 (49H, m, 7 x Si(C*H*(C*H*$_3$)$_2$); ^{13}C NMR (101 MHz; $CDCl_3$) δ: 144.1 (C-1), 79.6 (C-5), 72.8 (C-3), 72.4 (C-4), 63.1 (C-6), 18.14 (Si(CH(*C*H$_3$)(CH$_3$)), 18.10 (Si(CH(*C*H$_3$)(CH$_3$)), 17.8 (Si(CH(*C*H$_3$)(CH$_3$)), 17.62 (Si(CH(*C*H$_3$)(CH$_3$)), 17.56 (Si(CH(*C*H$_3$)(CH$_3$)), 17.48 (Si(CH(*C*H$_3$)(CH$_3$)), 17.47 (Si(CH(*C*H$_3$)(CH$_3$)), 17.41 (Si(CH(*C*H$_3$)(CH$_3$)), 17.36 (Si(CH(*C*H$_3$)(CH$_3$)), 17.3 (Si(CH(*C*H$_3$)(CH$_3$)), 13.3 (Si(*C*H(CH$_3$)$_2$), 13.1 (Si(*C*H(CH$_3$)$_2$), 12.7 (Si(*C*H(CH$_3$)$_2$), 12.5 (Si(*C*H(CH$_3$)$_2$), 12.2 (Si(*C*H(CH$_3$)$_2$). MALDI-HRMS for $C_{27}H_{55}DNaO_5Si_3^+$ $(M+Na)^+$ calculated: 568.3391; found: 568.3398. $[\alpha]_D^{21}$ = +21 (*c* 1.0, $CHCl_3$).

Acceptor Synthesis

Methyl 2,3,4-tri-*O*-benzyl-α-D-glucopyranoside (3a)

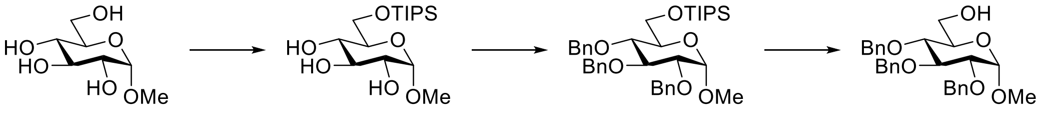

S5 **S18** **S19** **3a**

Methyl α-D-glucopyranoside **S5** (1.06 g, 5.46 mmol), which had been pre-dried under vacuum in a flame-dried flask for 1 h, was dissolved in anhydrous DMF (20 mL) under a N_2 atmosphere. Imidazole (940 mg, 13.8 mmol) was added and the solution was cooled to 0 °C, followed by the dropwise addition of TIPSCl (1.2 mL, 5.6 mmol) and then stirred at room temperature for 16 h. The solution was concentrated *in vacuo* and the residue was dissolved in CH_2Cl_2 (50 mL) washed with H_2O (40 mL), brine (40 mL), dried over $MgSO_4$ filtered and concentrated *in vacuo* to afford **S18**. The **S18** mixture was then dissolved in anhydrous DMF (50 mL) and cooled to 0 °C. NaH (60% in mineral oil, 1.25 g, 31.3 mmol) was added and the solution was then stirred at room temperature for 30 min, after which the mixture was cooled to 0 °C and BnBr (5.2 mL, 44 mmol) was added dropwise. The solution was then stirred at

room temperature for 16 h, at which time MeOH (5 mL) was added and the solution concentrated *in vacuo* and the residue was dissolved in CH_2Cl_2 (50 mL) washed with H_2O (40 mL), brine (40 mL), dried over $MgSO_4$ filtered and concentrated *in vacuo* to afford **S19**. The **S19** residue was dissolved in a 1 M THF solution of TBAF (15 mL, 15 mmol) and stirred for 2 h at room temperature, after which the reaction was diluted with CH_2Cl_2 (50 mL) and washed with H_2O (40 mL), brine (40 mL), dried over $MgSO_4$, filtered and concentrated *in vacuo*. Following purification by column chromatography (99:1 to 7:3, Petrol ether (40-60):EtOAc) product **3a** was obtained as a white solid (2.17 g, 86%). ^{1}H NMR (400 MHz; $CDCl_3$) δ: 7.40-7.27 (15H, m, Ph), 4.99 (1H, d, *J* = 10.9 Hz, PhC*H*H), 4.89 (1H, d, *J* = 11.0 Hz, PhC*H*H), 4.84 (1H, d, *J* = 10.9 Hz, PhCH*H*), 4.80 (1H, d, *J* = 12.1 Hz, PhC*H*H), 4.66 (1H, d, *J* = 12.1 Hz, PhCH*H*), 4.64 (1H, d, *J* = 10.9 Hz, PhCH*H*), 4.57 (1H, d, *J* = 3.5 Hz, H-1), 4.01 (1H, t, *J* = 9.3 Hz, H-3), 3.80-3.62 (3H, m, H-5, H6a, H6b), 3.52 (1H, t, *J* = 9.0 Hz, H-4), 3.50 (1H, dd, *J* = 9.7, 3.6 Hz, H-2), 3.37 (3H, s, OCH_3), 1.63 (1H, br t, *J* = 5.8 Hz, OH); ^{13}C NMR (101 MHz; $CDCl_3$) δ: 138.9 (4° C), 138.27 (4° C), 138.25 (4° C), 128.63 (CH), 128.61 (CH), 128.55 (CH), 128.3 (CH), 128.2 (CH), 127.11 (CH), 128.08 (CH), 128.0 (CH), 127.8 (CH), 98.3 (C-1), 82.1 (C-3), 80.1 (C-2), 77.5 (C-4), 75.9 (Ph*C*H_2), 75.2 (Ph*C*H_2), 73.6 (Ph*C*H_2), 70.8 (C-5), 62.0 (C-6), 55.3 (OCH_3). Proton and carbon NMR were consistent with literature data.[20]

Phenyl 2,3,4-tri-*O*-benzoyl-6-*O*-triisopropylsilyl-β-D-thioglucopyranoside (S24)

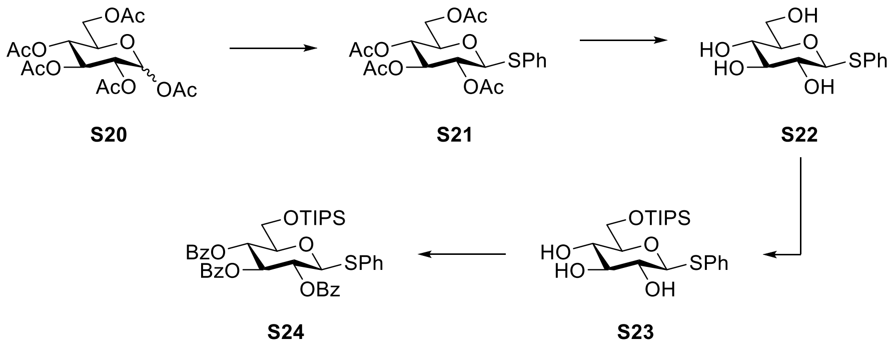

Following a reported synthesis,[21] penta-*O*-acetyl-D-glucopyranoside **S20** (4.70 g, 12.0 mmol) and zinc iodide (11.45 g, 36 mmol), that had been pre-dried under vacuum in a flame-dried flask for 1 h, was dissolved in anhydrous CH_2Cl_2 (120 mL) under a N_2 atmosphere, TMSSPh (6.8 mL, 36 mmol) was added and the mixture was stirred at room temperature for 6 h. The solution was filtered through Celite®, diluted with CH_2Cl_2 (50 mL), washed with 1 M HCl

(100 mL), $NaHCO_3$ (sat. aq.) (2 x 100 mL), brine (100 mL), dried over $MgSO_4$, filtered and concentrated *in vacuo*. Following purification by column chromatography (95:5 to 8:2, Hexane:EtOAc), the product **S21** was dissolved in a solution of MeOH (80 mL), H_2O (10 mL) and Et_3N (10 mL) and stirred for 18 h after which the solvents were removed to yield the deacetylated thioglycoside **S22**. Thioglycoside **S22** and imidazole (1.24 g, 18.2 mmol) were dissolved in anhydrous DMF (80 mL) under N_2 and TIPSCl (3.9 mL, 18 mmol) was added and the reaction was stirred at room temperature for 24 h. The solution was concentrated *in vacuo* and the residue was dissolved in EtOAc (100 mL) and washed with H_2O (100 mL). The aqueous phase was washed with EtOAc (2 x 100 mL). The combined organics were dried over $MgSO_4$, filtered and concentrated *in vacuo* to afford **S23**. The TIPS protected thioglycoside **S23** was then dissolved in pyridine (60 mL), followed by the addition of BzCl (6.3 mL, 54 mmol) and DMAP (146 mg, 1.20 mmol) and the solution was then stirred at room temperature for 4 h. MeOH was added at 0 °C followed by co-evaporation of the solvent with PhMe, the resulting residue was dissolved in CH_2Cl_2 (150 mL) washed with H_2O (2 x 50 mL), brine (50 mL), dried over $MgSO_4$, filtered and concentrated *in vacuo*. Following purification by column chromatography (99:1 to 9:1, Petrol ether (40-60):EtOAc) product **S24** was obtained as an oil (7.73 g, 87%). ^1H NMR (400 MHz; $CDCl_3$) δ: 7.99-7.93 (2H, m, Ph), 7.93-7.89 (2H, m, Ph), 7.82-7.77 (2H, m, Ph), 7.56-7.22 (14H, m, Ph), 5.87 (1H, t, *J* = 9.5 Hz, H-3), 5.54 (1H, t, *J* = 9.5 Hz, H-4), 5.46 (1H, dd, *J* = 10.0, 9.6 Hz, H-2), 5.05 (1H, d, *J* = 10.0 Hz, H-1), 3.98-3.89 (3H, m, H-5, H-6a, H-6b), 1.08-1.00 (21H, m, Si(*i*Pr)$_3$); ^{13}C NMR (101 MHz; $CDCl_3$) δ: 166.0 (Ph*C*(O)O), 165.22 (Ph*C*(O)O), 165.21 (Ph*C*(O)O), 133.4 (4° C), 133.2 (4° C), 133.8 (4° C), 132.7 (4° C), 130.0 (CH), 129.90 (CH), 129.86 (CH), 129.5 (CH), 129.3 (CH), 129.1 (CH), 129.0 (CH), 128.50 (CH), 128.47 (CH), 128.4 (CH), 86.5 (C-1), 80.1 (C-5), 74.8 (C-3), 70.8 (C-2), 69.4 (C-4), 63.1 (C-6), 18.1 (CH(CH$_3$)$_2$), 12.0 (CH(CH$_3$)$_2$). Proton and carbon NMR were consistent with literature data.[21]

Phenyl 2,3,4-tri-*O*-benzoyl-β-D-thioglucopyranoside (3b)

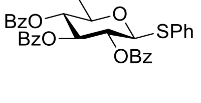

Phenyl 2,3,4-tri-*O*-benzoyl-6-*O*-triisopropylsilyl-β-D-thioglucopyranoside **S24** (3.36 g, 4.54 mmol) was weighed into a flask followed by the addition of THF (15 mL), H_2O (5 mL) and

TFA (7 mL) and stirred for 18 h, after which the solution was concentrated *in vacuo* by azeotroping with PhMe. Following purification by column chromatography (8:2 to 6:4, Petrol ether (40-60):EtOAc) product **3b** was obtained as a white solid (2.34 g, 88%). ^{1}H NMR (400 MHz; $CDCl_3$) δ: 8.00-7.89 (4H, m, Ph), 7.84-7.77 (2H, m, Ph), 7.57-7.47 (4H, m, Ph), 7.45-7.23 (10H, m, Ph), 5.94 (1H, t, *J* = 9.5 Hz, H-3), 5.48 (1H, t, *J* = 9.8 Hz, H-2), 5.47 (1H, t, *J* = 9.8 Hz, H-4), 5.06 (1H, d, *J* = 10.0 Hz, H-1), 3.90-3.81 (2H, m, H-5, H-6a), 3.78-3.71 (1H, m, H-6b); ^{13}C NMR (101 MHz; $CDCl_3$) δ: 166.1 (Ph*C*(O)O), 165.9 (Ph*C*(O)O), 165.2 (Ph*C*(O)O), 133.8 (4° C), 133.5 (4° C), 133.4 (4° C), 133.3 (4° C), 131.9 (CH), 130.1 (CH), 130.0 (CH), 129.9 (CH), 129.3 (CH), 129.2 (CH), 128.9 (CH), 128.7 (CH), 128.64 (CH), 128.60 (CH), 128.55 (CH), 128.4 (CH), 86.4 (C-1), 79.0 (C-5), 74.2 (C-3), 70.7 (C-2), 69.4 (C-4), 61.8 (C-6). ESI-HRMS for $C_{33}H_{32}NaO_8S^+$ $(M+Na)^+$ calculated: 607.1397; found: 607.1394. $[\alpha]_D^{21}$ = +22 (*c* 1.0, $CHCl_3$).

Methyl 2,3-di-*O*-trimethylsilyl-4,6-*O*-benzylidene-α-D-glucopyranoside (S25)

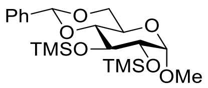

Methyl 4,6-*O*-benzylidene-α-D-glucopyranoside **S6** (3.414 g, 12.09 mmol), which had been pre-dried under vacuum in a flame-dried flask for 1 h, was dissolved in anhydrous CH_2Cl_2 (120 mL) under a N_2 atmosphere. Imidazole (3.30 g, 48.5 mmol) and TMSCl (4.6 mL, 36 mmol) were added and the solution was stirred for 16 h. The solution was washed with H_2O (2 x 100 mL), brine (1 x 50 mL), dried over $MgSO_4$, filtered and concentrated *in vacuo*. Following purification by column chromatography (99:1 to 95:5, Petrol ether (40-60):EtOAc) product **S25** was obtained as a white solid (3.68 g, 71%). ^{1}H NMR (400 MHz; $CDCl_3$) δ: 7.52-7.45 (2H, m, Ph), 7.39-7.31 (3H, m, Ph), 5.50 (1H, s, $PhCHO_2$), 4.62 (1H, d, *J* = 3.7 Hz, H-1), 4.27 (1H, dd, *J* = 9.9, 4.6 Hz, H-6a), 3.96 (1H, t, *J* = 8.9 Hz, H-3), 3.81 (1H, m, H-5), 3.71 (1H, app t, *J* = 10.1 Hz, H-6b), 3.62 (1H, dd, *J* = 8.8, 3.8 Hz, H-2), 3.42 (3H, s, OCH_3), 3.41 (1H, t, *J* = 9.2 Hz, H-4), 0.17 (9H, s, $Si(CH_3)_3$), 0.11 (9H, s, $Si(CH_3)_3$); ^{13}C NMR (101 MHz; $CDCl_3$) δ: 137.6 (4° C), 129.0 (CH), 128.3 (CH), 126.3 (CH), 101.8 ($PhCHO_2$), 101.2 (C-1), 82.3 (C-4), 74.3 (C-2), 71.9 (C-3), 69.3 (C-6), 62.5 (C-5), 55.5 (OCH_3), 0.8 (Si(CH_3)$_3$), 0.5 (Si(CH_3)$_3$).

Methyl 3-*O*-benzyl-4,6-*O*-benzylidene-α-D-glucopyranoside (3c)

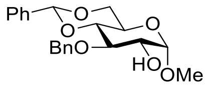

Following the reported synthesis,[22] methyl 2,3-di-*O*-trimethylsilyl-4,6-*O*-benzylidene-α-D-glucopyranoside **S25** (1.50 g, 3.52 mmol), anhydrous CH_2Cl_2 (30 mL), benzaldehyde (429 μL, 4.22 mmol) and Et_3SiH (618 μL, 3.87 mmol) were added to a flask containing activated molecular sieves, the solution was stirred at room temperature for 30 min under N_2. The mixture was then cooled to -78 °C and TMSOTf (65 μL, 0.36 mmol) was added dropwise, when the starting material was totally consumed a 1 M solution of TBAF in THF (7 mL, 7.0 mmol) was added and the solution was gradually warmed to room temperature, after 1.5 h the solution was filtered through Celite® and concentrated *in vacuo*. Following purification by column chromatography (9:1 to 6:4, Petrol ether (40-60):EtOAc) product **3c** was obtained as a solid (960 mg, 73%). ^{1}H NMR (400 MHz; $CDCl_3$) δ: 7.52-7.46 (2H, m, Ph), 7.42-7.27 (8H, m, Ph), 5.57 (1H, s, $PhCHO_2$), 4.96 (1H, d, *J* = 11.6, PhC*H*H), 4.82 (1H, d, *J* = 3.9 Hz, H-1), 4.79 (1H, d, *J* = 11.6 Hz, PhCH*H*), 4.30 (1H, dd, *J* = 9.8, 4.3 Hz, H-6a), 3.88-3.80 (2H, m, H-3, H-5), 3.79-3.70 (2H, m, H-2, H-6b), 3.65 (1H, t, *J* = 9.2 Hz, H-4), 3.45 (3H, s, OCH_3), 2.29 (1H, d, *J* = 7.4 Hz, OH); ^{13}C NMR (101 MHz; $CDCl_3$) δ: 138.6 (4° C), 137.5 (4° C), 129.1 (CH), 128.5 (CH), 128.4 (CH), 128.1 (CH), 127.9 (CH), 126.2 (CH), 101.5 ($PhCHO_2$), 100.1 (C-1), 82.1 (C-4), 79.0 (C-3), 75.0 ($PhCH_2$), 72.6 (C-2), 69.2 (C-6), 62.8 (C-5), 55.6 (OCH_3). Proton and carbon NMR were consistent with literature data.[23]

1,3,4,6-Tetra-*O*-acetyl-2-deoxy-2-(2,2,2-trichloroethoxycarbonylamino)-α/β-D-glucopyranoside (S26)

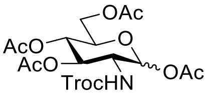

Glucosamine hydrochloride (7.06 g, 32.7 mmol) was dissolved in $NaHCO_3$ (sat. aq.) (300 mL) followed by the addition of TrocCl (8.9 mL, 65 mmol) and stirred for 18 h at room temperature. The precipitate was then filtered off and washed with H_2O and dried under vacuum for 4 h. The solid was then dissolved in a mixture of pyridine (26 mL) and Ac_2O (13 mL) and stirred for 18 h. The solution was then diluted with CH_2Cl_2 (150 mL), washed with 1M HCl (2 x 100 mL), $NaHCO_3$ (sat aq.) (2 x 100 mL), brine (100 mL), dried over $MgSO_4$

filtered and concentrated *in vacuo*, to afford glucosamine **S26** as a white solid (14.57 g, 85%, α:β = 10:1). **α-anomer**: ^{1}H NMR (400 MHz; $CDCl_3$) δ: 6.23 (1H, d, *J* = 3.4 Hz, H-1), 5.28 (1H, dd, *J* = 10.7, 9.6 Hz, H-3), 5.20 (1H, t, *J* = 9.8 Hz, H-4), 5.14 (1H, d, *J* = 9.3 Hz, N*H*Troc), 4.82 (1H, d, *J* = 12.1 Hz, C*H*$HCCl_3$), 4.62 (1H, d, *J* = 12.1 Hz, C*H*$HCCl_3$), 4.28 (1H, dd, *J* = 12.4, 4.0 H, H-6a), 4.20 (1H, ddd, *J* = 10.7, 9.4, 3.8 Hz, H-2), 4.06 (1H, dd, *J* = 12.5, 2.4 Hz, H-6b), 4.06-4.01 (1H, m, H-5), 2.20 (3H, s, CH_3), 2.09 (3H, s, CH_3), 2.04 (6H, 2 x s, 2 x CH_3); ^{13}C NMR (100 MHz; $CDCl_3$) δ: 171.4 (C=O), 170.8 (C=O), 169.3 (C=O), 168.7 (C=O), 154.2 (Troc, C=O), 95.4 (CH_2*C*Cl_3), 90.6 (C-1), 74.8 (*C*H_2CCl_3), 70.5, 69.9 (C-5), 67.6, 61.6 (C-6), 53.4 (C-2), 21.1 (CH_3), 20.82 (CH_3), 20.79 (CH_3), 20.7 (CH_3). **β-anomer**: ^{1}H NMR (400 MHz; $CDCl_3$) δ: 5.74 (1H, d, *J* = 8.8 Hz, H-1). Proton and carbon NMR were consistent with literature data.[24]

Phenyl 3,4,6-tri-*O*-acetyl-2-deoxy-1-thio-2-(2,2,2-trichloroethoxycarbonylamino)-β-D-glucopyranoside (S27)

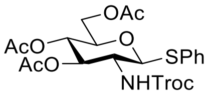

Troc-protected glucosamine **S26** (14.50 g, 27.74 mmol), which had been pre-dried under vacuum for 1 h, was dissolved in anhydrous CH_2Cl_2 (140 mL). Thiophenol (3.4 mL, 33 mmol) and $BF_3\cdot OEt$ (17 mL) were added and the reaction was stirred at room temperature for 18 h. The reaction mixture was diluted with CH_2Cl_2 (100 mL), washed with $NaHCO_3$ $_{(sat.\ aq.)}$ (2 x 100 mL), dried over $MgSO_4$, filtered and concentrated *in vacuo*. Purification by column chromatography (8:2 to 7:3, Hexane:EtOAc) to afford thioglycoside **S27** as an off-white solid (10.80 g, 68%). ^{1}H NMR (400 MHz; $CDCl_3$) δ: 7.55-7.48 (2H, m, Ph), 7.35-7.28 (3H, m, Ph), 5.32 (1H, d, *J* = 9.2 Hz, N*H*Troc), 5.28 (1H, t, *J* = 9.8, H-3), 5.02 (1H, t, *J* = 9.7 Hz, H-4), 4.86 (1H, d, *J* = 10.3, H-1), 4.79 (1H, d, *J* = 12.0 Hz, C*H*$HCCl_3$), 4.72 (1H, d, *J* = 12.0 Hz, C*H*$HCCl_3$), 4.23 (1H, dd, *J* = 12.4, 5.3 Hz, H-6a), 4.16 (1H, dd, *J* = 12.4, 2.5 Hz, H-6b), 3.77-3.65 (2H, m, H-2, H-5), 2.07 (3H, s, CH_3), 2.00 (6H, 2 x s, 2 x CH_3); ^{13}C NMR (100 MHz; $CDCl_3$) δ: 170.8 (C=O), 170.7 (C=O), 169.6 (C=O), 154.0 (Troc, C=O), 133.1 (CH), 132.1 (4° C), 129.1 (CH), 128.5 (CH), 95.5 (CH_2*C*Cl_3), 86.7 (C-1), 75.9 (C-5), 74.7 (*C*H_2CCl_3), 73.3 (C-3), 68.7 (C-4), 62.5 (C-6), 55.2 (C-2), 20.9 (CH_3), 20.8 (CH_3), 20.7 (CH_3). The proton NMR was consistent with literature data,[25] the carbon NMR was

consistent with the literature apart from the reported signal at 119.2 which was observed at 95.5.[25c]

Phenyl 4,6-*O*-benzylidene-2-deoxy-1-thio-2-(2,2,2-trichloroethoxycarbonyl-amino)-β-D-glucopyranoside (3d)

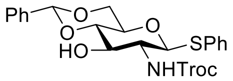

Troc-protected thioglycoside **S27** (10.80 g, 18.86 mmol), was dissolved in a mixture of MeOH/H_2O/Et_3N (8/1/1, 30 mL) and stirred at room temperature for 18 h before being concentrated in vacuo. The resulting deacetylated Troc-protected thioglucosamine and $Cu(OTf)_2$ (360 mg, 0.995 mmol), which had been pre-dried under vacuum for 1 h, were dissolved in anhydrous MeCN (100 mL). Then distilled $PhCH(OMe)_2$ (3.4 mL, 23 mmol) was added and the mixture was sonicated under a balloon of N_2 at room temperature for 2 h. The reaction was quenched by the addition of Et_3N, and concentrated *in vacuo*. Purification by column chromatography (3:1 to 1:1, Hexane/EtOAc) afforded thioglycoside **3d** as a white solid (8.069 g, 80%). ^{1}H NMR (400 MHz; $CDCl_3$) δ: 7.53-7.45 (4H, m, Ph), 7.40-7.30 (6H, m, Ph), 5.54 (1H, s, PhC*H*O_2), 5.29 (1H, br d, *J* = 7.5 Hz, N*H*Troc), 4.91 (1H, d, *J* = 10.2 Hz, H-1), 4.82 (1H, d, *J* = 12.0 Hz, C*H*HCCl_3), 4.71 (1H, d, *J* = 12.0 Hz, CH*H*CCl_3), 4.37 (1H, dd, *J* = 10.6, 4.6 Hz, H-6a), 4.08-3.96 (1H, m, H-3), 3.79 (1H, app t, *J* = 10.3 Hz, H-6b), 3.57-3.40 (3H, m, H-2, H-4, H-5), 2.90 (1H, br s, OH); ^{13}C NMR (101 MHz, $CDCl_3$) δ: 154.3 (C=O, *Troc*), 137.0, 133.0, 129.3, 129.1, 128.6, 128.5, 126.4, 102.1 (Ph<u>C</u>H), 100.0 (CH_2*C*Cl_3), 86.9 (C-1), 81.4, 75.0 (O*C*H_2CCl_3), 72.5 (C-3), 70.5, 68.7 (C-6), 57.6. Proton and carbon NMR were consistent with literature data.[26]

***N*-(*tert*-Butoxycarbonyl)-L-serine methyl ester (3e)**

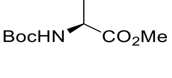

L-Serine methyl ester hydrochloride (2.075 g, 13.34 mmol) and $NaHCO_3$ (3.01 g, 35.8 mmol) were dissolved in MeOH:H_2O (1:1, 24 mL), Boc_2O (3.80 g, 17.4 mmol) was added and the solution was stirred at room temperature for 20 h. The mixture was concentrated and acidified to pH ~4.5 with 1 M aqueous citric acid. The mixture was then extracted with

EtOAc (3 x 30 mL) and the combined organics were dried over $MgSO_4$, filtered and concentrated *in vacuo* to afford Boc-protected serine **3e** as a colourless oil (2.690 g, 92%). ^{1}H NMR (400 MHz; $CDCl_3$) δ: 5.49 (1H, br s, NH), 4.38 (1H, br s, CH), 3.95 (1H, dd, J = 11.2, 4.0 Hz, C*H*H), 3.91 (1H, dd, J = 11.2, 3.6 Hz, CH*H*), 3.77 (3H, s, OCH_3), 2.69 (1H, br s, OH), 1.44 (9H, s, $C(CH_3)_3$); ^{13}C NMR (101 MHz, $CDCl_3$) δ: 171.5 (CH(*C*=O)OCH_3), 155.9 (O(C=O)N), 80.5 (*C*$(CH_3)_3$), 63.7 (CH_2), 55.8 (CH), 52.8 (OCH_3), 28.4 (C(*C*$H_3)_3$). Proton and carbon NMR were consistent with literature data.[27]

N-[(9-Fluorenylmethoxy)carbonyl]-L-serine methyl ester (3f)

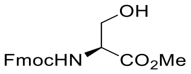

L-Serine methyl ester hydrochloride (2.068 g, 13.29 mmol) and $NaHCO_3$ (2.749 g, 32.72 mmol) were dissolved in H_2O (30 mL) and cooled to 0 °C. A solution of 9-fluorenylmethyl *N*-hydroxysuccinimide ester (4.404 g, 13.06 mmol) in 1,4-dioxane (30 mL) was added dropwise over 30 min, after which the ice bath was removed and the mixture was stirred for a further 1 h. H_2O (15 mL) was added and the solution was extracted with EtOAc (3 x 35 mL), the combined organics were washed with 0.2 M HCl (15 mL), brine (15 mL), dried over Na_2SO_4, filtered and concentrated *in vacuo*. The residue was purified by column chromatography (1:0 to 95:5, CH_2Cl_2:MeOH) to afford Fmoc-protected serine **3f** as a white solid (4.222 g, 93%). ^{1}H NMR (400 MHz; $CDCl_3$) δ: 7.76 (2H, d, J = 7.5 Hz, Ar), 7.64-7.55 (2H, m, Ar), 7.40 (2H, t, J = 7.8 Hz, Ar), 7.31 (2H, tt, J = 7.5, 1.1 Hz, Ar), 5.77 (1H, d, J = 6.7 Hz, NH), 4.53-4.35 (3H, m, Fmoc CH_2, C*H*CH_2OH), 4.22 (1H, t, J = 6.8 Hz, Fmoc CH), 4.00 (1H, dd, J = 11.0, 3.0 Hz, C*H*HOH), 3.91 (1H, dd, J = 11.0, 2.3 Hz, CH*H*OH), 3.78 (3H, s, OCH_3), 2.24 (1H, br s, OH); ^{13}C NMR (101 MHz, $CDCl_3$) δ: 171.1 ((*C*=O)OCH_3), 156.4 (O(C=O)N), 143.9 (4° C), 143.8 (4° C), 141.5 (4° C), 141.4 (4° C), 127.9 (CH), 127.22 (CH), 127.19 (CH), 125.2 (CH), 120.14 (CH), 120.12 (CH), 67.3 (Fmoc CH_2), 63.4 (CH_2OH), 56.1 (*C*HCH_2OH), 52.9 (OCH_3), 47.3 (Fmoc CH). Proton and carbon NMR were consistent with literature data.[28]

L-Threonine methyl ester hydrochloride (S28)

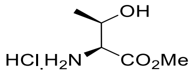

L-Threonine methyl ester (5.00 g, 29.5 mmol) was dissolved in 3 M HCl in methanol (40 mL) and stirred at room temperature under N_2 for 18 h. The reaction was found to be incomplete (TLC iPr:H_2O:$NH_4OH_{(conc.)}$, 7:3:1) and the mixture was heated at reflux for 24 h. The solution was concentrated *in vacuo* to afford threonine **S28** as a white solid (7.12 g, 100%). ^{1}H NMR (400 MHz; d6-DMSO) δ: 8.53 (3H, br s, NH_3), 5.68 (1H, br s, OH), 4.10 (1H, qd, *J* = 6.5, 3.8 Hz, C*H*CH_3), 3.96-3.86 (1H, m, C*H*CO_2Me), 3.69 (3H, s, OCH_3), 1.16 (3H, d, *J* = 6.5 Hz, CHC*H*$_3$); ^{13}C NMR (101 MHz, d6-DMSO) δ: 168.6 (CH(*C*=O)OCH_3), 65.1 (*C*HCH_3), 57.9 (*C*HCO_2Me), 52.7 (OCH_3), 20.0 (CH*C*H$_3$). Proton and carbon NMR were consistent with literature data.[29]

***N*-(*t*-Butoxycarbonyl)-L-threonine methyl ester (3g)**

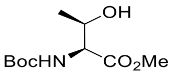

L-Threonine methyl ester hydrochloride **S28** (2.121 g, 12.51 mmol) and $NaHCO_3$ (3.14 g, 37.4 mmol) were dissolved in MeOH:H_2O (1:1, 24 mL), Boc_2O (3.80 g, 17.4 mmol) was added and the solution was stirred at room temperature for 20 h. The mixture was concentrated and acidified to pH ~4.5 with 1 M aqueous citric acid. The mixture was then extracted with EtOAc (3 x 30 mL) and the combined organics were dried over $MgSO_4$, filtered and concentrated *in vacuo* to afford Boc-protected threonine **3g** as a colourless oil (2.690 g, 92%). ^{1}H NMR (400 MHz; $CDCl_3$) δ: 5.35 (1H, d, *J* = 8.7 Hz, NH), 4.35-4.18 (2H, m, C*H*CH_3, C*H*CO_2Me), 3.76 (3H, s, OCH_3), 2.34 (1H, br s, OH), 1.44 (9H, s, $C(CH_3)_3$), 1.24 (3H, d, *J* = 6.3 Hz, CHC*H*$_3$); ^{13}C NMR (101 MHz, $CDCl_3$) δ: 172.0 (CH(*C*=O)OCH_3), 156.1 (O(C=O)N), 80.1 (*C*$(CH_3)_3$), 68.1 (CH), 58.7 (CH), 52.5 (OCH_3), 28.3 (C(*C*H$_3$)$_3$), 19.9 (CH*C*H$_3$). Proton and carbon NMR were consistent with literature data.[30]

Glycosylation Reactions

General Procedure

The monosaccharide acceptor (~0.100 mmol) and glucal (0.120-0.150 mmol) were weighed into a round bottom flask and then put under vacuum for 1 h. Then the flask was filled with N_2, followed by the addition of a stock solution (1 mL, ~0.001 mmol, 1 mol%) of $TsOH{\cdot}H_2O$ in anhydrous CH_2Cl_2 (2 mg (0.01 mmol) in 10 mL). The solutions were then stirred at room temperature for the times stated in tables 11, 12 and 13 (section 2.3). The solutions were then quenched by the addition of Et_3N (except for reactions involving Fmoc protected serine **3f**) concentrated *in vacuo* and purified by column chromatography. All reactions were conducted in duplicate.

Glucal Scope with Acceptor 3a

Methyl 2,3,4-tri-*O*-benzyl-6-*O*-(3,4,6-tri-*O*-benzyl-2-deoxy-α/β-D-*erythro*-hexapyranosyl)-α-D-glucopyranoside (6a) and Methyl 2,3,4-tri-*O*-benzyl-6-*O*-(4,6-di-*O*-benzyl-2,3-dideoxy-α/β-D-*erythro*-hex-2-enopyranosyl)-α-D-glucopyranoside (7a)

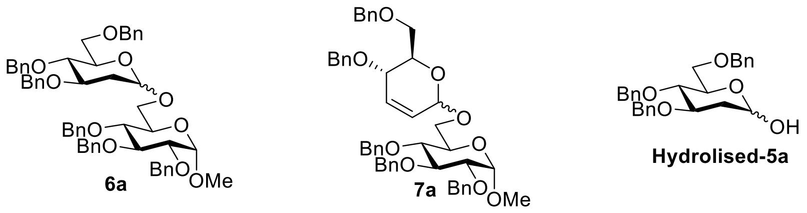

Following the general procedure, glucal **5a** (61 mg, 0.15 mmol), glucoside **3a** (46 mg, 0.099 mmol) and a CH_2Cl_2 stock solution of $TsOH{\cdot}H_2O$ (1 mL, 1 mol%) were used. Purification by column chromatography (Hexane:EtOAc, 9:1 to 8:2) afforded an inseparable mixture of the products **6a** and **7a** and **hydrolysed-5a** (76 mg, **6a** ~74%, α:β = 6:1, **7a** ~11%, **hydrolysed-5a** ~4%).

6a α-anomer: ^{1}H NMR (400 MHz, $CDCl_3$) δ: 3.37 (3H, s, OCH_3), 2.33 (1H, app dd, J = 12.6, 5.0 Hz, H-2a'); ^{13}C NMR (101 MHz, $CDCl_3$) δ: 98.0 (C-1), 97.9 (C-1'), 55.2 (OCH_3), 35.4 (C-2'). **6a β-anomer:** ^{1}H NMR (400 MHz, $CDCl_3$) δ: 3.39 (3H, s, OCH_3), 2.20 (1H, app dd, J = 12.1, 4.9 Hz, H-2a'); ^{13}C NMR (101 MHz, $CDCl_3$) δ: 100.1 (C-1'), 36.6 (C-2').

7a α/β-anomer: ^{1}H NMR (400 MHz, $CDCl_3$) δ: 6.11-6.01 (1.14H, m, α/β-C*H*=CH), 5.85-5.76 (1.14H, m, α/β-CH=C*H*), 5.23 (1H, br s, α-H-1); ^{13}C NMR (101 MHz, $CDCl_3$) δ: 130.8

(α/β-*C*H=CH), 126.5 (α/β-CH=*C*H), 100.1 (α-C-1'). ESI-HRMS for $C_{48}H_{52}NaO_9^+$ (MNa^+) calculated: 795.3504; found: 795.3508.

Hydrolysed-5a ^{1}H NMR (400 MHz, $CDCl_3$) δ: 5.41 (1H, br s, H-1). ESI-HRMS for $C_{27}H_{36}NaO_5^+$ (MNa^+) calculated: 457.1985; found: 457.1976.

Methyl 2,3,4-tri-*O*-benzyl-6-*O*-(3,4,6-tri-*O*-*tert*-butyldimethylsilyl-2-deoxy-α/β-D-*erythro*-hexapyranosyl)-α-D-glucopyranoside (6b)

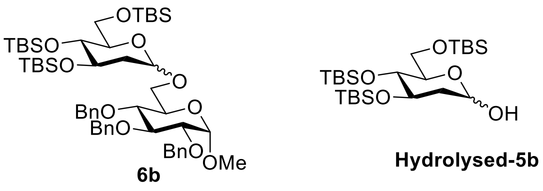

Following the general procedure, glucal **5b** (76 mg, 0.16 mmol), glucoside **3a** (46 mg, 0.099 mmol) and a CH_2Cl_2 stock solution of $TsOH{\cdot}H_2O$ (1 mL, 1 mol%) were used. Purification by column chromatography (Hexane:EtOAc, 95:5 to 92:8) afforded an inseparable mixture of the product **6b** and **hydrolysed-5b** as a syrup (62 mg, **6b** ~60%, α:β = 5:1, **hydrolysed-5b** ~10%). **6b α-anomer:** ^{1}H NMR (400 MHz, $CDCl_3$) δ: 7.41-7.20 (15H, m, Ph), 4.91-4.88 (1H, m, H-1'), 4.98 (1H, d, *J* = 10.7 Hz, OC*H*HPh), 4.89 (1H, d, *J* = 11.3 Hz, OC*H*HPh), 4.83 (1H, d, *J* = 10.7 Hz, OCH*H*Ph), 4.80 (1H, d, *J* = 12.2 Hz, OC*H*HPh), 4.67 (1H, d, *J* = 12.1 Hz, OCH*H*Ph), 4.62 (1H, d, *J* = 11.4 Hz, OCH*H*Ph), 4.59 (1H, d, *J* = 3.6 Hz, H-1), 4.02-3.93 (2H, m, 2 x CH), 3.84 (1H, dd, *J* = 11.3, 4.9 Hz, C*H*H), 3.78 (1H, dd, *J* = 10.9, 2.6 Hz, C*H*H), 3.76-3.70 (1H, m, CH), 3.64 (1H, dd, *J* = 11.0, 6.4 Hz, CH*H*), 3.60 (1H, dd, *J* = 11.5, 1.8 Hz, CH*H*), 3.56-3.45 (1H, m, CH), 3.40-3.33 (1H, m, CH), 3.36 (3H, s, OCH_3), 2.08, (1H, ddd, *J* = 13.2, 4.6, 2.3 Hz, H-2a'), 1.63-1.55 (1H, m, H-2b'), 0.97-0.77 (27H, m, 3 x $SiC(CH_3)_3$), 0.16-0.00 (18H, m, 6 x $SiCH_3$); ^{13}C NMR (101 MHz, $CDCl_3$) δ: 138.9 (4° C), 138.5 (4° C), 138.3 (4° C), 98.0 (C-1), 97.0 (C-1'), 82.4 (CH), 80.1 (CH), 78.2 (CH), 75.9 (O*C*H_2Ph), 75.2 (O*C*H_2Ph), 74.6 (CH), 73.5 (O*C*H_2Ph), 72.8 (CH), 71.3 (CH), 70.4 (CH), 65.2, 62.9 (C-6, C-6'), 55.0 (OCH_3), 38.7 (C-2'), 26.45 (SiC(*C*H_3)$_3$), 26.2 (SiC(*C*H_3)$_3$), 26.1 (SiC(*C*H_3)$_3$), 18.6 (Si*C*(CH_3)$_3$), 18.5 (Si*C*(CH_3)$_3$), 18.2 (Si*C*(CH_3)$_3$), -2.9 ($SiCH_3$), -3.0 ($SiCH_3$), -4.1 ($SiCH_3$), -4.5 ($SiCH_3$), -4.8 ($SiCH_3$), -5.2 ($SiCH_3$). **6b β-anomer:** ^{1}H NMR (400 MHz, $CDCl_3$) δ: 7.41-7.20 (15H, m, Ph), 4.59 (1H, d, *J* = 3.6 Hz, H-1), 4.36 (1H, dd, *J* = 9.5, 2.0 Hz, H-1'), 3.36 (3H, s, OCH_3),), 0.97-0.77 (27H, m, 3 x $SiC(CH_3)_3$), 0.16- -0.04

(18H, m, 6 x $SiCH_3$); ^{13}C NMR (101 MHz, $CDCl_3$) δ: 99.5 (C-1'), 98.1 (C-1), 51.2 (OCH_3), 40.3 (C-2'), 26.47 ($SiC(\mathit{C}H_3)_3$), 26.3 ($SiC(\mathit{C}H_3)_3$), 25.8 ($SiC(\mathit{C}H_3)_3$). ESI-HRMS for $C_{52}H_{84}NaO_{10}Si_3^+$ (MNa^+) calculated: 975.5264; found: 975.5236.

Hydrolysed-5b ^1H NMR (400 MHz, $CDCl_3$) δ: 5.27 (1H, br s, H-1). ESI-HRMS for $C_{24}H54NaO_5Si_3^+$ (MNa^+) calculated: 529.3171; found: 529.3152.

Methyl 2,3,4-tri-*O*-benzyl-6-*O*-(4,6-di-*O*-*tert*-butyldimethylsilyl-2,3-dideoxy-α/β-D-*erythro*-hex-2-enopyranosyl)-α-D-glucopyranoside (7b)

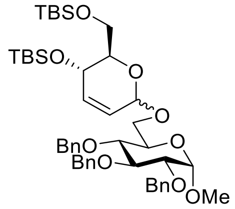

Further elution of the above column afforded the Ferrier rearrangement product **7b** (28 mg, 34%, α:β = 4:1). **α-anomer:** ^1H NMR (400 MHz, $CDCl_3$) δ: 7.38-7.22 (15H, m, Ph), 5.86-5.80 (1H, m, C*H*=CH), 5.68 (1H, ddd, *J* = 10.2, 2.6, 2.0 Hz, CH=C*H*), 5.01 (1H, br s, H-1'), 4.97 (1H, d, *J* = 10.7 Hz, OC*H*HPh), 4.89 (1H, d, *J* = 11.0 Hz, OC*H*HPh), 4.81 (1H, d, *J* = 10.9 Hz, OCH*H*Ph), 4.79 (1H, d, *J* = 12.1 Hz, OC*H*HPh), 4.67 (1H, d, *J* = 12.3 Hz, OCH*H*Ph), 4.66 (1H, d, *J* = 11.4 Hz, OCH*H*Ph), 4.61 (1H, d, *J* = 3.5 Hz, H-1), 4.22 (1H, dq, *J* = 9.0, 1.8 Hz, H-4'), 4.02 (1H, dd, *J* = 11.3, 3.9 Hz, C*H*H), 3.99 (1H, t, *J* = 9.1 Hz, H-3), 3.79-3.72 (1H, m, CH), 3.69 (1H, dd, *J* = 11.4, 2.1 Hz, CH*H*), 3.67-3.58 (3H, m, CH and CH_2), 3.58-3.54 (1H, m, CH), 3.52 (1H, dd, *J* = 9.7, 3.6 Hz, H-2), 3.38 (3H, s, OCH_3), 0.88 (9H, s, $SiC(CH_3)_3$), 0.86 (9H, s, $SiC(CH_3)_3$), 0.08 (3H, s, $SiCH_3$), 0.06 (3H, s, $SiCH_3$), 0.01 (3H, m, $SiCH_3$), 0.00 (3H, m, $SiCH_3$); ^{13}C NMR (101 MHz, $CDCl_3$) δ: 139.0 (4° C), 138.6 (4° C), 138.4 (4° C), 134.5 (C*H*=CH), 125.5 (CH=C*H*), 98.21 (C-1), 94.8 (C-1'), 82.2 (C-3), 80.1 (C-2), 78.0 (C-4), 75.8 (O*C*H_2Ph), 75.0 (O*C*H_2Ph), 73.5 (O*C*H_2Ph), 72.6 (CH), 70.3 (CH), 66.6 (CH_2), 63.7 (C-4'), 62.2 (CH_2), 55.3 (OCH_3), 26.10 ($SiC(\mathit{C}H_3)_3$), 25.88 ($SiC(\mathit{C}H_3)_3$), 18.6 ($Si\mathit{C}(CH_3)_3$), 18.1 ($Si\mathit{C}(CH_3)_3$), -4.1 ($SiCH_3$), -4.7 ($SiCH_3$), -5.0 ($SiCH_3$), -5.2 ($SiCH_3$). **β-anomer:** ^1H NMR (400 MHz, $CDCl_3$) δ: 7.38-7.22 (15H, m, Ph), 5.63 (1H, dt, *J* = 10.2, 1.7 Hz, CH=C*H*), 5.04 (1H, q, *J* = 1.7 Hz, H-1'), 4.58 (1H, d, *J* = 3.6 Hz, H-1), 3.36 (3H, s, OCH_3), 0.89 (9H, s, $SiC(CH_3)_3$), 0.87 (9H, s, $SiC(CH_3)_3$); ^{13}C NMR (101 MHz, $CDCl_3$) δ: 133.1 (C*H*=CH), 127.4 (CH=C*H*), 98.15 (C-1), 96.8 (C-1'), 55.2 (OCH_3), 26.07

(SiC(CH_3)$_3$), 25.94 (SiC(CH_3)$_3$), 18.5 (Si*C*(CH_3)$_3$), 18.2 (Si*C*(CH_3)$_3$). ESI-HRMS for $C_{46}H_{68}NaO_9Si_2^+$ (MNa^+) calculated: 843.4294; found: 843.4270.

Methyl 2,3,4-tri-*O*-benzyl-6-*O*-(4-*O*-benzyl-2-deoxy-3,6-di-*O*-*tert*-butyldimethylsilyl-α/β-D-*erythro*-hexapyranosyl)-α-D-glucopyranoside (6c)

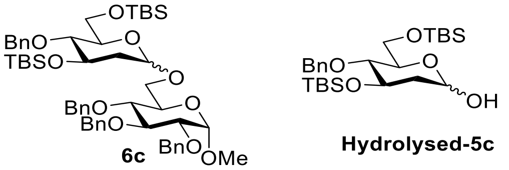

Following the general procedure, glucal **5c** (71 mg, 0.15 mmol), glucoside **3a** (46 mg, 0.099 mmol) and a CH_2Cl_2 stock solution of TsOH·H_2O (1 mL, 1 mol%) were used. Purification by column chromatography (Hexane:EtOAc, 95:5 to 8:2) afforded an inseparable mixture of the product **6c** and **hydrolysed-5c** as a syrup (60 mg, **6c** ~60%, α:β = 15:1, **hydrolysed-5c** ~8%). **6c α-anomer:** ^{1}H NMR (500 MHz, $CDCl_3$) δ: 7.38-7.23 (20H, m, Ph), 4.98 (1H, d, *J* = 11.0 Hz, OC*H*HPh), 4.91 (1H, d, *J* = 11.0 Hz, OC*H*HPh), 4.90 (1H, br. d, *J* = 3.6 Hz, H-1'), 4.88 (1H, d, *J* = 11.3 Hz, OCH*H*Ph), 4.82 (1H, d, *J* = 10.9 Hz, OCH*H*Ph), 4.79 (1H, d, *J* = 12.1 Hz, OC*H*HPh), 4.67 (1H, d, *J* = 12.2 Hz, OCH*H*Ph), 4.60 (2H, d, *J* = 10.9 Hz, 2 x OCH*H*Ph), 4.59 (1H, d, *J* = 3.7 Hz, H-1), 4.13 (1H, ddd, *J* = 11.2, 8.7, 5.0 Hz, H-3'), 3.99 (1H, t, *J* = 9.2 Hz, H-3), 3.77-3.71 (2H, m, H-5 or H-5', and H-6a or H-6a'), 3.70-3.67 (2H, m, H-6 or H-6'), 3.60-3.53 (2H, m, H-5 or H-5', and H-6b or H-6b'), 3.51 (1H, dd, *J* = 9.6, 3.6 Hz, H-2), 3.44 (1H, t, *J* = 9.2, H-4), 3.36 (3H, s, OCH_3), 3.31 (1H, dd, J = 9.7, 8.7 Hz, H-4'), 2.06 (1H, ddd, *J* = 12.9, 5.1, 1.5 Hz, H-2a'), 1.65 (1H, ddd, J = 13.0, 11.3, 3.5 Hz, H-2b'), 0.90 (9H, s, Si(CH_3)$_3$), 0.88 (9H, s, Si(CH_3)$_3$), 0.063 (3H, s, $SiCH_3$), 0.061 (3H, s, $SiCH_3$), 0.02 (3H, s, $SiCH_3$), 0.01 (3H, s, $SiCH_3$); ^{13}C NMR (126 MHz, $CDCl_3$) δ: 139.1 (4° C), 138.8 (4° C), 138.4 (4° C), 138.3 (4° C), 128.60 (CH), 128.55 (CH), 128.5 (CH), 128.4 (CH), 128.22 (CH), 128.19 (CH), 128.1 (CH), 127.77 (CH), 127.75 (CH), 127.7 (CH), 127.5 (CH), 97.9 (C-1), 97.6 (C-1'), 82.4 (C-3), 80.2 (C-2), 79.8 (C-4'), 78.3 (C-4), 76.0 (O*C*H_2Ph), 75.2 (O*C*H_2Ph), 75.0 (O*C*H_2Ph), 73.5 (O*C*H_2Ph), 72.6 (C-5 or C-5'), 70.7 (C-3'), 70.2 (C-5 or C-5'), 65.5, 62.7 (C-6, C-6'), 55.0 (OCH_3), 39.3 (C-2'), 26.1 (SiC(CH_3)$_3$), 26.04 (SiC(CH_3)$_3$), 18.5 (Si*C*(CH_3)$_3$), 18.2 (Si*C*(CH_3)$_3$), -4.3 ($SiCH_3$), -4.4 ($SiCH_3$), -4.9 ($SiCH_3$), -5.2 ($SiCH_3$). **6c β-anomer:** ^{1}H NMR (400 MHz, $CDCl_3$) δ: 3.37 (3H, s, OCH_3). ESI-HRMS for $C_{53}H_{76}NaO_{10}Si_2^+$ (MNa^+) calculated: 951.4869; found: 951.4823.

Hydrolysed-5c ^{1}H NMR (400 MHz, CDCl$_3$) δ: 5.33 (1H, d, *J* = 3.5 Hz, H-1). ESI for $C_{25}H_{46}NaO_5Si_2^+$ (MNa$^+$) calculated: 503.3; found: 505.3.

Methyl 2,3,4-tri-*O*-benzyl-6-*O*-(4-*O*-benzyl-6-*O*-*tert*-butyldimethylsilyl-2,3-dideoxy-α/β-D-*erythro*-hex-2-enopyranosyl)-α-D-glucopyranoside (7c)

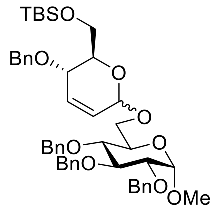

Further elution of the above column afforded the Ferrier rearrangement product **7c** as a syrup (16 mg, 20%, α:β = 4:1). **α-anomer:** ^{1}H NMR (500 MHz, CDCl$_3$) δ: 7.41-7.21 (20H, m, Ph), 6.04 (1H, dt, *J* = 10.3, 1.2 Hz, C*H*=CH), 5.77 (1H, ddd, *J* = 10.3, 2.7, 2.0 Hz, CH=C*H*), 5.05 (1H, dt, *J* = 2.4, 1.2 Hz, H-1'), 4.97 (1H, d, *J* = 10.9 Hz, OC*H*HPh), 4.88 (1H, d, *J* = 11.0 Hz, OC*H*HPh), 4.80 (1H, d, *J* = 10.9 Hz, OCH*H*Ph), 4.79 (1H, d, *J* = 12.1 Hz, OC*H*HPh), 4.66 (1H, d, *J* = 12.2 Hz, OCH*H*Ph), 4.64 (1H, d, *J* = 11.0 Hz, OCH*H*Ph), 4.63 (1H, d, *J* = 11.7 Hz, OC*H*HPh), 4.62 (1H, d, *J* = 3.6 Hz, H-1), 4.53 (1H, d, *J* = 11.6 Hz, OCH*H*Ph), 4.12 (1H, dq, *J* = 8.7, 1.8 Hz, H-4'), 4.04 (1H, dd, *J* = 11.3, 3.7 Hz, CH*H*), 3.98 (1H, dd, *J* = 9.6, 8.9 Hz, H-3), 3.78-3.64 (5H, m, H-5, H-5' and 3 x C*H*H), 3.60 (1H, dd, *J* = 10.0, 8.9 Hz, H-4), 3.52 (1H, dd, *J* = 9.6, 3.6 Hz, H-2), 3.37 (3H, s, OCH$_3$), 0.86 (9H, s, SiC(C*H*$_3$)$_3$), 0.02 (6H, s, 2 x SiCH$_3$); ^{13}C NMR (126 MHz, CDCl$_3$) δ: 139.0 (4° C), 138.6 (4° C), 138.44 (4° C), 138.38 (4° C), 130.9, 126.7 (C-2', C-3'), 98.2 (C-1), 95.0 (C-1'), 82.2 (C-3), 80.1 (C-2), 78.0 (C-4), 75.8 (O*C*H$_2$Ph), 75.0 (O*C*H$_2$Ph), 73.5 (O*C*H$_2$Ph), 71.2 (O*C*H$_2$Ph), 70.8 (CH), 70.23 (CH), 70.15 (C-4'), 66.81, 62.5 (C-6 and C-6'), 55.3 (OCH$_3$), 26.14 (SiC(CH$_3$)$_3$), 18.6 (Si*C*(CH$_3$)$_3$), 5.05 (SiCH$_3$), 5.18 (SiCH$_3$). **β-anomer:** ^{1}H NMR (500 MHz, CDCl$_3$) δ: 5.99 (1H, ddd, *J* = 10.3, 3.5, 1.5 Hz, C*H*=CH), 3.36 (3H, s, OCH$_3$), 0.86 (9H, s, SiC(C*H*$_3$)$_3$), 0.01 (3H, s, SiCH$_3$), 0.00 (3H, s, SiCH$_3$); ^{13}C NMR (126 MHz, CDCl$_3$) δ: 98.2 (C-1), 95.6 (C-1'), 82.4 (CH), 80.0 (CH), 77.1 (CH), 75.8 (O*C*H$_2$Ph), 71.1 (O*C*H$_2$Ph), 69.9 (CH), 69.1 (CH), 66.2, 63.2 (C-6 and C-6'), 55.2 (OCH$_3$). 26.05 (SiC(*C*H$_3$)$_3$), 18.4 (Si*C*(CH$_3$)$_3$). ESI-HRMS for $C_{47}H_{60}NaO_9Si^+$ (MNa$^+$) calculated: 819.3899; found: 819.3891.

Methyl 2,3,4-tri-*O*-benzyl-6-*O*-(4-*O*-acetyl-2-deoxy-3,6-di-*O*-*tert*-butyldimethylsilyl-α/β-D-*erythro*-hexapyranosyl)-α-D-glucopyranoside (6d)

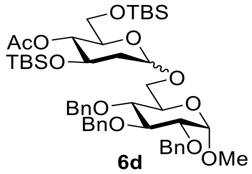

6d

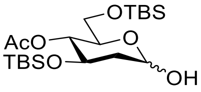

Hydrolysed-5d

Following the general procedure, glucal **5d** (66 mg, 0.16 mmol), glucoside **3a** (47 mg, 010 mmol) and a CH_2Cl_2 stock solution of TsOH·H_2O (1 mL, 1 mol%) were used. Purification by column chromatography (Hexane:EtOAc, 95:5 to 8:2) afforded an inseparable mixture of the product **6d** and **hydrolysed-5d** as a syrup (63 mg, **6d** ~64%, α:β = 15:1, **hydrolysed-5d** ~15%). **6d α-anomer:** ^{1}H NMR (500 MHz, $CDCl_3$) δ: 7.39-7.26 (15H, m, Ph), 4.98 (1H, d, *J* = 10.7 Hz, OC*H*HPh), 4.95 (1H, br. d, *J* = 3.8 Hz, H-1'), 4.93 (1H, d, *J* = 11.3 Hz, OC*H*HPh), 4.82 (1H, d, *J* = 10.7 Hz, OCH*H*Ph), 4.80 (1H, d, *J* = 12.2 Hz, OC*H*HPh), 4.72 (1H, dd, *J* = 10.0, 8.9 Hz, H-4'), 4.68 (1H, d, *J* = 12.1 Hz, OCH*H*Ph), 4.62 (1H, d, *J* = 11.4 Hz, OCH*H*Ph), 4.61 (1H, d, *J* = 3.5 Hz, H-1), 4.06 (1H, ddd, *J* = 11.1, 9.0, 5.1 Hz, H-3'), 4.00 (1H, t, *J* = 9.3 Hz, H-3), 3.81 (1H, dd, *J* = 11.2, 5.4 Hz, H-6a or H-6a'), 3.78-3.74 (1H, m, H-5), 3.69-3.64 (1H, m, H-5'), 3.64-3.60 (1H, m, H-6b or H-6b'), 3.55 (2H, app d, *J* = 4.5 Hz, H-6 or H-6'), 3.52 (1H, dd, *J* = 9.6, 3.6 Hz, H-2), 3.45 (1H, dd, *J* = 9.9, 8.9 Hz, H-4), 3.38 (3H, s, OCH_3), 2.06 (1H, ddd, *J* = 13.0, 5.2, 1.1 Hz, H-2a'), 2.02 (3H, s, (C=O)CH_3), 1.71 (1H, ddd, J = 13.1, 11.2, 3.5 Hz, H-2b'), 0.86 (9H, s, $SiC(CH_3)_3$), 0.84 (9H, s, $SiC(CH_3)_3$), 0.02 (3H, s, $SiCH_3$), 0.01 (6H, s, 2 x $SiCH_3$), 0.00 (3H, s, $SiCH_3$); ^{13}C NMR (126 MHz, $CDCl_3$) δ: 169.8 (C=O), 138.8 (4° C), 138.4 (4° C), 138.3 (4° C), 128.61 (CH), 128.58 (CH), 128.55 (CH), 128.3 (CH), 128.2 (CH), 128.1 (CH), 127.84 (CH), 127.82 (CH), 127.7 (CH), 98.0 (C-1), 97.3 (C-1'), 82.4 (C-3), 80.2 (C-2), 78.4 (C-4), 76.1 (O*C*H_2Ph), 75.2 (O*C*H_2Ph), 73.5 (O*C*H_2Ph), 73.2 (C-4'), 71.5 (C-5'), 70.3 (C-5), 68.1 (C-3'), 65.5, 63.3 (C-6, C-6'), 55.0 (OCH_3), 38.9 (C-2'), 26.0 (SiC(*C*H_3)$_3$), 25.7 (SiC(*C*H_3)$_3$), 21.3 ((C=O)*C*H_3), 18.5 (Si*C*(CH_3)$_3$), 18.0 (Si*C*(CH_3)$_3$), -4.4 ($SiCH_3$), -4.7 ($SiCH_3$), -5.2 ($SiCH_3$), -5.3 ($SiCH_3$). **6d β-anomer:** ^{1}H NMR (400 MHz, $CDCl_3$) δ: 4.25 (1H, dd, *J* = 9.7, 1.8 Hz, H-1'). ESI-HRMS for $C_{48}H_{72}NaO_{11}Si_2^+$ (MNa^+) calculated: 903.4505; found: 903.4532.

Hydrolysed-5d ^{1}H NMR (400 MHz, $CDCl_3$) δ: 5.36 (1H, d, *J* = 3.4 Hz, H-1). ESI-HRMS for $C_{20}H_{42}NaO_5Si_2{}^+$ (MNa^+) calculated: 457.2412; found: 457.2404.

Methyl 2,3,4-tri-*O*-benzyl-6-*O*-(4-*O*-acetyl-6-*O*-*tert*-butyldimethylsilyl-2,3-dideoxy-α/β-D-*erythro*-hex-2-enopyranosyl)-α-D-glucopyranoside (7d)

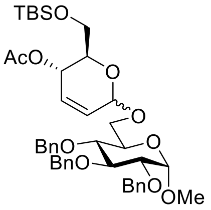

Further elution of the above column afforded the Ferrier rearrangement product **7d** as a syrup (1 mg, 2%, α:β = 3:1). **α-anomer:** ^{1}H NMR (500 MHz, $CDCl_3$) δ: 7.39-7.23 (15H, m, Ph), 5.90-5.85 (1H, m, C*H*=CH), 5.81 (1H, ddd, *J* = 10.2, 2.6, 1.8 Hz, CH=C*H*), 5.31 (1H, dq, *J* = 9.5, 1.6 Hz, H-4'), 5.09 (1H, br s, H-1'), 4.98 (1H, d, *J* = 10.9 Hz, OC*H*HPh), 4.90 (1H, d, *J* = 10.9 Hz, OC*H*HPh), 4.80 (1H, d, *J* = 10.9 Hz, OCH*H*Ph), 4.79 (1H, d, *J* = 12.1 Hz, OC*H*HPh), 4.67 (1H, d, *J* = 12.2 Hz, OCH*H*Ph), 4.64 (1H, d, *J* = 11.0 Hz, OCH*H*Ph), 4.61 (1H, d, *J* = 3.6 Hz, H-1), 4.04 (1H, dd, *J* = 11.1, 3.6 Hz, CH*H*), 3.99 (1H, dd, *J* = 9.6, 8.9 Hz, H-3), 3.79-3.69 (3H, m, H-5, H-5' and C*H*H), 3.64-3.58 (3H, m, H-4 and CH_2), 3.52 (1H, dd, *J* = 9.7, 3.5 Hz, H-2), 3.37 (3H, s, OCH_3), 2.05 ((C=O)CH_3) 0.856 (9H, s, SiC(CH_3)$_3$), 0.000 (3H, s, $SiCH_3$), -0.003 (3H, s, $SiCH_3$); ^{13}C NMR (126 MHz, $CDCl_3$) δ: 170.40 (C=O), 138.93 (4° C), 138.54 (4° C), 138.32 (4° C), 129.4 (C-2'), 98.20 (C-1), 94.8 (C-1'), 82.2 (C-3), 80.1 (C-2), 78.0 (C-4), 75.88 (O*C*H_2Ph), 75.06 (O*C*H_2Ph), 73.48 (O*C*H_2Ph), 73.48 (CH), 70.2 (CH), 69.6 (CH_2), 65.5 (C-4'), 62.5 (CH_2), 55.3 (OCH_3), 26.1 (SiC(*C*H_3)$_3$), 21.22 ((C=O)*C*H_3), 18.6 (Si*C*(CH_3)$_3$), -5.27 ($SiCH_3$), -5.31 ($SiCH_3$). **β-anomer:** ^{1}H NMR (500 MHz, $CDCl_3$) δ: 7.39-7.23 (15H, m, Ph), 5.95 (1H, ddd, *J* = 10.3, 3.9, 1.6 Hz, C*H*=CH), 5.20 (1H, ddt, *J* = 5.0, 3.7, 1.2 Hz, H-4'), 3.58 (1H, s, *J* = 3.7 Hz, H-1), 3.36 (3H, s, OCH_3), 2.06 ((C=O)CH_3), 0.860 (9H, s, SiC(CH_3)$_3$), 0.01 (6H, s, 2 x $SiCH_3$); ^{13}C NMR (126 MHz, $CDCl_3$) δ: 170.38 (C=O), 138.91 (4° C), 138.49 (4° C), 138.30 (4° C), 130.1, 126.5 (C-2', C-3'), 98.19 (C-1), 95.1 (C-1'), 82.4 (CH), 80.0 (CH), 77.7 (CH), 76.1 (CH), 75.89 (O*C*H_2Ph), 75.05 (O*C*H_2Ph), 73.51 (O*C*H_2Ph), 69.9 (CH), 66.3 (CH_2), 64.8 (C-4'), 63.2 (CH_2), 55.2 (OCH_3), 26.0 (SiC(*C*H_3)$_3$), 21.24 ((C=O)CH_3), 18.4 (Si*C*(CH_3)$_3$), -5.21 ($SiCH_3$), -5.25 ($SiCH_3$). ESI-HRMS for $C_{42}H_{56}NaO_{10}Si^+$ (MNa^+) calculated: 771.3535; found: 771.3558.

Methyl 2,3,4-tri-*O*-benzyl-6-*O*-(4-*O*-allyl-2-deoxy-3,6-di-*O*-triisopropylsilyl-α/β-D-*erythro*-hexapyranosyl)-α-D-glucopyranoside (6e)

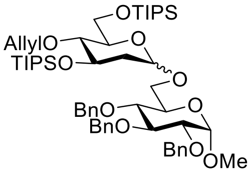

Following the general procedure, glucal **5e** (75 mg, 0.15 mmol), glucoside **3a** (46 mg, 0.099 mmol) and a CH_2Cl_2 stock solution of TsOH·H_2O (1 mL, 1 mol%) were used. Purification by column chromatography (Hexane:EtOAc, 95:5 to 8:2) afforded the product **6e** as a syrup (77 mg, 81%, α:β = 15:1). **α-anomer:** ^{1}H NMR (400 MHz, $CDCl_3$) δ: 7.38-7.23 (15H, m, Ph), 5.89 (1H, ddt, *J* = 17.2, 10.5, 5.8 Hz, C*H*=CH_2), 5.18 (1H, dq, *J* = 17.1, 1.7 Hz, CH=C*H*H), 5.11-5.06 (1H, m, CH=CH*H*), 4.98 (1H, d, *J* = 11.0 Hz, OC*H*HPh), 4.89 (1H, d, *J* = 11.0 Hz, OC*H*HPh), 4.87 (1H, br d, *J* = 2.9 Hz, H-1'), 4.82 (1H, d, *J* = 11.0 Hz, OCH*H*Ph), 4.78 (1H, d, *J* = 12.2 Hz, OC*H*HPh), 4.58 (1H, d, *J* = 12.2 Hz, OCH*H*Ph), 4.58 (1H, d, *J* = 11.0 Hz, OCH*H*Ph), 4.57 (1H, d, *J* = 3.6 Hz, H-1), 4.34 (1H, ddt, *J* = 12.3, 5.4, 1.4 Hz, C*H*HCH=CH_2), 4.23 (1H, ddd, *J* = 11.1, 8.6, 4.9 Hz, H-3'), 4.09 (1H, ddt, *J* = 12.3, 5.8, 1.4 Hz, CH*H*CH=CH_2), 3.98 (1H, dd, *J* = 9.6, 8.9 Hz, H-3), 3.82 (1H, dd, *J* = 11.0, 2.0 Hz, H-6a or H-6a'), 3.80-3.71 (3H, m, H-5 and, H-6a and H-6b' or H-6a' and H-6b), 3.60-3.51 (2H, m, H-5' and H6b or H6b'), 3.49 (1H, dd, *J* = 9.6, 3.6 Hz, H-2), 3.45 (1H, t, *J* = 9.3 Hz, H-4), 3.35 (3H, s, OCH_3), 3.18 (1H, t, *J* = 9.2 Hz, H-4'), 2.14-2.06 (1H, m, H-2a'), 1.66-1.57 (1H, m, H-2b'), 1.14-0.98 (42H, m, 2 x Si(C*H*(CH_3)$_2$)$_3$); ^{13}C NMR (126 MHz, $CDCl_3$) δ: 139.0 (4° C), 138.5 (4° C), 138.3 (4° C), 135.5 (C*H*=CH_2), 128.6 (CH), 128.51 (CH), 128.49 (CH), 128.2 (CH), 128.0 (CH), 127.74 (CH), 127.69 (CH), 116.1 (CH=CH_2), 97.9 (C-1), 97.5 (C-1'), 82.3 (C-3), 80.1 (C-2), 79.8 (C-4'), 78.4 (C-4), 75.8 (O*C*H_2Ph), 75.2 (O*C*H_2Ph), 73.9 (O*C*H_2CH=CH_2), 73.4 (O*C*H_2Ph), 72.9 (C-5'), 70.8 (C-3'), 70.3 (C-5), 65.3, 63.1 (C-6 and C-6'), 55.0 (OCH_3), 39.5 (C-2'), 18.3 (Si(CH(*C*H_3)$_2$)), 18.22 (Si(CH(*C*H_3)$_2$)), 18.17 (Si(CH(*C*H_3)$_2$)), 18.1 (Si(CH(*C*H_3)$_2$)), 12.9 (Si(*C*H(CH_3)$_2$)), 12.2 (Si(*C*H(CH_3)$_2$)). **β-anomer:** ^{1}H NMR (400 MHz, $CDCl_3$) δ: 3.36 (3H, s, OCH_3). ESI-HRMS for $C_{55}H_{86}NaO_{10}Si_2^+$ (MNa^+) calculated: 985.5652; found: 985.5634.

Methyl 2,3,4-tri-*O*-benzyl-6-*O*-(4-*O*-allyl-6-*O*-triisopropylsilyl-2,3-dideoxy-α/β-D-*erythro*-hex-2-enopyranosyl)-α-D-glucopyranoside (7e)

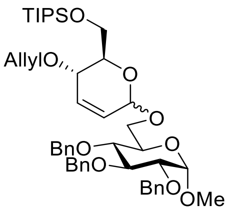

Further elution of the above column afforded the Ferrier rearrangement product **7e** as a syrup (2 mg, 4%, α:β = 3:1). **α-anomer:** ^{1}H NMR (400 MHz, $CDCl_3$) δ: 7.39-7.23 (15H, m, Ph), 5.04 (1H, br s, H-1'), 4.61 (1H, d, *J* = 3.6 Hz, H-1), 3.37 (3H, s, OCH_3), 1.10-1.00 (21H, m, Si(C*H*(C*H*$_3$)$_2$)$_3$); ^{13}C NMR (101 MHz, $CDCl_3$) δ: 139.0 (4° C), 138.6 (4° C), 138.4 (4° C), 135.0 (C*H*=CH_2), 131.2, 126.5 (C-2', C-3'), 117.1 (CH=C*H*$_2$), 98.2 (C-1), 94.9 (C-1'), 82.2 (CH), 80.02 (CH), 77.9 (CH), 75.8 (O*C*H$_2$Ph), 75.0 (O*C*H$_2$Ph), 73.5 (O*C*H$_2$Ph), 71.0 (CH), 70.3 (CH and *C*H$_2$CH=CH_2), 69.8 (CH), 66.6 (CH_2), 62.6, 55.3 (OCH_3), 18.1 (Si(CH(*C*H$_3$)$_2$)), 12.1 (Si(*C*H(CH$_3$)$_2$)). **β-anomer:** ^{1}H NMR (400 MHz, $CDCl_3$) δ: 7.39-7.23 (15H, m, Ph), 4.94 (1H, dd, *J* = 3.3, 1.6 Hz, H-1'), 4.58 (1H, d, *J* = 3.7 Hz, H-1), 3.36 (3H, s, OCH_3), 1.10-1.00 (21H, m, Si(C*H*(C*H*$_3$)$_2$)$_3$); ^{13}C NMR (101 MHz, $CDCl_3$) δ: 138.9 (4° C), 138.5 (4° C), 138.3 (4° C), 134.9 (C*H*=CH_2), 117.3 (CH=C*H*$_2$), 98.1 (C-1), 95.2 (C-1'), 82.4 (CH), 80.04 (CH), 77.7 (CH), 76.8 (CH), 75.9 (O*C*H$_2$Ph), 75.1 (O*C*H$_2$Ph), 70.2, 69.9 (CH), 68.9 (CH), 66.1 (CH_2), 63.4 (CH_2), 55.1 (OCH_3), 18.06 Si (CH(*C*H$_3$)$_2$). ESI-HRMS for $C_{46}H_{64}NaO_9Si^+$ (MNa$^+$) calculated: 811.4212; found: 811.4225.

Methyl 2,3,4-tri-*O*-benzyl-6-*O*-(4,6-*O*-benzylidene-3-*O*-triisopropylsilyl-2-deoxy-α/β-D-*erythro*-hexapyranosyl)-α-D-glucopyranoside (6f)

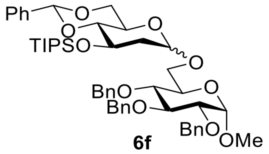

6f

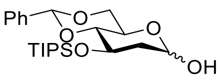

Hydrolysed-5f

Following the general procedure, glucal **5f** (59 mg, 0.15 mmol), glucoside **3a** (46 mg, 0.099 mmol) and a CH_2Cl_2 stock solution of TsOH·H_2O (1 mL, 1 mol%) were used. Purification by column chromatography (Hexane:EtOAc, 9:1 to 8:2) afforded an inseparable mixture of the product **6f** and **hydrolysed-5f** as a syrup (36 mg, **6f** ~39%, α:β = 3:1, **hydrolysed-5f ~5%**).

6f α-anomer: ^{1}H NMR (400 MHz, $CDCl_3$) δ: 5.53 (1H, s, PhC*H*O_2), 4.95 (1H, br s, H-1'), 4.62 (1H, d, *J* = 3.7 Hz, H-1), 3.373 (3H, s, OCH_3); ^{13}C NMR (101 MHz, $CDCl_3$) δ: 102.0 (Ph*C*HO_2), 98.5 (C-1'), 97.9 (C-1), 55.1 (OCH_3). **6f β-anomer:** ^{1}H NMR (400 MHz, $CDCl_3$) δ: 5.50 (1H, s, PhC*H*O_2), 4.38 (1H, dd, *J* = 10.0, 2.2 Hz, H-1'), 3.369 (3H, s, OCH_3); ^{13}C NMR (101 MHz, $CDCl_3$) δ: 101.9 (Ph*C*HO_2), 100.5 (C-1'), 98.2 (C-1), 55.3 (OCH_3). ESI-HRMS for $C_{50}H_{66}NaO_{10}Si^+$ (MNa^+) calculated: 877.4317; found: 877.4310.

Hydrolysed-5f ^{1}H NMR (400 MHz, $CDCl_3$) δ: 5.77 (1H, s, PhC*H*O_2), 5.35 (1H, d, *J* = 3.6 Hz, H-1). ESI for $C_{22}H_{36}NaO_5Si^+$ (MNa^+) calculated: 431.2; found: 431.3.

Methyl 2,3,4-tri-*O*-benzyl-6-*O*-(4,6-*O*-[bis(*tert*-butyl)silylene]-3-*O*-triisopropylsilyl-α/β-D-*erythro*-hexapyranosyl)-α-D-glucopyranoside (6g)

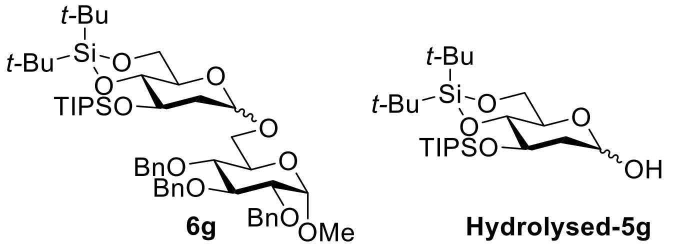

6g **Hydrolysed-5g**

Following the general procedure, glucal **5g** (66 mg, 0.15 mmol), glucoside **3a** (46 mg, 0.099 mmol) and a CH_2Cl_2 stock solution of TsOH·H_2O (1 mL, 1 mol%) were used. Purification by column chromatography (Hexane:EtOAc, 95:5 to 9:1) afforded an inseparable mixture of the product **6g** and **hydrolysed-5g** as a syrup (36 mg, **6g** ~34%, α:β = 3:1, **hydrolysed-5g** ~10%). **6g α-anomer:** ^{1}H NMR (400 MHz, $CDCl_3$) δ: 4.83 (1H, br d, *J* = 3.0 Hz, H-1'), 4.51 (1H, d, *J* = 3.6 Hz, H-1), 3.34 (3H, s, OCH_3); ^{13}C NMR (101 MHz, $CDCl_3$) δ: 97.8 (C-1, C-1'), 54.9 (OCH_3). **6g β-anomer:** ^{1}H NMR (400 MHz, $CDCl_3$) δ: 4.59 (1H, d, *J* = 3.6 Hz, H-1), 4.33 (1H, dd, *J* = 10.0, 2.0 Hz, H-1'), 3.36 (3H, s, OCH_3); ^{13}C NMR (101 MHz, $CDCl_3$) δ: 100.2 (C-1'), 98.2 (C-1), 55.3 (OCH_3). ESI-HRMS for $C_{51}H_{78}NaO_{10}Si_2^+$ (MNa^+) calculated: 929.5026; found: 929.5001.

Hydrolysed-5g ^{1}H NMR (400 MHz, $CDCl_3$) δ: 5.28 (1H, d, *J* = 3.5 Hz, H-1). ESI-HRMS for $C_{23}H_{48}NaO_5Si_3^+$ (MNa^+) calculated: 483.2932; found: 483.2925.

Methyl 2,3,4-tri-*O*-benzyl-6-*O*-(6-*O*-benzyl-2-deoxy-3,4-*O*-(1,1,3,3-tetraisopropyldisiloxane-1,3-diyl)-α-D-*erythro*-hexapyranosyl)-α-D-glucopyranoside (6h)

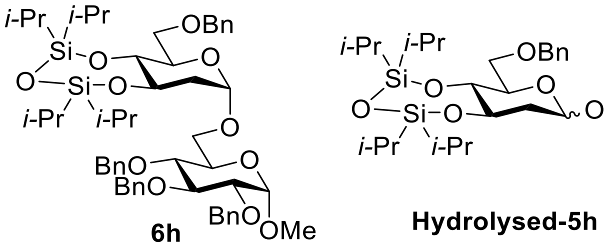

Following the general procedure, glucal **5h** (57 mg, 0.12 mmol), glucoside **3a** (43 mg, 0.093 mmol) and a CH_2Cl_2 stock solution of TsOH·H_2O (1 mL, 1 mol%) were used. Purification by column chromatography (Hexane:EtOAc, 9:1 to 8:2) afforded an inseparable mixture of the product **6h** and **hydrolysed-5h** as a syrup (74 mg, **6h** ~76%, **hydrolysed-5h** ~5%). **6h** ^{1}H NMR (400 MHz, $CDCl_3$) δ: 7.38-7.23 (20H, m, Ph), 4.99 (1H, d, *J* =3.5 Hz, H-1'), 4.98 (1H, d, *J* = 10.7 Hz, OC*H*HPh), 4.91 (1H, d, *J* = 11.0 Hz, OC*H*HPh), 4.81 (1H, d, *J* = 10.8 Hz, OCH*H*Ph), 4.79 (1H, d, *J* = 12.0 Hz, OC*H*HPh), 4.67 (1H, d, *J* = 12.0 Hz, OCH*H*Ph), 4.61 (1H, d, *J* = 10.9 Hz, OCH*H*Ph), 4.60 (1H, d, *J* = 3.8 Hz, H-1), 4.55 (1H, d, *J* = 12.0 Hz, OC*H*HPh), 4.50 (1H, d, *J* = 12.0 Hz, OCH*H*Ph), 4.05 (1H, ddd, *J* = 11.5, 8.0, 5.2 Hz, H-3'), 4.00 (1H, t, *J* = 9.3 Hz, H-3), 3.83 (1H, dd, *J* = 11.2, 5.0 Hz, H-6a or H-6a'), 3.80-3.74 (1H, m, CH), 3.73-3.67 (1H, m, CH), 3.66-3.59 (4H, m, CH, (H-6a or H-6a'), H-6b, H-6b'), 3.52 (1H, dd, *J* = 9.6, 3.6 Hz, H-2), 3.48 (1H, dd, *J* = 9.7, 9.3 Hz, H-4), 3.36 (3H, s, OCH_3), 2.12 (1H, app dd, *J* = 13.2, 5.2 Hz, H-2a'), 1.73 (1H, ddd, *J* = 13.3, 11.6, 3.6 Hz, H-2b'), 1.12-0.82 (28H, m, 4 x Si(C*H*(C*H*$_3$)$_2$); ^{13}C NMR (101 MHz, $CDCl_3$) δ: 138.8 (4° C), 138.44 (4° C), 138.39 (4° C), 138.3 (4° C), 128.6 (CH), 128.54 (CH), 128.51 (CH), 128.3 (CH), 128.2 (CH), 128.1 (CH), 128.0 (CH), 127.81 (CH), 127.75 (CH), 127.6 (CH), 127.5 (CH), 98.0 (C-1), 97.8 (C-1'), 82.4 (C-3), 80.2 (C-2), 78.2 (C-4), 75.9 (O*C*H$_2$Ph), 75.1 (O*C*H$_2$Ph), 74.5 (CH), 73.53 (O*C*H$_2$Ph), 73.47 (O*C*H$_2$Ph), 71.9 (C-3'), 71.8 (CH), 70.1 (CH), 69.4 (CH_2), 65.7 (CH_2), 55.0 (OCH_3), 38.1 (C-2'), 17.8 (Si(CH(*C*H$_3$)$_2$)), 17.54 (Si(CH(*C*H$_3$)$_2$)), 17.50 (Si(CH(*C*H$_3$)$_2$)), 17.47 (Si(CH(*C*H$_3$)$_2$)), 17.46 (Si(CH(*C*H$_3$)$_2$)), 17.4 (Si(CH(*C*H$_3$)$_2$)), 13.2 (Si(*C*H(CH$_3$)$_2$)), 13.0 (Si(*C*H(CH$_3$)$_2$)), 12.5 (Si(*C*H(CH$_3$)$_2$)), 12.4 (Si(*C*H(CH$_3$)$_2$)). MALDI-HRMS for $C_{53}H_{74}NaO_{11}Si_2^+$ (MNa$^+$) calculated: 965.4662; found: 965.4665.

Hydrolysed-5h ^{1}H NMR (400 MHz, $CDCl_3$) δ: 5.39 (1H, d, *J* = 3.5 Hz, H-1). ESI-HRMS for $C_{25}H_{44}NaO_6Si_2^+$ (MNa$^+$) calculated: 496.2676; found: 496.2681.

Methyl 2,3,4-tri-*O*-benzyl-6-*O*-(2-deoxy-3,4-*O*-(1,1,3,3-tetraisopropyldisiloxane-1,3-diyl)-6-*O*-triisopropylsilyl-α-D-*erythro*-hexapyranosyl)-α-D-glucopyranoside (6i)

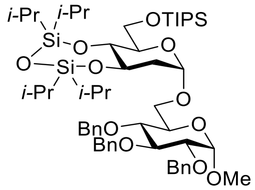

Following the general procedure, glucal **5i** (65 mg, 0.12 mmol), glucoside **3a** (43 mg, 0.093 mmol) and a CH_2Cl_2 stock solution of TsOH·H_2O (1 mL, 1 mol%) were used. Purification by column chromatography (Hexane:EtOAc, 95:5 to 92:8) afforded the product **6i** as a syrup (80 mg, 85%). ^{1}H NMR (400 MHz, $CDCl_3$) δ: 7.38-7.24 (15H, m, Ph), 4.97 (1H, d, *J* = 10.8 Hz, OC*H*HPh), 4.94 (1H, br d, *J* = 3.1 Hz, H-1'), 4.88 (1H, d, *J* = 11.0 Hz, OC*H*HPh), 4.81 (1H, d, *J* = 10.8 Hz, OCH*H*Ph), 4.79 (1H, d, *J* = 11.9 Hz, OC*H*HPh), 4.67 (1H, d, *J* = 12.1 Hz, OCH*H*Ph), 4.60 (1H, d, *J* = 10.9 Hz, OCH*H*Ph), 4.57 (1H, d, *J* = 3.6 Hz, H-1), 4.05 (1H, ddd, *J* = 11.3, 8.2, 5.2 Hz, H-3'), 4.01-3.96 (2H, m, H-3 and H-6a or H-6a'), 3.82 (1H, d, *J* = 11.2, 5.6 Hz, H-6a or H-6a'), 3.78-3.72 (1H, m, CH), 3.73 (1H, dd, *J* = 10.7, 6.6 Hz, H-6b or H-6b'), 3.65-3.59 (2H, m, CH and H-6b or H-6b'), 3.50 (1H, dd, *J* = 9.6, 3.6 Hz, H-2), 3.47-3.40 (2H, m, H-4, H-4'), 3.35 (3H, s, OCH_3), 2.09 (1H, ddd, *J* = 13.3, 5.2, 1.1 Hz, H-2a'), 1.65 (1H, ddd, *J* = 13.2, 11.3, 3.5 Hz, H-2b'), 1.11-0.86 (49H, m, 7 x $Si(CH(CH_3)_2)$); ^{13}C NMR (126 MHz, $CDCl_3$) δ: 138.9 (4° C), 138.4 (4° C), 138.3 (4° C), 128.6 (CH), 128.54 (CH), 128.51 (CH), 128.21 (CH), 128.15 (CH), 128.0 (CH), 127.77 (CH), 127.75 (CH), 127.7 (CH), 97.9 (C-1), 97.2 (C-1'), 82.4 (C-3), 80.2 (C-2), 78.5 (CH), 76.0 (OCH$_2$Ph), 75.2 (OCH$_2$Ph), 74.9 (CH), 73.6 (CH), 73.5 (OCH$_2$Ph), 71.9 (C-3'), 70.3 (CH), 65.3, 63.7 (C-6,C-6'), 54.9 (OCH_3), 38.2 (C-2'), 18.2 ($Si(CH(CH_3)_2)$), 18.1 ($Si(CH(CH_3)_2)$), 17.8 ($Si(CH(CH_3)_2)$), 17.54 ($Si(CH(CH_3)_2)$), 17.50 ($Si(CH(CH_3)_2)$), 17.45 ($Si(CH(CH_3)_2)$), 17.41 ($Si(CH(CH_3)_2)$), 17.38 ($Si(CH(CH_3)_2)$), 13.1 ($Si(CH(CH_3)_2)$), 13.0 ($Si(CH(CH_3)_2)$), 12.5 ($Si(CH(CH_3)_2)$), 12.3 ($Si(CH(CH_3)_2)$), 12.2 ($Si(CH(CH_3)_2)$). ESI-HRMS for $C_{55}H_{88}NaO_{11}Si_3^+$ (MNa^+) calculated: 1031.5527; found: 1031.5532. $[\alpha]_D^{22}$ = +49 (*c* = 0.0066, $CHCl_3$).

Methyl 2,3,4-tri-*O*-benzyl-6-*O*-(2-deoxy-3,4-*O*-(1,1,3,3-tetramethyldisiloxane-1,3-diyl)-6-*O*-triisopropylsilyl-α-D-*erythro*-hexapyranosyl)-α-D-glucopyranoside (6j)

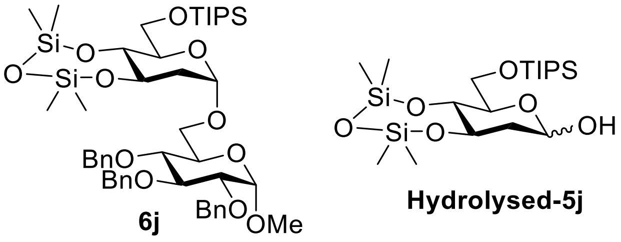

Following the general procedure, glucal **5j** (56 mg, 0.12 mmol), glucoside **3a** (46 mg, 0.099 mmol) and a CH_2Cl_2 stock solution of TsOH·H_2O (1 mL, 1 mol%) were used. Purification by column chromatography (Hexane:EtOAc, 95:5 to 9:1) afforded an inseparable mixture of the product **6j** and **hydrolysed-5j** as a syrup (56 mg, **5j** ~61%, **hydrolysed-5j** ~2%). **6j** ^{1}H NMR (400 MHz, $CDCl_3$) δ: 7.40-7.22 (15H, m, Ph), 4.98 (1H, d, *J* = 10.7 Hz, OC*H*HPh), 4.93 (1H, d, *J* = 11.1 Hz, OC*H*HPh), 4.93-4.91 (1H, m, H-1'), 4.81 (1H, d, *J* = 10.7 Hz, OCH*H*Ph), 4.88 (1H, d, *J* = 12.2 Hz, OC*H*HPh), 4.67 (1H, d, *J* = 12.1 Hz, OCH*H*Ph), 4.61 (1H, d, *J* = 3.6 Hz, H-1), 4.60 (1H, d, *J* = 11.1 Hz, OCH*H*Ph), 4.05 (1H, ddd, *J* = 11.2, 8.4, 4.9 Hz, H-3'), 4.00 (1H, t, *J* = 9.3 Hz, H-3), 3.80 (1H, dd, *J* = 11.1, 4.8 Hz, H-6a or H-6a'), 3.79-3.70 (3H, m, CH, H-6a and H-6b, or H-6a' and H-6b'), 3.63 (1H, dd, J = 11.2, 1.7 Hz, H-6b or H-6b'), 3.56-3.50 (3H, m, 3 x CH), 3.49 (1H, dd, J = 9.8, 9.2 Hz, CH), 3.37 (3H, s, OCH_3), 2.10 (1H, ddd, *J* = 13.0, 5.2, 1.1 Hz, H-2a'), 1.66 (1H, ddd, *J* = 13.2, 11.4, 3.35 Hz, H-2b'), 1.06-1.00 (21H, m, Si(C*H*(C*H*$_3$)$_2$), 0.16 (3H, s, $SiCH_3$), 0.15 (3H, s, $SiCH_3$), 0.14 (3H, s, $SiCH_3$), 0.11 (3H, s, $SiCH_3$); ^{13}C NMR (101 MHz, $CDCl_3$) δ: 138.8 (4° C), 138.5 (4° C), 138.3 (4° C), 128.61 (CH), 128.57 (CH), 128.5 (CH), 128.24 (CH), 128.22 (CH), 128.1 (CH), 127.81 (CH), 127.76 (CH), 127.7 (CH), 98.0 (C-1), 97.5 (C-1'), 82.4 (C-3), 80.2 (CH), 78.1 (CH), 76.0 (PhC*H*$_2$O), 75.1 (PhC*H*$_2$O), 73.8 (CH), 73.5 (PhC*H*$_2$O), 73.2 (CH), 71.8 (C-3'), 70.1 (CH), 65.5, 62.8 (C-6, C-6'), 55.1 (OCH_3), 38.1 (C-2'), 18.2 (Si(CH(*C*H$_3$)$_2$)), 18.12 (Si(CH(*C*H$_3$)$_2$)), 12.2 (Si(*C*H(CH$_3$)$_2$)), -0.8 ($SiCH_3$), -1.0 ($SiCH_3$), -2.00 ($SiCH_3$), -2.02 ($SiCH_3$). MALDI-HRMS for $C_{47}H_{72}NaO_{11}Si_3^+$ (MNa^+) calculated: 919.4275; found: 919.4270.

Hydrolysed-5j ^{1}H NMR (400 MHz, $CDCl_3$) δ: 5.38 (1H, d, *J* = 3.5 Hz, H-1). ESI-HRMS for $C_{19}H_{42}NaO_6Si_3^+$ (MNa^+) calculated: 473.2187; found: 473.2252.

Acceptor Scope with Glucal 5i

Phenyl 2,3,4-tri-*O*-benzoyl-6-*O*-(2-deoxy-3,4-*O*-(1,1,3,3-tetraisopropyldisiloxane-1,3-diyl)-6-*O*-triisopropylsilyl-α-D-*erythro*-hexapyranosyl)-β-D-thioglucopyranoside (8b)

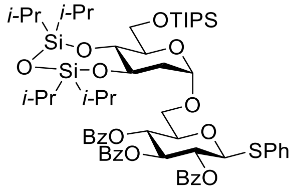

Following the general procedure, glucal **5i** (65 mg, 0.12 mmol), glucoside **3b** (59 mg, 0.10 mmol) and a CH_2Cl_2 stock solution of $TsOH{\cdot}H_2O$ (1 mL, 1 mol%) were used. Purification by column chromatography (Hexane:EtOAc, 95:5 to 92:8) afforded the product **8b** as a syrup (90 mg, 80%). ^{1}H NMR (400 MHz, $CDCl_3$) δ: 7.99-7.94 (2H, m, Ph), 7.91-7.86 (2H, m, Ph), 7.81-7.76 (2H, m, Ph), 7.56-7.23 (14H, m, Ph), 5.85 (1H, t, J = 9.5 Hz, H-3), 5.53 (1H, t, J = 9.7 Hz, H-4), 5.42 (1H, dd, J = 9.9, 9.5 Hz, H-2), 4.99 (1H, d, J = 10.0 Hz, H-1), 4.88 (1H, br. d, J = 3.3 Hz, H-1'), 4.05-3.96 (2H, m, H-3', H-5), 3.94-3.87 (2H, m, H-6a, H-6a'), 3.72 (1H, dd, J = 10.7, 5.8 Hz, H-6b'), 3.66 (1H, dd, J = 11.4, 3.3 Hz, H-6b), 3.50 (1H, ddd, J = 9.7, 6.0, 1.8 Hz, H-5'), 3.44 (1H, dd, J = 9.5, 8.0 Hz, H-4'), 1.94 (1H, app dd, J = 13.3, 5.2 Hz, H-2a'), 1.59 (1H, ddd, J = 13.5, 11.4, 3.8 Hz, H-2a'), 1.19-0.83 (49H, m, 7 x Si(C*H*(C*H*$_3$)$_2$); ^{13}C NMR (101 MHz, $CDCl_3$) δ: 166.0 (C=O), 165.2 (C=O), 165.0 (C=O), 133.42, 133.39, 133.3, 133.2, 132.0, 130.0, 129.9, 129.5, 129.3, 129.1, 129.0, 128.52, 128.48, 128.40, 128.35, 97.5 (C-1'), 86.26 (C-1), 77.4 (C-5 or C-3'), 74.7 (C-4'), 74.6 (C-3), 73.6 (C-5'), 71.7 (C-5 or C-3'), 70.8 (C-2), 69.9 (C-4), 66.2 (C-6), 63.4 (C-6'), 38.1 (C-2'), 18.14 (Si(CH(*C*H$_3$)$_2$)), 18.11 (Si(CH(*C*H$_3$)$_2$)), 17.8 (Si(CH(*C*H$_3$)$_2$)), 17.60 (Si(CH(*C*H$_3$)$_2$)), 17.59 (Si(CH(*C*H$_3$)$_2$)), 17.54 (Si(CH(*C*H$_3$)$_2$)), 17.52 (Si(CH(*C*H$_3$)$_2$)), 17.48 (Si(CH(*C*H$_3$)$_2$)), 17.45 (Si(CH(*C*H$_3$)$_2$)), 13.1 (Si(*C*H(CH$_3$)$_2$)), 13.0 (Si(*C*H(CH$_3$)$_2$)), 12.53 (Si(*C*H(CH$_3$)$_2$)), 12.48 (Si(*C*H(CH$_3$)$_2$)), 12.1 (Si(*C*H(CH$_3$)$_2$)). MALDI-HRMS for $C_{60}H_{84}NaO_{13}SSi_3^+$ (MNa$^+$) calculated: 1151.4833; found: 1151.4860. $[\alpha]_D^{22}$ = +45 (c = 0.004, $CHCl_3$).

Methyl 3-*O*-benzyl-2-*O*-(2-deoxy-3,4-*O*-(1,1,3,3-tetraisopropyldisiloxane-1,3-diyl)-6-*O*-triisopropylsilyl-α-D-*erythro*-hexapyranosyl)-4,6-*O*-benzylidene-α-D-glucopyranoside (8c)

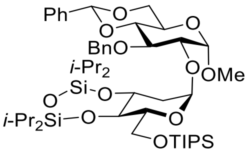

Following the general procedure, glucal **5i** (66 mg, 0.12 mmol), glucoside **3c** (38 mg, 0.10 mmol) and a CH_2Cl_2 stock solution of $TsOH{\cdot}H_2O$ (1 mL, 1 mol%) were used. Purification by column chromatography (Hexane:EtOAc, 95:5 to 92:8) afforded the product **8c** as a syrup (76 mg, 81%). ^{1}H NMR (400 MHz, $CDCl_3$) δ: 7.50-7.44 (2H, m, Ph), 7.41-7.31 (5H, m, Ph), 7.25-7.20 (3H, m, Ph), 5.53 (1H, s, PhC*H*O_2), 5.09 (1H, d, *J* = 3.3 Hz, H-1'), 4.99 (1H, d, *J* = 3.3 Hz, H-1), 4.88 (1H, d, *J* = 11.7 Hz, OC*H*HPh), 4.78 (1H, d, *J* = 11.7 Hz, OCH*H*Ph), 4.29 (1H, dd, *J* = 9.9, 4.5 Hz, C*H*H), 4.13 (1H, ddd, *J* = 11.3, 8.3, 5.3 Hz, H-3'), 4.07-4.01 (1H, m, C*H*H), 3.94 (1H, dd, *J* = 9.4, 3.4 Hz, H-2), 3.92 (1H, dd, *J* = 9.4, 8.9 Hz, H-3), 3.87-3.75 (3H, m, H-5, H-5', CH*H*), 3.71 (1H, t, *J* = 10.1 Hz, CH*H*), 3.61 (1H, t, *J* = 9.1 Hz, H-4), 3.51 (1H, dd, *J* = 9.2, 8.4 Hz, H-4'), 3.43 (3H, s, OCH_3), 2.25 (1H, app dd, *J* = 13.2, 5.1 Hz, H-2a'), 1.73 (1H, ddd, *J* = 13.5, 11.4, 3.7 Hz, H-2b'), 1.17-0.86 (49H, m, 7 x SiC*H*(C*H*$_3$)$_2$); ^{13}C NMR (101 MHz, $CDCl_3$) δ: 139.0 (4° C), 137.6 (4° C), 129.0 (CH), 128.28 (CH), 128.25 (CH), 127.7 (CH), 127.4 (CH), 126.2 (CH), 101.5 (PhC*H*O_2), 97.4 (C-1), 92.9 (C-1'), 81.5 (H-4), 77.3 (C-3), 75.2 (PhC*H*$_2$O), 74.7 (C-4'), 73.7 (CH), 73.5 (C-2), 71.9 (C-3'), 69.2, 63.5 (C-6, C-6'), 62.6 (CH), 55.3 (OCH_3), 38.0 (C-2'), 18.24 (Si(CH(*C*H$_3$)$_2$)), 18.20 (Si(CH(*C*H$_3$)$_2$)), 18.15 (Si(CH(*C*H$_3$)$_2$)), 17.7 (Si(CH(*C*H$_3$)$_2$)), 17.6 (Si(CH(*C*H$_3$)$_2$))0, 17.59 (Si(CH(*C*H$_3$)$_2$)), 17.53 (Si(CH(*C*H$_3$)$_2$)), 17.48 (Si(CH(*C*H$_3$)$_2$)), 17.45 (Si(CH(*C*H$_3$)$_2$)), 17.4 (Si(CH(*C*H$_3$)$_2$)), 13.1 (Si(*C*H(CH$_3$)$_2$)), 12.5 (Si(*C*H(CH$_3$)$_2$)), 12.4 (Si(*C*H(CH$_3$)$_2$)), 12.2 (Si(*C*H(CH$_3$)$_2$)), 12.2 (Si(*C*H(CH$_3$)$_2$)). MALDI-HRMS for $C_{48}H_{80}NaO_{11}Si_3^+$ (MNa^+) calculated: 939.4869; found: 939.4896. $[\alpha]_D^{22}$ = +52 (*c* = 0.0031, $CHCl_3$).

Phenyl 4,6-*O*-benzylidene-3-*O*-(2-deoxy-3,4-*O*-(1,1,3,3-tetraisopropyldisiloxane-1,3-diyl)-6-*O*-triisopropylsilyl-α-D-*erythro*-hexapyranosyl)-2-deoxy-1-thio-2-(2,2,2-trichloroethoxycarbonylamino)-β-D-glucopyranoside (8d)

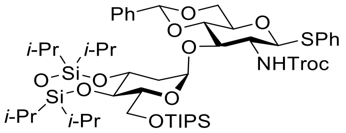

Following the general procedure, glucal **5i** (66 mg, 0.12 mmol), glucoside **3d** (53 mg, 0.099 mmol) and a CH_2Cl_2 stock solution of $TsOH{\cdot}H_2O$ (2 mL, 2 mol%) were used. Purification by column chromatography (Hexane:EtOAc, 95:5 to 92:8) afforded the product **8d** as a syrup (87 mg, 81%). ^{1}H NMR (500 MHz, $CDCl_3$) δ: 7.49-7.28 (10H, m, Ph), 5.88 (1H , d, *J* = 9.4 Hz, NH), 5.55 (1H, s, PhC*H*O_2), 5.31 (1H, d, *J* = 4.2 Hz, H-1'), 5.07 (1H, d, *J* = 12.0 Hz, C*H*HCCl_3), 4.72 (1H, d, *J* = 10.3 Hz, H-1), 4.52 (1H, d, *J* = 12.0 Hz, CH*H*CCl_3), 4.35 (1H, dd, *J* = 10.6, 5.0 Hz, C*H*H), 4.16 (1H, dd, *J* = 10.1, 1.9 Hz, C*H*H), 4.06 (1H, t, *J* = 9.7 Hz, CH), 3.92 (1H, ddd, *J* = 11.2, 8.2, 5.6 Hz, H-3'), 3.90-3.80 (3H, m, 2 x CH and CH*H*), 3.74 (2H, m, CH and CH*H*), 3.45 (1H, td, *J* = 9.7, 5.0 Hz, CH), 3.37 (1H, dd, *J* = 9.4, 8.4 Hz, H-4'), 2.16 (1H, dd, *J* = 13.7, 5.6 Hz, H-2a'), 1.69 (1H, ddd, *J* = 13.7, 11.2, 4.5 Hz, H-2b'), 1.16-0.81 (49H, m, 7 x SiC*H*(C*H*$_3$)$_2$); ^{13}C NMR (126 MHz, $CDCl_3$) δ: 154.7 (C=O), 137.2 (4° C), 133.7 (4° C), 132.3 (CH), 129.2 (CH), 129.1 (CH), 128.4 (CH), 128.0 (CH), 126.0 (CH), 101.3 (PhC*H*O_2), 97.7 (C-1'), 95.7 (CH_2*C*Cl_3), 89.9 (C-1), 82.1 (CH), 75.3 (C-4'), 75.1 (CH), 74.7 (*C*H_2CCl_3), 72.7 (CH), 72.1 (C-3'), 70.7 (CH), 68.7, 64.2 (C-6 and C6'), 55.9 (CH), 38.1 (C-2'), 18.2 (Si(CH(*C*H_3)$_2$)), 17.8 (Si(CH(*C*H_3)$_2$)), 17.6 (Si(CH(*C*H_3)$_2$)), 17.49 (Si(CH(*C*H_3)$_2$)), 17.46 (Si(CH(*C*H_3)$_2$)), 17.4 (Si(CH(*C*H_3)$_2$)), 12.99 (Si(*C*H(CH_3)$_2$)), 12.97 (Si(*C*H(CH_3)$_2$)), 12.6 (Si(*C*H(CH_3)$_2$)), 12.4 (Si(*C*H(CH_3)$_2$)), 12.3 (Si(*C*H(CH_3)$_2$)). MALDI-HRMS for $C_{49}H_{78}Cl_3NNaO_{11}SSi_3^+$ (MNa^+) calculated: 1100.3561; found: 1100.3580. $[\alpha]_D^{22}$ = +35 (*c* = 0.0017, $CHCl_3$).

***O*-(2-Deoxy-3,4-*O*-(1,1,3,3-tetraisopropyldisiloxane-1,3-diyl)-6-*O*-triisopropylsilyl-α-D-*erythro*-hexapyranosyl)-*N*-(*tert*-butoxycarbonyl)-L-serine methyl ester (8e)**

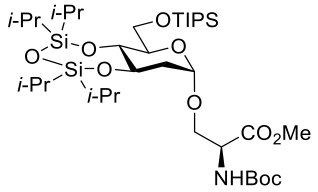

Following the general procedure, glucal **5i** (66 mg, 0.12 mmol), serine **3e** (22 mg, 0.10 mmol) and a CH_2Cl_2 stock solution of $TsOH·H_2O$ (1 mL, 1 mol%) were used. Purification by column chromatography (Hexane:EtOAc, 95:5 to 92:8) afforded the product **8e** as a syrup (61 mg, 80%). ^{1}H NMR (500 MHz, $CDCl_3$) δ: 5.25 (1H, d, *J* = 8.9 Hz, NH), 4.84 (1H, br d, *J* = 3.2 Hz, H-1), 4.52-4.40 (1H, m, C*H*CO_2Me), 4.09-3.94 (2H, m, H-6a and H-3), 3.88 (1H, dd, *J* = 10.2, 3.9 Hz, OC*H*HCH), 3.84-3.73 (2H, m, H-6b and CH*H*CH), 3.74 (3H, s, OCH_3), 3.55-3.44 (2H, m, H-4 and H-5), 2.03 (1H, dd, *J* = 13.4, 5.3 Hz, H-2a), 1.65 (1H, ddd, *J* = 13.4, 11.4, 3.6 Hz, H-2b), 1.16-0.98 (49H, m, 7 x SiC*H*(C*H*$_3$)$_2$); ^{13}C NMR (126 MHz, $CDCl_3$) δ: 171.4 (CH(*C*=O)OCH_3), 155.5 (O(C=O)N), 97.6 (C-1), 80.1 (*C*(CH_3)$_3$), 74.5, 73.9 (C-4 and C-5), 71.7 (C-3), 67.2 (OC*H*$_2$CH), 63.4 (C-6), 53.9 (C*H*CO_2Me), 52.5 (OCH_3), 38.0 (C-2), 28.4 (C(*C*H$_3$)$_3$), 18.13 (Si(CH(*C*H$_3$)$_2$)), 18.11 (Si(CH(*C*H$_3$)$_2$)), 18.09 (Si(CH(*C*H$_3$)$_2$)), 17.8 (Si(CH(*C*H$_3$)$_2$)), 17.6 (Si(CH(*C*H$_3$)$_2$)), 17.49 (Si(CH(*C*H$_3$)$_2$)), 17.45 (Si(CH(*C*H$_3$)$_2$)), 17.44 (Si(CH(*C*H$_3$)$_2$)), 17.42 (Si(CH(*C*H$_3$)$_2$)), 17.40 (Si(CH(*C*H$_3$)$_2$)), 13.2 (Si(*C*H(CH_3)$_2$)), 13.0 (Si(*C*H(CH_3)$_2$)), 12.5 (Si(*C*H(CH_3)$_2$)), 12.4 (Si(*C*H(CH_3)$_2$)), 12.1 (Si(*C*H(CH_3)$_2$)). MALDI-HRMS for $C_{36}H_{73}NNaO_{10}Si_3^+$ (MNa^+) calculated: 786.4434; found: 786.4424. $[\alpha]_D^{22}$ = +51 (*c* = 0.043, $CHCl_3$).

***O*-(2-Deoxy-3,4-*O*-(1,1,3,3-tetraisopropyldisiloxane-1,3-diyl)-6-*O*-triisopropylsilyl-α-D-*erythro*-hexapyranosyl)-*N*-[(9-fluorenylmethoxy)carbonyl]-L-serine methyl ester (8f)**

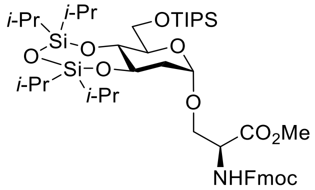

Following the general procedure, glucal **5i** (68 mg, 0.12 mmol), serine **3f** (35 mg, 0.10 mmol) and a CH_2Cl_2 stock solution of TsOH·H_2O (1 mL, 1 mol%) were used. Purification by column chromatography (Hexane:EtOAc, 95:5 to 85:15) afforded the product **8f** as a syrup (81 mg, 89%). ^{1}H NMR (500 MHz, $CDCl_3$) δ: 7.77 (2H, d, *J* = 7.5 Hz, Ph), 7.61 (2H, dd, *J* = 7.5, 3.5 Hz, Ph), 7.40 (2H, t, *J* = 7.4 Hz, Ph), 7.31 (2H, t, *J* = 7.4 Hz, Ph), 5.58 (1H, d, *J* = 8.8 Hz, NH), 4.86 (1H, br d, *J* = 3.0 Hz, H-1), 4.56 (1H, dt, *J* = 8.4, 4.1 Hz, NCH), 4.43 (1H, dd, *J* = 10.4, 7.2 Hz, C*H*H), 4.33 (1H, dd, *J* = 10.6, 7.3 Hz, CH*H*), 4.25 (1H, t, *J* = 7.3 Hz, CH), 4.07 (1H, dd, *J* = 10.7, 1.8 Hz, H-6a), 4.01 (1H, ddd, *J* = 11.0, 7.8, 5.3 Hz, H-3), 3.93 (1H, dd, *J* = 10.4, 3.9 Hz, C*H*H), 3.86 (1H, dd, *J* = 10.4, 4.2 Hz, CH*H*), 3.83 (1H, s, J = 10.6, 6.1 Hz, H-6b), 3.77 (3H, s, OCH_3), 3.58-3.45 (2H, m, H-4, H-5), 2.07 (1H, dd, *J* = 13.3, 5.3 Hz, H-2a), 1.68 (1H, ddd, *J* = 13.5, 11.4, 4.0 Hz, H-2b), 1.15-0.99 (49H, m, 7 x SiC*H*(C*H*$_3$)$_2$); ^{13}C NMR (126 MHz, $CDCl_3$) δ: 170.9 (CH(*C*=O)OCH_3), 156.0 (O(C=O)N), 143.9 (4° C), 143.8 (4° C), 141.27 (4° C), 141.26 (4° C), 127.7 (CH), 127.08 (CH), 127.06 (CH), 125.1 (CH), 120.0 (CH), 97.8 (C-1), 74.4, 73.9 (C-4 and C-5), 71.5 (C-3), 67.4 (CH_2), 67.2 (CH_2), 63.3 (C-6), 54.2 (CH), 52.5 (OCH_3), 47.1 (CH), 37.9 (C-2), 18.00 (Si(CH(*C*H$_3$)$_2$)), 17.97 (Si(CH(*C*H$_3$)$_2$)), 17.95 (Si(CH(*C*H$_3$)$_2$)), 17.6 (Si(CH(*C*H$_3$)$_2$)), 17.41 (Si(CH(*C*H$_3$)$_2$)), 17.35 (Si(CH(*C*H$_3$)$_2$)), 17.32 (Si(CH(*C*H$_3$)$_2$)), 17.30 (Si(CH(*C*H$_3$)$_2$)), 17.28 (Si(CH(*C*H$_3$)$_2$)), 17.26 (Si(CH(*C*H$_3$)$_2$)), 13.0 (Si(*C*H(CH$_3$)$_2$)), 12.9 (Si(*C*H(CH$_3$)$_2$)), 12.4 (Si(*C*H(CH$_3$)$_2$)), 12.3 (Si(*C*H(CH$_3$)$_2$)), 12.0 (Si(*C*H(CH$_3$)$_2$)). MALDI-HRMS for $C_{46}H_{75}NNaO_{10}Si_3^+$ (MNa^+) calculated: 908.4591; found: 908.4578. $[\alpha]_D^{22}$ = +43 (*c* = 0.0028, $CHCl_3$).

***O*-(2-Deoxy-3,4-*O*-(1,1,3,3-tetraisopropyldisiloxane-1,3-diyl)-6-*O*-triisopropylsilyl-α-D-*erythro*-hexapyranosyl)-*N*-(*tert*-butoxycarbonyl)-L-threonine methyl ester (8g)**

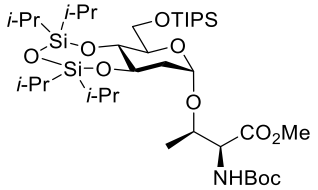

Following the general procedure, glucal **5i** (66 mg, 0.12 mmol), threonine **3g** (24 mg, 0.10 mmol) and a CH_2Cl_2 stock solution of TsOH·H_2O (1 mL, 1 mol%) were used. Purification by column chromatography (Hexane:EtOAc, 95:5 to 92:8) afforded the product **8g** as a white solid (79 mg, 86%). ^{1}H NMR (500 MHz, $CDCl_3$) δ: 5.24 (1H, d, *J* = 8.9 Hz, NH), 4.83 (1H, d, *J* = 3.1 Hz, H-1), 4.33-4.23 (2H, m, 2 x CH), 4.07 (1H, dd, *J* = 10.5, 1.7 Hz, H-6a), 3.95 (1H, ddd, *J* = 11.6, 8.3, 5.3 Hz, H-3), 3.75 (1H, dd, *J* = 10.6, 6.9 Hz, H-6b), 3.72 (3H, s, OCH_3), 3.68-3.60 (1H, m, H-5), 3.38 (1H, t, *J* = 8.9 Hz, H-4), 1.93 (1H, dd, *J* = 13.4, 5.3 Hz, H-2a), 1.60 (1H, ddd, *J* = 13.4, 11.4, 3.8 Hz, H-2b), 1.47 (9H, s, $C(CH_3)_3$), 1.32 (3H, d, *J* = 6.6 Hz, $OCHCH_3$), 1.13-0.98 (49H, m, 7 x $SiCH(CH_3)_2$); ^{13}C NMR (126 MHz, $CDCl_3$) δ: 171.8 ($CH(C{=}O)OCH_3$), 156.2 (O(C=O)N), 98.9 (C-1), 80.1 ($C(CH_3)_3$), 75.7 (CH), 75.0 (C-4), 74.1 (C-5), 71.5 (C-3), 63.9 (C-6), 58.4 (CH), 52.4 (OCH_3), 38.6 (C-2), 28.5 ($C(CH_3)_3$), 18.8 ($OCHCH_3$), 18.1 ($Si(CH(CH_3)_2)$), 17.7 ($Si(CH(CH_3)_2)$), 17.6 ($Si(CH(CH_3)_2)$), 17.53 ($Si(CH(CH_3)_2)$), 17.48 ($Si(CH(CH_3)_2)$), 17.43 ($Si(CH(CH_3)_2)$), 17.40 ($Si(CH(CH_3)_2)$), 13.09 ($Si(CH(CH_3)_2)$), 13.06 ($Si(CH(CH_3)_2)$), 12.5 ($Si(CH(CH_3)_2)$), 12.1 ($Si(CH(CH_3)_2)$). MALDI-HRMS for $C_{37}H_{75}NNaO_{10}Si_3^+$ (MNa^+) calculated: 800.4591; found: 800.4585. $[\alpha]_D^{22}$ = +41 (*c* = 0.0017, $CHCl_3$).

Cholesteryl (2-deoxy-3,4-*O*-(1,1,3,3-tetraisopropyldisiloxane-1,3-diyl)-6-*O*-tri-isopropylsilyl-α/β-D-*erythro*-hexapyranosyl) (8h)

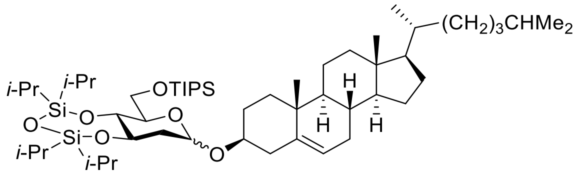

Following the general procedure, glucal **5i** (68 mg, 0.12 mmol), cholesterol **3h** (39 mg, 0.10 mmol) and a CH_2Cl_2 stock solution of TsOH·H_2O (1 mL, 1 mol%) were used. Purification by column chromatography (Hexane:EtOAc, 95:5 to 8:2) afforded the product **8h** as a syrup (77 mg, 82%, α:β = 20:1). **α-anomer:** ^{1}H NMR (500 MHz, $CDCl_3$) δ: 5.33-5.29 (1H, m, C=CH), 5.09 (1H, br d, *J* = 3.5 Hz, H-1), 4.10 (1H, dd, *J* = 10.7, 1.8 Hz, H-6a), 4.05 (1H, ddd, *J* = 11.2, 8.2, 5.3 Hz, H-3), 3.74 (1H, dd, *J* = 10.6, 7.3 Hz, H-6b), 3.65 (1H, ddd, *J* = 9.3, 7.3, 1.8 Hz, H-5), 3.61-3.50 (1H, m, CH), 3.37 (1H, dd, *J* = 9.5, 8.2 Hz, H-4), 2.30 (1H, d, *J* = 7.8 Hz, CH_2), 2.05-1.89 (4H, m, H-2a and 3 x C*H*H), 1.68 (1H, ddd, *J* = 13.2, 11.3, 3.8 Hz, H-2b), 1.61-1.20 (13H, m), 1.20-0.93 (63H, m), 0.92 (3H, d, *J* = 6.5 Hz, CHC*H*$_3$), 0.867 (3H, d, *J* = 6.6 Hz, $CHCH_3$), 0.862 (3H, d, *J* = 6.6 Hz, $CHCH_3$), 0.68 (3H, s, CCH_3); ^{13}C NMR (126 MHz, $CDCl_3$) δ: 140.9 (*C*=CH), 121.6 (C=*C*H), 93.6 (C-1), 75.4 (C-4), 74.4 (CH), 73.5 (C-5), 71.9 (C-3), 64.1 (C-6), 57.0 (CH), 56.3 (CH), 50.3 (CH), 42.5 (4° C), 39.99 (CH_2), 39.97 (CH_2), 39.7 (CH_2), 38.7 (C-2), 37.1 (CH_2), 36.9 (4° C), 36.4 (CH_2), 35.9 (CH), 32.13, 32.05 (CH and CH_2), 28.4 (CH_2), 28.2 (CH), 27.1 (CH_2), 24.5 (CH_2), 24.0 (CH_2), 23.0 (CH_3), 22.7 (CH_3), 21.2 (CH_2), 19.6 (CH_3), 18.9 (CH_3), 18.2 (Si(CH(*C*H$_3$)$_2$)), 17.8 (Si(CH(*C*H$_3$)$_2$)), 17.59 (Si(CH(*C*H$_3$)$_2$)), 17.55 (Si(CH(*C*H$_3$)$_2$)), 17.54 (Si(CH(*C*H$_3$)$_2$)), 17.52 (Si(CH(*C*H$_3$)$_2$)), 17.46 (Si(CH(*C*H$_3$)$_2$)), 17.45 (Si(CH(*C*H$_3$)$_2$)), 13.1 (Si(*C*H(CH$_3$)$_2$)), 13.0 (Si(*C*H(CH$_3$)$_2$)), 12.51 (Si(*C*H(CH$_3$)$_2$)), 12.49 (Si(*C*H(CH$_3$)$_2$)), 12.2 (Si(*C*H(CH$_3$)$_2$)), 12.0 (CH_3). **β-anomer:** ^{1}H NMR (500 MHz, $CDCl_3$) δ: 5.20 (1H, app d, *J* = 3.4 Hz, C=CH), 4.74 (1H, dd, *J* = 10.2, 2.0 Hz, H-1). MALDI-HRMS for $C_{54}H_{102}NaO_6Si_3^+$ (MNa^+) calculated: 953.6876; found: 953.6880.

Acceptor Scope with Rhamnal 9

Methyl 2,3,4-tri-*O*-benzyl-6-*O*-(2,6-deoxy-3,4-*O*-(1,1,3,3-tetraisopropyldisiloxane-1,3-diyl)-α/β-L-*erythro*-hexapyranosyl)-α-D-glucopyranoside (11a)

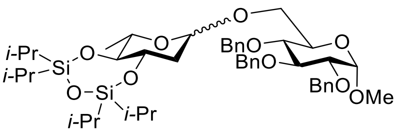

Following the general procedure, rhamnal **9** (46 mg, 0.12 mmol), glucoside **3a** (49 mg, 0.11 mmol) and a CH_2Cl_2 stock solution of TsOH·H_2O (1 mL, 1 mol%) were used. Purification by column chromatography (Hexane:EtOAc, 95:5 to 85:15) afforded the product **11a** as a syrup (71 mg, 81%, α:β = 9:1). **α-anomer:** ^{1}H NMR (400 MHz, $CDCl_3$) δ: 7.40-7.23 (15H, m, Ph), 4.99 (1H, d, *J* = 10.8 Hz, OC*H*HPh), 4.89 (1H, d, *J* = 11.0 Hz, OC*H*HPh), 4.82 (1H, d, *J* = 10.8 Hz, OCH*H*Ph), 4.80 (1H, d, *J* = 12.2 Hz, OC*H*HPh), 4.78 (1H, br d, *J* = 3.2 Hz, H-1'), 4.66 (1H, d, *J* = 12.1 Hz, OCH*H*Ph), 4.58 (1H, d, *J* = 3.5 Hz, H-1), 4.54 (1H, d, *J* = 11.0 Hz, OCH*H*Ph), 4.01 (1H, t, *J* = 9.4 Hz, H-3), 3.98 (1H, ddd, *J* = 11.0, 8.7, 5.1 Hz, H-3'), 3.88 (1H, dd, *J* = 10.7, 1.7 Hz, H-6a), 3.79 (1H, ddd, *J* = 10.1, 6.9, 1.7 Hz, H-5), 3.67-3.58 (1H, m, H-5'), 3.51 (1H, dd, *J* = 9.6, 3.5 Hz, H-2), 3.41 (1H, dd, *J* = 10.7, 6.8 Hz, H-6b), 3.37 (1H, dd, *J* = 10.2, 8.9 Hz, H-4), 3.37 (3H, s, OCH_3), 3.32 (1H, t, *J* = 8.7 Hz, H-4'), 3.32 (1H, ddd, *J* = 13.3, 5.2, 1.1 Hz, H-2a'), 2.08 (1H, ddd, *J* = 13.3, 11.3, 3.7 Hz, H-2b'), 1.24 (3H, d, *J* = 6.2 Hz, H-6), 1.11-0.86 (28H, m, 4 x $Si(CH(CH_3)_2)$); ^{13}C NMR (101 MHz, $CDCl_3$) δ: 138.8 (4° C), 138.3 (4° C), 128.61 (CH), 128.59 (CH), 128.56 (CH), 128.3 (CH), 128.2 (CH), 128.1 (CH), 128.0 (CH), 127.8 (CH), 97.9 (C-1), 97.7 (C-1'), 82.3 (C-3), 80.2 (CH), 80.1 (CH), 78.5 (CH), 76.0 (O*C*H_2Ph), 75.2 (O*C*H_2Ph), 73.6 (O*C*H_2Ph), 71.5 (C-3'), 70.4 (CH), 68.2 (C-5'), 66.3 (C-6), 54.9 (OCH_3), 38.5 (C-2'), 18.1 (C-6'), 17.7 (Si(CH(*C*H_3)$_2$)), 17.6 (Si(CH(*C*H_3)$_2$)), 17.53 (Si(CH(*C*H_3)$_2$)), 17.49 (Si(CH(*C*H_3)$_2$)), 17.43 (Si(CH(*C*H_3)$_2$)), 17.40 (Si(CH(*C*H_3)$_2$)), 13.1 (Si(*C*H(CH_3)$_2$)), 13.0 (Si(*C*H(CH_3)$_2$)), 12.49 (Si(*C*H(CH_3)$_2$)), 12.45 (Si(*C*H(CH_3)$_2$)). **β-anomer:** ^{1}H NMR (400 MHz, $CDCl_3$) δ: 4.61 (1H, d, *J* = 3.6 Hz, H-1), 4.22 (1H, dd, *J* = 11.1, 3.1 Hz, H-6a), 2.21 (1H, ddd, *J* = 12.9, 5.3, 1.9 Hz, H-2a'), 1.29 (3H, d, *J* = 6.3 Hz, H-6'); ^{13}C NMR (101 MHz, $CDCl_3$) δ: 98.2 (C-1), 55.3 (OCH_3), 18.1 (C-6'). MALDI-HRMS for $C_{46}H_{68}NaO_{10}Si_2^+$ (MNa^+) calculated: 859.4243; found: 859.4244.

Phenyl 4,6-*O*-benzylidene-3-*O*-(2,6-deoxy-3,4-*O*-(1,1,3,3-tetraisopropyldisiloxane-1,3-diyl)-α-L-*erythro*-hexapyranosyl)-2-deoxy-1-thio-2-(2,2,2-trichloroethoxycarbonyl-amino)-β-D-glucopyranoside (11d)

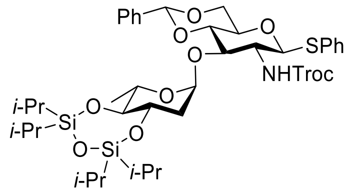

Following the general procedure, rhamnal **9** (44 mg, 0.12 mmol), glucoside **3d** (53 mg, 0.099 mmol) and a CH_2Cl_2 stock solution of TsOH·H_2O (2 mL, 2 mol%) were used. Purification by column chromatography (Hexane:EtOAc, 95:5 to 9:1) afforded the product **11d** as a white solid (76 mg, 84%). ^{1}H NMR (500 MHz, $CDCl_3$) δ: 7.53-7.40 (4H, m, Ph), 7.38-7.27 (m, 6H), 5.54 (1H, s, PhC*H*O_2), 5.21 (1H, d, *J* = 9.0 Hz, NH), 4.99 (1H, d, *J* = 3.5 Hz, H-1'), 4.96 (1H, d, *J* = 10.4 Hz, H-1), 4.79 (1H, d, *J* = 12.0 Hz, C*H*HCCl_3), 4.74 (1H, d, *J* = 12.0 Hz, CH*H*CCl_3), 4.39 (1H, dd, *J* = 10.6, 4.8 Hz, H-6a), 4.06 (1H, t, *J* = 9.1 Hz, CH), 3.87 (1H, ddd, *J* = 11.3, 8.3, 5.3 Hz, H-3'), 3.82-3.74 (2H, m, H-5' and H-6b), 3.67-3.52 (3H, m, 3 x CH), 3.09 (1H, dd, *J* = 9.4, 8.3 Hz, H-4'), 2.04 (1H, dd, *J* = 13.2, 5.4 Hz, H-2a'), 1.58 (1H, ddd, *J* = 13.2, 11.4, 4.1 Hz, H2b'), 1.11-0.83 (28H, m, 4 x $Si(CH(CH_3)_2)$), 0.69 (3H, d, *J* = 6.1 Hz, H-6'); ^{13}C NMR (126 MHz, $CDCl_3$) δ: 153.9 (C=O), 137.2 (4° C), 132.7 (CH), 132.4 (4° C), 129.3 (CH), 129.2 (CH), 128.3 (CH), 126.5 (CH), 102.3 (PhC*H*O_2), 98.3 (C-1'), 95.5 (CH_2*C*Cl_3), 87.1 (C-1), 79.9 (CH), 79.8 (C-4'), 76.4 (CH), 74.7 (*C*H_2Cl_3), 71.3 (C-3'), 71.0 (CH), 68.8 (C-6), 68.3 (C-5'), 57.9 (CH), 38.6 (C-2'), 17.9 ($Si(CH(CH_3)_2)$), 17.53 ($Si(CH(CH_3)_2)$), 17.52 ($Si(CH(CH_3)_2)$), 17.50 ($Si(CH(CH_3)_2)$), 17.49 ($Si(CH(CH_3)_2)$), 17.44 ($Si(CH(CH_3)_2)$), 17.36 (C-6'), 13.04 ($Si(CH(CH_3)_2)$), 13.03 ($Si(CH(CH_3)_2)$), 12.41 ($Si(CH(CH_3)_2)$), 12.39 ($Si(CH(CH_3)_2)$). MALDI-HRMS for $C_{40}H_{58}Cl_3NNaO_{10}Si_2^+$ (MNa^+) calculated: 928.2278; found: 928.2257. $[\alpha]_D^{21}$ = -64 (*c* = 0.0031, $CHCl_3$).

***O*-(2,6-Deoxy-3,4-*O*-(1,1,3,3-tetraisopropyldisiloxane-1,3-diyl)-α/β-L-*erythro*-hexapyranosyl)-*N*-[(9-fluorenylmethoxy)carbonyl]-L-serine methyl ester (11f)**

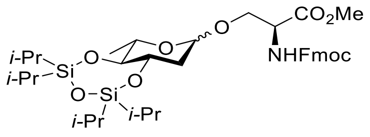

Following the general procedure, rhamnal **9** (44 mg, 0.12 mmol), serine **3f** (35 mg, 0.10 mmol) and a CH_2Cl_2 stock solution of TsOH·H_2O (1 mL, 1 mol%) were used. Purification by column chromatography (Hexane:EtOAc, 9:1 to 85:15) afforded the product **11f** as a syrup (69 mg, 95%, α:β = 10:1). **α-anomer:** ^{1}H NMR (500 MHz, $CDCl_3$) δ: 7.79-7.76 (2H, m, Ph), 7.64-7.59 (2H, m, Ph), 7.43-7.39 (2H, m, Ph), 7.35-7.29 (2H, m, Ph), 5.63 (1H, d, *J* = 8.6 Hz, NH), 4.81 (1H, d, *J* = 3.0 Hz, H-1), 4.58 (1H, dt, *J* = 8.7, 3.0 Hz, NCH), 4.46 (1H, dd, *J* = 10.7, 7.1 Hz, C*H*H), 4.39 (1H, dd, *J* = 10.7, 7.1 Hz, CH*H*), 4.25 (1H, t, *J* = 7.1 Hz, CH), 4.08 (1H, dd, *J* = 9.8, 3.2 Hz, C*H*H), 3.89 (1H, ddd, *J* = 11.4, 8.3, 5.2, H-3), 3.76 (3H, s, OCH_3), 3.59 (1H, dd, *J* = 9.8, 2.9 Hz, CH*H*), 3.44 (1H, dq, *J* = 9.4, 6.3 Hz, H-5), 3.28-3.18 (1H, m, H-4), 2.09 (1H, dd, *J* = 13.5, 5.4 Hz, H-2a), 1.70 (1H, ddd, *J* = 13.5, 11.4, 3.7, H-2b), 1.28 (3H, d, *J* = 6.3 Hz, H-6), 1.12-0.98 (28H, m, 4 x Si(C*H*(C*H*$_3$)$_2$)); ^{13}C NMR (126 MHz, $CDCl_3$) δ: 170.4 (CH(*C*=O)OCH_3), 155.9 (O(C=O)N), 143.9 (4° C), 143.7 (4° C), 141.31 (4° C), 141.30 (4° C), 127.7 (CH), 127.1 (CH), 125.1 (CH), 120.0 (CH), 119.98 (CH), 97.1 (C-1), 79.7 (C-4), 71.3 (C-3), 68.4 (C-5), 67.2 (CH_2), 66.7 (CH_2), 54.2 (CH), 52.4 (OCH_3), 47.2 (CH), 38.0 (C-2), 17.9 (C-6), 17.6 (Si(CH(*C*H$_3$)$_2$)), 17.39 (Si(CH(*C*H$_3$)$_2$)), 17.35 (Si(CH(*C*H$_3$)$_2$)), 17.31 (Si(CH(*C*H$_3$)$_2$)), 17.24 (Si(CH(*C*H$_3$)$_2$)), 12.94 (Si(*C*H(CH$_3$)$_2$)), 12.91 (Si(*C*H(CH$_3$)$_2$)), 12.3 (Si(*C*H(CH$_3$)$_2$)), 12.2 (Si(*C*H(CH$_3$)$_2$)). **β-anomer:** ^{1}H NMR (400 MHz, $CDCl_3$) δ: 6.19 (1H, d, *J* = 8.7 Hz, NH), 3.37 (3H, s, OCH_3); ^{13}C NMR (126 MHz, $CDCl_3$) δ: 100.2 (C-1), 52.6 (OCH_3). MALDI-HRMS for $C_{37}H_{55}NNaO_9Si_2^+$ (MNa^+) calculated: 736.3308; found: 736.3308.

Cholesteryl (2,6-deoxy-3,4-*O*-(1,1,3,3-tetraisopropyldisiloxane-1,3-diyl)-α-L-*erythro*-hexapyranosyl) (11h-α)

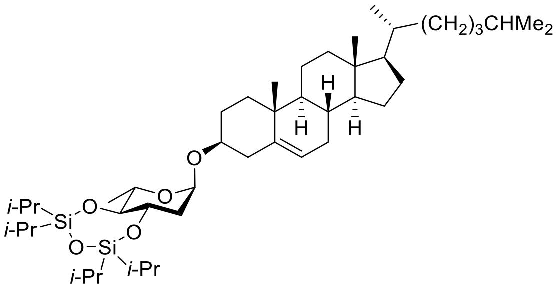

Following the general procedure, rhamnal **9** (44 mg, 0.12 mmol), cholesterol **3h** (38 mg, 0.098 mmol) and a CH_2Cl_2 stock solution of TsOH·H_2O (1 mL, 1 mol%) were used. Purification by column chromatography (Hexane:PhMe, 9:1 to 6:4) afforded the product **11h-α** as a syrup (54 mg, 72%). ^{1}H NMR (500 MHz, $CDCl_3$) δ: 5.36-5.30 (1H, m, C=CH), 4.98 (1H, br d, *J* = 3.5 Hz, H-1), 3.99 (1H, ddd, *J* = 11.1, 8.3, 5.3 Hz, H-3), 3.73-3.64 (1H, m, H-5), 3.46-3.37 (1H, m, CH), 3.22 (1H, t, *J* = 8.7 Hz, H-4), 2.35-2.28 (1H, m, C*H*H), 2.23-2.14 (1H, m, C*H*H), 2.07-1.92 (3H, m, H-2a and 2 x C*H*H), 1.88-1.78 (3H, m, 3 x C*H*H), 1.69 (1H, ddd, *J* = 13.3, 11.3, 3.9 Hz, H-2b), 1.63-0.89 (52H, m), 1.25 (3H, d, *J* = 6.5 Hz, CH_3), 0.91 (3H, d, *J* = 6.4 Hz, CH_3), 0.867 (3H, d, *J* = 6.7 Hz, CH_3), 0.863 (3H, d, *J* = 6.7 Hz, CH_3), 0.68 (3H, s, CH_3); ^{13}C NMR (126 MHz, $CDCl_3$) δ: 141.0 (*C*=CH), 121.7 (C=*C*H), 95.5 (C-1), 80.4 (C-4), 76.5 (CH), 71.7 (C-3), 68.0 (C-5), 56.9 (CH), 56.3 (CH), 50.3 (CH), 42.5 (4° C), 40.0 (CH_2), 39.7 (CH_2), 39.2 (C-2), 38.8 (CH_2), 37.6 (CH_2), 37.0 (4° C), 36.4 (CH_2), 36.0 (CH), 32.11, 32.06 (CH and CH_2), 29.7 (CH_2), 28.4 (CH_2), 28.2 (CH), 24.5 (CH_2), 24.0 (CH_2), 23.0 (CH_3), 22.7 (CH_3), 21.2 (CH_2), 19.6, 18.9 (CH_3), 18.2 (C-6), 17.8, 17.62, 17.59, 17.54, 17.53, 17.4, 13.11, 13.06, 12.6, 12.5, 12.0 (CH_3). MALDI-HRMS for $C_{45}H_{82}NaO_5Si_2^+$ (MNa^+) calculated: 781.5593; found: 781.5615. $[\alpha]_D^{21}$ = -78 (*c* = 0.0043, $CHCl_3$).

Cholesteryl (2,6-deoxy-3,4-*O*-(1,1,3,3-tetraisopropyldisiloxane-1,3-diyl)-β-L-*erythro*-hexapyranosyl) (11h-β)

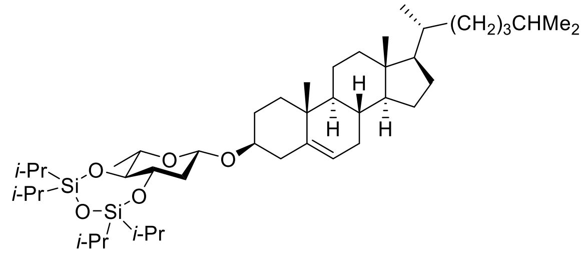

Further elution of the above column afforded glycoside **11h-β** (17 mg, 23%). ^{1}H NMR (500 MHz, $CDCl_3$) δ: 5.38-5.33 (1H, m, C=CH), 4.60 (1H, dd, *J* = 9.9, 2.0 Hz, H-1), 3.69 (1H, ddd, *J* = 11.4, 7.5, 5.3 Hz, H-3), 3.55 (1H, tt, *J* = 11.3, 4.5 Hz, CH), 3.28-3.19 (2H, m, H-4, H-5), 2.41 (1H, dd, *J* = 13.3, 5.1, 2.1 Hz, C*H*H), 2.37-2.28 (1H, m, CH*H*), 2.11 (1H, ddd, *J* = 12.9, 5.2, 2.0 Hz, H-2a), 2.04-1.93 (2H, m, 2 x C*H*H), 1.90-1.77 (3H, m, 3 x C*H*H), 1.68 (1H, ddd, *J* = 13.0, 11.6, 9.9 Hz, H-2b), 1.61-0.88 (52H, m), 1.32 (3H, d, *J* = 5.7 Hz, CH_3), 0.91 (3H, d, *J* = 6.5 Hz, CH_3), 0.856 (3H, d, *J* = 6.6 Hz, CH_3), 0.862 (3H, d, *J* = 6.6 Hz, CH_3), 0.67 (3H, s, CH_3); ^{13}C NMR (126 MHz, $CDCl_3$) δ: 141.1 (*C*=CH), 121.8 (C=*C*H), 97.8 (C-1), 79.6 (CH), 78.1 (CH), 74.3 (C-3), 72.3 (CH), 57.0 (CH), 56.3 (CH), 50.4 (CH), 42.5 (4° C), 40.5 (C-2), 40.4 (CH_2), 40.0 (CH_2), 39.7 (CH_2), 37.3 (CH_2), 36.9 (4° C), 36.4 (CH_2), 35.9 (CH), 32.1, 32.1 (CH and CH_2), 28.40 (CH_2), 28.39 (CH_2), 28.2 (CH), 24.5 (CH_2), 24.0 (CH_2), 23.0 (CH_3), 22.7 (CH_3), 21.2 (CH_2), 19.5, 18.9 (CH_3), 18.3 (C-6), 17.8, 17.6, 17.52, 17.48, 17.4, 13.06, 13.05, 12.49, 12.45, 12.0 (CH_3). MALDI-HRMS for $C_{45}H_{82}NaO_5Si_2^+$ (MNa^+) calculated: 781.5593; found: 781.5613. $[\alpha]_D^{21}$ = -38 (*c* = 0.0011, $CHCl_3$).

Rhamnal 10 scope with acceptor 3a

Methyl 2,3,4-tri-*O*-benzyl-6-*O*-(2,6-deoxy-3-*O*-acetyl-4-*O*-(*tert*-butyldimethylsilyl)-α/β-L-*erythro*-hexapyranosyl)-α-D-glucopyranoside (12)

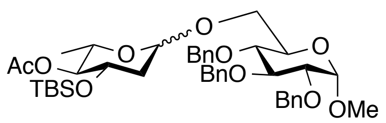

Following the general procedure, rhamnal **10** (37 mg, 0.13 mmol), glucoside **3a** (50 mg, 0.11 mmol) and a CH_2Cl_2 stock solution of $TsOH·H_2O$ (1 mL, 1 mol%) were used. Purification by column chromatography (Hexane:EtOAc, 95:5 to 85:15) afforded the product **12** as a syrup

(43 mg, 57%, α:β = 4:1). **α-anomer:** ^{1}H NMR (400 MHz, $CDCl_3$) δ: 7.40-7.23 (15H, m, Ph), 5.00 (1H, d, *J* = 10.8 Hz, OC*H*HPh), 4.90 (1H, d, *J* = 11.0 Hz, OC*H*HPh), 4.85-4.80 (2H, m, 2 x OCH*H*Ph), 4.77 (1H, br d, *J* = 3.0 Hz, H-1'), 4.68 (1H, d, *J* = 12.3 Hz, OCH*H*Ph), 4.64-4.57 (2H, m, H-4', H-1), 4.55 (1H, d, *J* = 11.0 Hz, OCH*H*Ph), 4.04-3.97 (2H, m, H-3, H-3'), 3.85 (1H, d, *J* = 10.7, 1.5 Hz, H-6a), 3.81-3.66 (2H, m, H-5, H-5'), 3.53 (1H, dd, *J* = 9.8, 3.8 Hz, H-2), 3.45-3.36 (2H, m, H-6b, H-4), 3.38 (3H, s, OCH_3), 2.07 (3H, s, CH_3), 2.03 (1H, ddd, *J* = 13.5, 5.2, 1.1 Hz, H-2a'), 1.71 (1H, ddd, *J* = 13.5, 11.4, 3.6 Hz, H-2b'), 1.10 (3H, d, *J* = 6.3 Hz, H-6'), 0.84 (9H, s, $Si(C(CH_3)_3)$), 0.02 (3H, s, $SiCH_3$), 0.02 (3H, s, $SiCH_3$); ^{13}C NMR (101 MHz, $CDCl_3$) δ: 170.1 (C=O),138.7 (4° C), 138.2 (4° C), 128.61 (CH), 128.59 (CH), 128.56 (CH), 128.3 (CH), 128.2 (CH), 128.1 (CH), 128.0 (CH), 127.8 (CH), 97.9 (C-1), 97.6 (C-1'), 82.3 (C-3), 80.2 (CH), 80.1 (C-2), 78.3 (C-4), 77.75 (C-4'), 76.0 (O*C*H_2Ph), 75.2 (O*C*H_2Ph), 73.5 (O*C*H_2Ph), 70.3 (C-5), 67.6 (CH), 66.5 (C-6), 66.1 (C-5'), 54.9 (OCH_3), 39.1 (C-2'), 25.7 ($Si(C(CH_3)_3)$), 21.3 (CH_3), 18.0 ($Si(C(CH_3)_3)$), 17.7 (C-6'), -4.44 ($Si(CH_3)_2$), -4.76 ($Si(CH_3)_2$). **β-anomer:** ^{1}H NMR (400 MHz, $CDCl_3$) δ: 4.27 (1H, dd, *J* = 11.0, 2.9 Hz, H-6a), 3.62 (1H, dd, *J* = 11.0, 1.5 Hz, H-6a), 2.21 (1H, ddd, *J* = 13.5, 6.0, 2.6 Hz, H-2a'), 1.1 (3H, d, *J* = 6.2 Hz, H-6'); ^{13}C NMR (101 MHz, $CDCl_3$) δ: 98.4 (C-1), 79.8 (C-3'), 40.4 (C-2'). MALDI-HRMS for $C_{42}H_{58}NaO_{10}Si^+$ (MNa^+) calculated: 750.3799; found: 750.3782.

Experiment with deuterated substrate

Methyl 2,3,4-tri-*O*-benzyl-6-*O*-((2*S*)-2-deoxy-(2-^{2}H)-3,4-*O*-(1,1,3,3-tetraisopropyldisiloxane-1,3-diyl)-6-*O*-triisopropylsilyl-α-D-*erythro*-hexapyranosyl)-α-D-glucopyranoside (14a) and Methyl 2,3,4-tri-*O*-benzyl-6-*O*-((2*R*)-2-deoxy-(2-^{2}H)-3,4-*O*-(1,1,3,3-tetraisopropyldisiloxane-1,3-diyl)-6-*O*-triisopropylsilyl-α-D-*erythro*-hexapyranosyl)-α-D-glucopyranoside (14b)

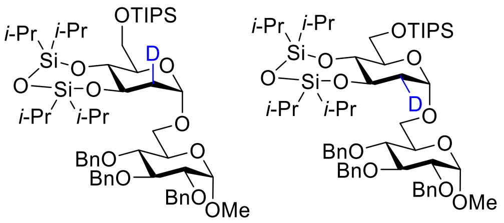

4:1

Following the general procedure, deuterated glucal **13** (66 mg, 0.12 mmol), glucoside **3a** (47 mg, 0.10 mmol) and a CH_2Cl_2 stock solution of TsOH·H_2O (1 mL, 1 mol%) were used. Purification by column chromatography (Hexane:EtOAc, 95:5 to 92:8) afforded the products **14a** and **14b** as a syrup (92 mg, 90%). ^{1}H NMR (400 MHz, $CDCl_3$) δ: 7.39-7.23 (15H, m, Ph), 4.97 (1H, d, *J* = 10.8 Hz, OC*H*HPh), 4.93 (1H, br d, *J* = 1.3 Hz, H-1'), 4.89 (d, *J* = 11.0 Hz, OC*H*HPh), 4.81 (d, *J* = 10.8 Hz, OCH*H*Ph), 4.79 (d, *J* = 12.2 Hz, OC*H*HPh), 4.67 (d, *J* = 12.2 Hz, OCH*H*Ph), 4.60 (d, *J* = 11.0 Hz, OCH*H*Ph), 4.57 (1H, d, *J* = 3.6 Hz, H-1), 4.08-4.02 (1H, m, H-3'), 4.02-3.95 (2H, m, H-3 and H-6a or H-6a'), 3.82 (1H, dd, *J* = 11.0, 5.5 Hz, H-6a or H-6a'), 3.78-3.73 (1H, m, CH), 3.74 (1H, dd, *J* = 10.6, 6.6 Hz, H-6b or H-6b'), 3.66-3.58 (2H, m, CH and H-6b or H-6b'), 3.50 (1H, dd, *J* = 9.6, 3.6 Hz, H-2), 3.47-3.40 (2H, m, H-4, H-4'), 3.35 (3H, s, OCH_3), 2.08 (0.8H, dd, *J* = 5.3, 1.2 Hz, H-2_{eq}), 1.63 (0.2H, dd, *J* = 11.4, 3.5 Hz, H-2_{ax}), 1.12-0.86 (49H, m, 7 x Si(CH(CH_3)$_2$)); ^{13}C NMR (101 MHz, $CDCl_3$) δ: 138.9 (4° C), 138.4 (4° C), 138.3 (4° C), 128.6 (CH), 128.54 (CH), 128.51 (CH), 128.21 (CH), 128.15 (CH), 128.0 (CH), 127.77 (CH), 127.75 (CH), 127.7 (CH), 97.9 (C-1), 97.2 (C-1'), 82.4 (C-3), 80.2 (C-2), 78.5 (CH), 76.0 (O*C*H_2Ph), 75.2 (O*C*H_2Ph), 74.8 (CH), 73.6 (CH), 73.5 (O*C*H_2Ph), 71.9 (C-3'), 70.3 (CH), 65.3, 63.7 (C-6,C-6'), 54.9 (OCH_3), 18.15 (Si(CH(*C*H_3)$_2$)), 18.14 (Si(CH(*C*H_3)$_2$)), 18.11 (Si(CH(*C*H_3)$_2$)), 17.8 (Si(CH(*C*H_3)$_2$)), 17.54 (Si(CH(*C*H_3)$_2$)), 17.50 (Si(CH(*C*H_3)$_2$)), 17.45 (Si(CH(*C*H_3)$_2$)), 17.41 (Si(CH(*C*H_3)$_2$)), 17.38 (Si(CH(*C*H_3)$_2$)), 13.1 (Si(*C*H(CH_3)$_2$)), 13.0 (Si(*C*H(CH_3)$_2$)), 12.5 (Si(*C*H(CH_3)$_2$)), 12.3 (Si(*C*H(CH_3)$_2$)), 12.1 (Si(*C*H(CH_3)$_2$)). ESI-HRMS for $C_{55}H_{87}DNaO_{11}Si_3^+$ (MNa^+) calculated: 1032.5589; found: 1032.5607.

References

1. (a) Chmielewski, M.; Fokt, I.; Grodner, J.; Grynkiewicz, G.; Szeja, W., *J. Carbohydr. Chem.* **1989**, *8*, 735-741; (b) Levecque, P.; Gammon, D. W.; Kinfe, H. H.; Jacobs, P.; De Vos, D.; Sels, B., *Adv. Synth. Catal.* **2008**, *350*, 1557-1568.
2. Shuto, S.; Yahiro, Y.; Ichikawa, S.; Matsuda, A., *J. Org. Chem.* **2000**, *65*, 5547-5557.
3. Lellouche, J. P.; Koeller, S., *J. Org. Chem.* **2001**, *66*, 693-696.
4. Berlin, W. K.; Zhang, W. S.; Shen, T. Y., *Tetrahedron* **1991**, *47*, 1-20.
5. (a) Kinzy, W.; Schmidt, R. R., *Tetrahedron Lett.* **1987**, *28*, 1981-1984; (b) Bartolozzi, A.; Pacciani, S.; Benvenuti, C.; Cacciarini, M.; Liguori, F.; Menichetti, S.; Nativi, C., *J. Org. Chem.* **2003**, *68*, 8529-8533.
6. Hesek, D.; Lee, M.; Zhang, W. L.; Noll, B. C.; Mobashery, S., *J. Am. Chem. Soc.* **2009**, *131*, 5187-5193.
7. Bouillot, A.; Khac, D. D.; Fetizon, M.; Guir, F.; Memoria, Y., *Synth. Commun.* **1993**, *23*, 2071-2081.
8. Adachi, M.; Yamada, H.; Isobe, M.; Nishikawat, T., *Org. Lett.* **2011**, *13*, 6532-6535.
9. Lohman, G. J. S.; Seeberger, P. H., *J. Org. Chem.* **2003**, *68*, 7541-7543.
10. Chen, C. L.; Namba, K.; Kishi, Y., *Org. Lett.* **2009**, *11*, 409-412.
11. Bucher, C.; Gilmour, R., *Angew. Chem., Int. Ed.* **2010**, *49*, 8724-8728.
12. Parker, K. A.; Georges, A. T., *Org. Lett.* **2000**, *2*, 497-499.
13. Steunenberg, P.; Jeanneret, V.; Zhu, Y. H.; Vogel, P., *Tetrahedron: Asymmetry* **2005**, *16*, 337-346.
14. Tran, A. T.; Jones, R. A.; Pastor, J.; Boisson, J.; Smith, N.; Galan, M. C., *Adv. Synth. Catal.* **2011**, *353*, 2593-2598.
15. Elchert, B.; Li, J.; Wang, J. H.; Hui, Y.; Rai, R.; Ptak, R.; Ward, P.; Takemoto, J. Y.; Bensaci, M.; Chang, C. W. T., *J. Org. Chem.* **2004**, *69*, 1513-1523.
16. Chambers, D. J.; Evans, G. R.; Fairbanks, A. J., *Tetrahedron: Asymmetry* **2003**, *14*, 1767-1769.
17. Seeberger, P. H.; Roehrig, S.; Schell, P.; Wang, Y.; Christ, W. J., *Carbohydr. Res.* **2000**, *328*, 61-69.
18. Di Bussolo, V.; Caselli, M.; Pineschi, M.; Crotti, P., *Org. Lett.* **2003**, *5*, 2173-2176.
19. Pilgrim, W.; Murphy, P. V., *Org. Lett.* **2009**, *11*, 939-942.
20. Boonyarattanakalin, S.; Liu, X. Y.; Michieletti, M.; Lepenies, B.; Seeberger, P. H., *J. Am. Chem. Soc.* **2008**, *130*, 16791-16799.
21. Tran, A. T.; Burden, R.; Racys, D. T.; Galan, M. C., *Chem. Commun.* **2011**, *47*, 4526-4528.
22. Wang, C. C.; Lee, J. C.; Luo, S. Y.; Fan, H. F.; Pai, C. L.; Yang, W. C.; Lu, L. D.; Hung, S. C., *Angew. Chem., Int. Ed.* **2002**, *41*, 2360-2362.
23. Matwiejuk, M.; Thiem, J., *Chem. Commun.* **2011**, *47*, 8379-8381.
24. Ciobanu, M.; Huang, K. T.; Daguer, J. P.; Barluenga, S.; Chaloin, O.; Schaeffer, E.; Mueller, C. G.; Mitchell, D. A.; Winssinger, N., *Chem. Commun.* **2011**, *47*, 9321-9323.
25. (a) Zhang, Z. Y.; Magnusson, G., *Carbohydr. Res.* **1996**, *295*, 41-55; (b) Miyajima, K.; Achiwa, K., *Chem. Pharm. Bull.* **1997**, *45*, 312-320; (c) Subramanian, V.; Moume-Pymbock, M.; Hu, T. S.; Crich, D., *J. Org. Chem.* **2011**, *76*, 3691-3709.
26. Benito-Alifonso, D.; Jones, R. A.; Tran, A. T.; Woodward, H.; Smith, N.; Galan, M. C., *Beilstein J. Org. Chem.* **2013**, *9*, 1867-1872.
27. Belanger, D.; Tong, X.; Soumare, S.; Dory, Y. L.; Zhao, Y., *Chem. Eur. J.* **2009**, *15*, 4428-4436.

28. Zhang, F.; Zhang, W.; Zhang, Y.; Curran, D. P.; Liu, G., *J. Org. Chem.* **2009**, *74*, 2594-2597.
29. van Dijk, M.; Postma, T. M.; Rijkers, D. T. S.; Liskamp, R. M. J.; van Nostrum, C. F.; Hennink, W. E., *Polymer* **2010**, *51*, 2479-2485.
30. Ferreira, P. M. T.; Maia, H. L. S.; Monteiro, L. S.; Sacramento, J., *J. Chem. Soc., Perkin Trans. 1* **1999**, 3697-3703.

Spectra

4-*O*-Allyl-3,6-di-*O*-triisopropylsilyl-1,2-dideoxy-2-deoxy-D-arabino-1-hexenopyranose (5e) ^{1}H NMR (400 MHz; $CDCl_3$)

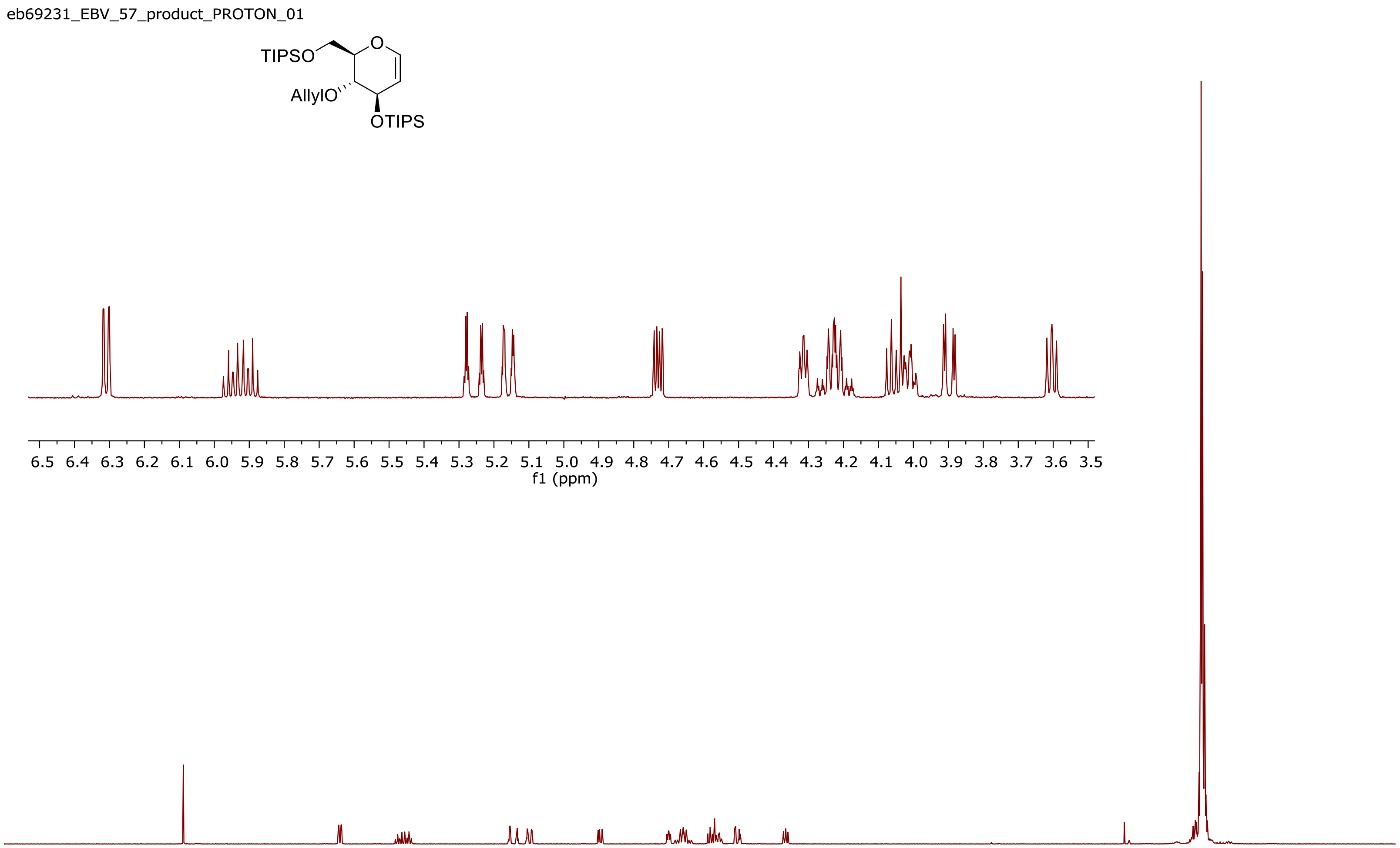

4-*O*-Allyl-3,6-di-*O*-triisopropylsilyl-1,2-dideoxy-2-deoxy-D-arabino-1-hexenopyranose (5e) ^{13}C NMR (101 MHz; $CDCl_3$)

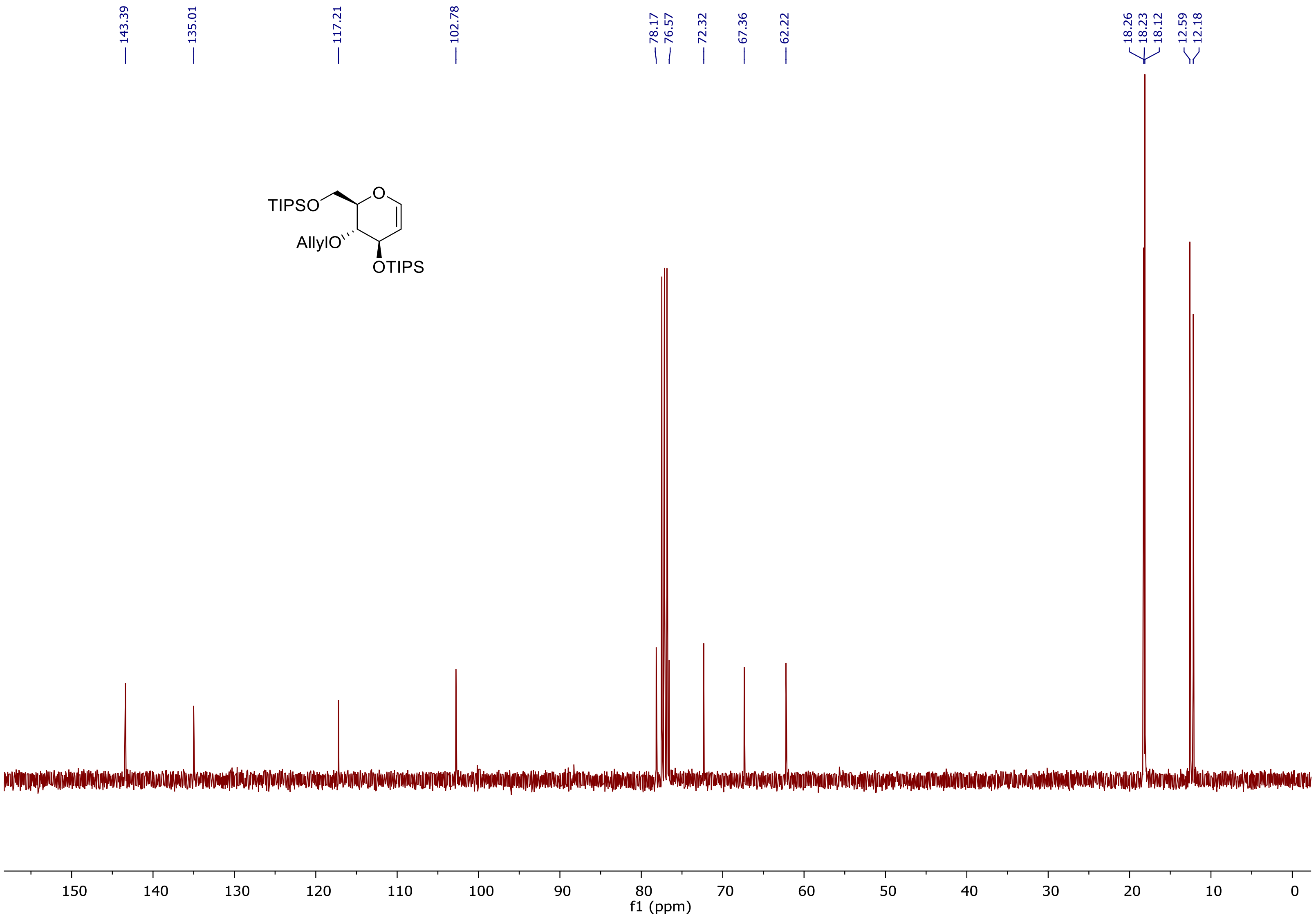

6-*O*-Benzyl-1,2-dideoxy-3,4-*O*-(1,1,3,3-tetraisopropyldisiloxane-1,3-diyl)-D-arabino-1-hexenopyranose (5h) ^{1}H NMR (400 MHz; $CDCl_3$)

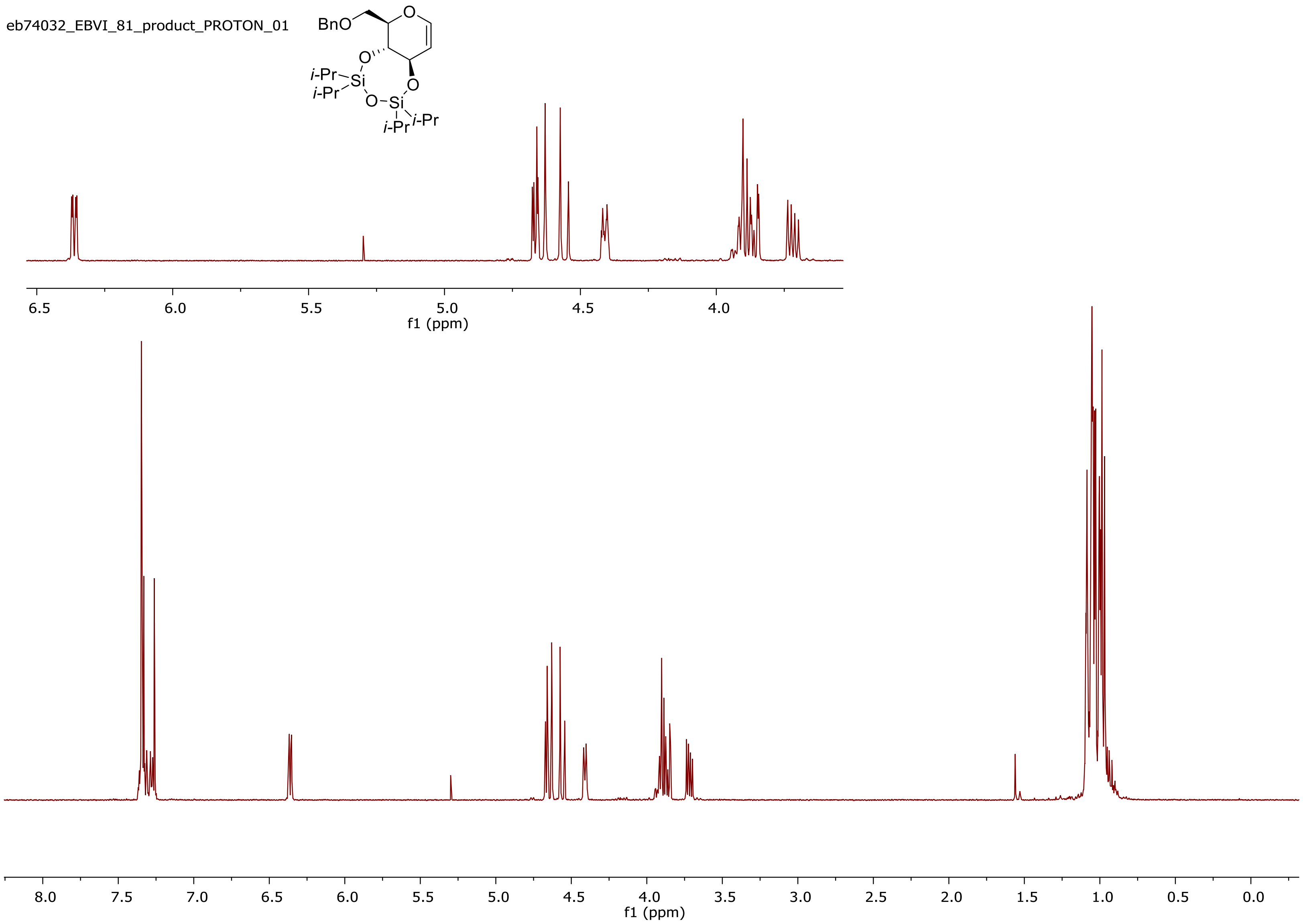

6-*O*-Benzyl-1,2-dideoxy-3,4-*O*-(1,1,3,3-tetraisopropyldisiloxane-1,3-diyl)-D-arabino-1-hexenopyranose (5h) ^{13}C NMR (101 MHz; CDCl$_3$)

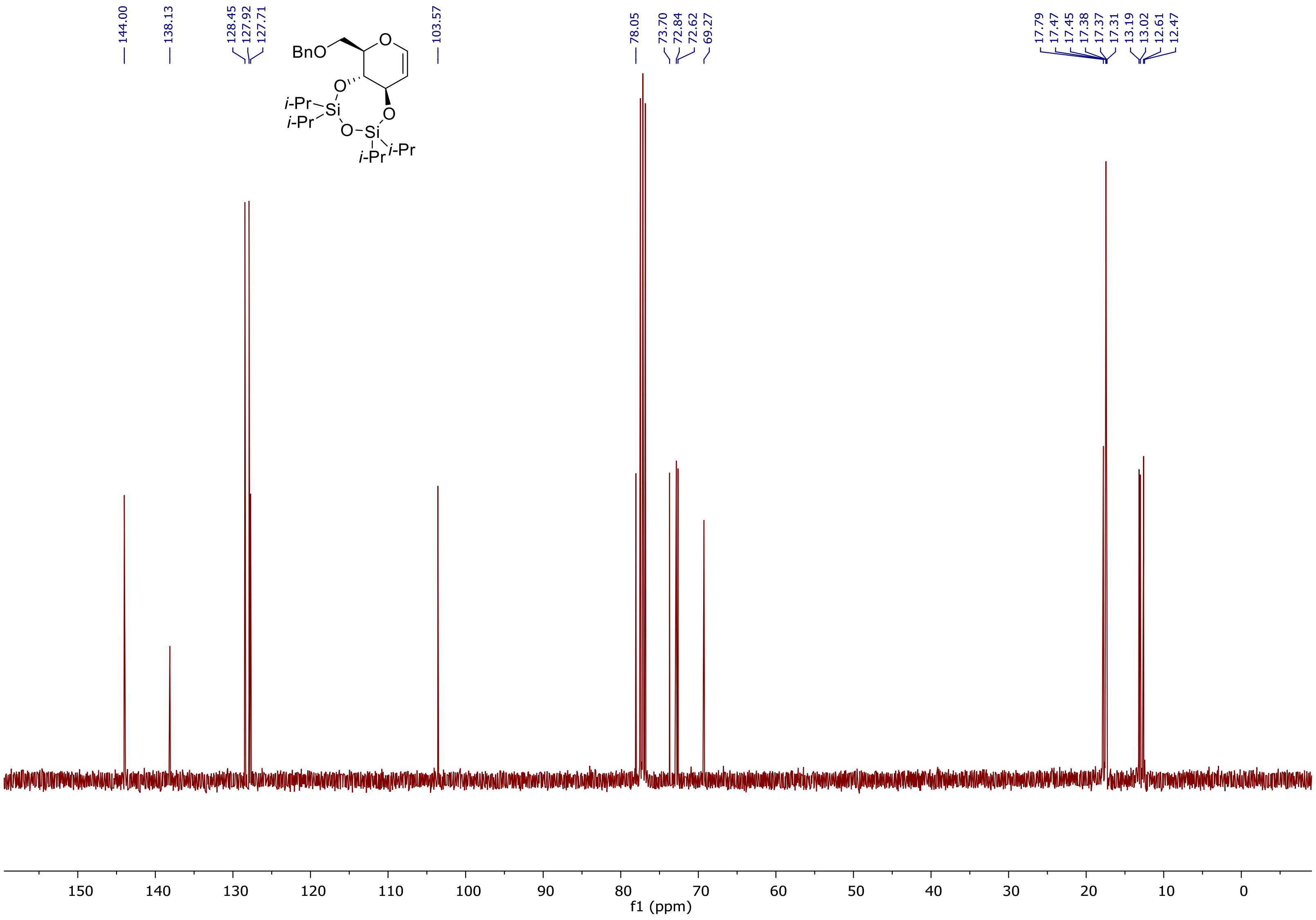

1,2-Dideoxy-3,4-*O*-(1,1,3,3-tetraisopropyldisiloxane-1,3-diyl)-6-*O*-triisopropylsilyl-D-arabino-1-hexenopyranose (5i) ^{1}H NMR (400 MHz; $CDCl_3$)

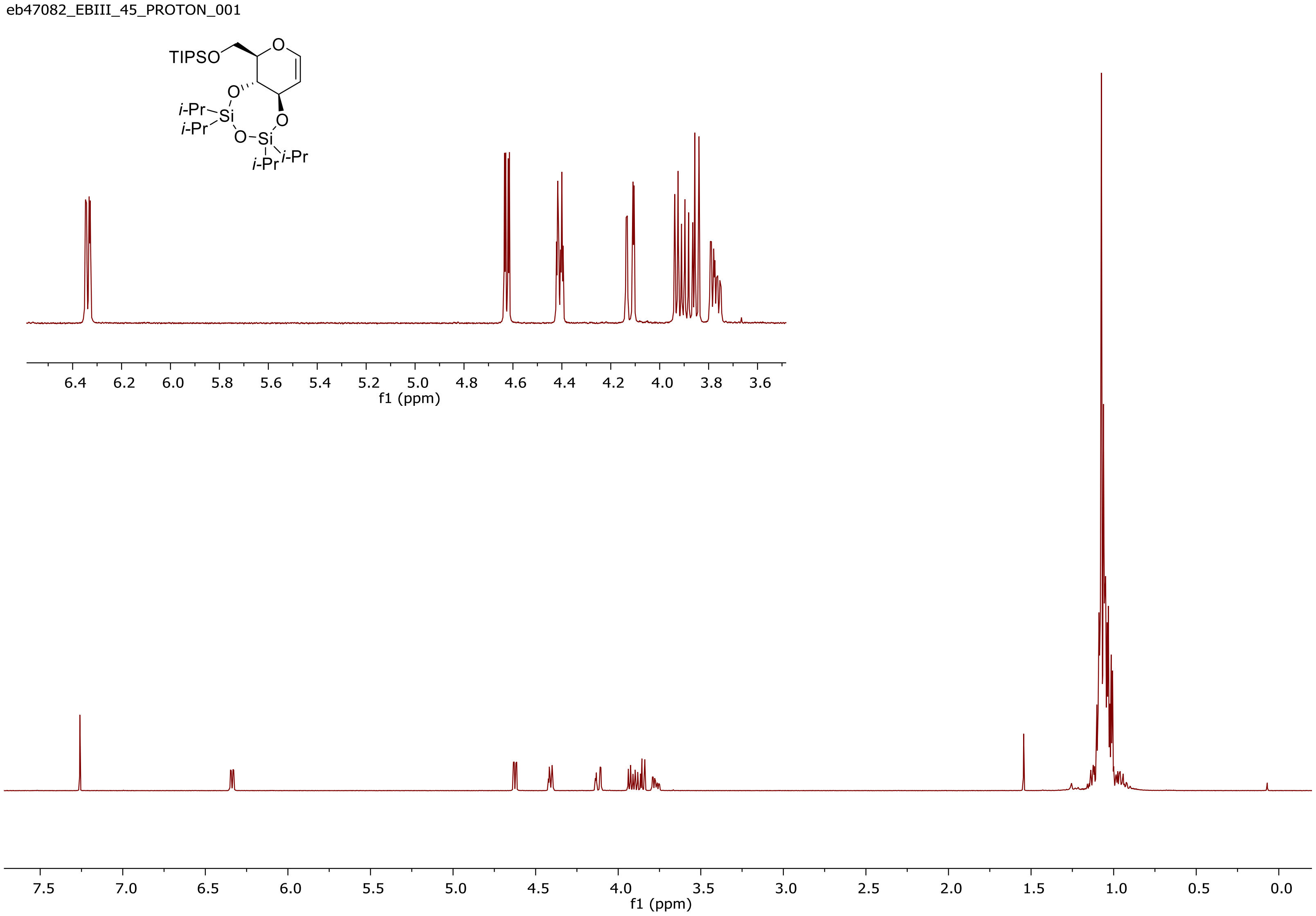

1,2-Dideoxy-3,4-*O*-(1,1,3,3-tetraisopropyldisiloxane-1,3-diyl)-6-*O*-triisopropylsilyl-D-arabino-1-hexenopyranose (5i) ^{13}C NMR (101 MHz; CDCl$_3$)

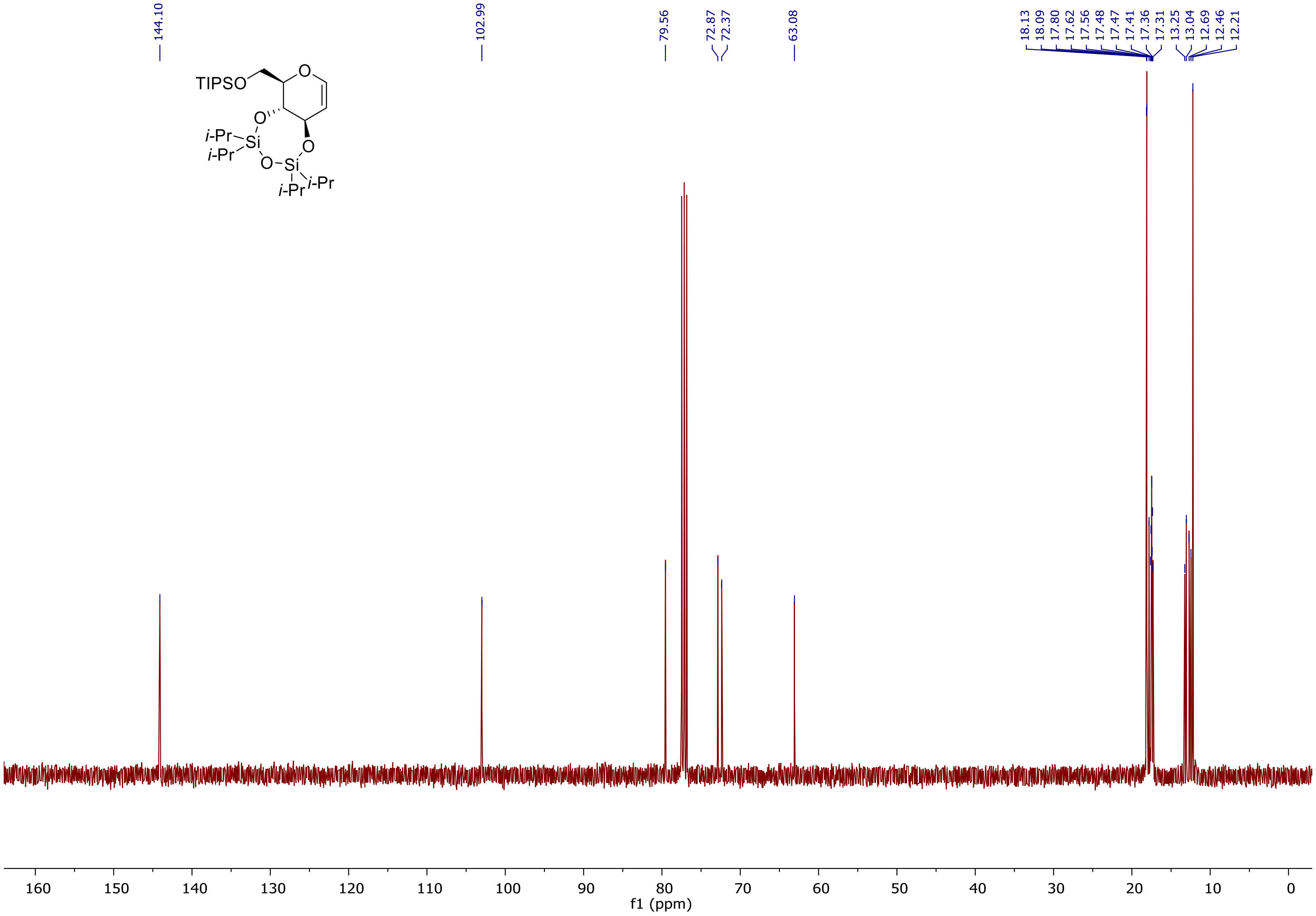

1,2-Dideoxy-3,4-*O*-(1,1,3,3-tetramethyldisiloxane-1,3-diyl)-6-*O*-triisopropylsilyl-D-arabino-1-hexenopyranose (5j) ^{1}H NMR (400 MHz; $CDCl_3$)

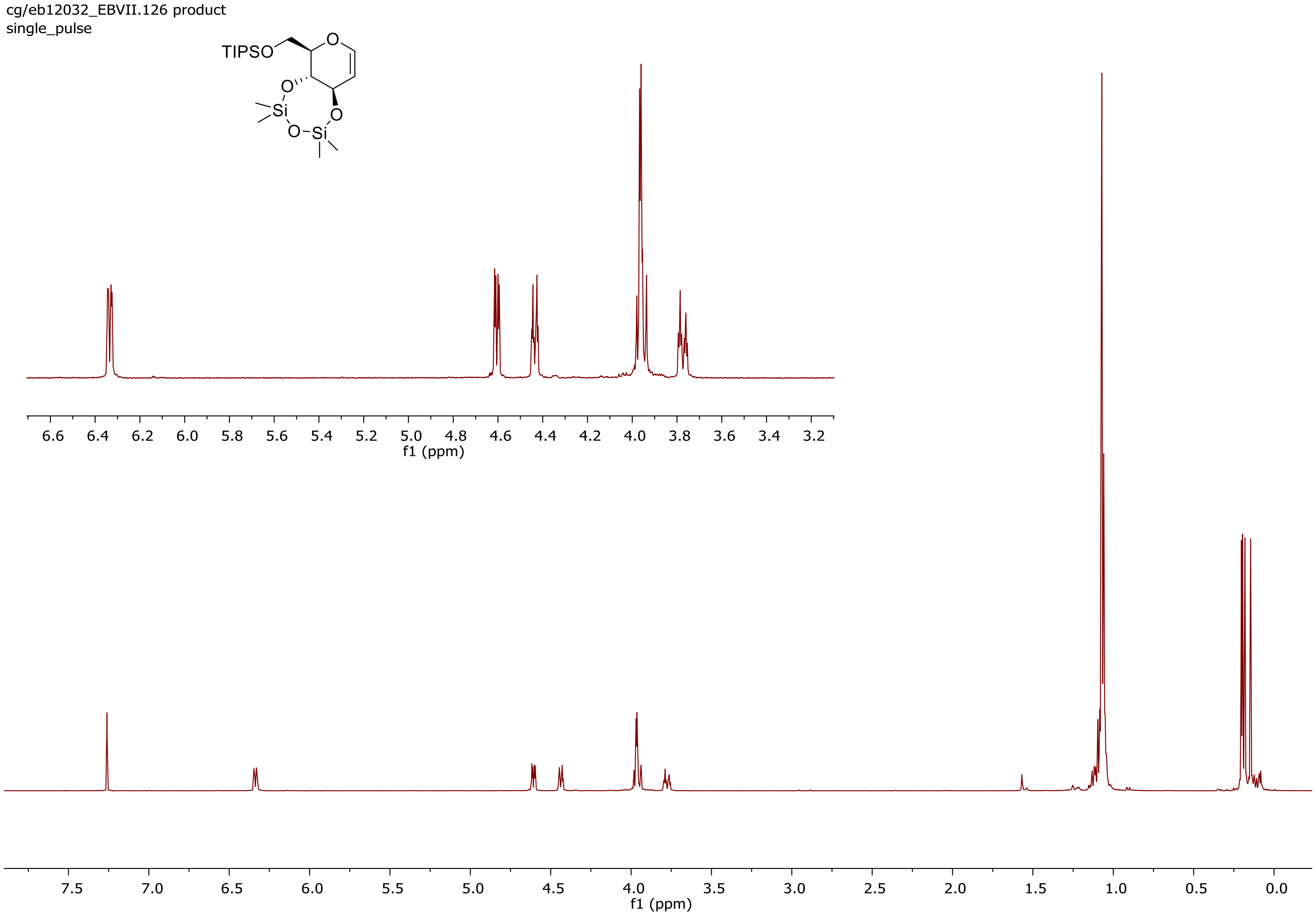

1,2-Dideoxy-3,4-*O*-(1,1,3,3-tetramethyldisiloxane-1,3-diyl)-6-*O*-triisopropylsilyl-D-arabino-1-hexenopyranose (5j) ^{13}C NMR (101 MHz; CDCl$_3$)

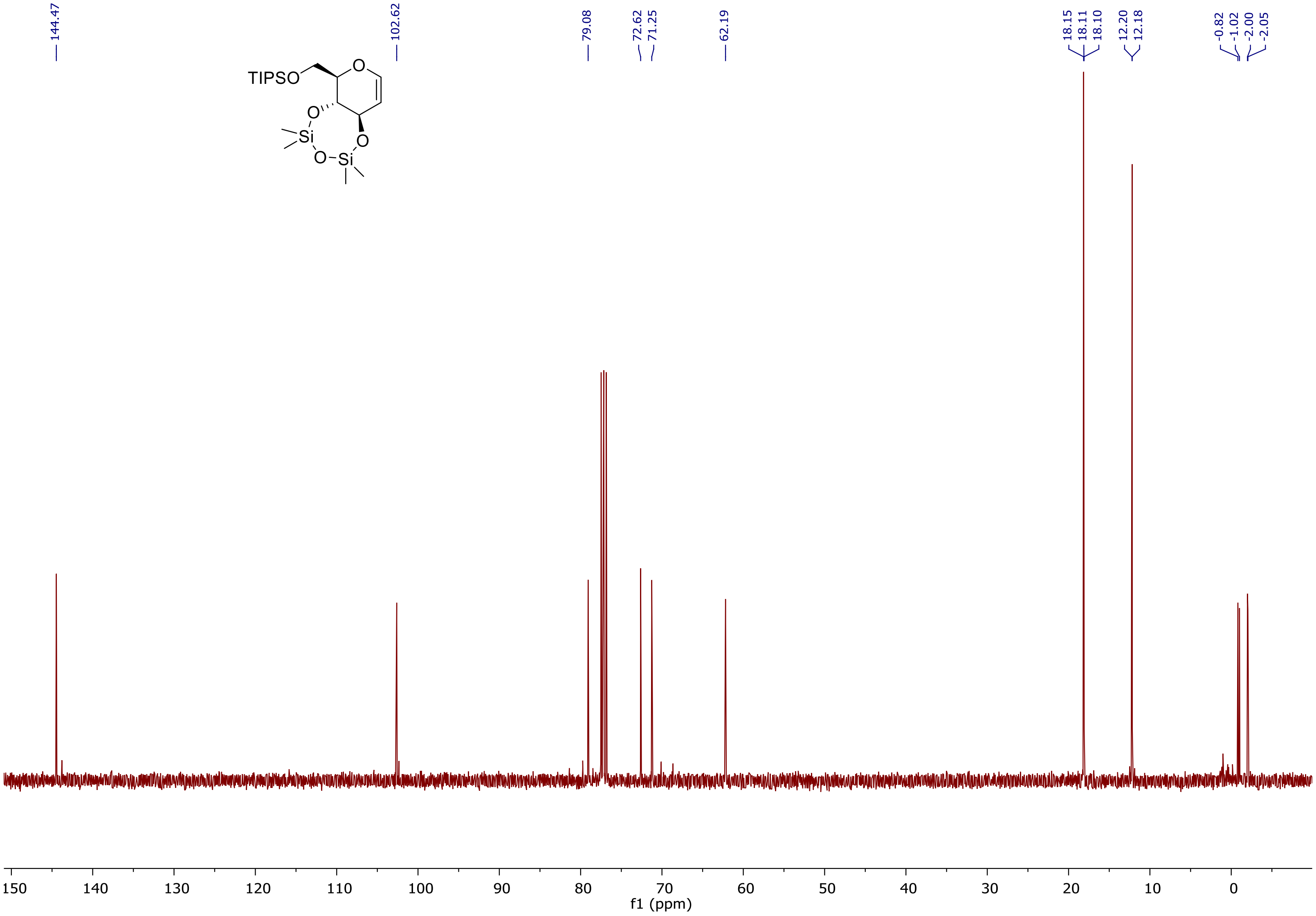

3,4-*O*-(1,1,3,3-Tetraisopropyldisiloxane-1,3-diyl)-1,2,6-trideoxy-L-arabino-1-hexenopyranose (9) ^{1}H NMR (400 MHz; CDCl$_3$)

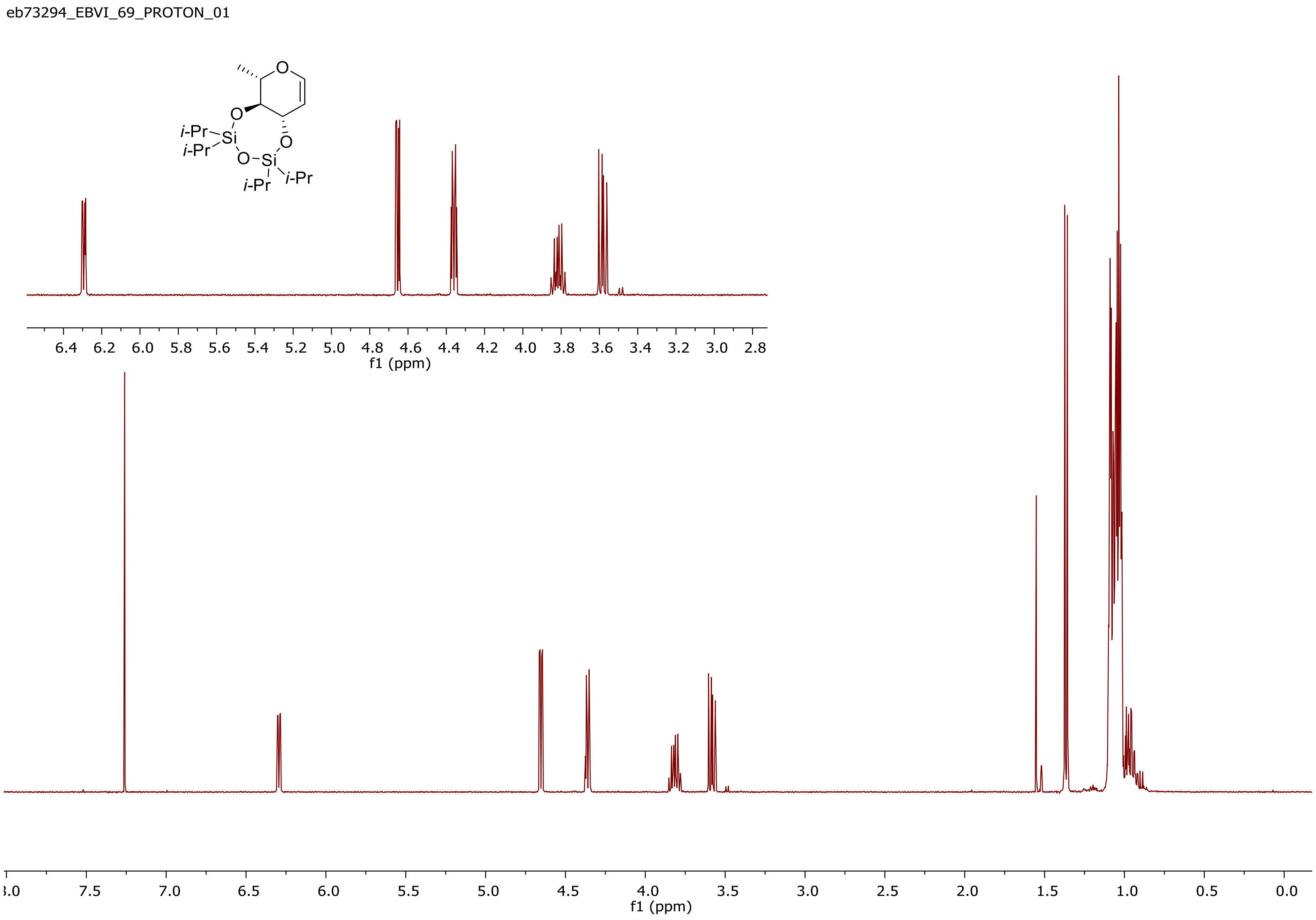

3,4-*O*-(1,1,3,3-Tetraisopropyldisiloxane-1,3-diyl)-1,2,6-trideoxy-L-arabino-1-hexenopyranose (9) ^{13}CNMR (101 MHz; CDCl$_3$)

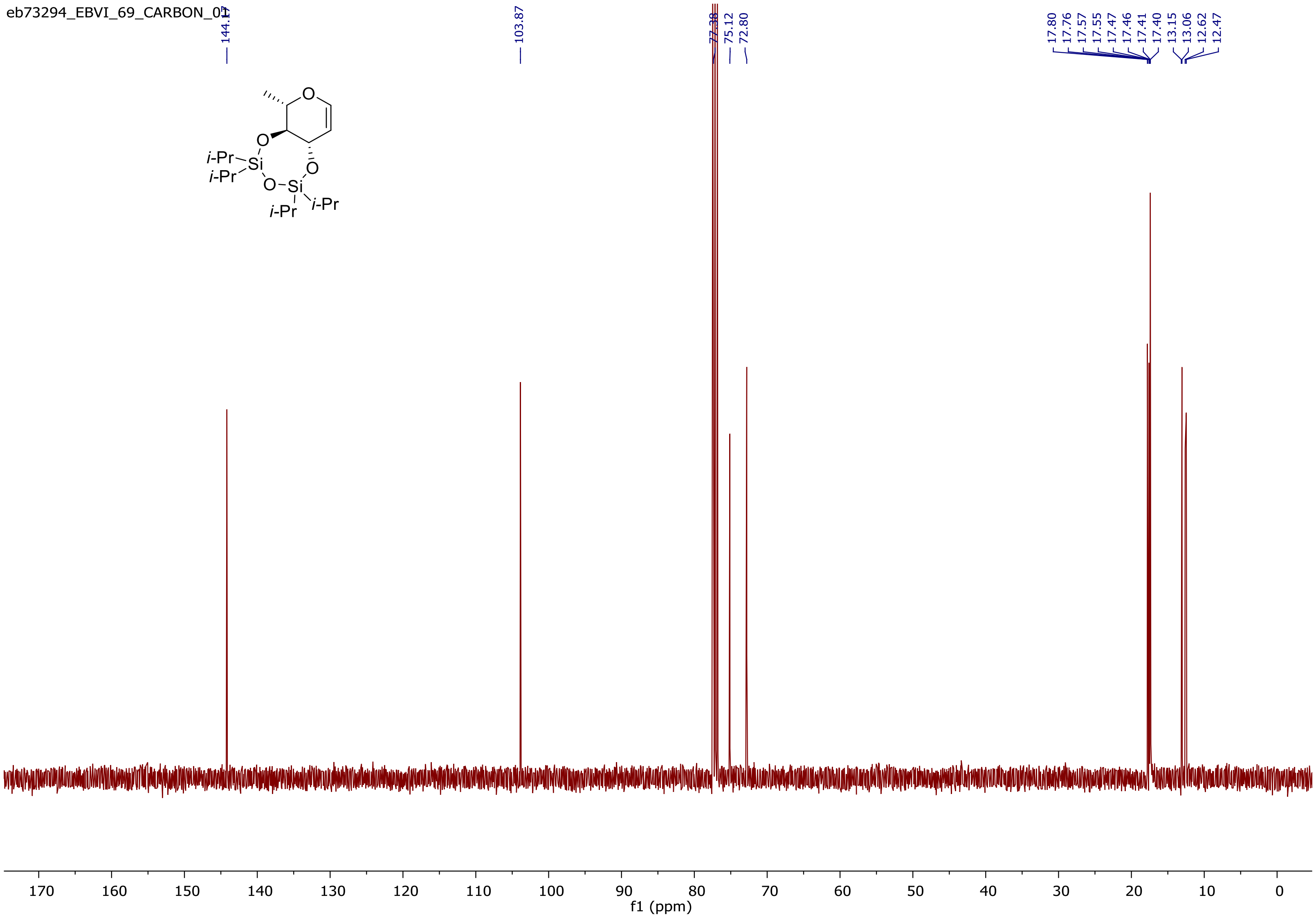

1,2-Dideoxy-[2-^{2}H]-3,4,6-tri-*O*-acetyl-D-arabino-1-hexenopyranose (S15) ^{1}H NMR (500 MHz; $CDCl_3$)

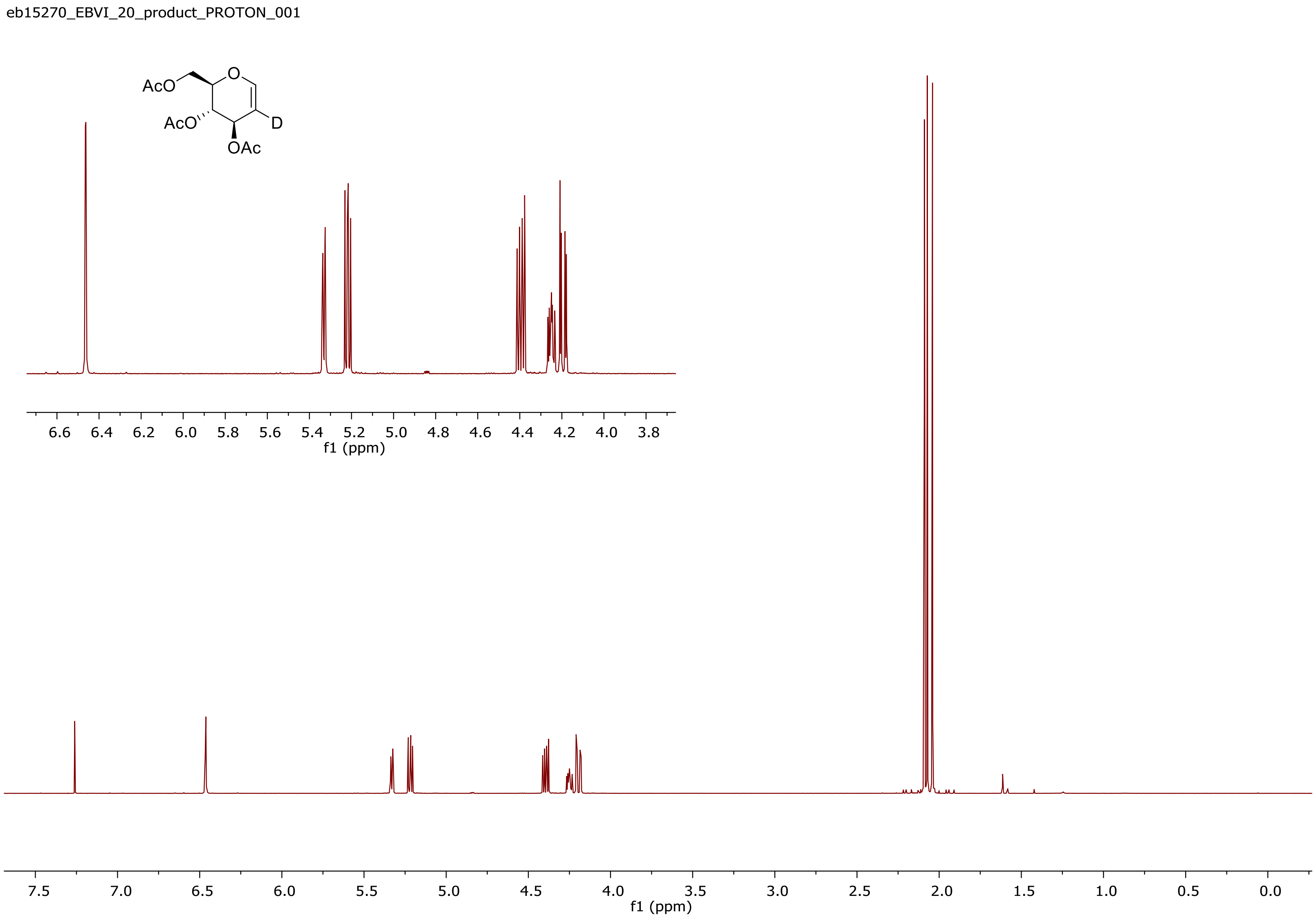

1,2-Dideoxy-[2-²H]-3,4,6-tri-*O*-acetyl-D-arabino-1-hexenopyranose (S15) ^{13}C NMR (126 MHz; CDCl$_3$)

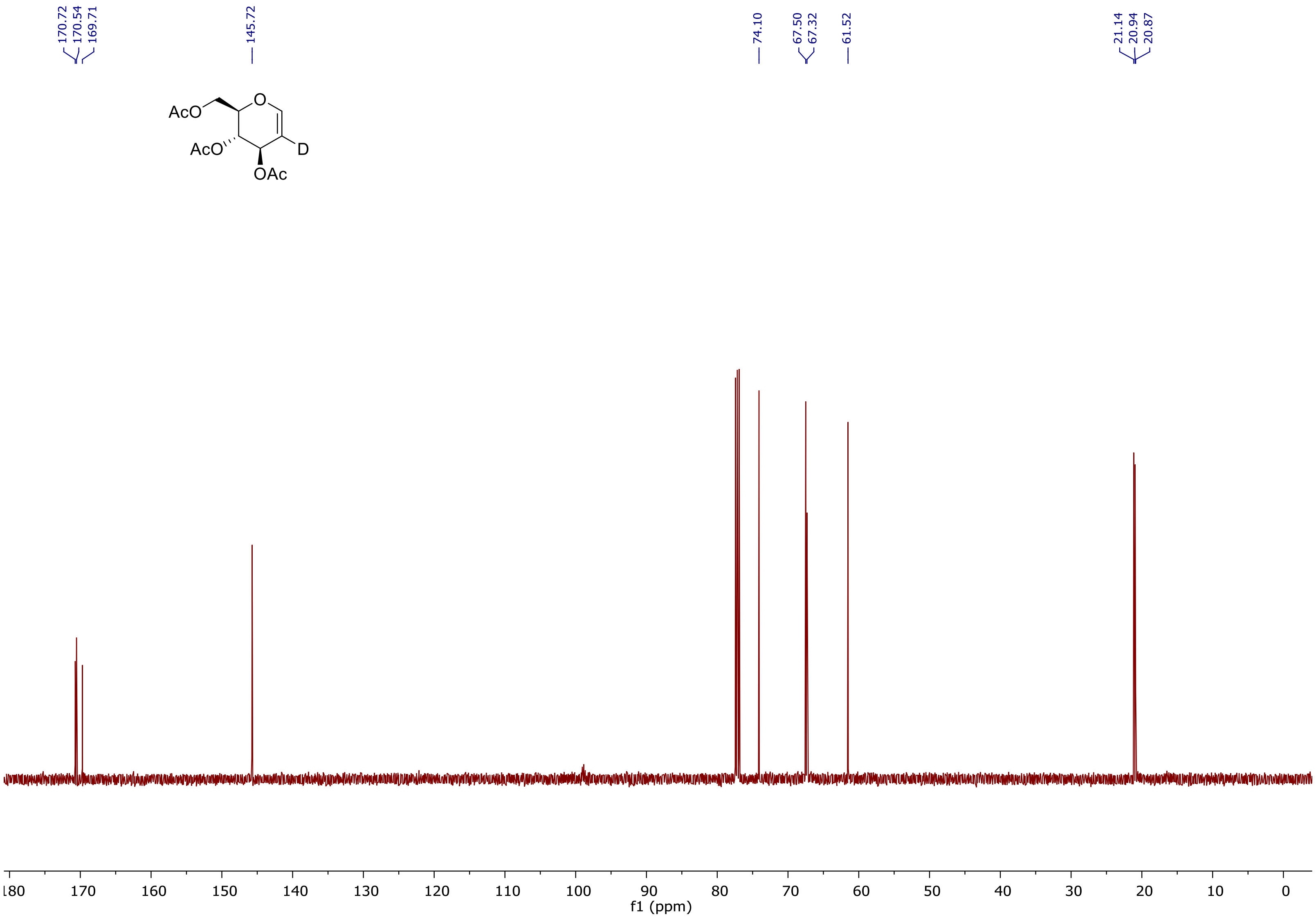

[2-^{2}H]-1,2-Dideoxy-3,4-*O*-(1,1,3,3-tetraisopropyldisiloxane-1,3-diyl)-6-*O*-triisopropylsilyl-D-arabino-1-hexenopyranose (13) ^{1}H NMR (400 MHz; CDCl$_3$)

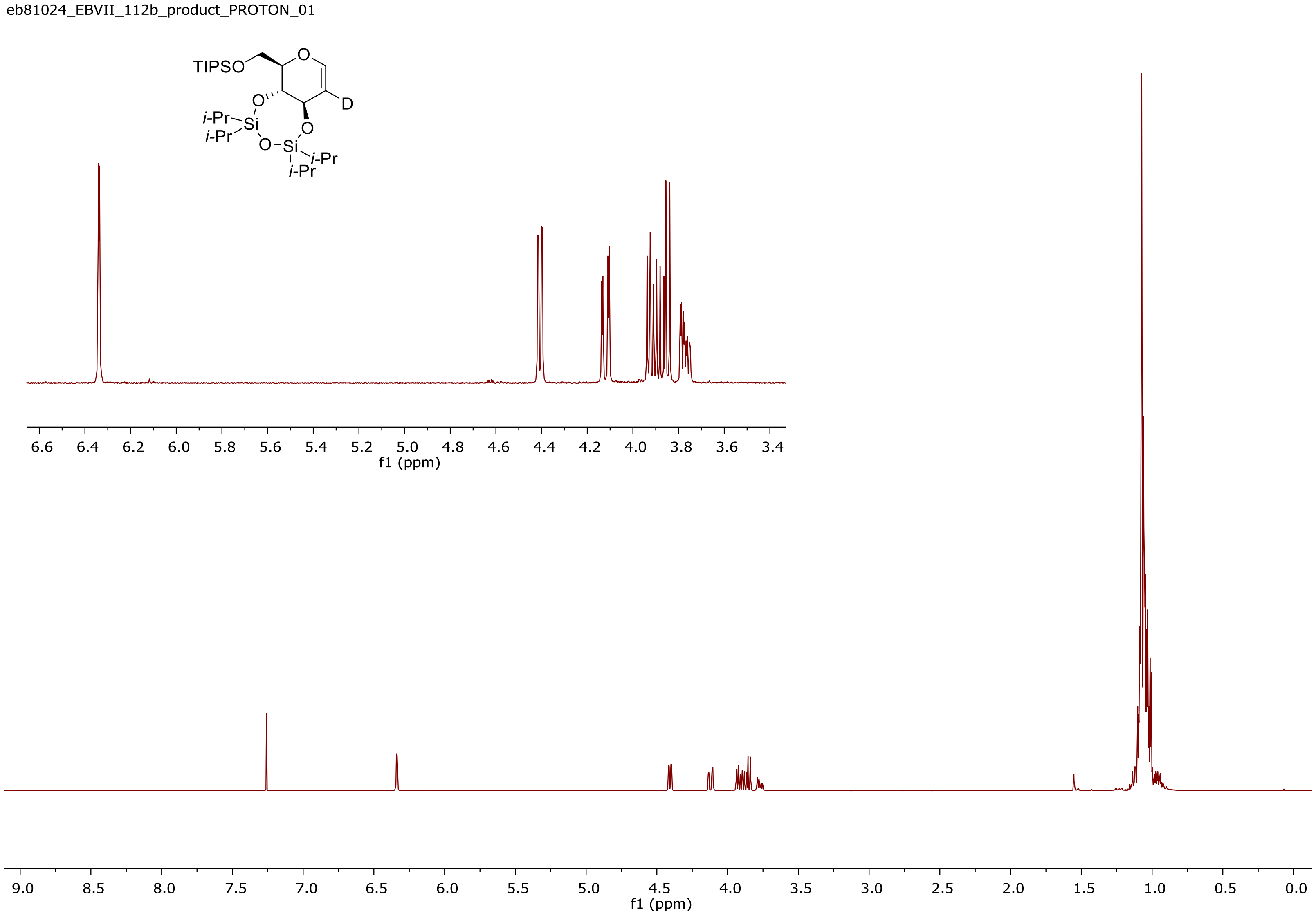

[2-^{2}H]-1,2-Dideoxy-3,4-*O*-(1,1,3,3-tetraisopropyldisiloxane-1,3-diyl)-6-*O*-triisopropylsilyl-D-arabino-1-hexenopyranose (13) ^{13}C NMR (101 MHz; CDCl$_3$)

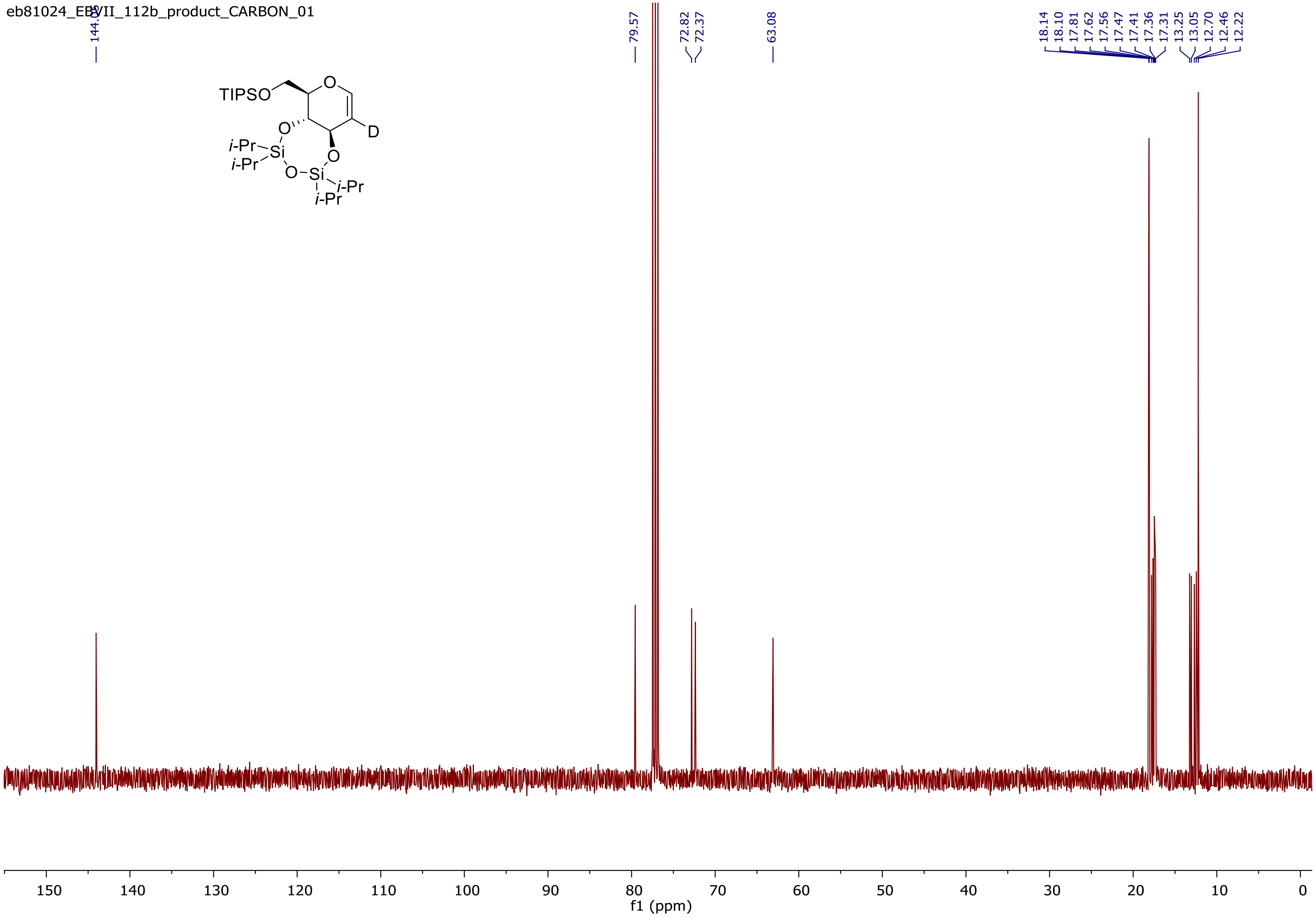

Methyl 2,3,4-tri-*O*-benzyl-6-*O*-(3,4,6-tri-*O*-benzyl-2-deoxy-α/β-D-erythro-hexapyranosyl)-α-D-glucopyranoside (6a) and Methyl 2,3,4-tri-*O*-benzyl-6-*O*-(4,6-di-*O*-benzyl-2,3-dideoxy-α/β-D-erythro-hex-2-enopyranosyl)-α-D-glucopyranoside (7a) ^{1}H NMR (400 MHz; $CDCl_3$)

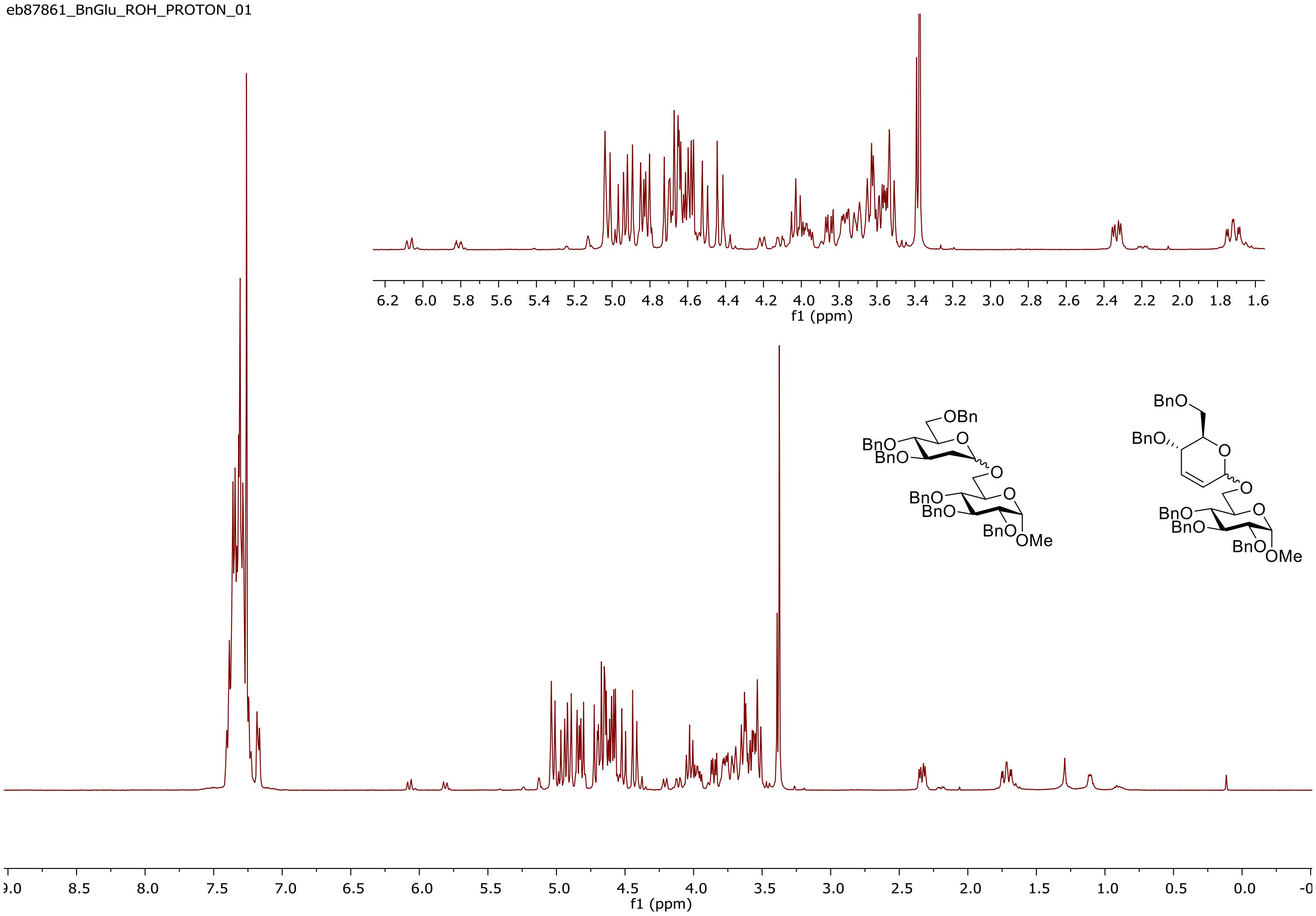

Methyl 2,3,4-tri-*O*-benzyl-6-*O*-(3,4,6-tri-*O*-benzyl-2-deoxy-α/β-D-erythro-hexapyranosyl)-α-D-glucopyranoside (6a) and Methyl 2,3,4-tri-*O*-benzyl-6-*O*-(4,6-di-*O*-benzyl-2,3-dideoxy-α/β-D-erythro-hex-2-enopyranosyl)-α-D-glucopyranoside (7a) ^{13}C NMR (101 MHz; $CDCl_3$)

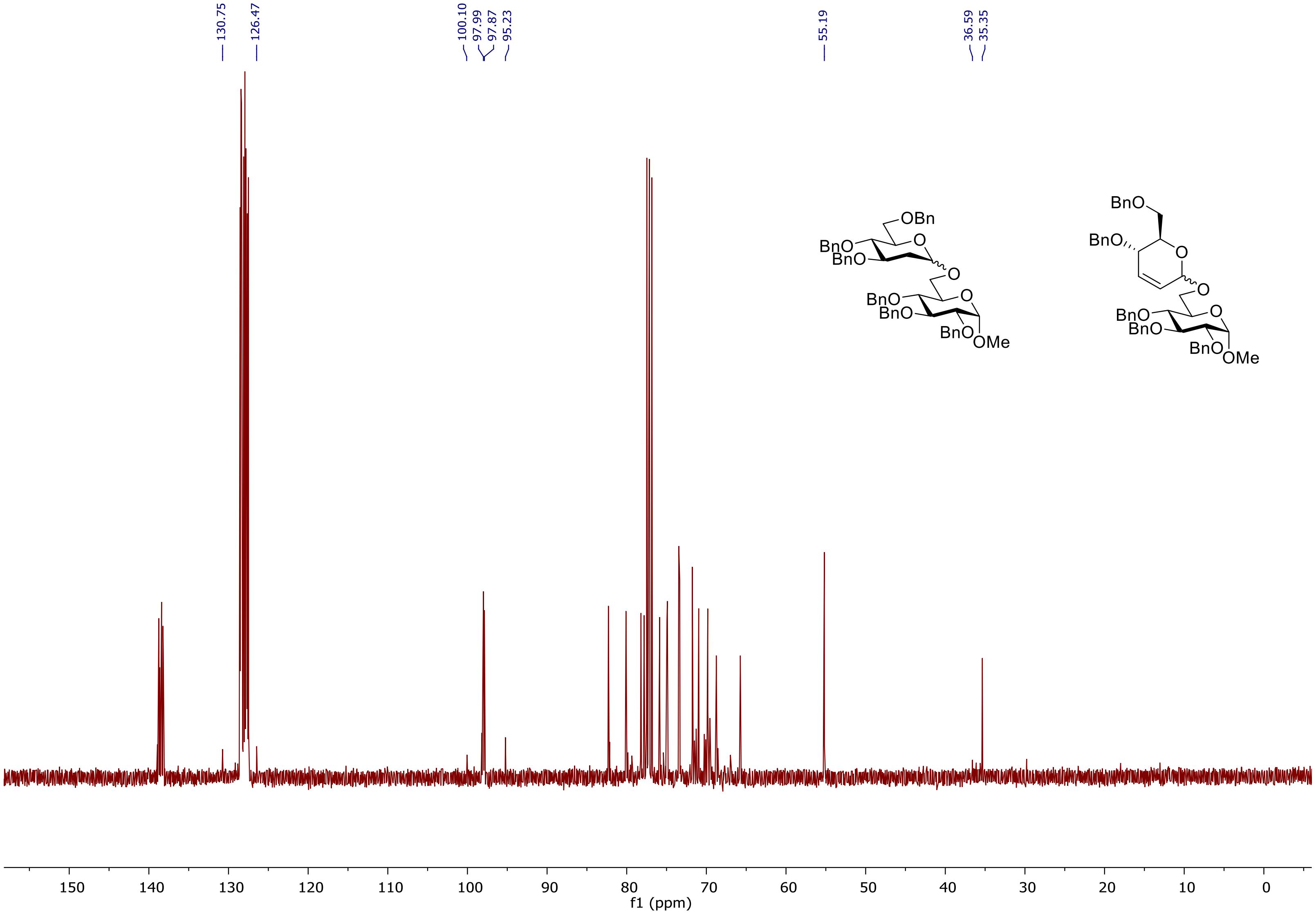

Methyl 2,3,4-tri-*O*-benzyl-6-*O*-(3,4,6-tri-*O*-*tert*-butyldimethylsilyl-2-deoxy-α/β-D-erythro-hexapyranosyl)-α-D-glucopyranoside (6b) ^{1}H NMR (400 MHz; $CDCl_3$)

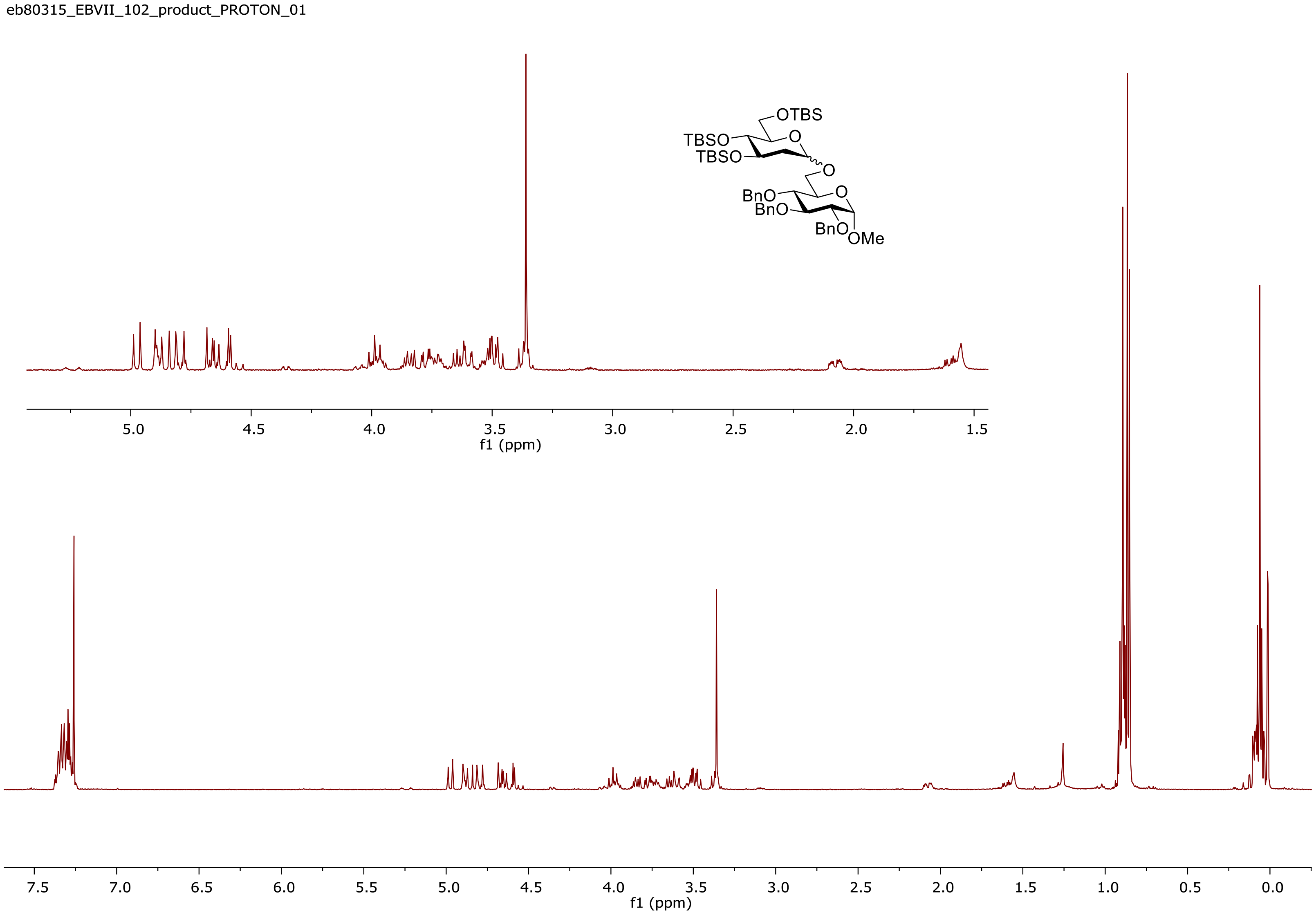

Methyl 2,3,4-tri-*O*-benzyl-6-*O*-(3,4,6-tri-*O*-*tert*-butyldimethylsilyl-2-deoxy-α/β-D-erythro-hexapyranosyl)-α-D-glucopyranoside (6b) ^{13}C NMR (400 MHz; $CDCl_3$)

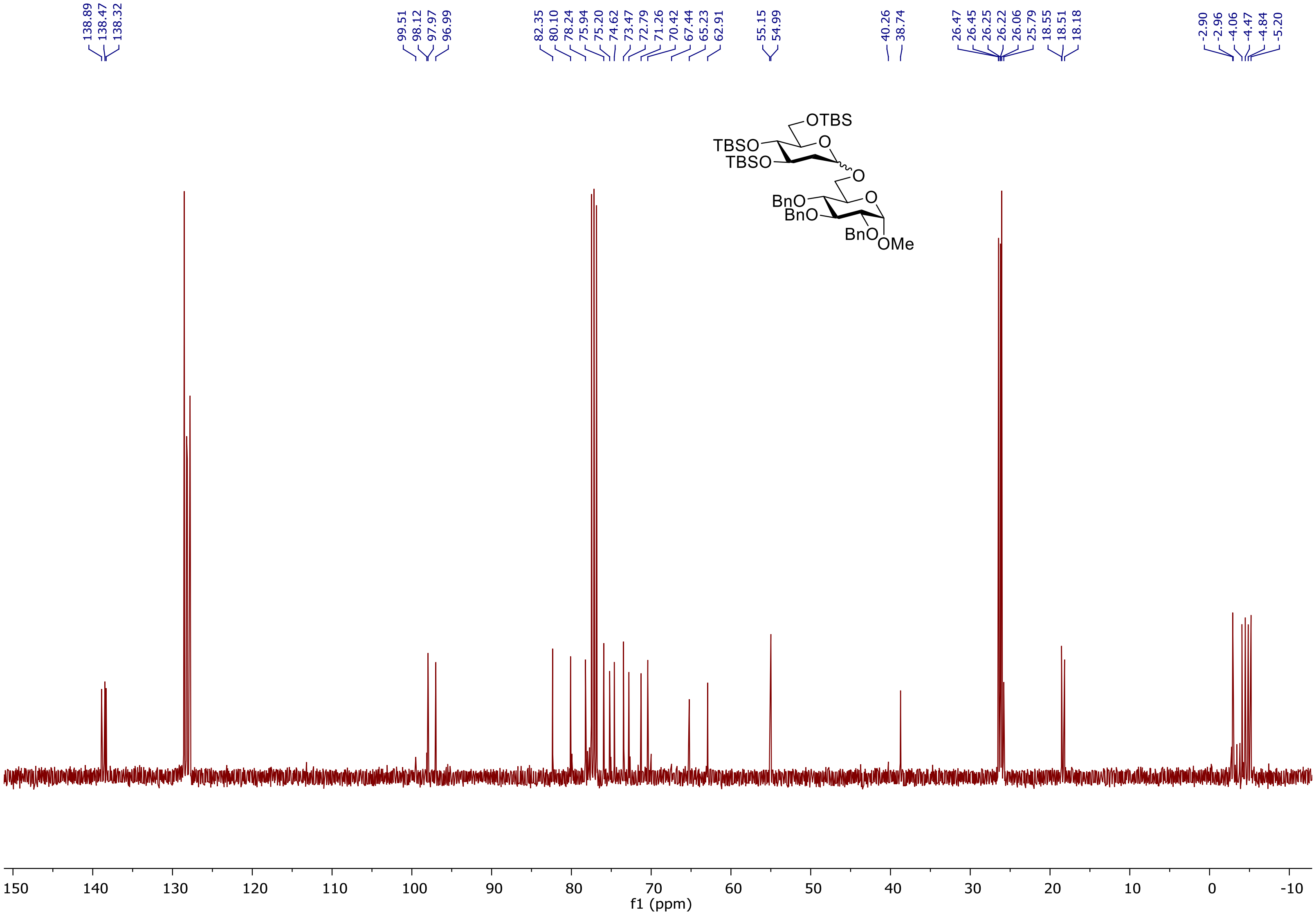

Methyl 2,3,4-tri-*O*-benzyl-6-*O*-(4,6-di-*O*-*tert*-butyldimethylsilyl-2,3-dideoxy-α/β-D-erythro-hex-2-enopyranosyl)-α-D-glucopyranoside (7b) ^{1}H NMR (400 MHz; $CDCl_3$)

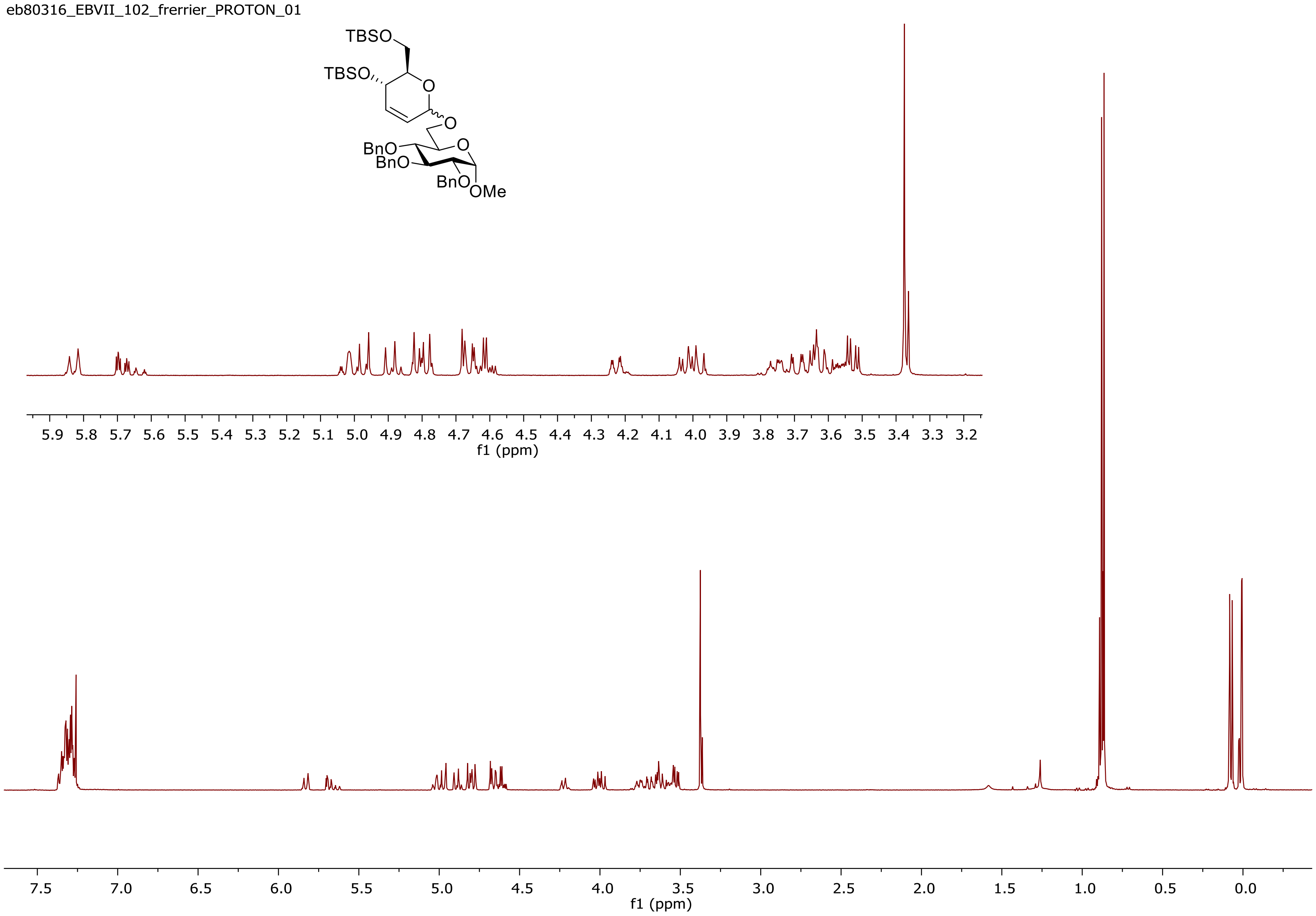

Methyl 2,3,4-tri-*O*-benzyl-6-*O*-(4,6-di-*O*-*tert*-butyldimethylsilyl-2,3-dideoxy-α/β-D-erythro-hex-2-enopyranosyl)-α-D-glucopyranoside (7b) ^{13}C NMR (400 MHz; CDCl$_3$)

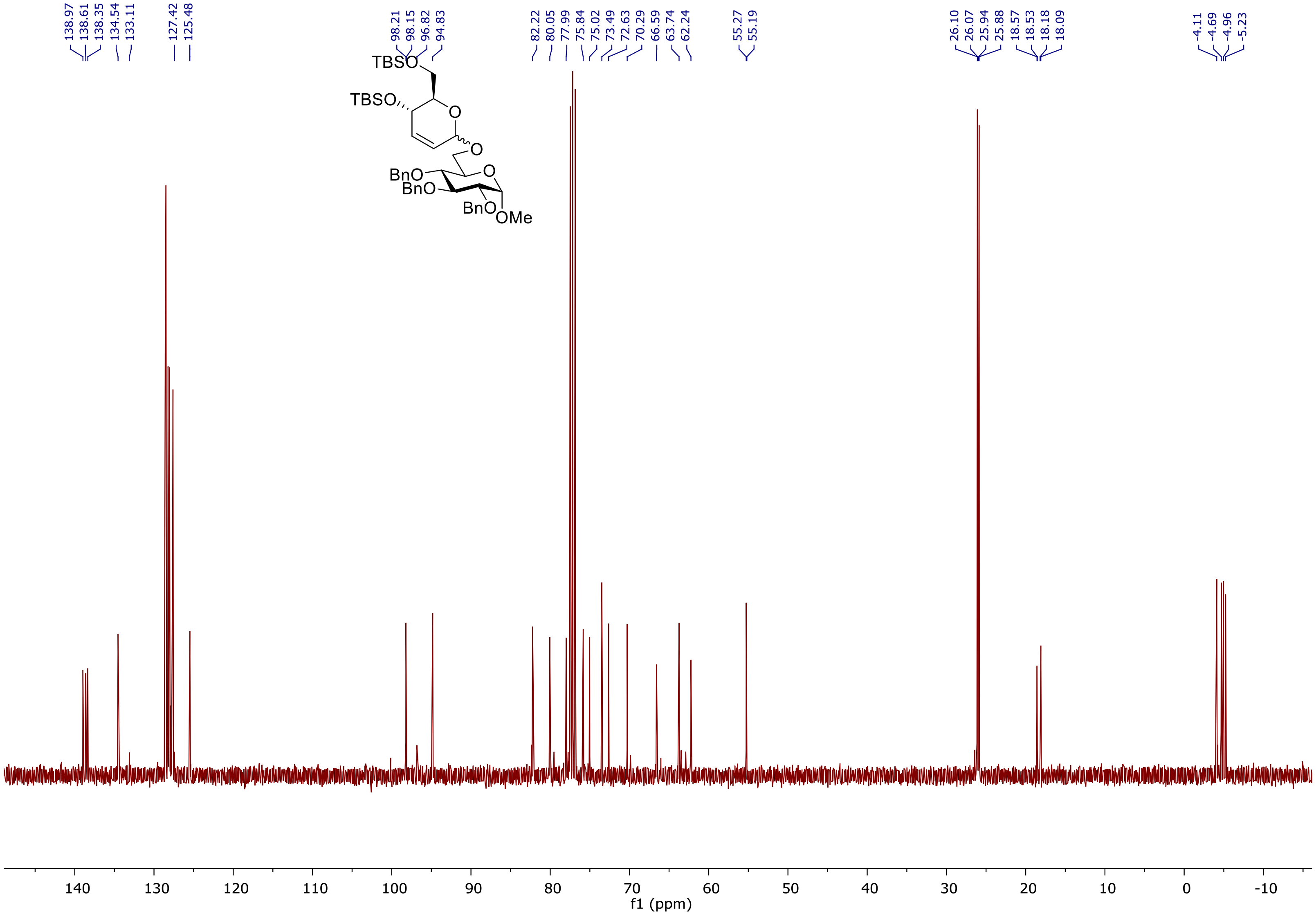

Methyl 2,3,4-tri-*O*-benzyl-6-*O*-(4-*O*-benzyl-2-deoxy-3,6-di-*O*-*tert*-butyldimethylsilyl-α/β-D-erythro-hexapyranosyl)-α-D-glucopyranoside (6c) ^{1}H NMR (500 MHz; $CDCl_3$)

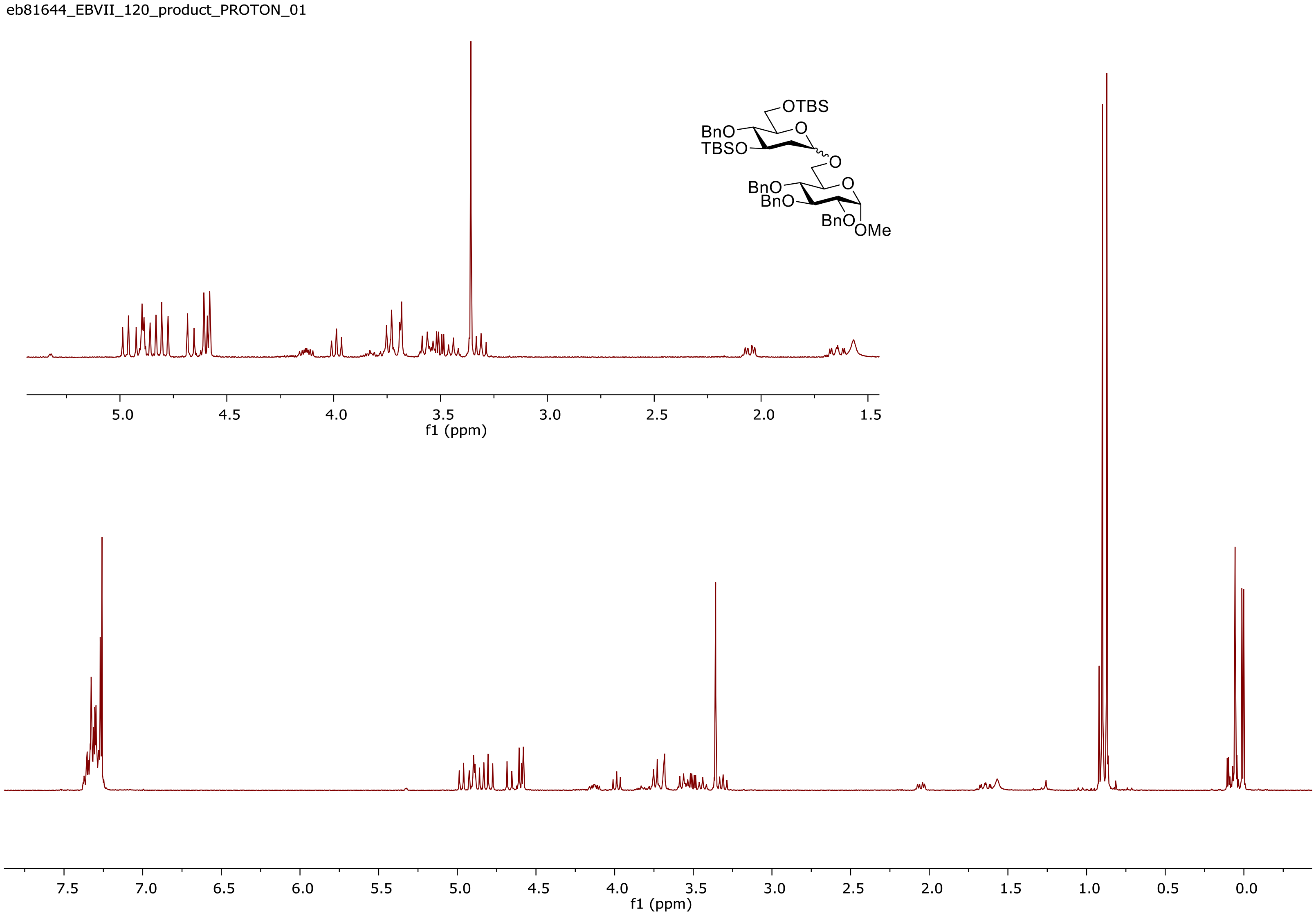

Methyl 2,3,4-tri-*O*-benzyl-6-*O*-(4-*O*-benzyl-2-deoxy-3,6-di-*O*-*tert*-butyldimethylsilyl-α/β-D-erythro-hexapyranosyl)-α-D-glucopyranoside (6c) ^{13}C NMR (126 MHz; $CDCl_3$)

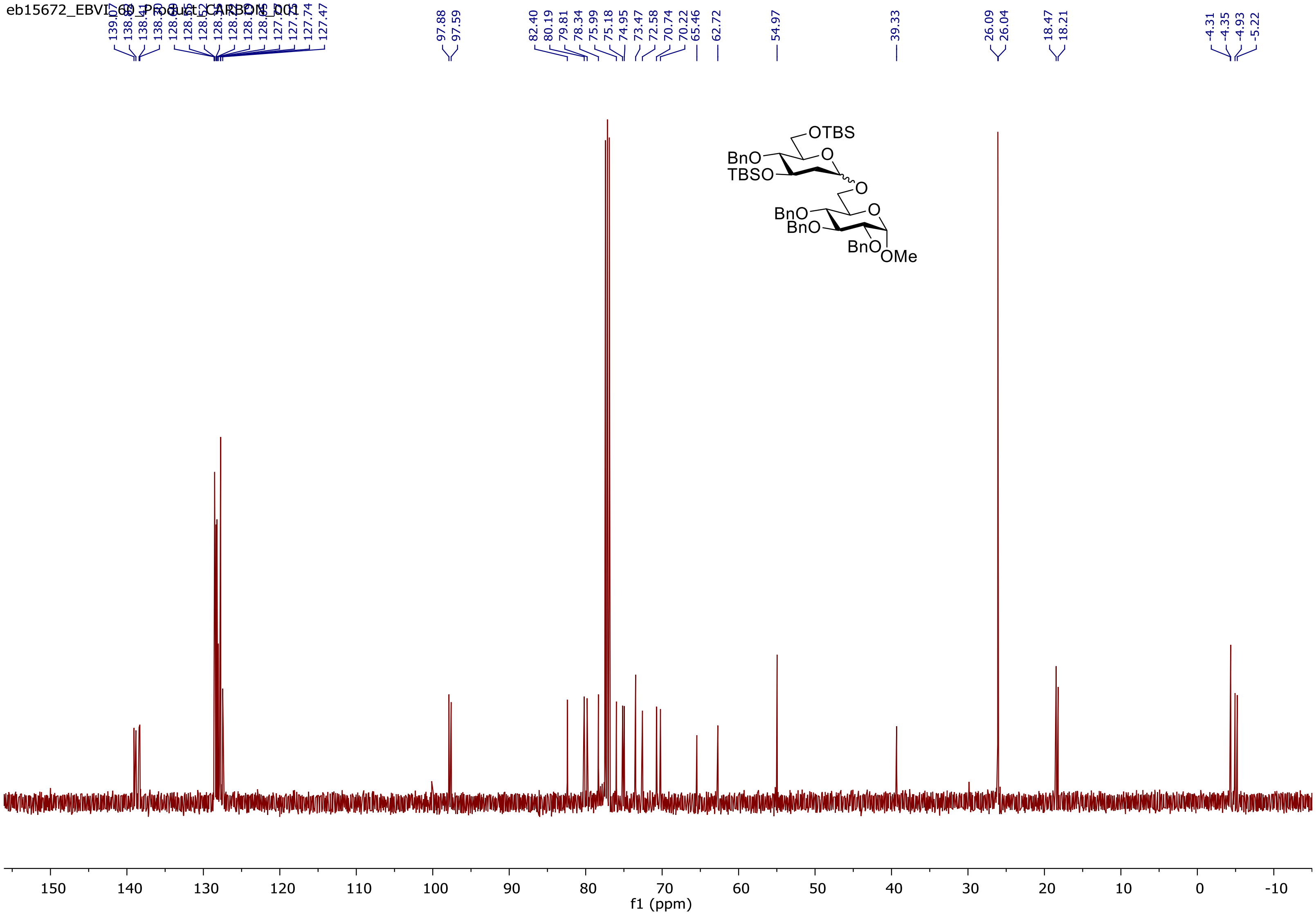

Methyl 2,3,4-tri-*O*-benzyl-6-*O*-(4-*O*-benzyl-6-*O*-*tert*-butyldimethylsilyl-2,3-dideoxy-α/β-D-erythro-hex-2-enopyranosyl)-α-D-glucopyranoside (7c) ^{1}H NMR (500 MHz; $CDCl_3$)

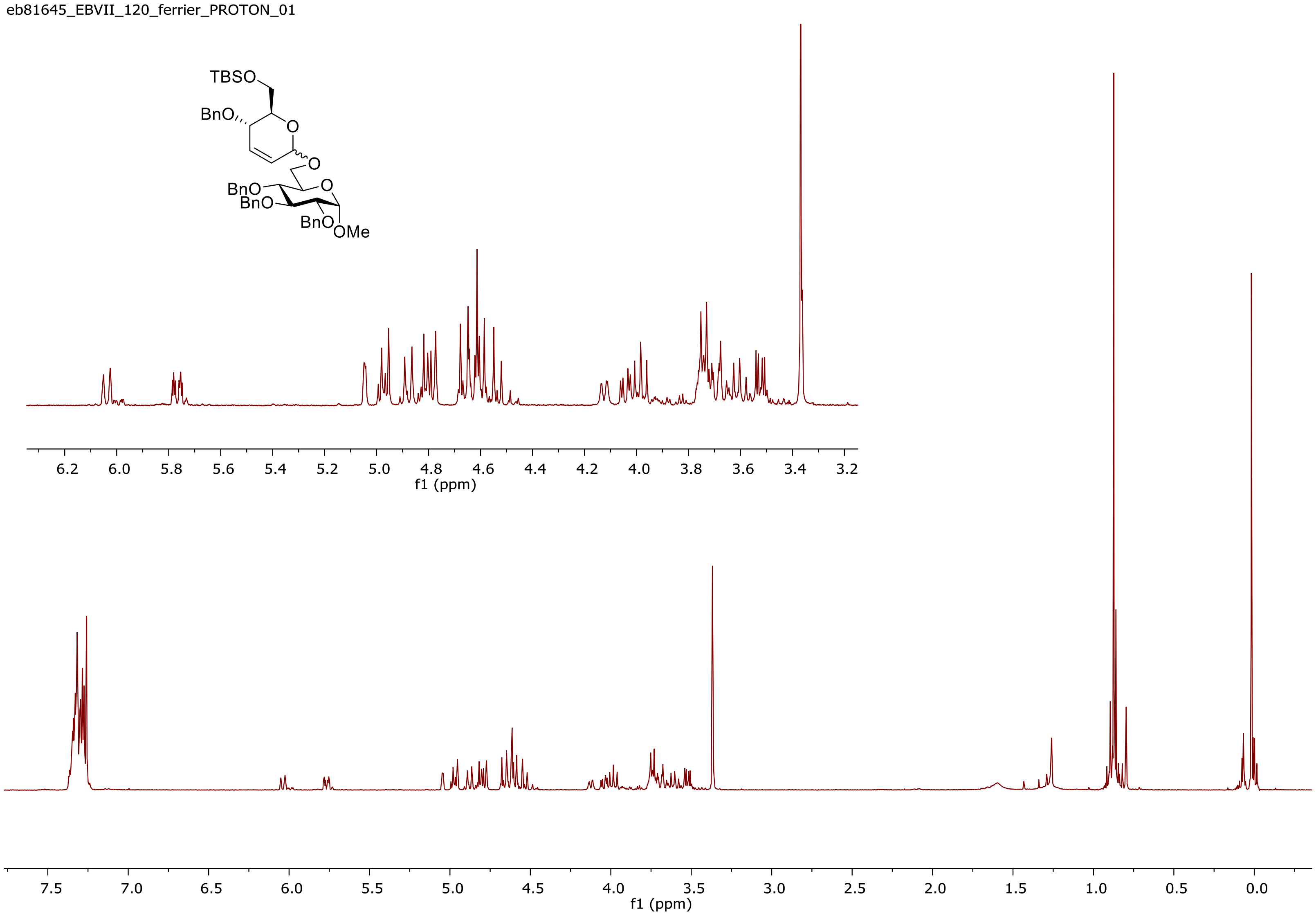

Methyl 2,3,4-tri-*O*-benzyl-6-*O*-(4-*O*-benzyl-6-*O*-*tert*-butyldimethylsilyl-2,3-dideoxy-α/β-D-erythro-hex-2-enopyranosyl)-α-D-glucopyranoside (7c) ^{13}C NMR (126 MHz; $CDCl_3$)

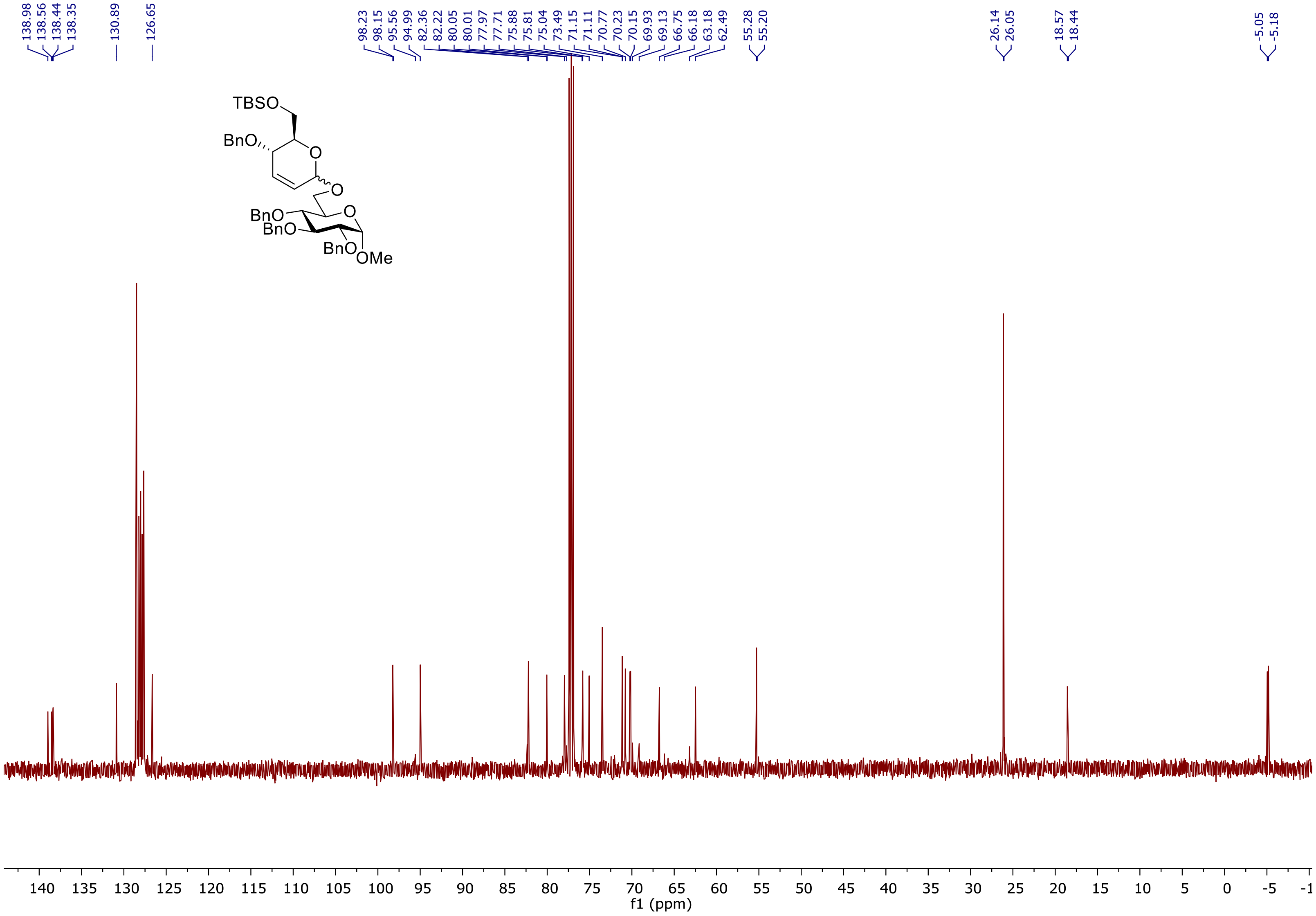

Methyl 2,3,4-tri-*O*-benzyl-6-*O*-(4-*O*-acetyl-2-deoxy-3,6-di-*O*-*tert*-butyldimethylsilyl-α/β-D-erythro-hexapyranosyl)-α-D-glucopyranoside (6d) ^{1}H NMR (500 MHz; $CDCl_3$)

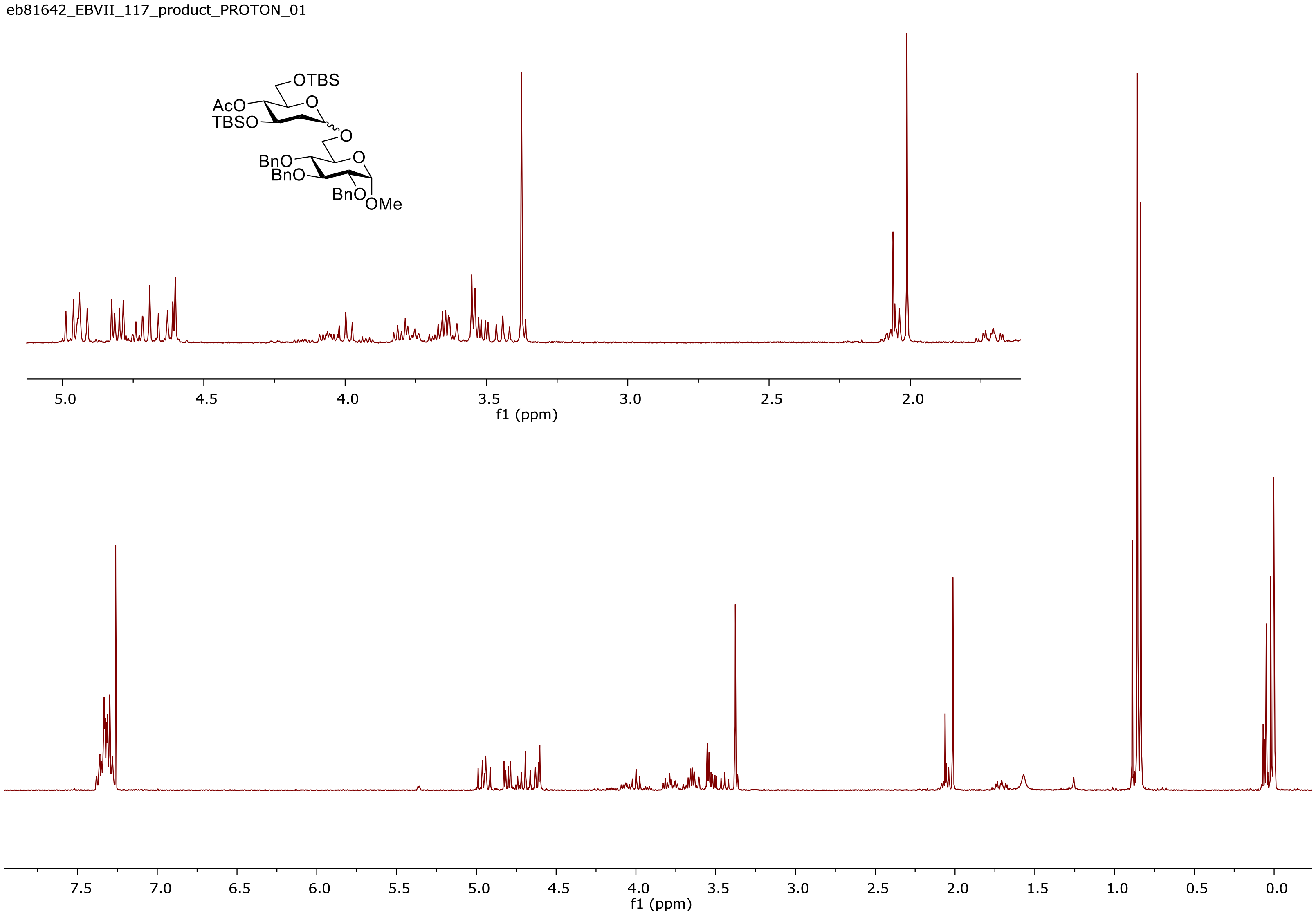

Methyl 2,3,4-tri-*O*-benzyl-6-*O*-(4-*O*-acetyl-2-deoxy-3,6-di-*O*-*tert*-butyldimethylsilyl-α/β-D-erythro-hexapyranosyl)-α-D-glucopyranoside (6d) ^{13}C NMR (126 MHz; $CDCl_3$)

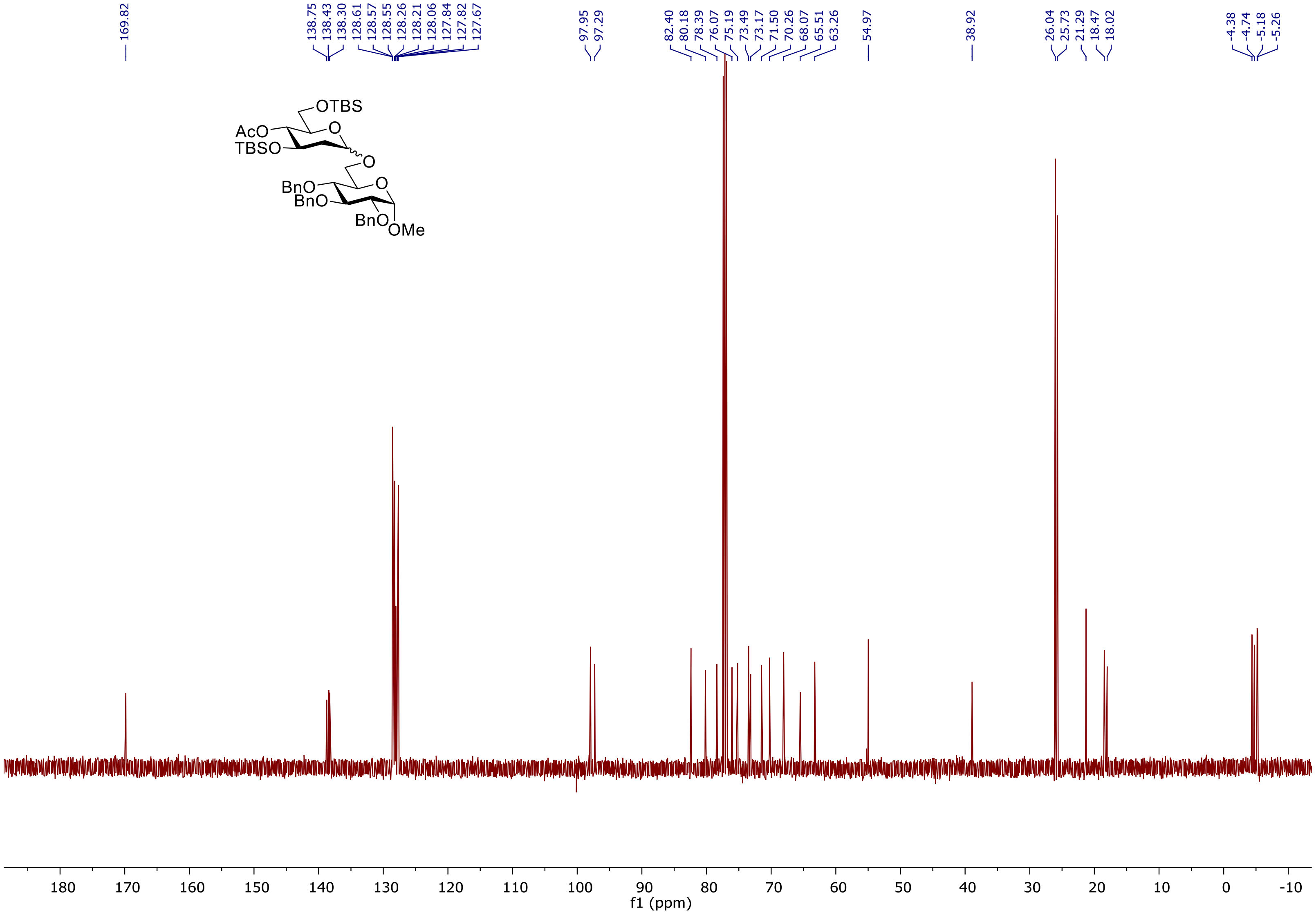

Methyl 2,3,4-tri-*O*-benzyl-6-*O*-(4-*O*-acetyl-6-*O*-*tert*-butyldimethylsilyl-2,3-dideoxy-α/β-D-erythro-hex-2-enopyranosyl)-α-D-glucopyranoside (7d) ^{1}H NMR (500 MHz; $CDCl_3$)

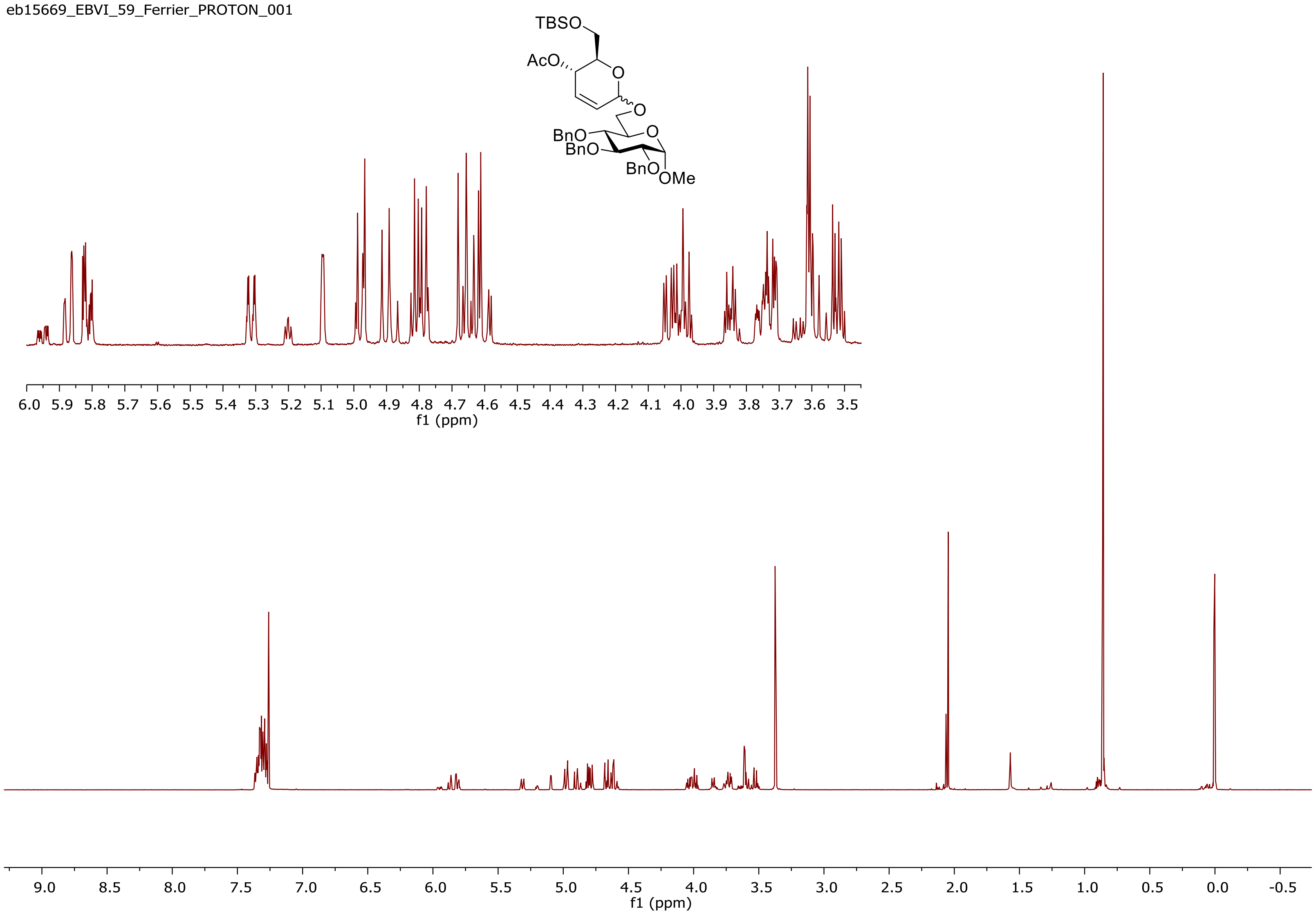

Methyl 2,3,4-tri-*O*-benzyl-6-*O*-(4-*O*-acetyl-6-*O*-*tert*-butyldimethylsilyl-2,3-dideoxy-α/β-D-erythro-hex-2-enopyranosyl)-α-D-glucopyranoside (7d) ^{13}C NMR (126 MHz; CDCl$_3$)

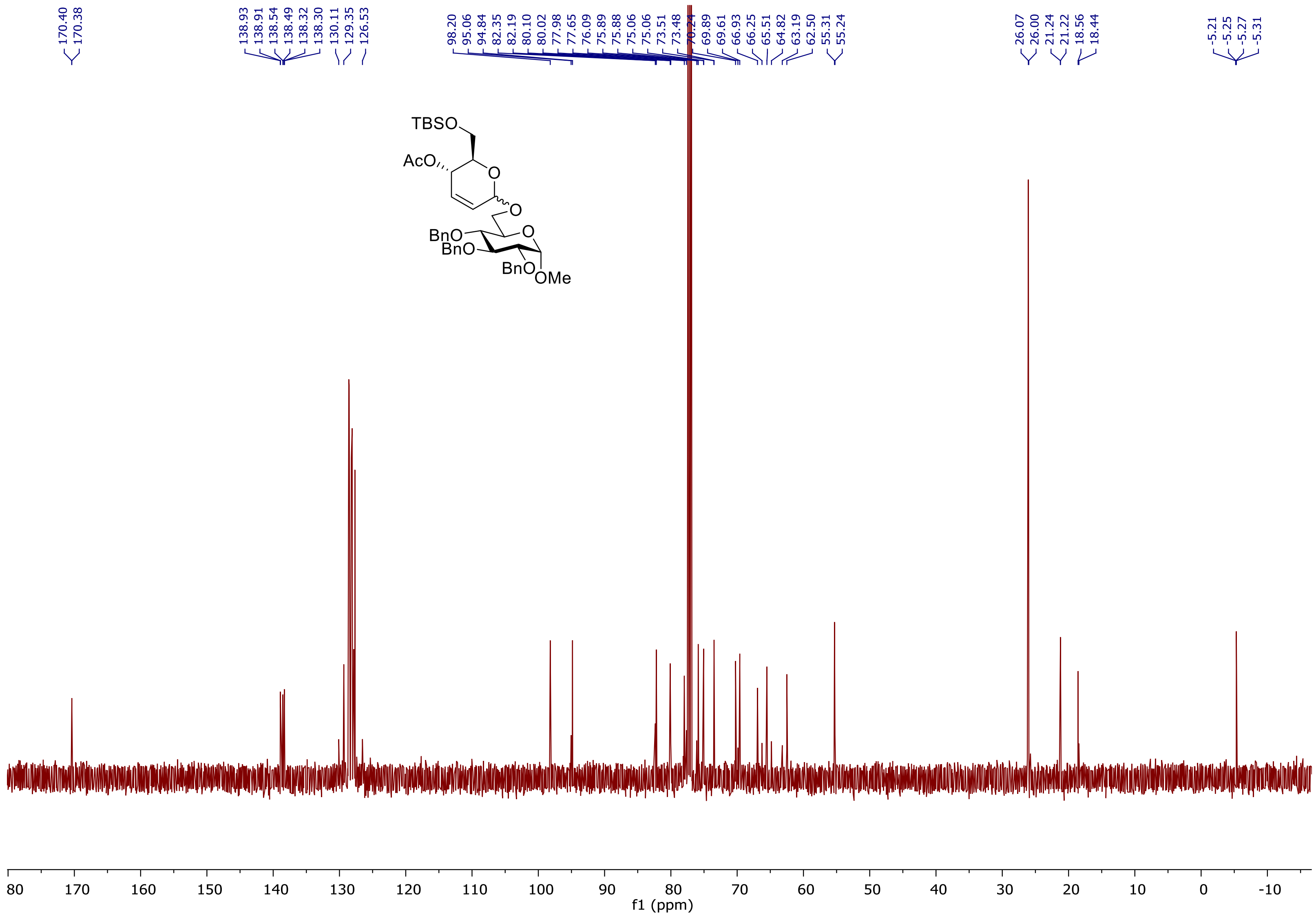

Methyl 2,3,4-tri-*O*-benzyl-6-*O*-(4-*O*-allyl-2-deoxy-3,6-di-*O*-triisopropylsilyl-α/β-D-erythro-hexapyranosyl)-α-D-glucopyranoside (6e) ^{1}H NMR (400 MHz; CDCl$_3$)

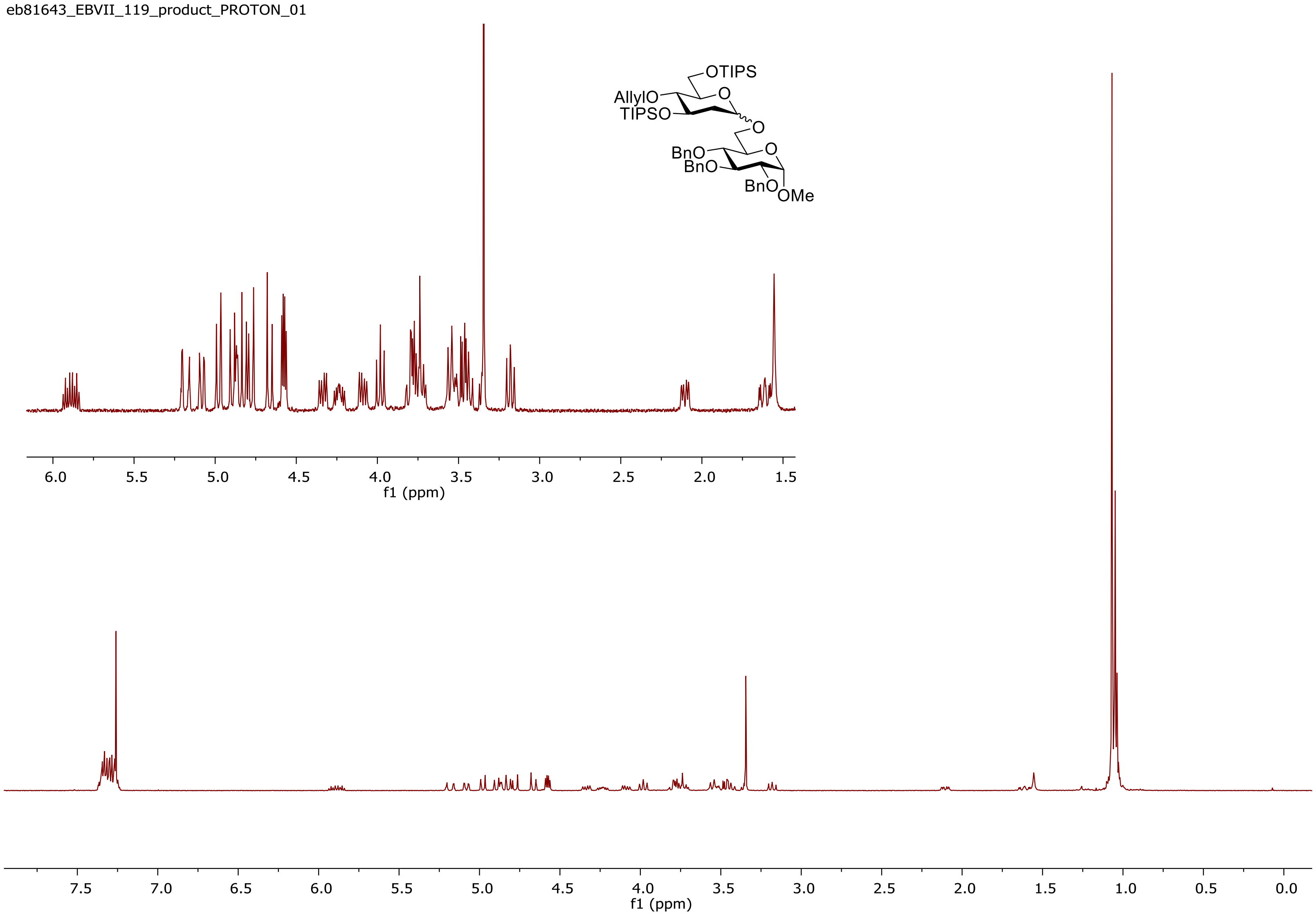

Methyl 2,3,4-tri-*O*-benzyl-6-*O*-(4-*O*-allyl-2-deoxy-3,6-di-*O*-triisopropylsilyl-α/β-D-erythro-hexapyranosyl)-α-D-glucopyranoside (6e) ^{13}C NMR (101 MHz; $CDCl_3$)

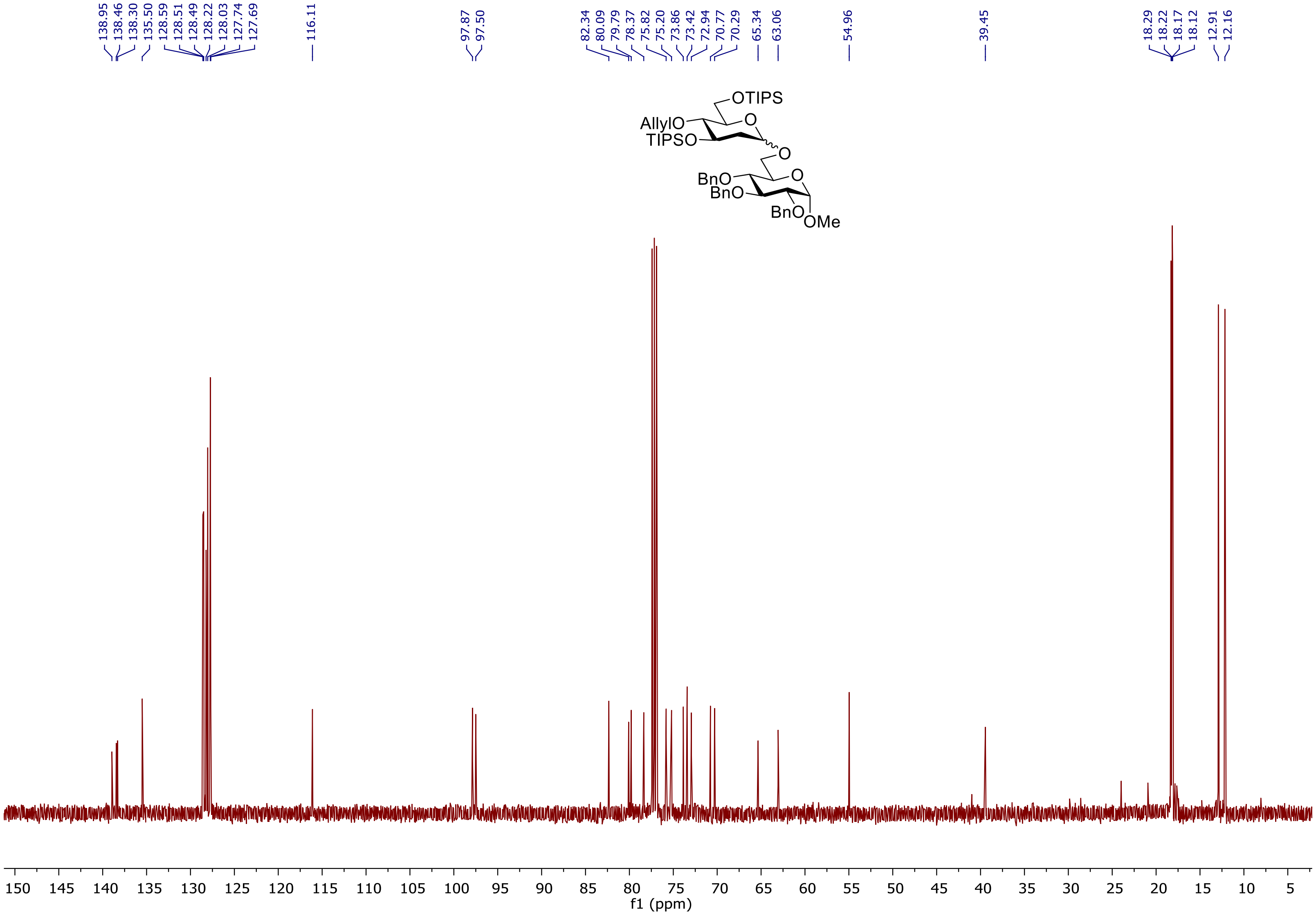

Methyl 2,3,4-tri-*O*-benzyl-6-*O*-(4-*O*-allyl-6-*O*-triisopropylsilyl-2,3-dideoxy-α/β-D-erythro-hex-2-enopyranosyl)-α-D-glucopyranoside (7e)

^{1}H NMR (400 MHz; CDCl$_3$)

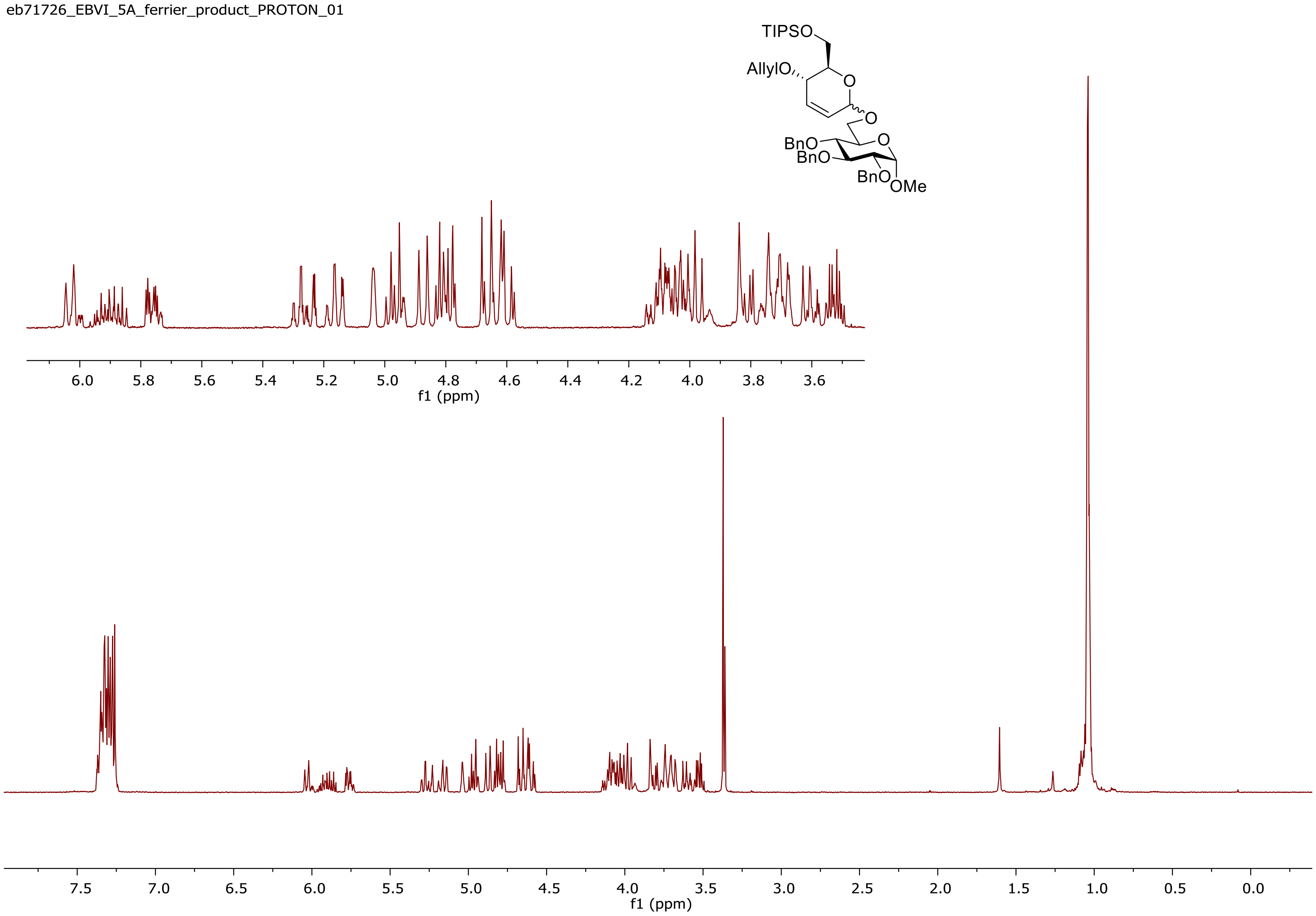

Methyl 2,3,4-tri-*O*-benzyl-6-*O*-(4-*O*-allyl-6-*O*-triisopropylsilyl-2,3-dideoxy-α/β-D-erythro-hex-2-enopyranosyl)-α-D-glucopyranoside (7e)

^{13}C NMR (101 MHz; $CDCl_3$)

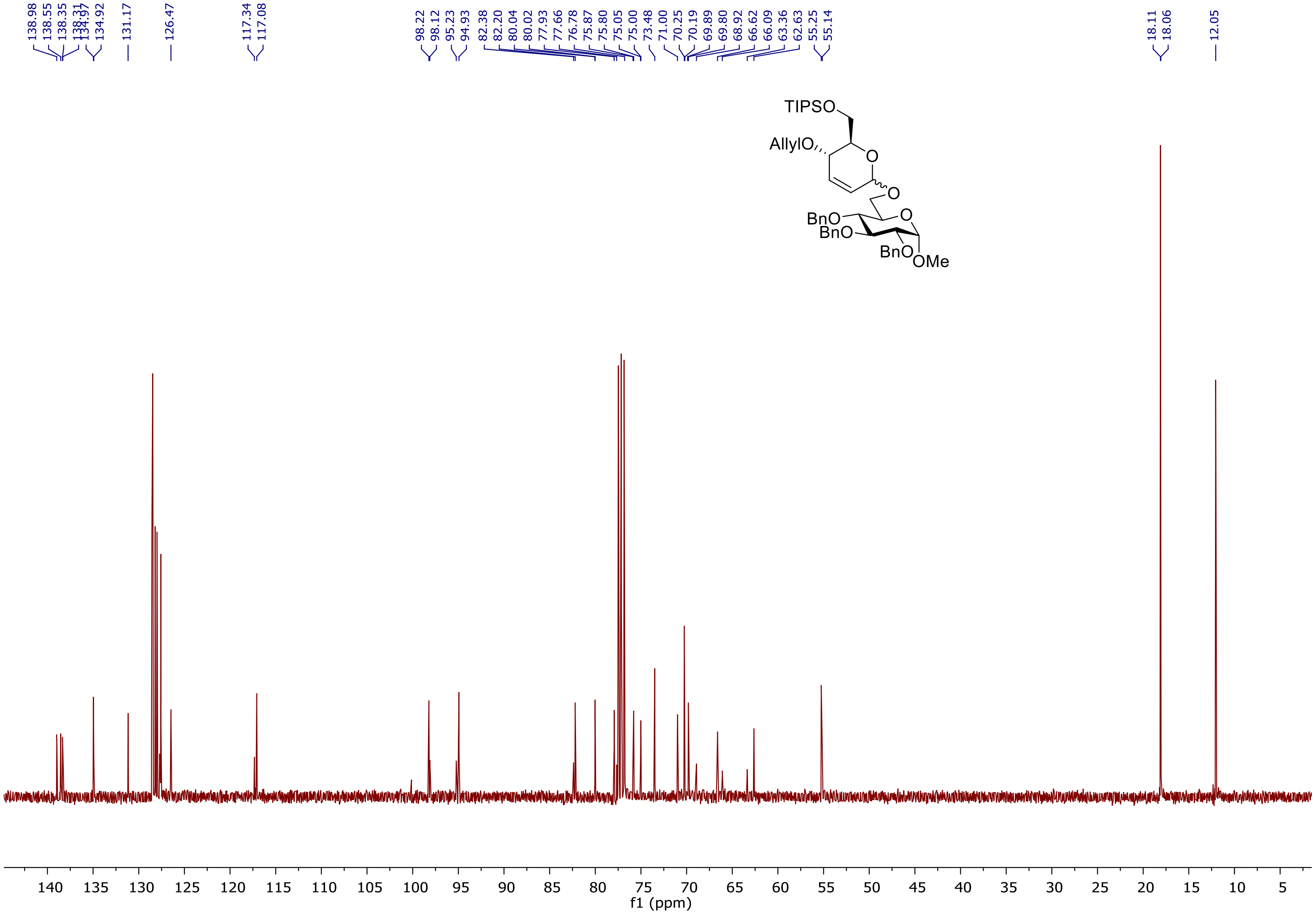

Methyl 2,3,4-tri-*O*-benzyl-6-*O*-(4,6-*O*-[Bis(*tert*-butyl)silylene]-3-*O*-triisopropylsilyl-α/β-D-erythro-hexapyranosyl)-α-D-glucopyranoside (6f) ^{1}H NMR (400 MHz; $CDCl_3$)

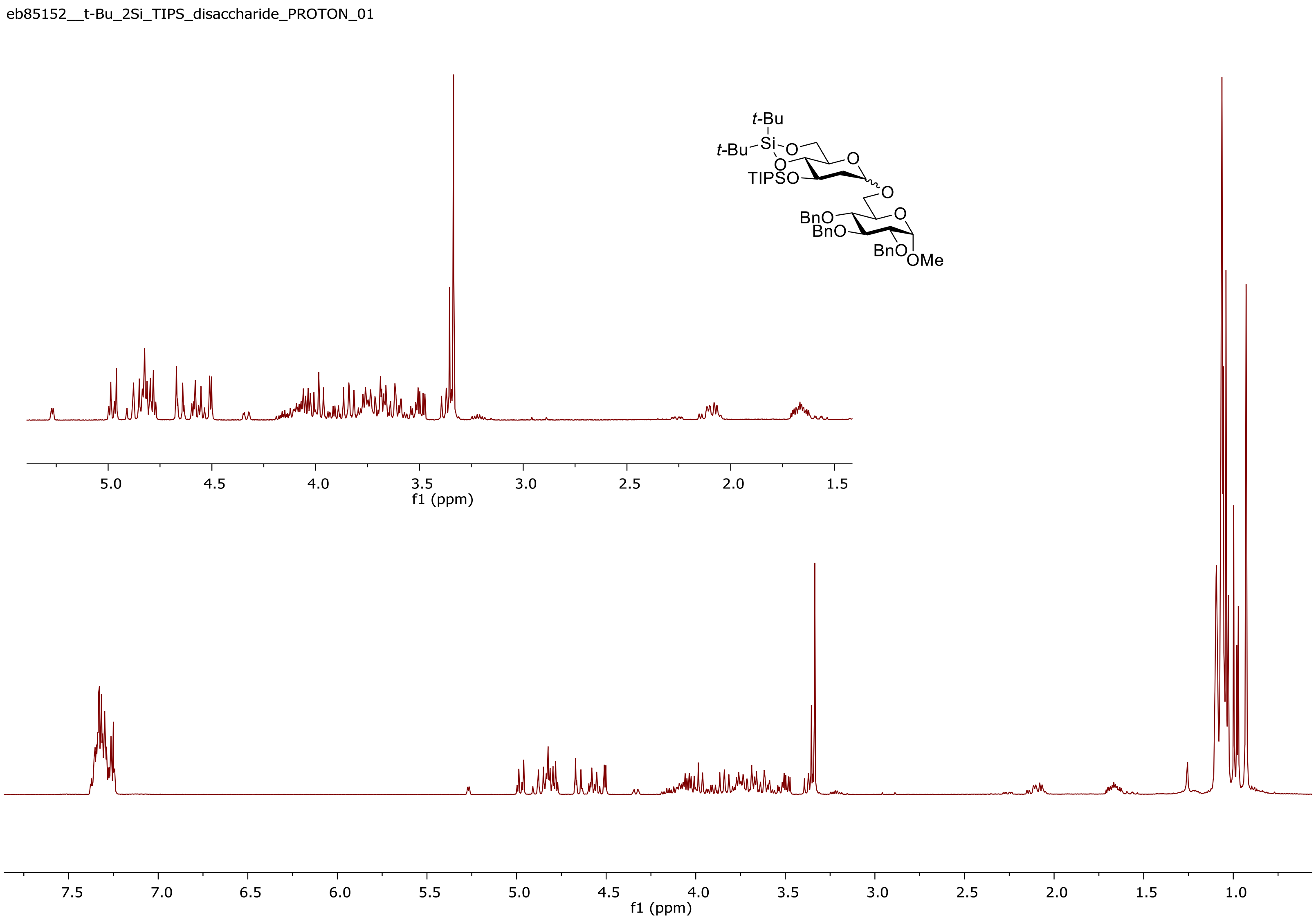

Methyl 2,3,4-tri-*O*-benzyl-6-*O*-(4,6-*O*-[Bis(*tert*-butyl)silylene]-3-*O*-triisopropylsilyl-α/β-D-erythro-hexapyranosyl)-α-D-glucopyranoside (6f) ^{13}C NMR (101 MHz; CDCl$_3$)

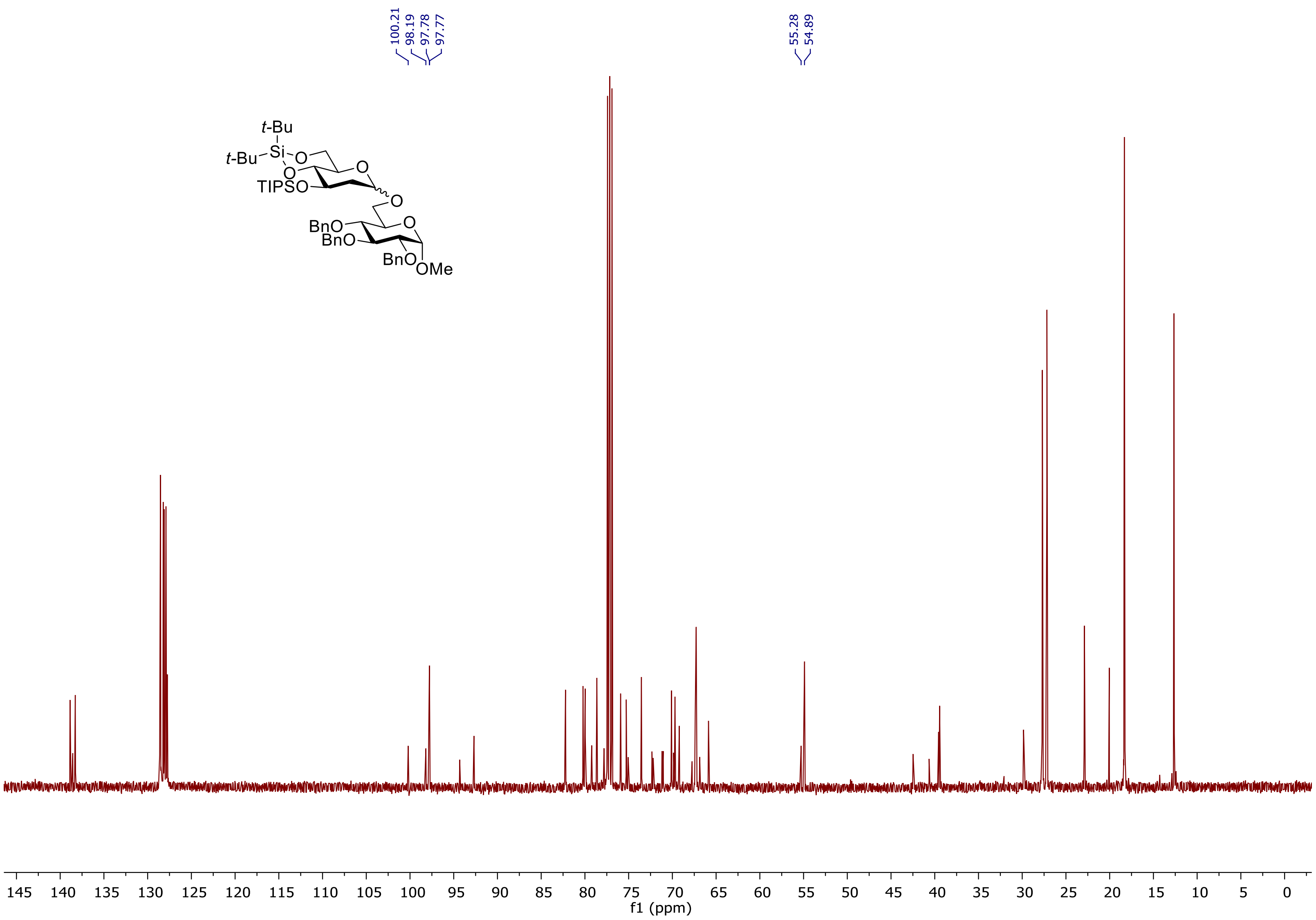

Methyl 2,3,4-tri-*O*-benzyl-6-*O*-(4,6-*O*-benzylidene-3-*O*-triisopropylsilyl-2-deoxy-α/β-D-erythro-hexapyranosyl)-α-D-glucopyranoside (6g)

^{1}H NMR (400 MHz; $CDCl_3$)

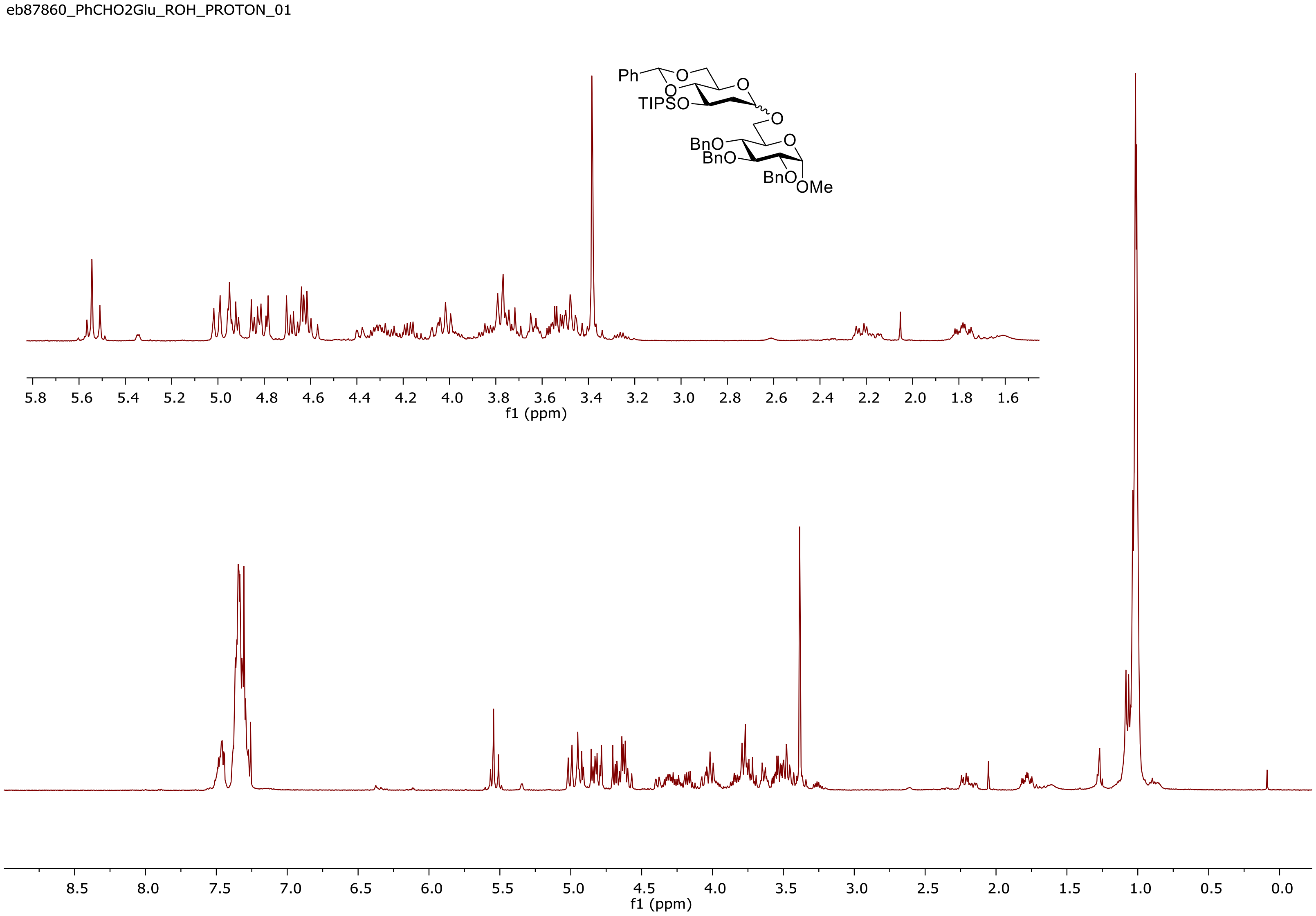

Methyl 2,3,4-tri-*O*-benzyl-6-*O*-(4,6-*O*-benzylidene-3-*O*-triisopropylsilyl-2-deoxy-α/β-D-erythro-hexapyranosyl)-α-D-glucopyranoside (6g)

^{13}C NMR (101 MHz; $CDCl_3$)

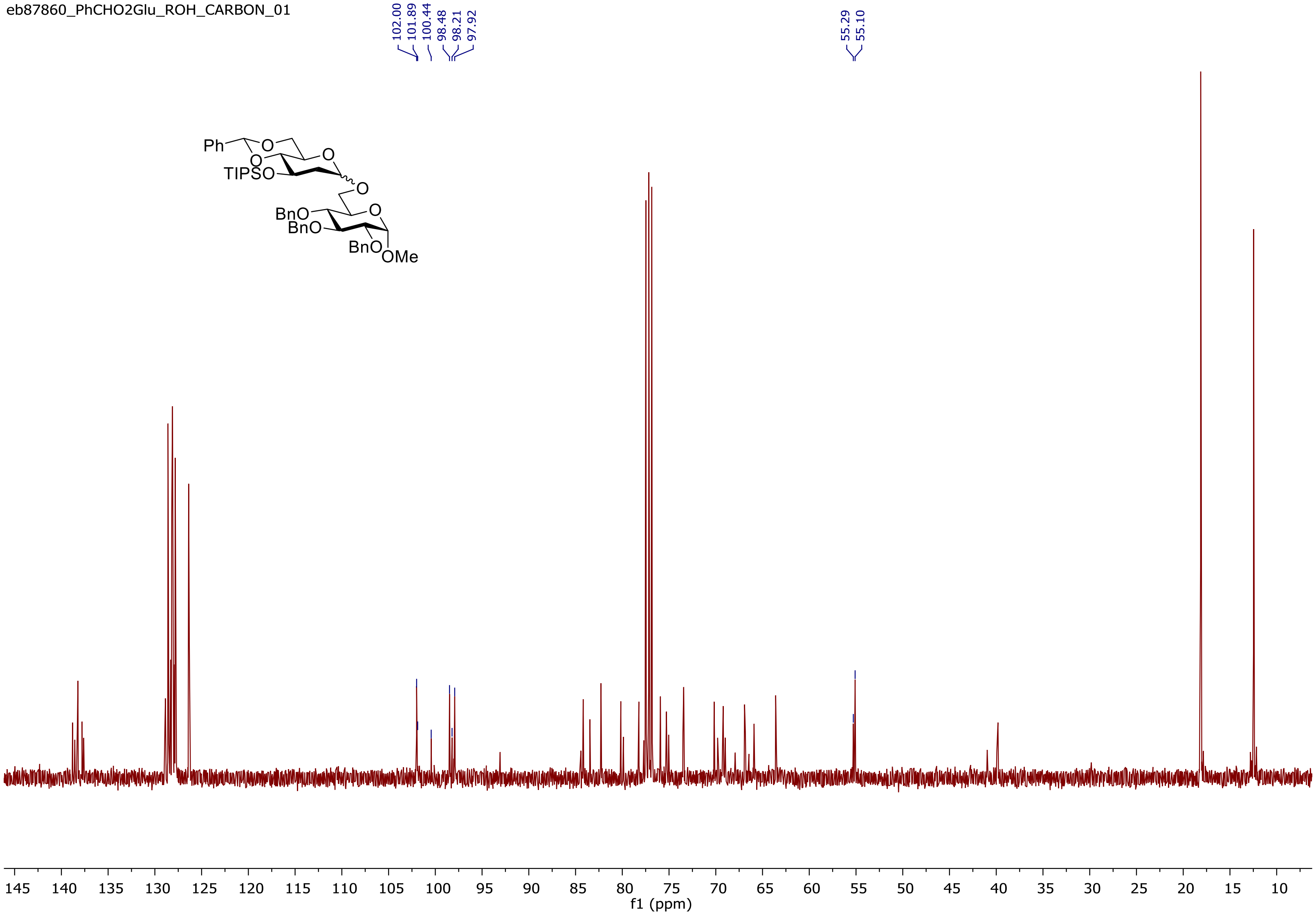

Methyl 2,3,4-tri-*O*-benzyl-6-*O*-(6-*O*-benzyl-2-deoxy-3,4-*O*-(1,1,3,3-tetraisopropyldisiloxane-1,3-diyl)-α-D-erythro-hexapyranosyl)-α-D-glucopyranoside (6h) ^{1}H NMR (400 MHz; $CDCl_3$)

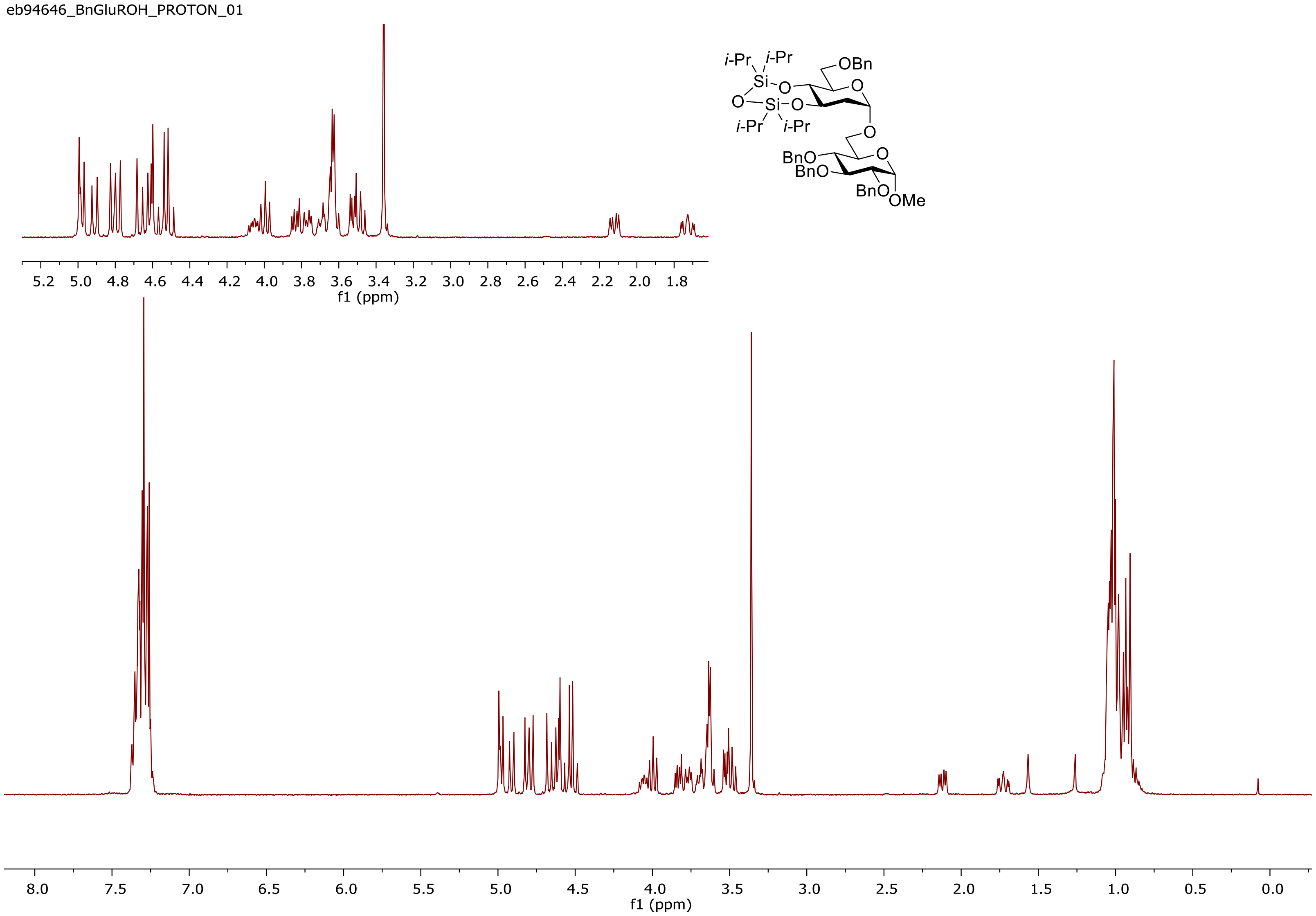

Methyl 2,3,4-tri-*O*-benzyl-6-*O*-(6-*O*-benzyl-2-deoxy-3,4-*O*-(1,1,3,3-tetraisopropyldisiloxane-1,3-diyl)-α-D-erythro-hexapyranosyl)-α-D-glucopyranoside (6h) ^{13}C NMR (101 MHz; $CDCl_3$)

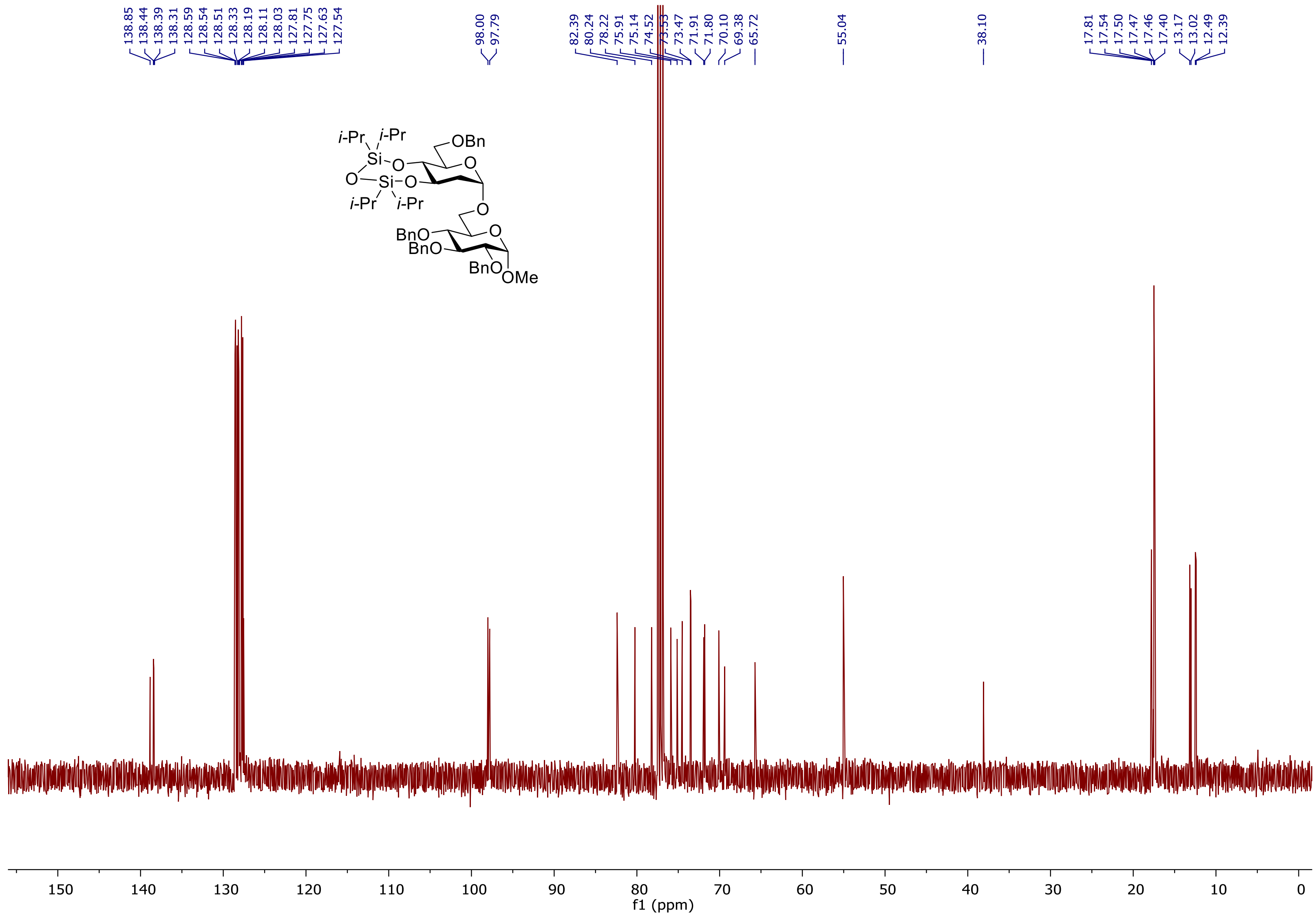

Methyl 2,3,4-tri-*O*-benzyl-6-*O*-(2-deoxy-3,4-*O*-(1,1,3,3-tetraisopropyldisiloxane-1,3-diyl)-6-*O*-triisopropylsilyl-α-D-erythro-hexapyranosyl)-α-D-glucopyranoside (6i) ^{1}H NMR (400 MHz; CDCl$_3$)

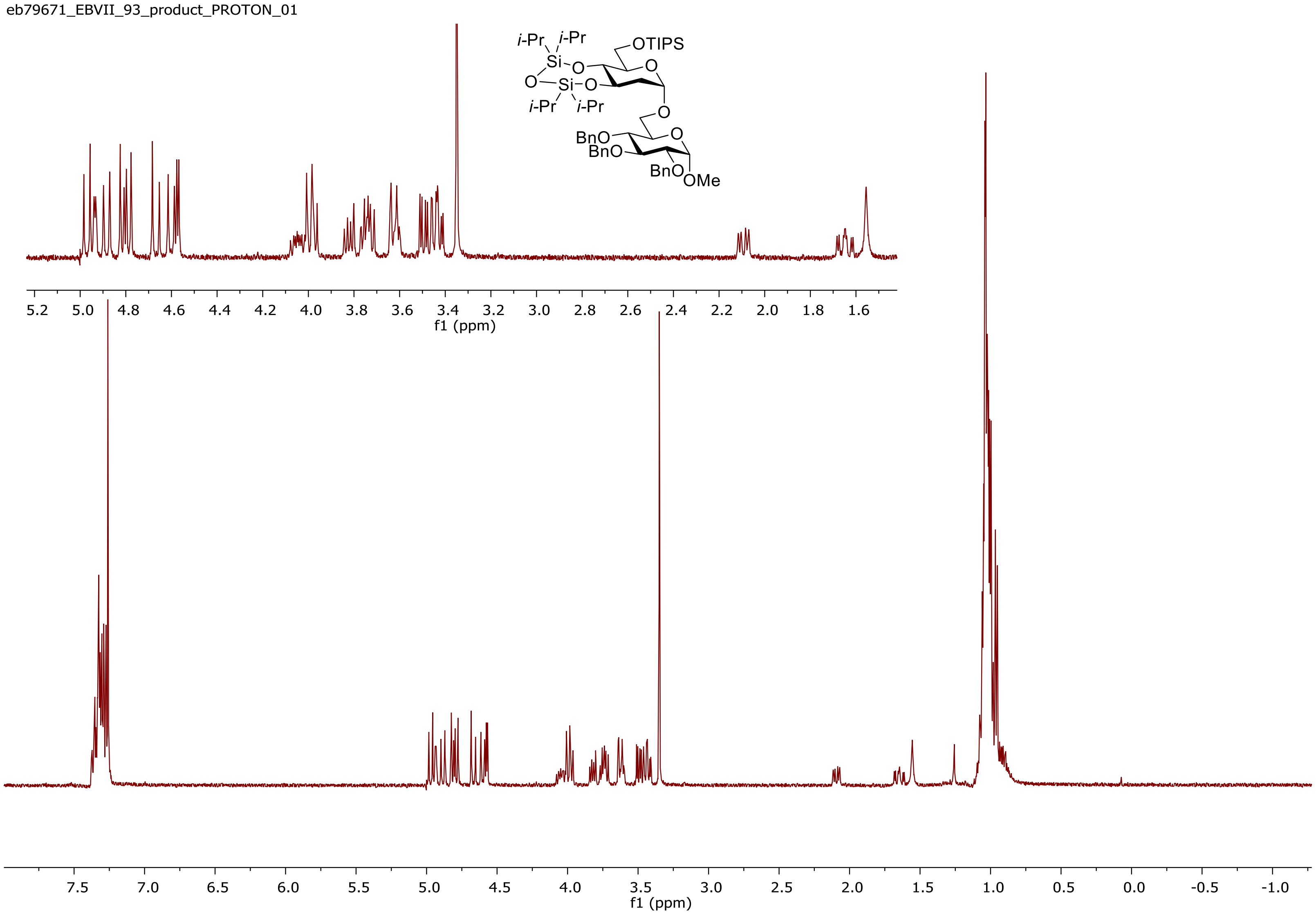

Methyl 2,3,4-tri-*O*-benzyl-6-*O*-(2-deoxy-3,4-*O*-(1,1,3,3-tetraisopropyldisiloxane-1,3-diyl)-6-*O*-triisopropylsilyl-α-D-erythro-hexapyranosyl)-α-D-glucopyranoside (6i) ^{13}C NMR (101 MHz; $CDCl_3$)

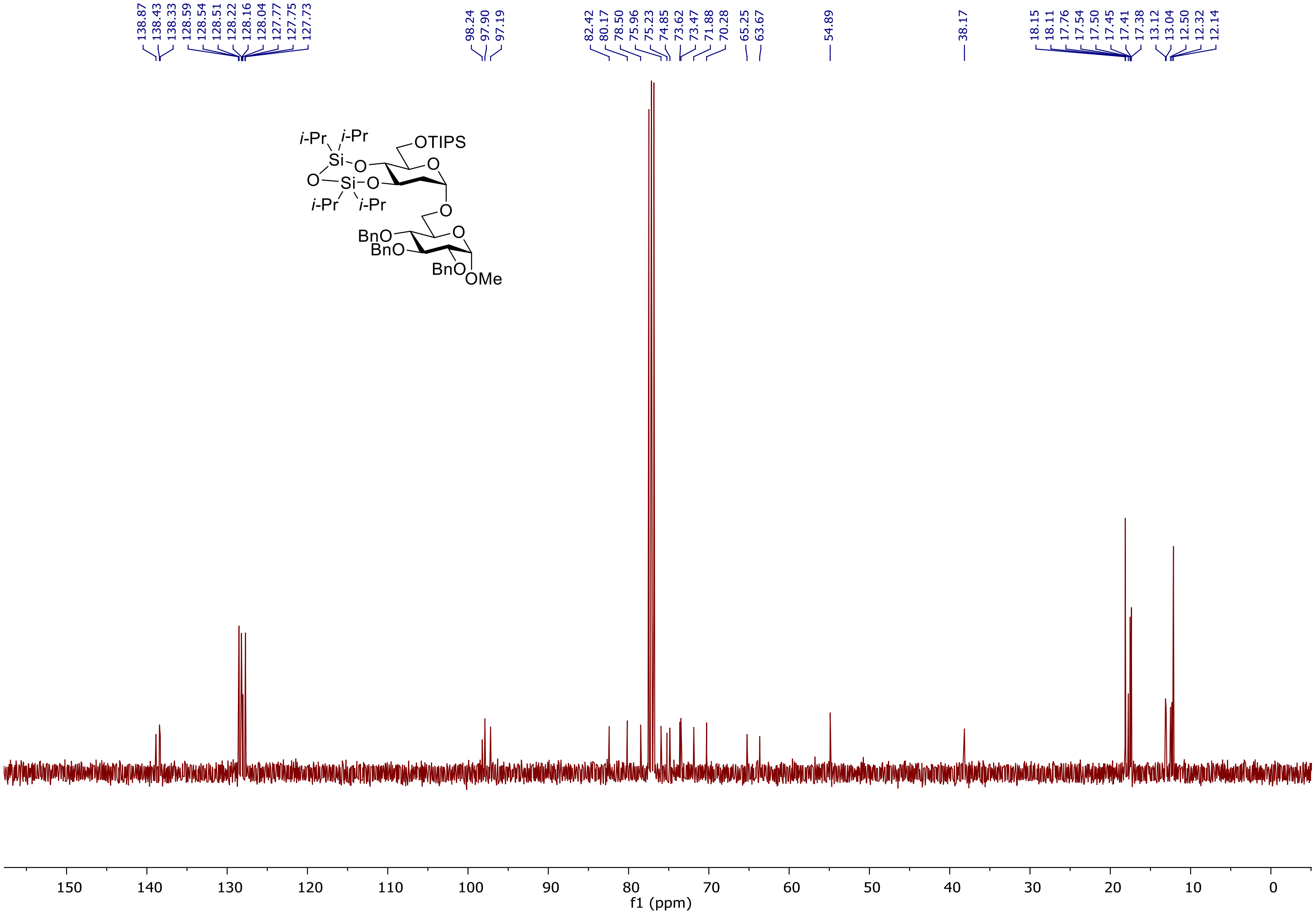

Methyl 2,3,4-tri-*O*-benzyl-6-*O*-(2-deoxy-3,4-*O*-(1,1,3,3-tetramethyldisiloxane-1,3-diyl)-6-*O*-triisopropylsilyl-α-D-erythro-hexapyranosyl)-α-D-glucopyranoside (6j) ^{1}H NMR (400 MHz; CDCl$_3$)

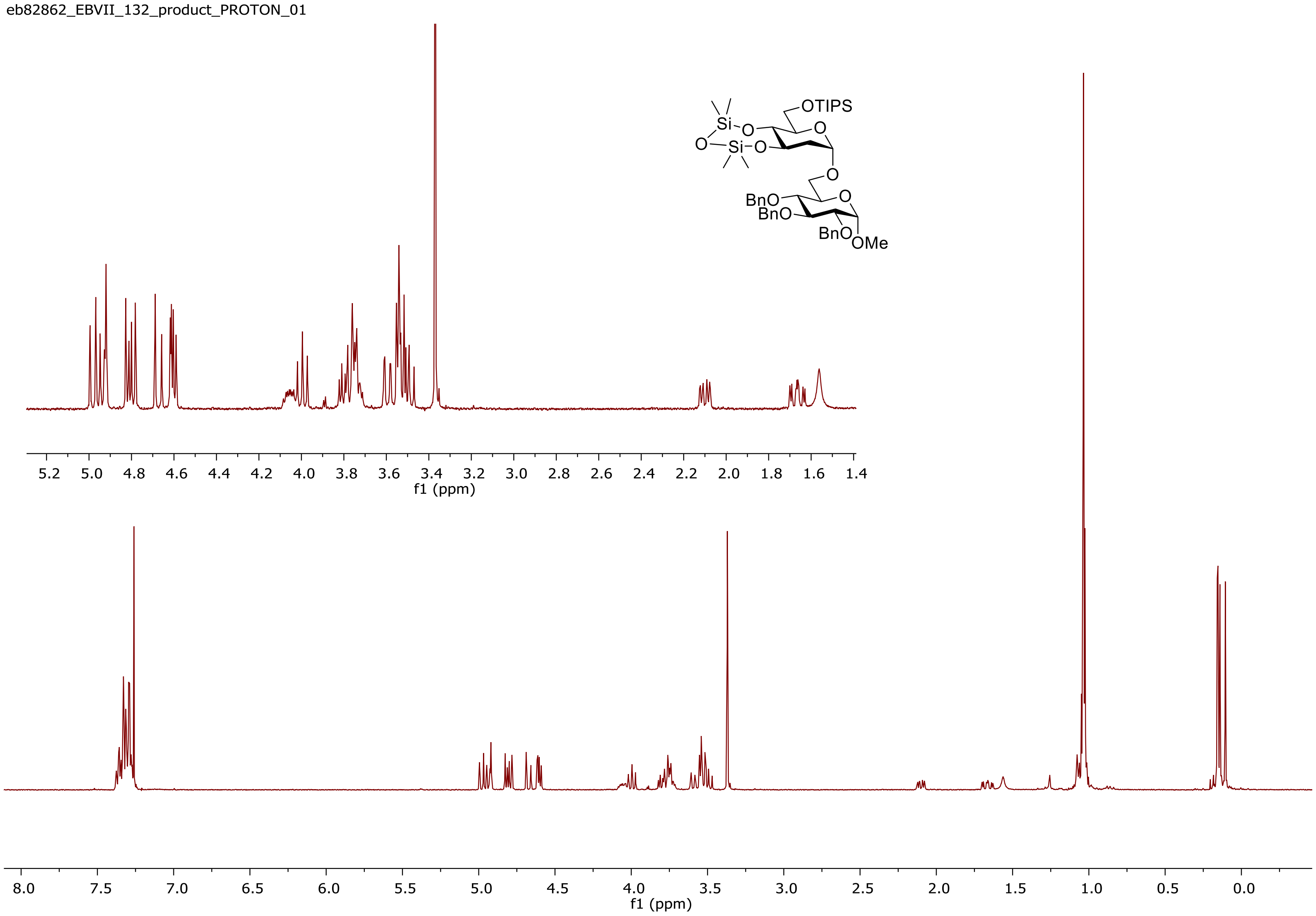

Methyl 2,3,4-tri-*O*-benzyl-6-*O*-(2-deoxy-3,4-*O*-(1,1,3,3-tetramethyldisiloxane-1,3-diyl)-6-*O*-triisopropylsilyl-α-D-erythro-hexapyranosyl)-α-D-glucopyranoside (6j) ^{13}C NMR (101 MHz; $CDCl_3$)

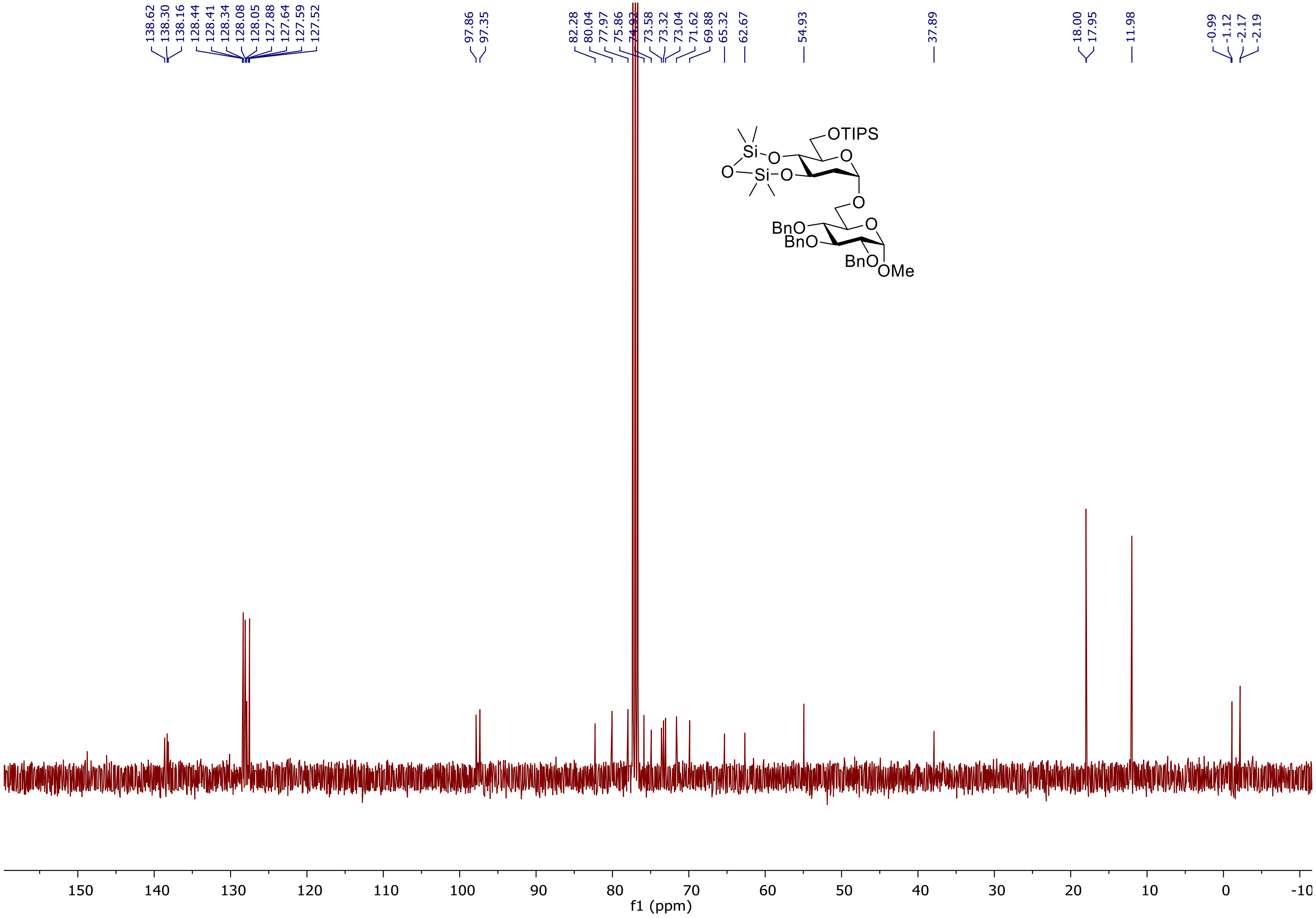

Phenyl 2,3,4-tri-*O*-benzoyl-6-*O*-(2-deoxy-3,4-*O*-(1,1,3,3-tetraisopropyldisiloxane-1,3-diyl)-6-*O*-triisopropylsilyl-α-D-erythro-hexapyranosyl)-β-D-thioglucopyranoside (8b) ^{1}H NMR (400 MHz; $CDCl_3$)

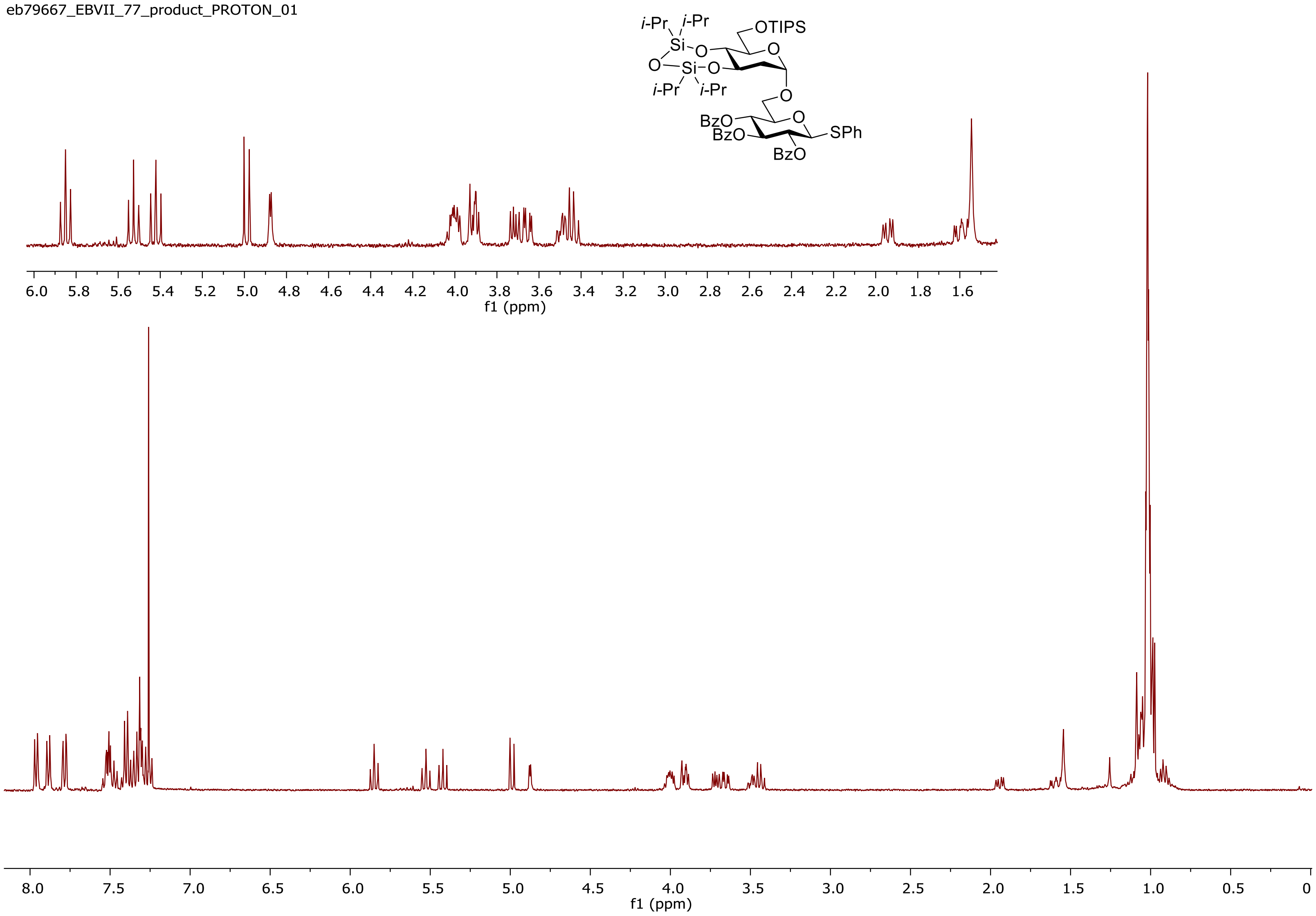

Phenyl 2,3,4-tri-*O*-benzoyl-6-*O*-(2-deoxy-3,4-*O*-(1,1,3,3-tetraisopropyldisiloxane-1,3-diyl)-6-*O*-triisopropylsilyl-α-D-erythro-hexapyranosyl)-β-D-thioglucopyranoside (8b) ^{13}C NMR (101 MHz; $CDCl_3$)

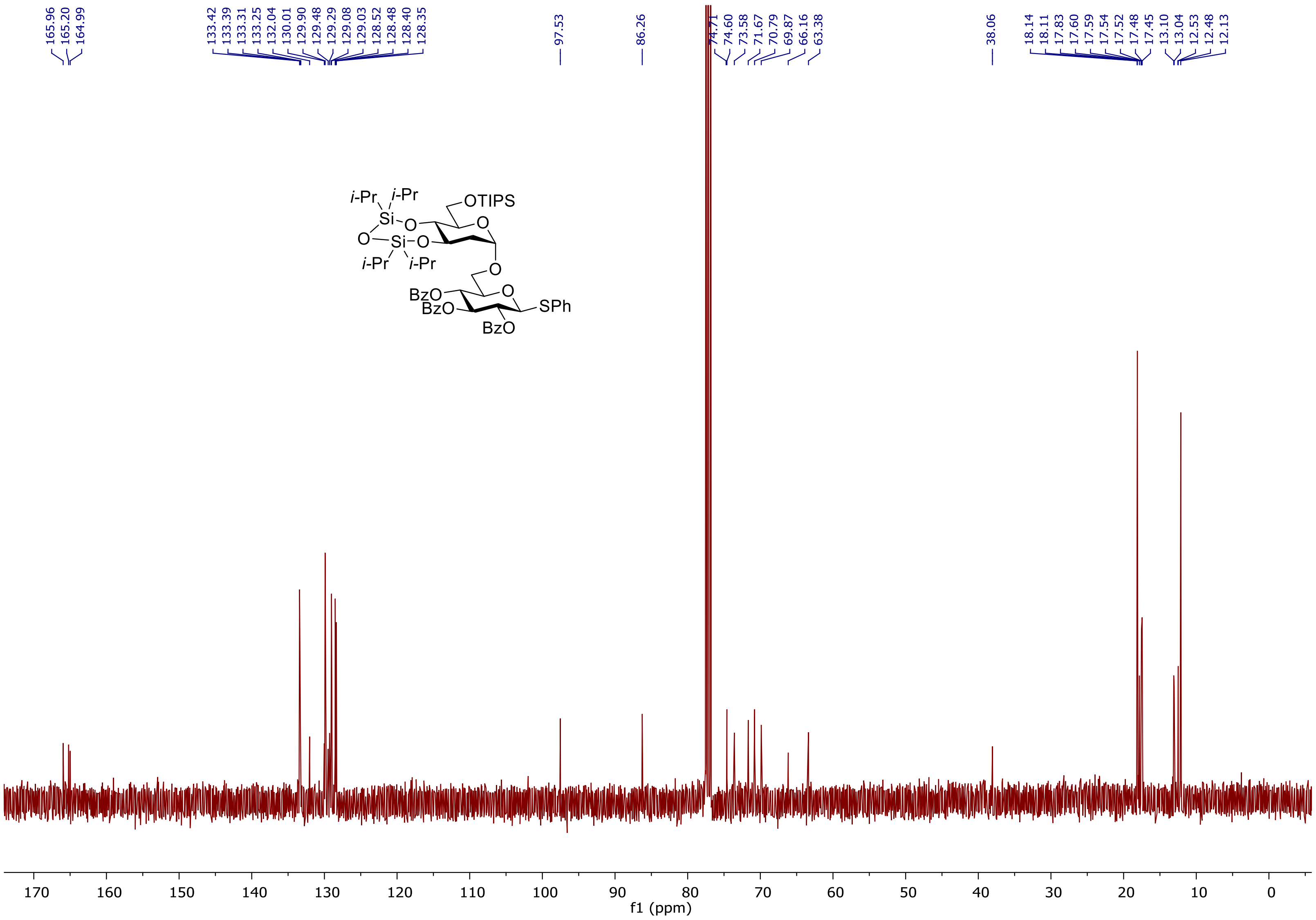

Methyl 3-*O*-benzyl-2-*O*-(2-deoxy-3,4-*O*-(1,1,3,3-tetraisopropyldisiloxane-1,3-diyl)-6-*O*-triisopropylsilyl-α-D-erythro-hexapyranosyl)-4,6-*O*-benzylidene-α-D-glucopyranoside (8c) ^{1}H NMR (400 MHz; $CDCl_3$)

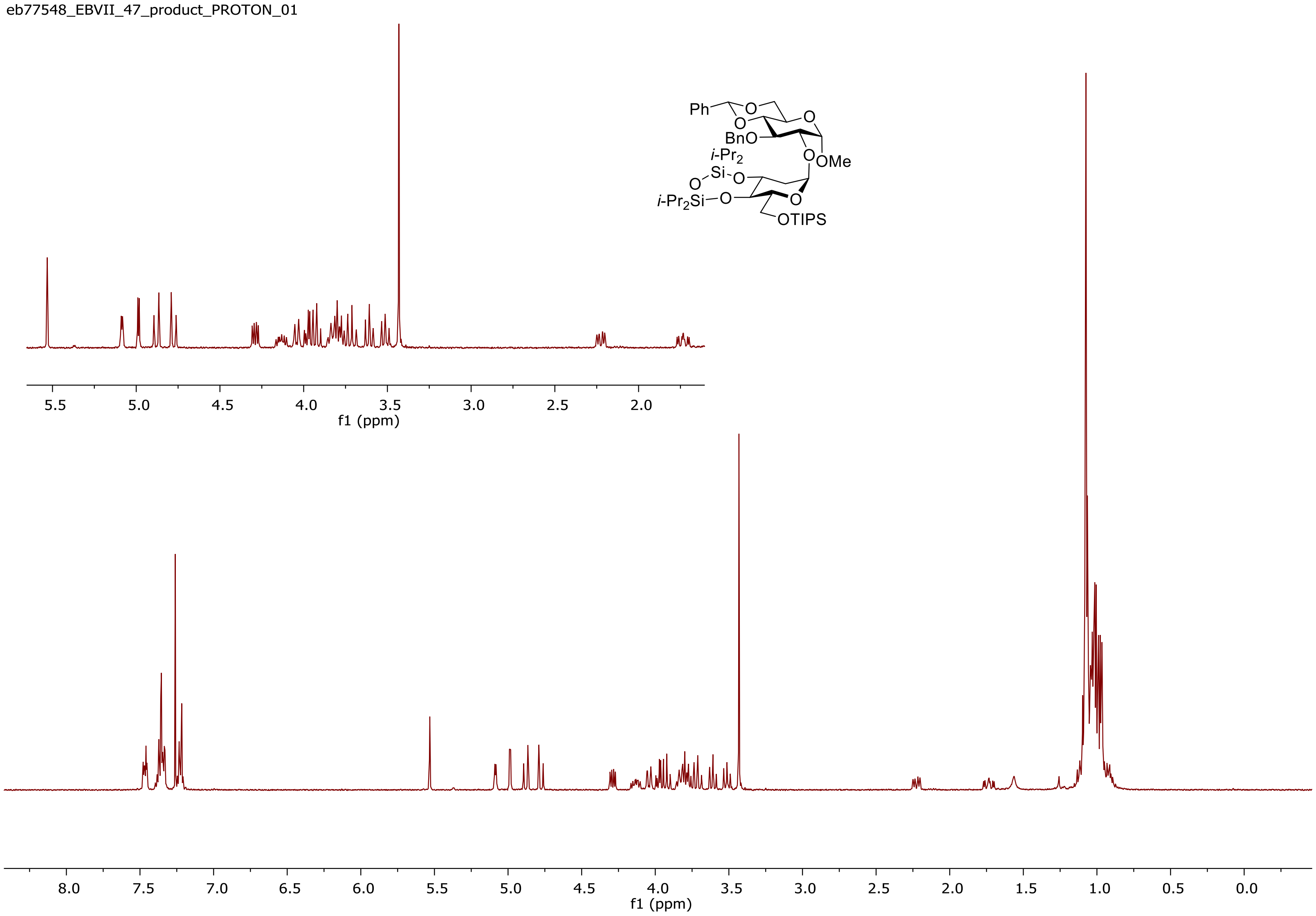

Methyl 3-*O*-benzyl-2-*O*-(2-deoxy-3,4-*O*-(1,1,3,3-tetraisopropyldisiloxane-1,3-diyl)-6-*O*-triisopropylsilyl-α-D-erythro-hexapyranosyl)-4,6-*O*-benzylidene-α-D-glucopyranoside (8c) ^{13}C NMR (101 MHz; $CDCl_3$)

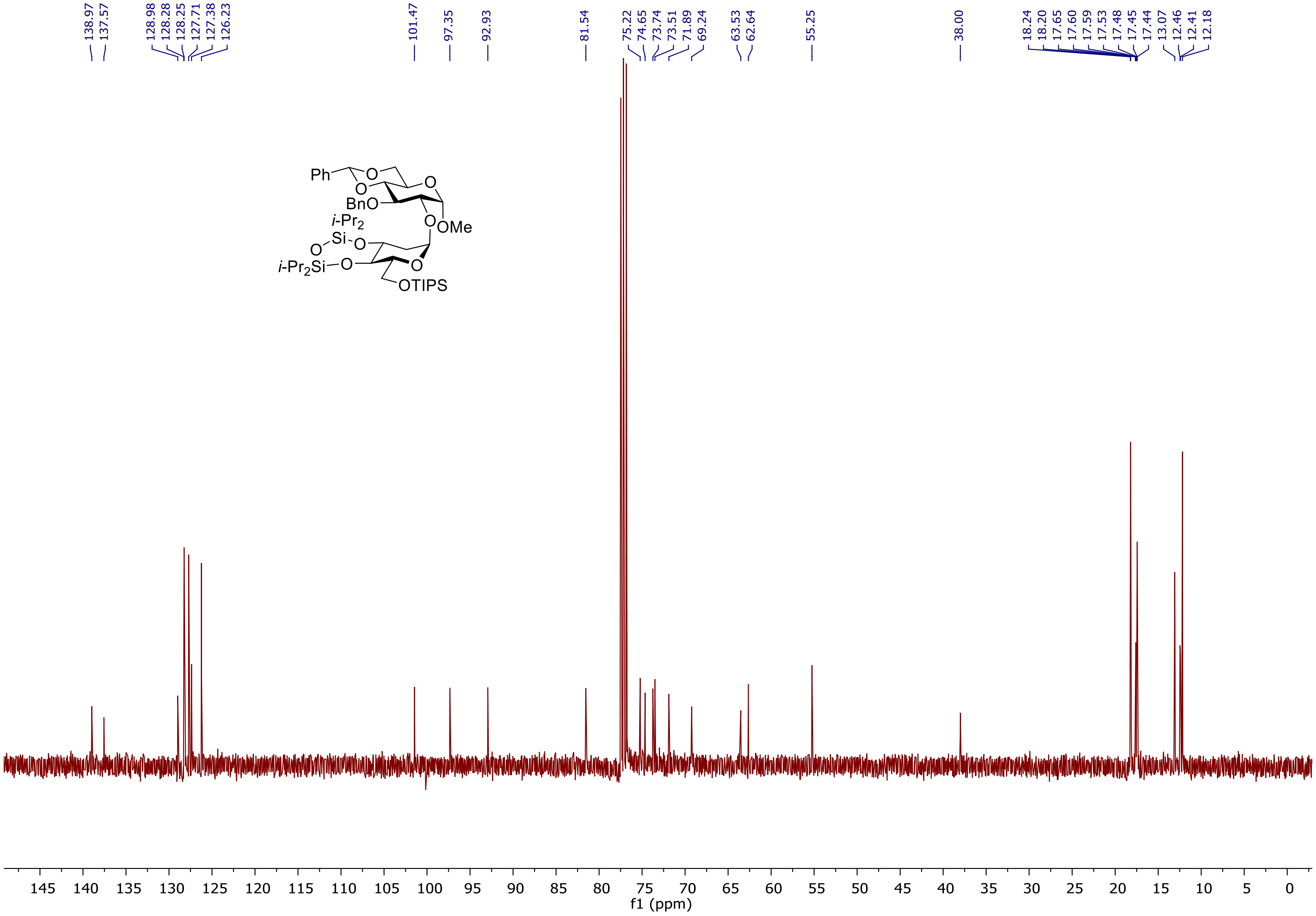

Phenyl 4,6-*O*-benzylidene-3-*O*-(2-deoxy-3,4-*O*-(1,1,3,3-tetraisopropyldisiloxane-1,3-diyl)-6-*O*-triisopropylsilyl-α-D-erythro-hexapyranosyl)-2-deoxy-1-thio-2-(2,2,2-trichloroethoxycarbonyl-amino)-β-D-glucopyranoside (8d) ^{1}H NMR (500 MHz; $CDCl_3$)

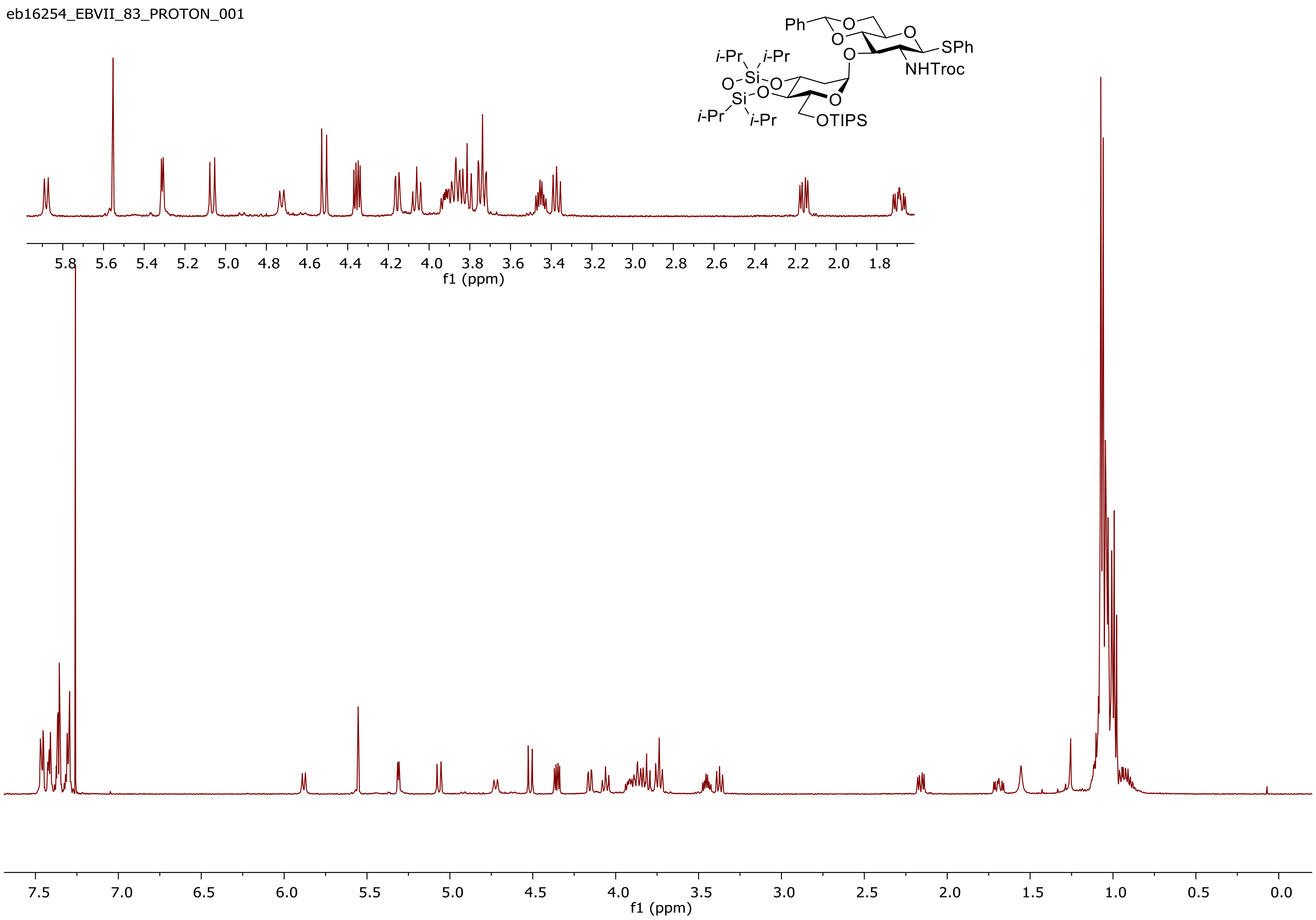

Phenyl 4,6-*O*-benzylidene-3-*O*-(2-deoxy-3,4-*O*-(1,1,3,3-tetraisopropyldisiloxane-1,3-diyl)-6-*O*-triisopropylsilyl-α-D-erythro-hexapyranosyl)-2-deoxy-1-thio-2-(2,2,2-trichloroethoxycarbonyl-amino)-β-D-glucopyranoside (8d) ^{13}C NMR (126 MHz; $CDCl_3$)

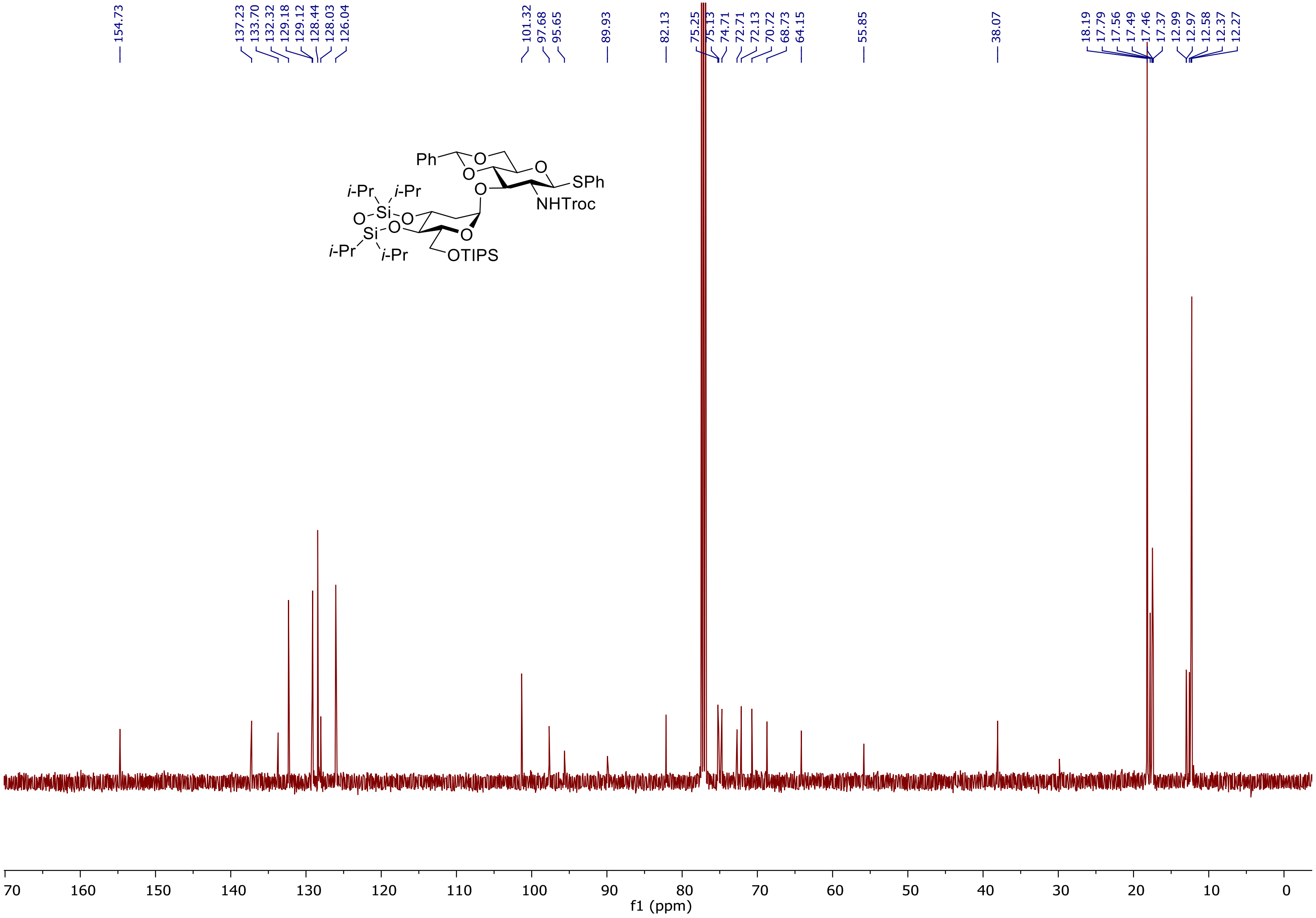

***O*-(2-Deoxy-3,4-*O*-(1,1,3,3-tetraisopropyldisiloxane-1,3-diyl)-6-*O*-triisopropylsilyl-α-D-erythro-hexapyranosyl)-*N*-(*tert*-Butoxycarbonyl)-L-serine methyl ester (8e) ^{1}H NMR (500 MHz; $CDCl_3$)**

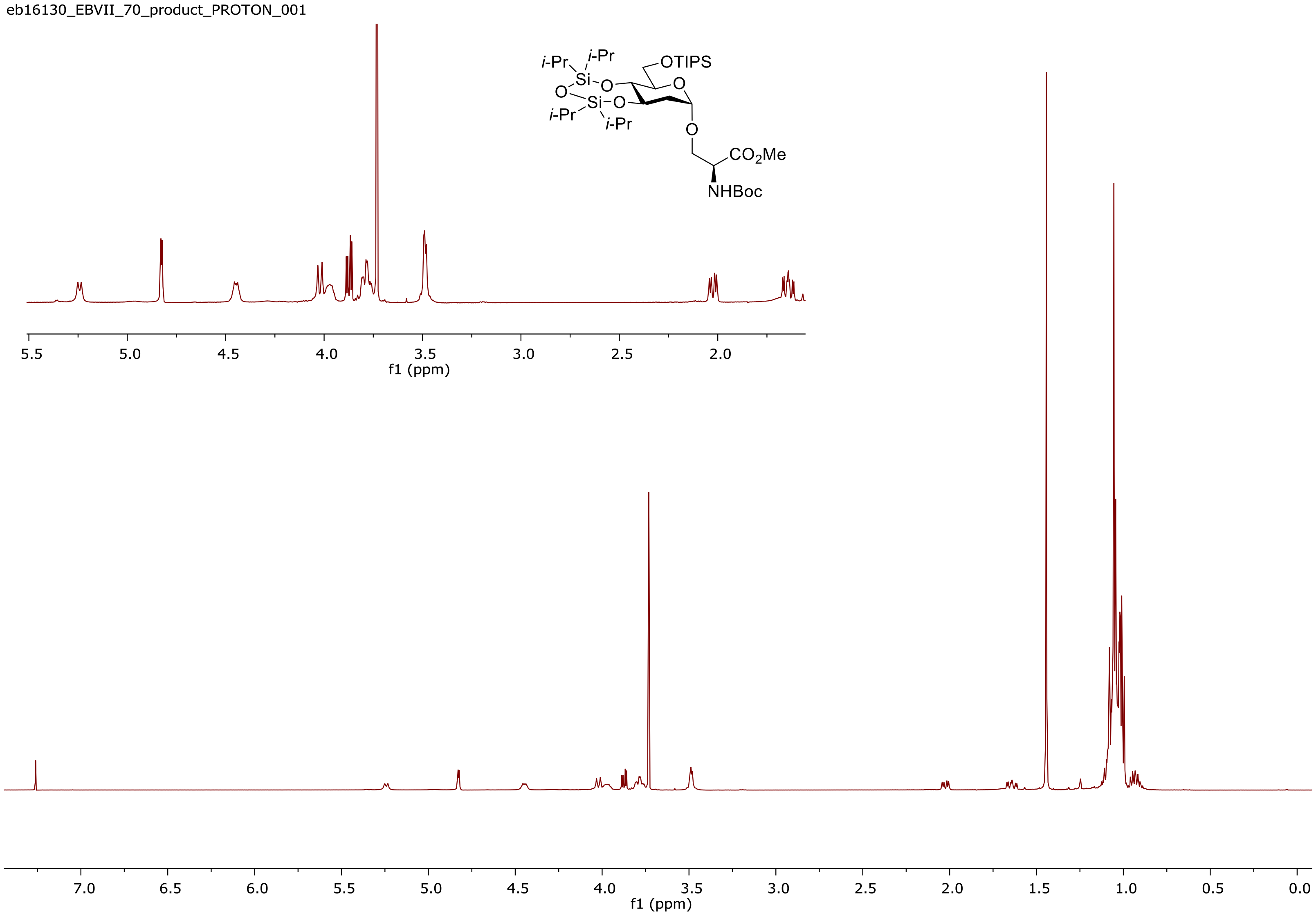

O-(2-Deoxy-3,4-O-(1,1,3,3-tetraisopropyldisiloxane-1,3-diyl)-6-O-triisopropylsilyl-α-D-erythro-hexapyranosyl)-N-(tert-Butoxycarbonyl)-L-serine methyl ester (8e) ^{13}C NMR (126 MHz; $CDCl_3$)

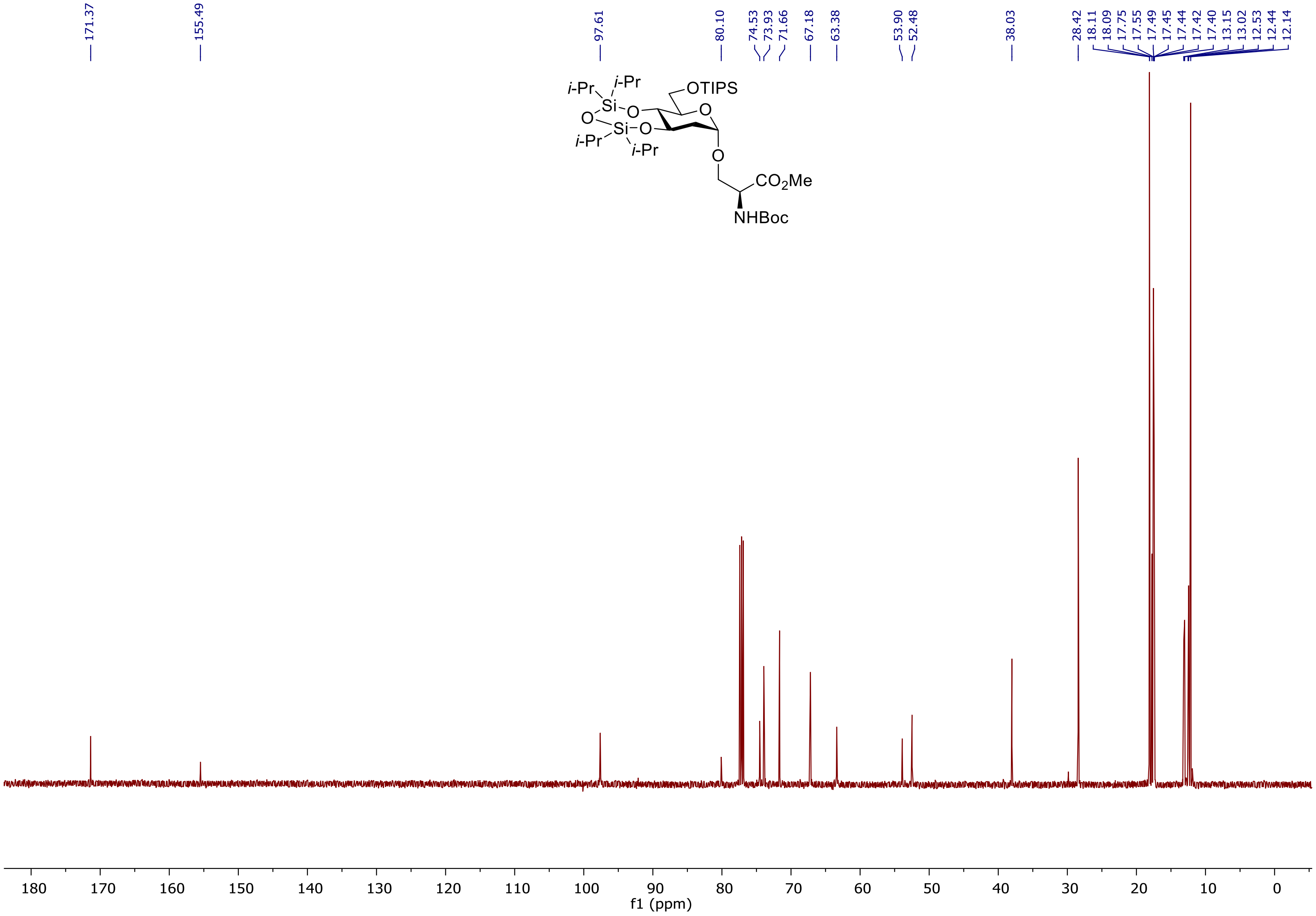

***O*-(2-Deoxy-3,4-*O*-(1,1,3,3-tetraisopropyldisiloxane-1,3-diyl)-6-*O*-triisopropylsilyl-α-D-erythro-hexapyranosyl)-*N*-[(9-fluorenylmethoxy)carbonyl]-L-serine methyl ester (8f) ^{1}H NMR (500 MHz; $CDCl_3$)**

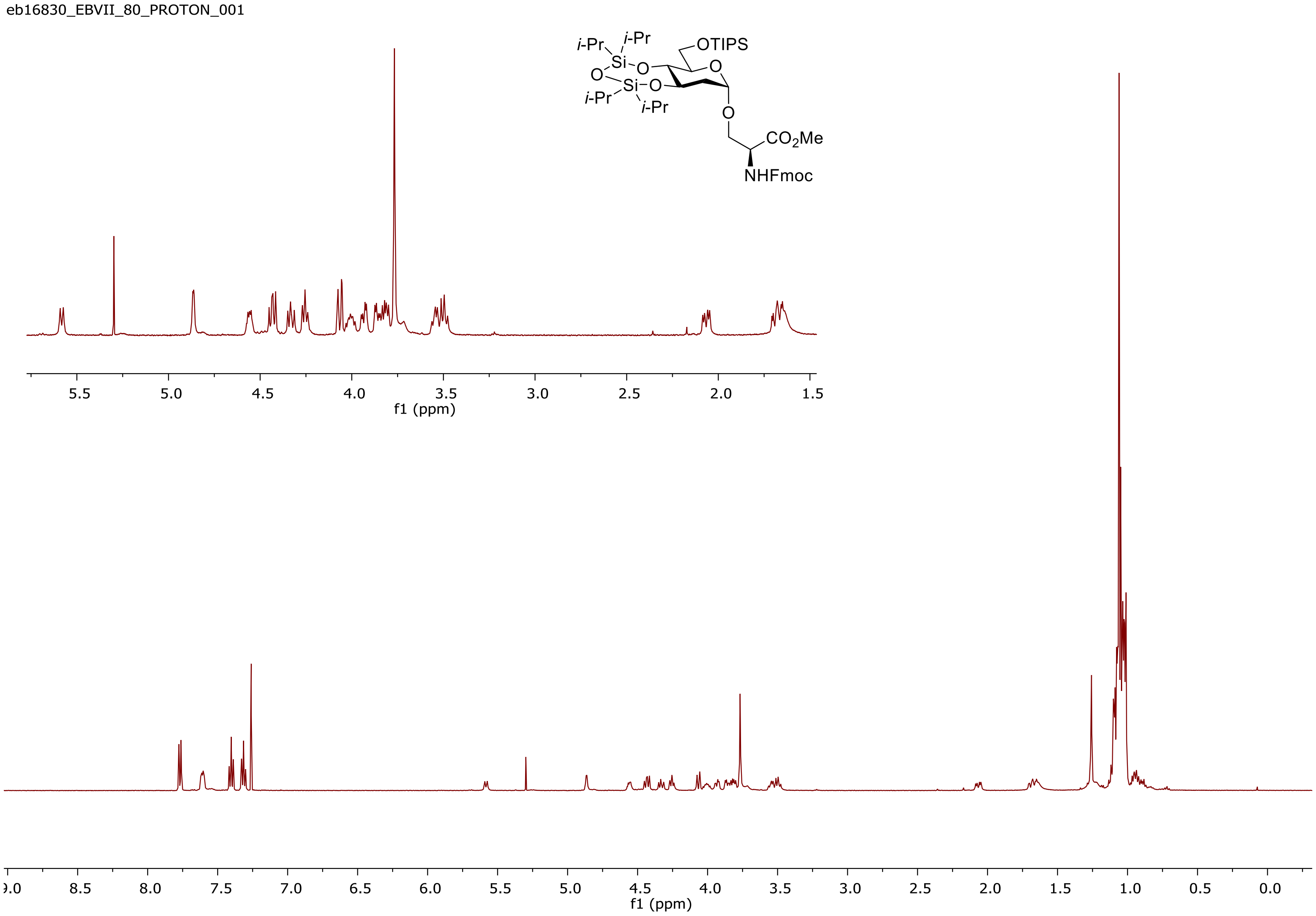

_O_-(2-Deoxy-3,4-_O_-(1,1,3,3-tetraisopropyldisiloxane-1,3-diyl)-6-_O_-triisopropylsilyl-α-D-erythro-hexapyranosyl)-_N_-[(9-fluorenylmethoxy)carbonyl]-L-serine methyl ester (8f) ^{13}C NMR (126 MHz; $CDCl_3$)

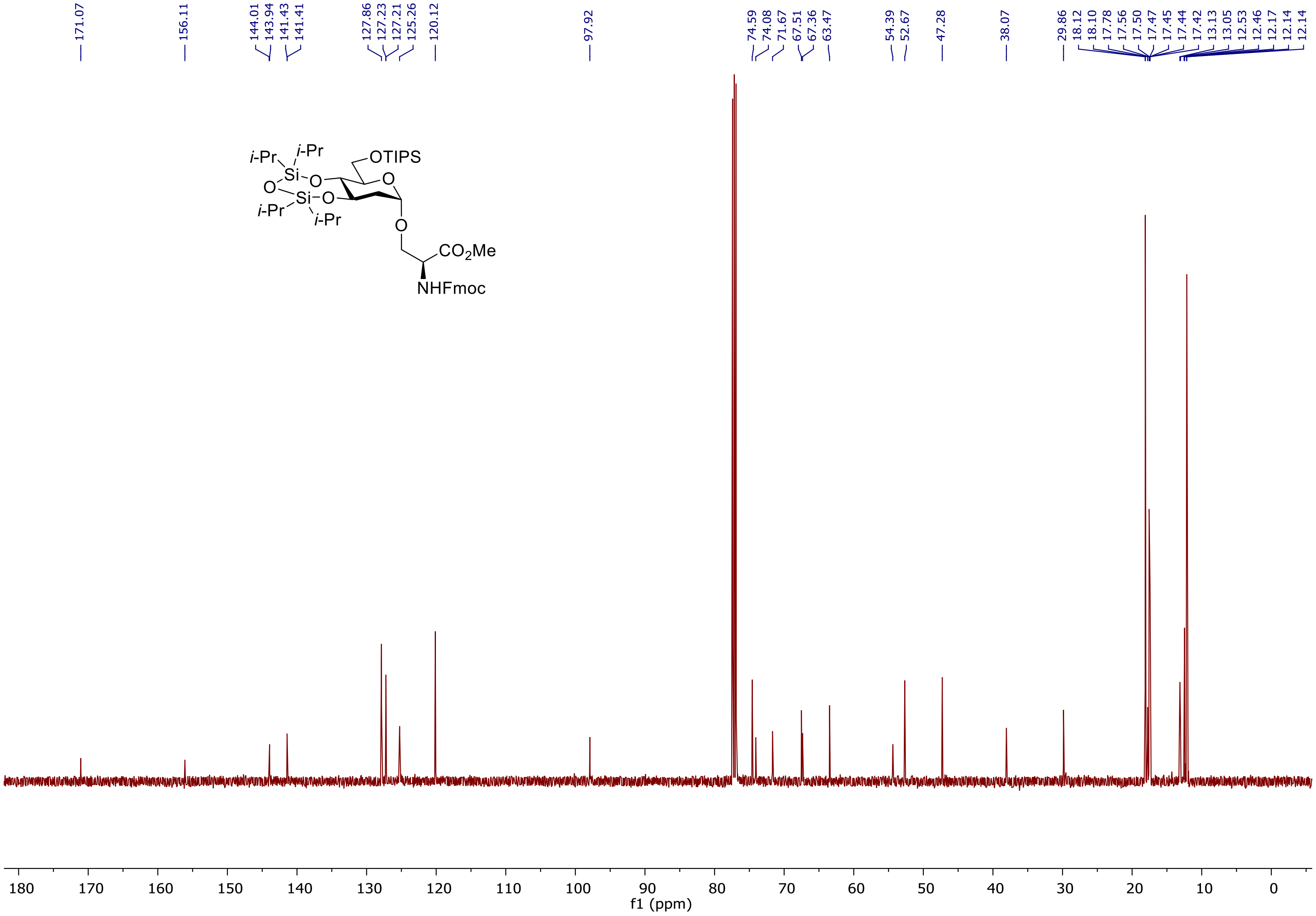

_O_-(2-Deoxy-3,4-_O_-(1,1,3,3-tetraisopropyldisiloxane-1,3-diyl)-6-_O_-triisopropylsilyl-α-D-erythro-hexapyranosyl)-_N_-(_tert_-Butoxycarbonyl)-L-threonine methyl ester (8g) ^{1}H NMR (400 MHz; $CDCl_3$)

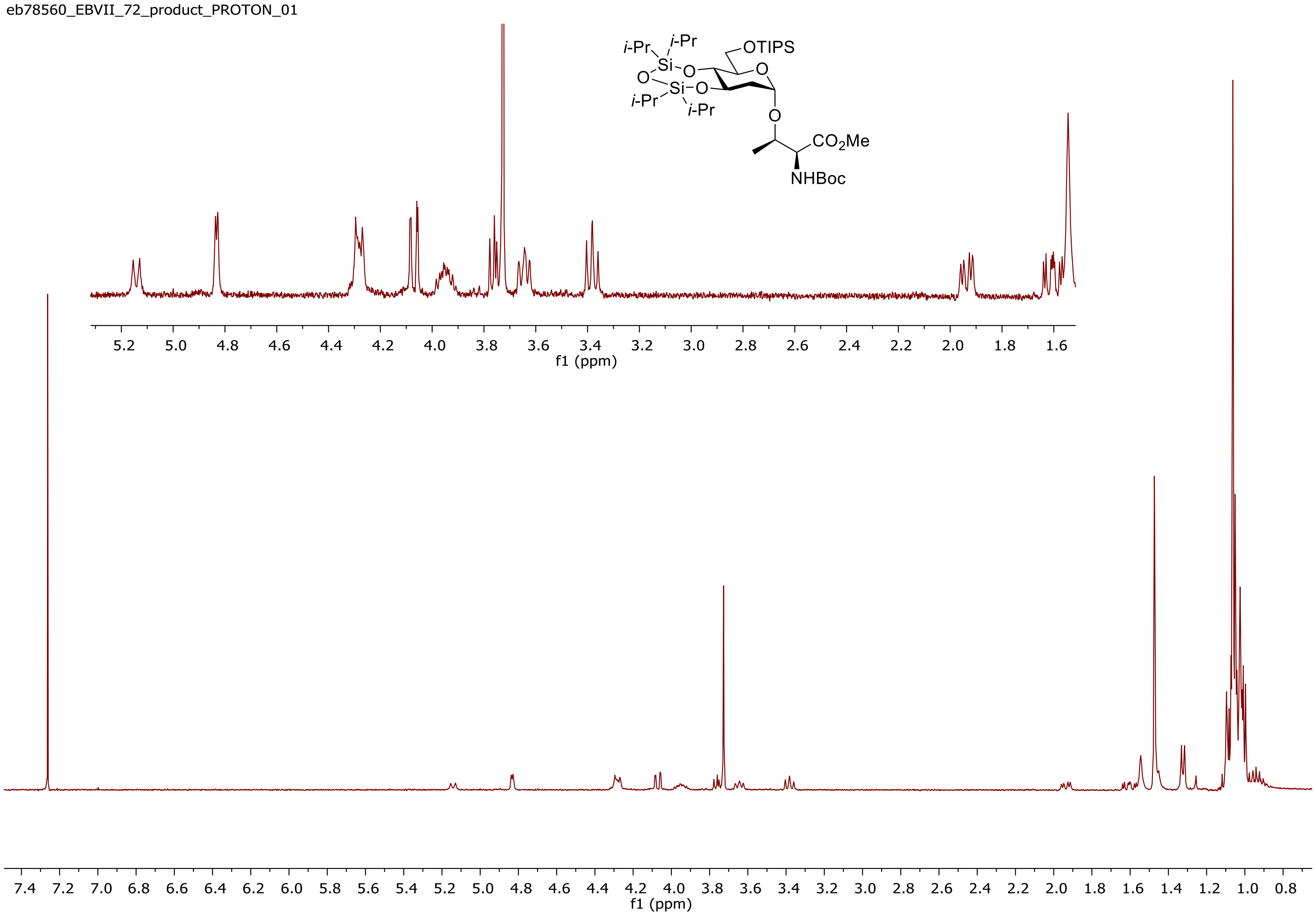

***O*-(2-Deoxy-3,4-*O*-(1,1,3,3-tetraisopropyldisiloxane-1,3-diyl)-6-*O*-triisopropylsilyl-α-D-erythro-hexapyranosyl)-*N*-(*tert*-Butoxycarbonyl)-L-threonine methyl ester (8g) ^{13}C NMR (126 MHz; CDCl$_3$)**

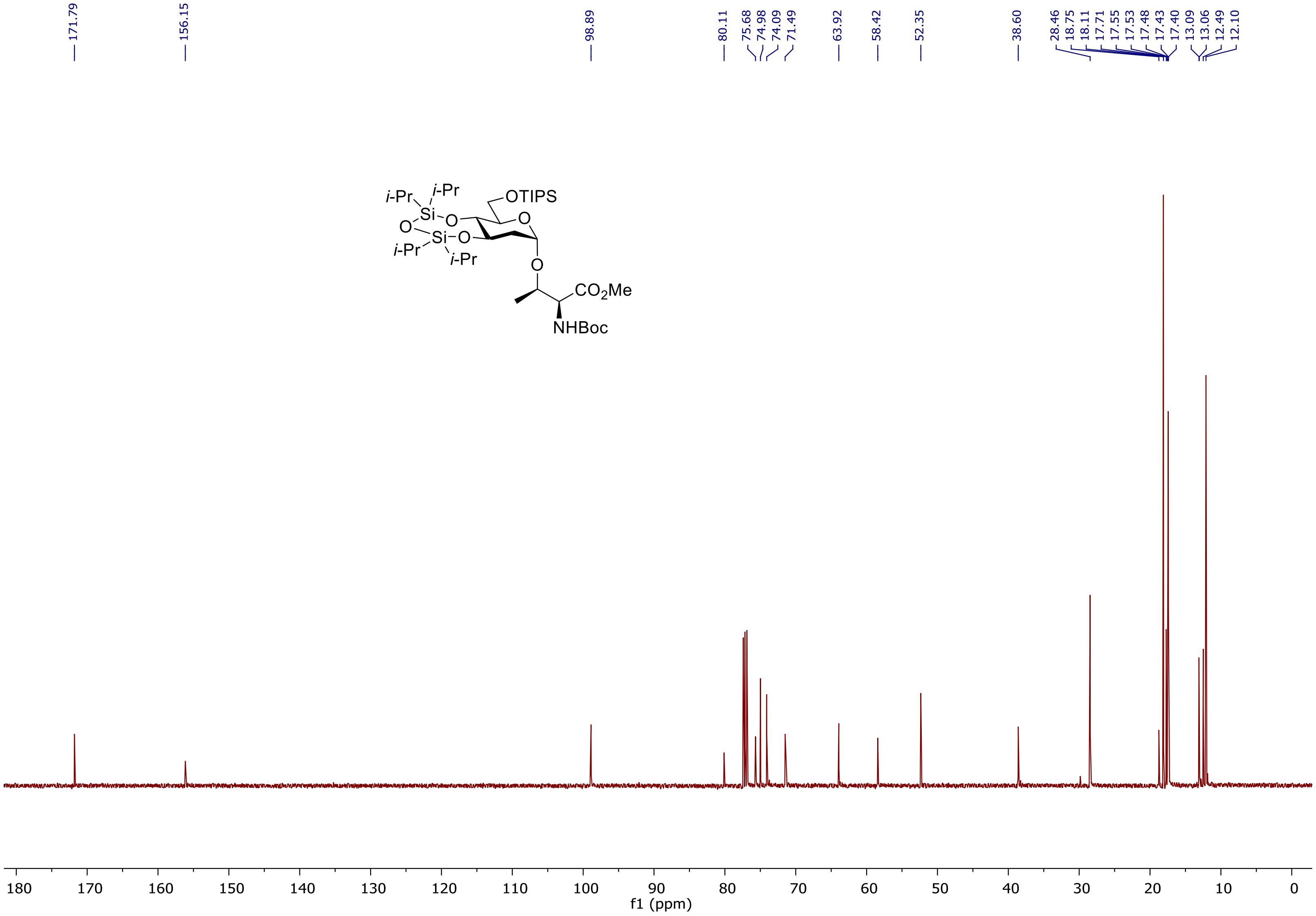

Cholesteryl (2-deoxy-3,4-*O*-(1,1,3,3-tetraisopropyldisiloxane-1,3-diyl)-6-*O*-triisopropylsilyl-α/β-D-erythro-hexapyranosyl) (8h) ^{1}H NMR (500 MHz; $CDCl_3$)

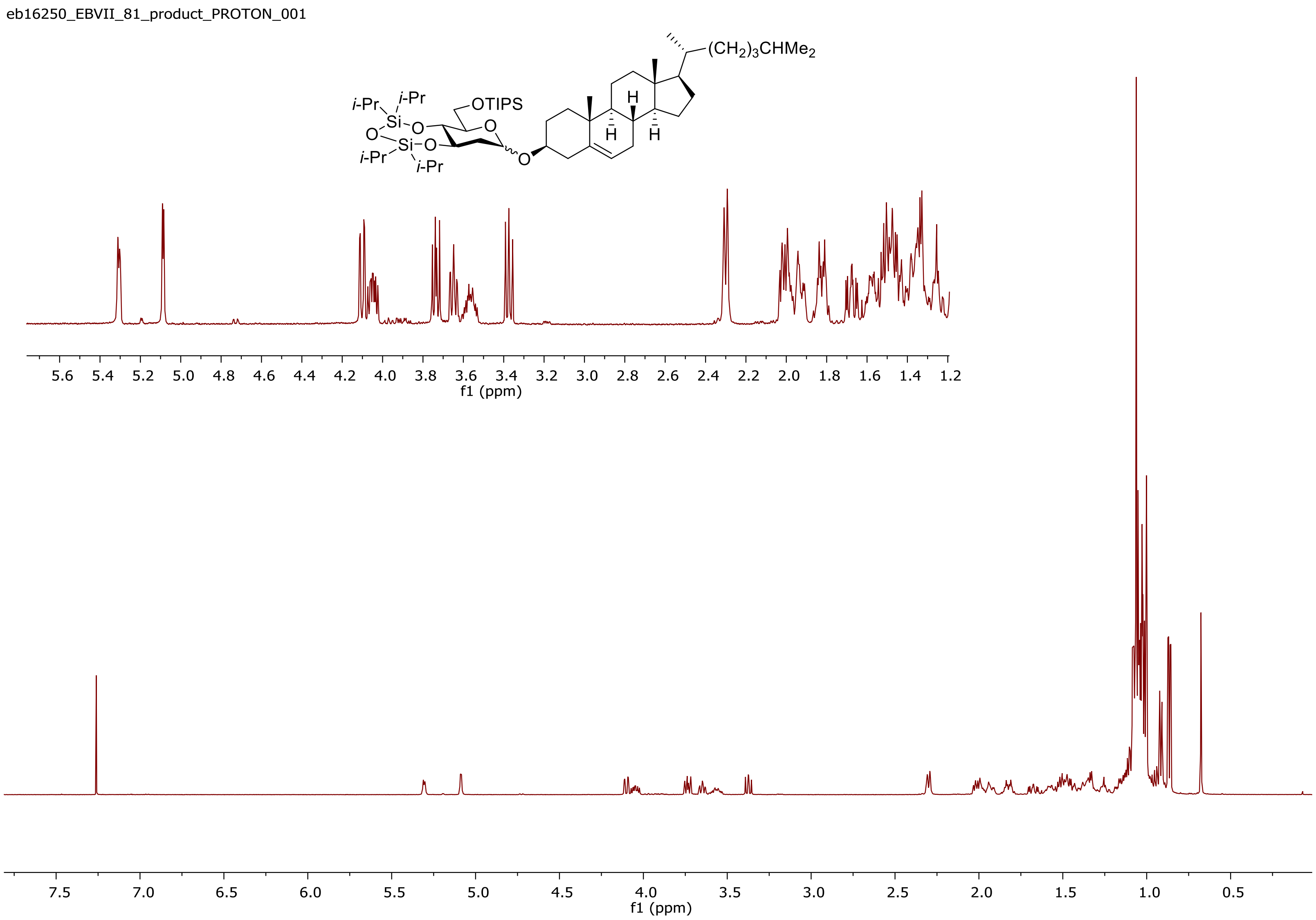

Cholesteryl (2-deoxy-3,4-*O*-(1,1,3,3-tetraisopropyldisiloxane-1,3-diyl)-6-*O*-triisopropylsilyl-α/β-D-erythro-hexapyranosyl) (8h) ^{13}C NMR (126 MHz; CDCl$_3$)

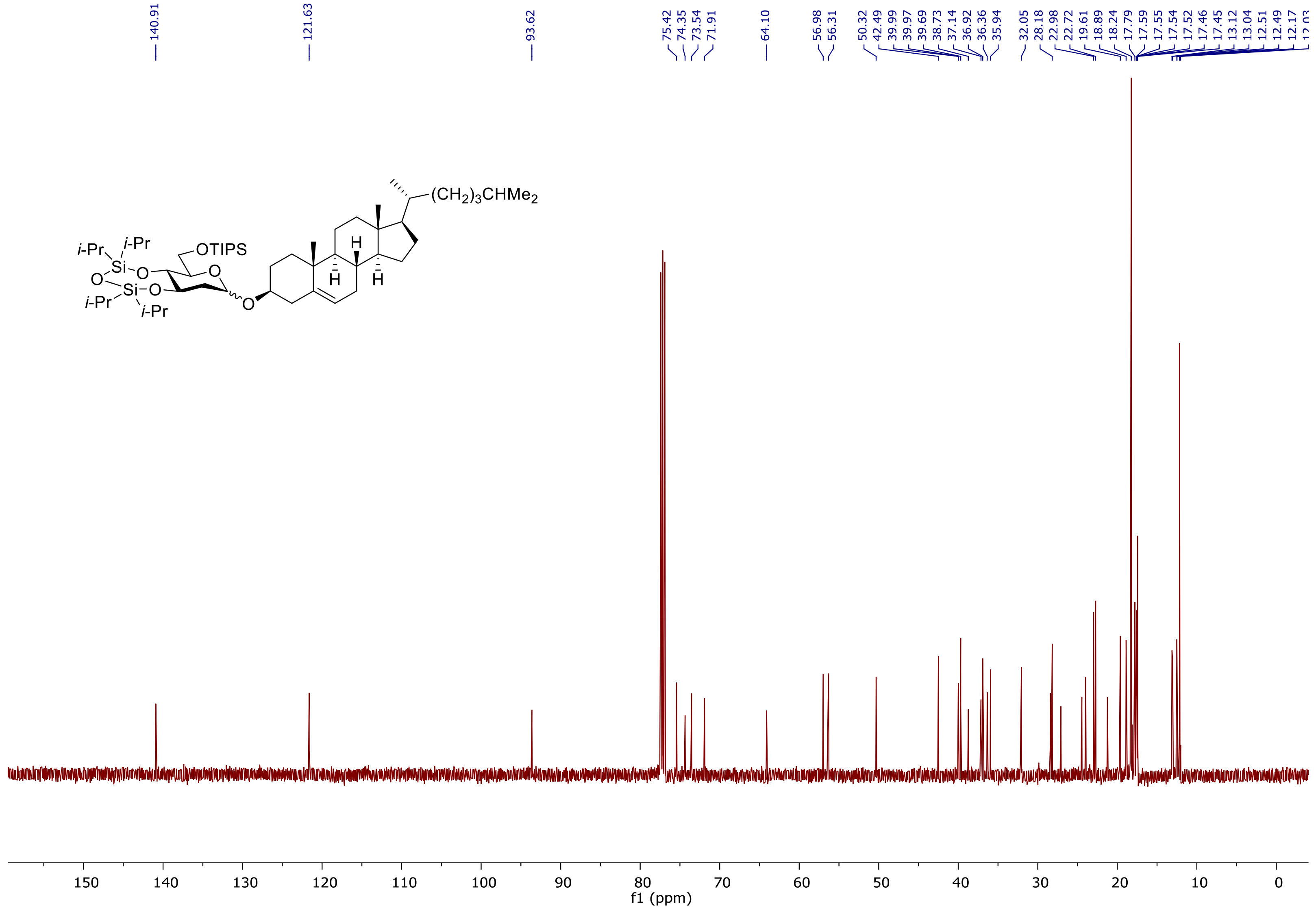

Methyl 2,3,4-tri-*O*-benzyl-6-*O*-(2,6-deoxy-3,4-*O*-(1,1,3,3-tetraisopropyldisiloxane-1,3-diyl)-α/β-L-erythro-hexapyranosyl)-α-D-glucopyranoside (11a) ^{1}H NMR (400 MHz; $CDCl_3$)

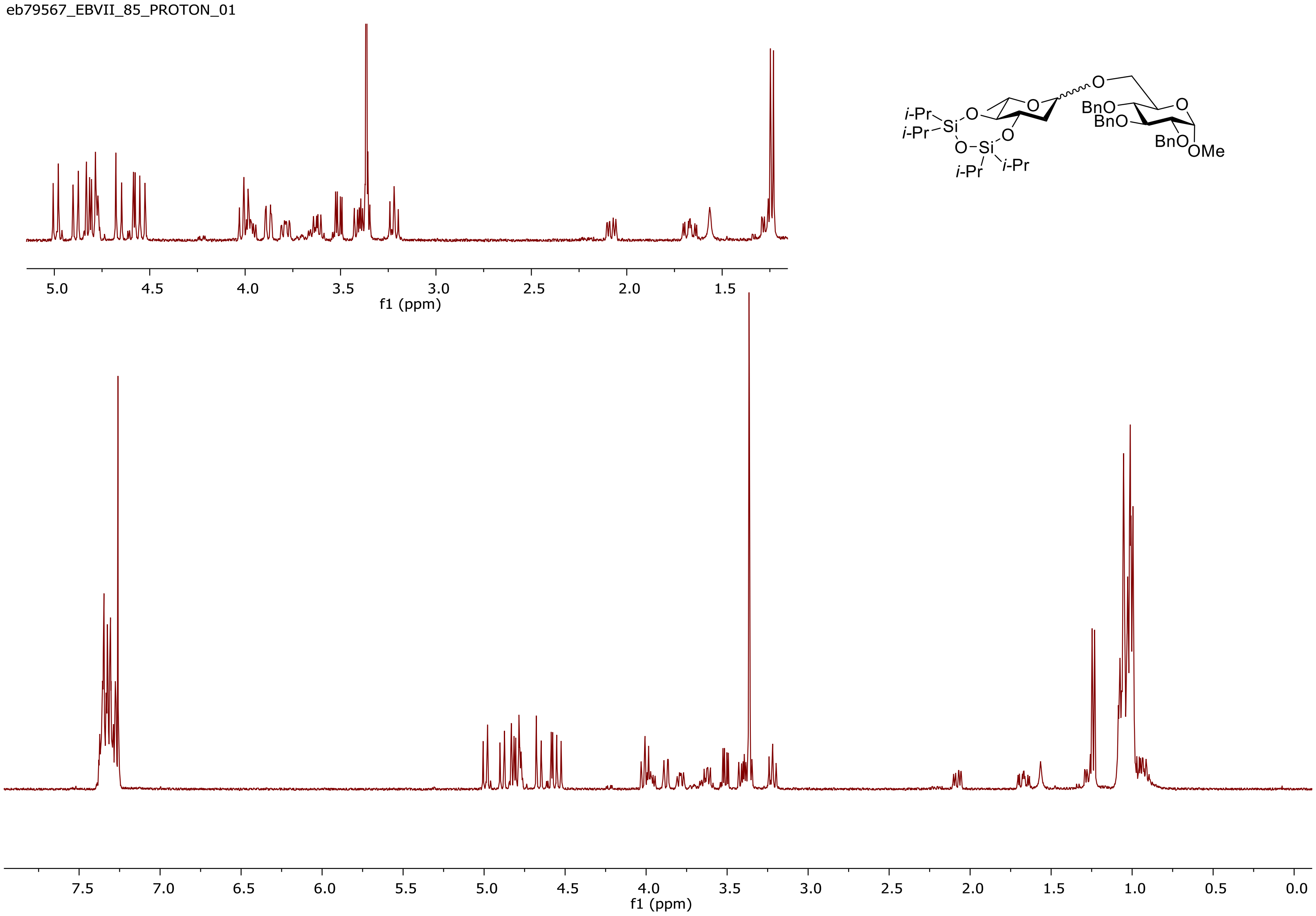

Methyl 2,3,4-tri-*O*-benzyl-6-*O*-(2,6-deoxy-3,4-*O*-(1,1,3,3-tetraisopropyldisiloxane-1,3-diyl)-α/β-L-erythro-hexapyranosyl)-α-D-glucopyranoside (11a) ^{13}C NMR (101 MHz; $CDCl_3$)

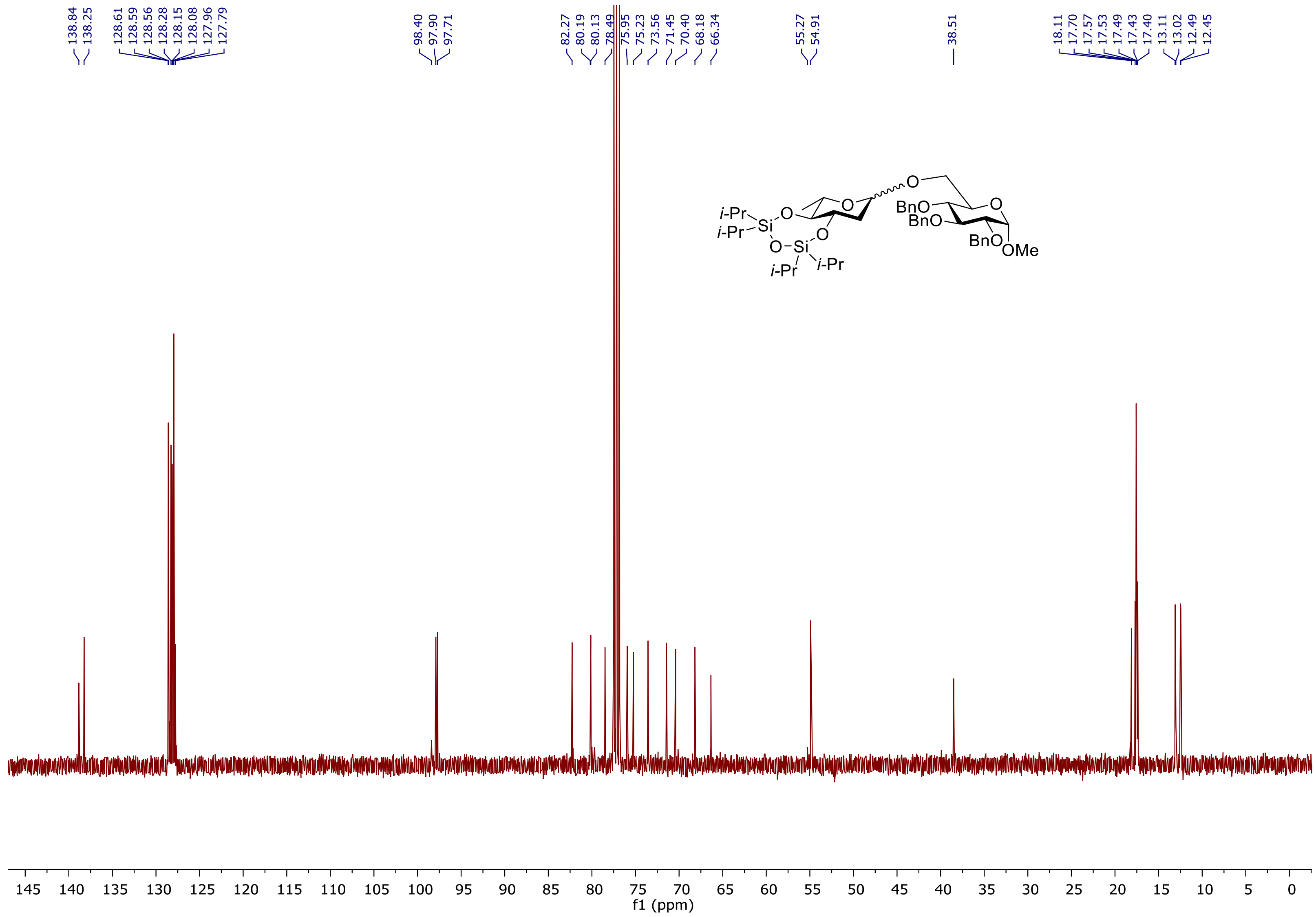

Phenyl 4,6-*O*-benzylidene-3-*O*-(2,6-deoxy-3,4-*O*-(1,1,3,3-tetraisopropyldisiloxane-1,3-diyl)-α-L-erythro-hexapyranosyl)-2-deoxy-1-thio-2-(2,2,2-trichloroethoxycarbonyl-amino)-β-D-glucopyranoside (11d) ^{1}H NMR (500 MHz; CDCl$_3$)

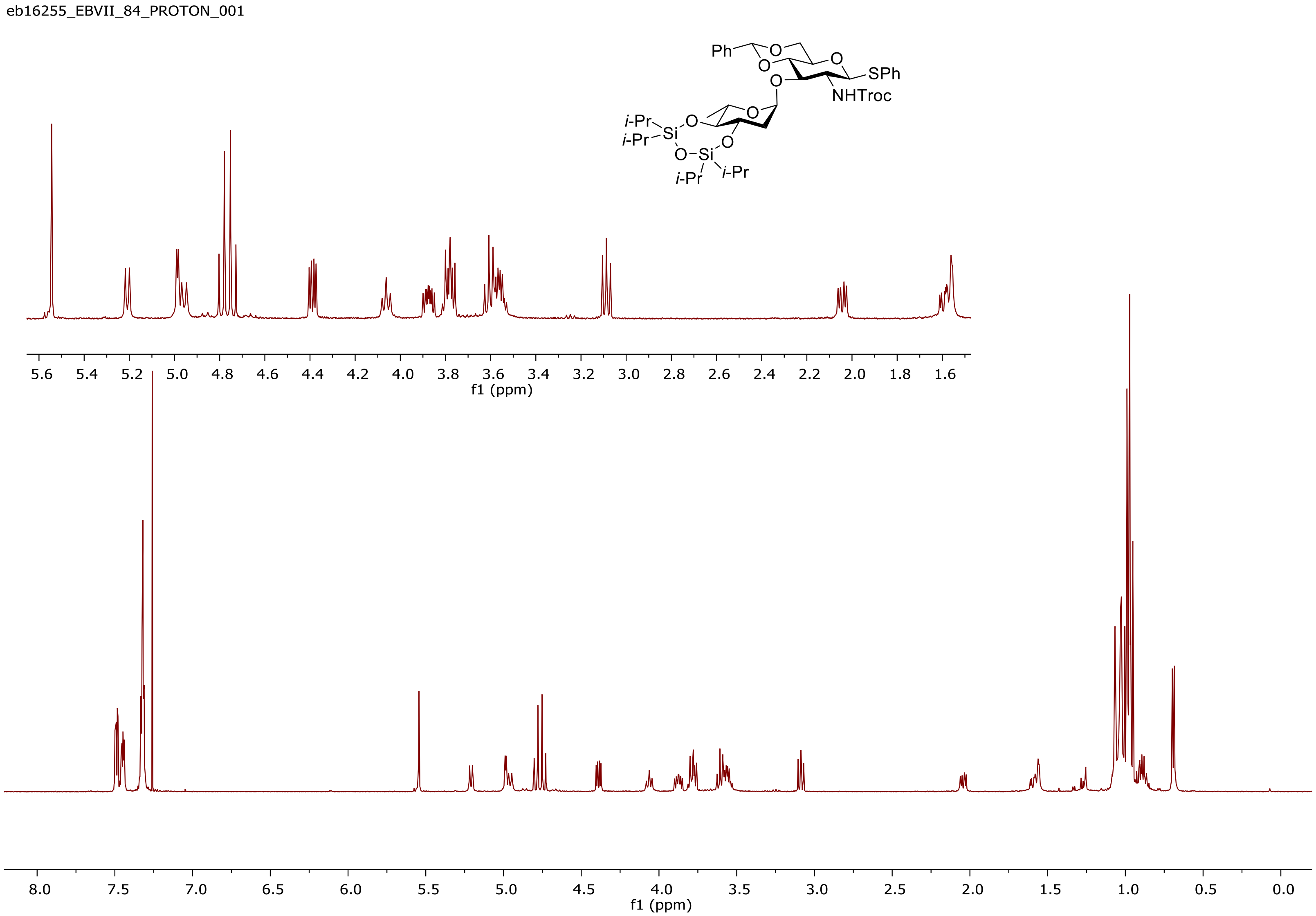

Phenyl 4,6-*O*-benzylidene-3-*O*-(2,6-deoxy-3,4-*O*-(1,1,3,3-tetraisopropyldisiloxane-1,3-diyl)-α-L-erythro-hexapyranosyl)-2-deoxy-1-thio-2-(2,2,2-trichloroethoxycarbonyl-amino)-β-D-glucopyranoside (11d) ^{13}C NMR (126 MHz; $CDCl_3$)

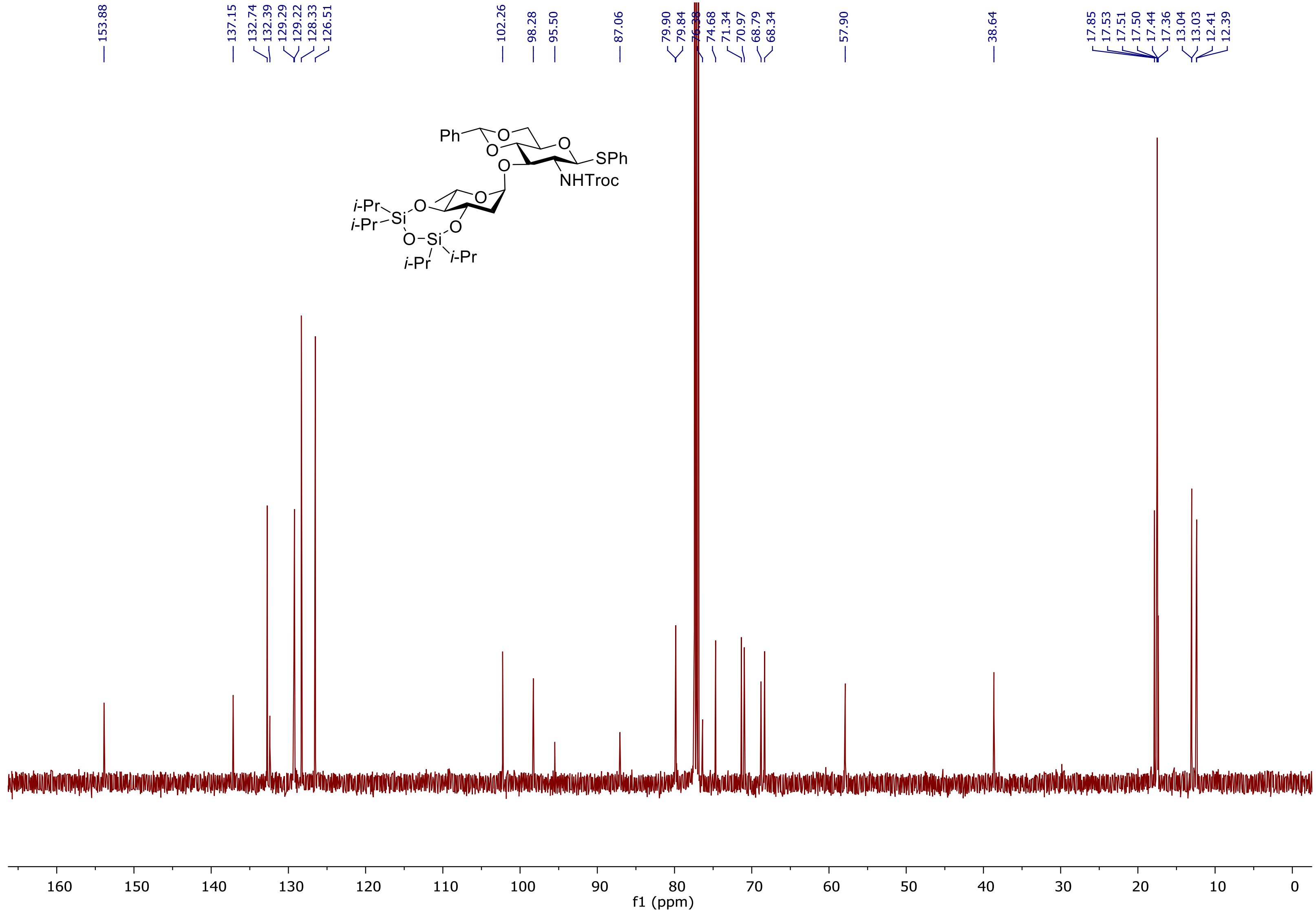

_O_-(2,6-Deoxy-3,4-_O_-(1,1,3,3-tetraisopropyldisiloxane-1,3-diyl)-α/β-L-erythro-hexapyranosyl)-_N_-[(9-fluorenylmethoxy)carbonyl]-L-serine methyl ester (11f) ^{1}H NMR (500 MHz; CDCl$_3$)

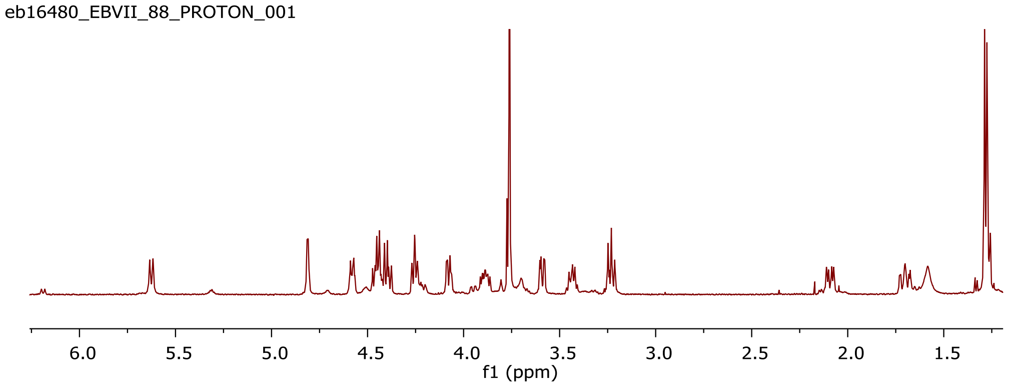

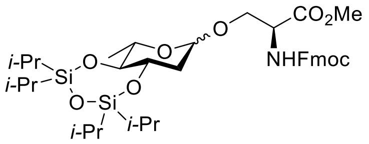

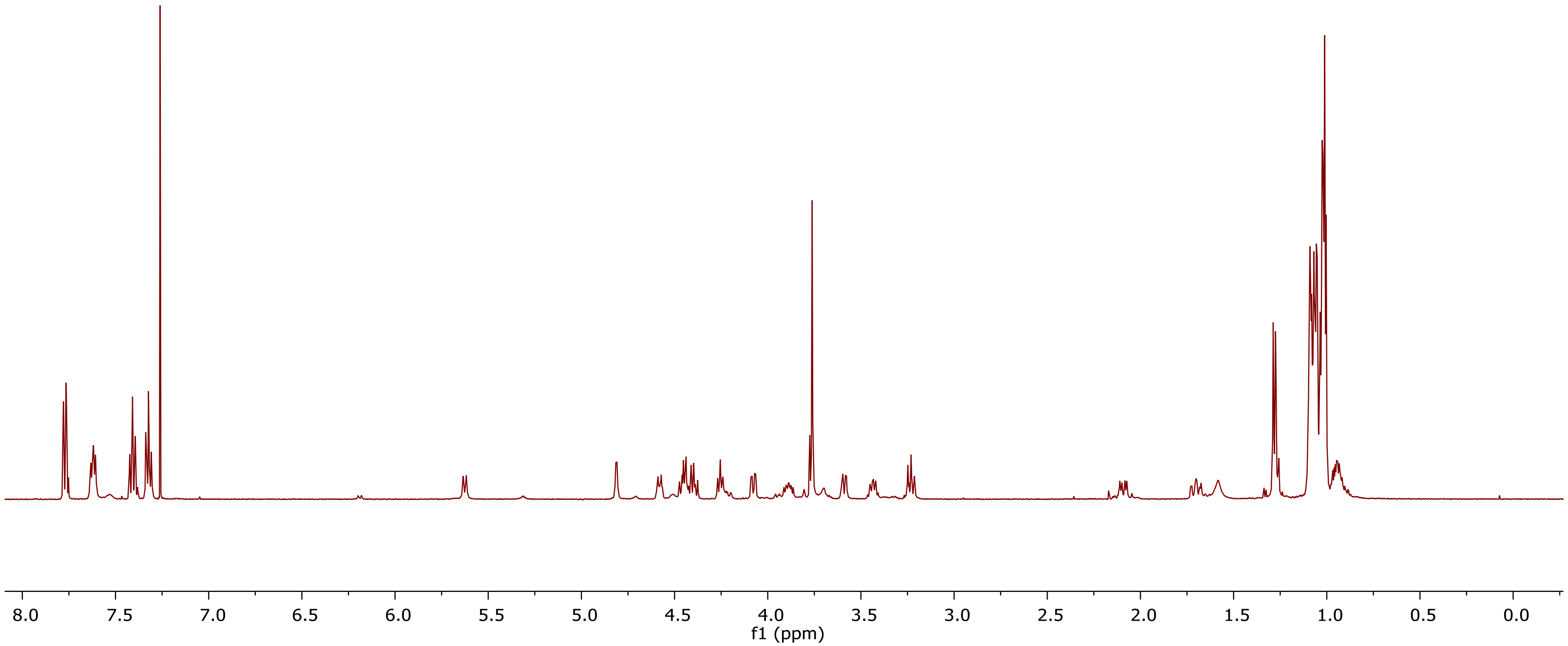

O-(2,6-Deoxy-3,4-O-(1,1,3,3-tetraisopropyldisiloxane-1,3-diyl)-α/β-L-erythro-hexapyranosyl)-N-[(9-fluorenylmethoxy)carbonyl]-L-serine methyl ester (11f) ^{13}C NMR (126 MHz; CDCl$_3$)

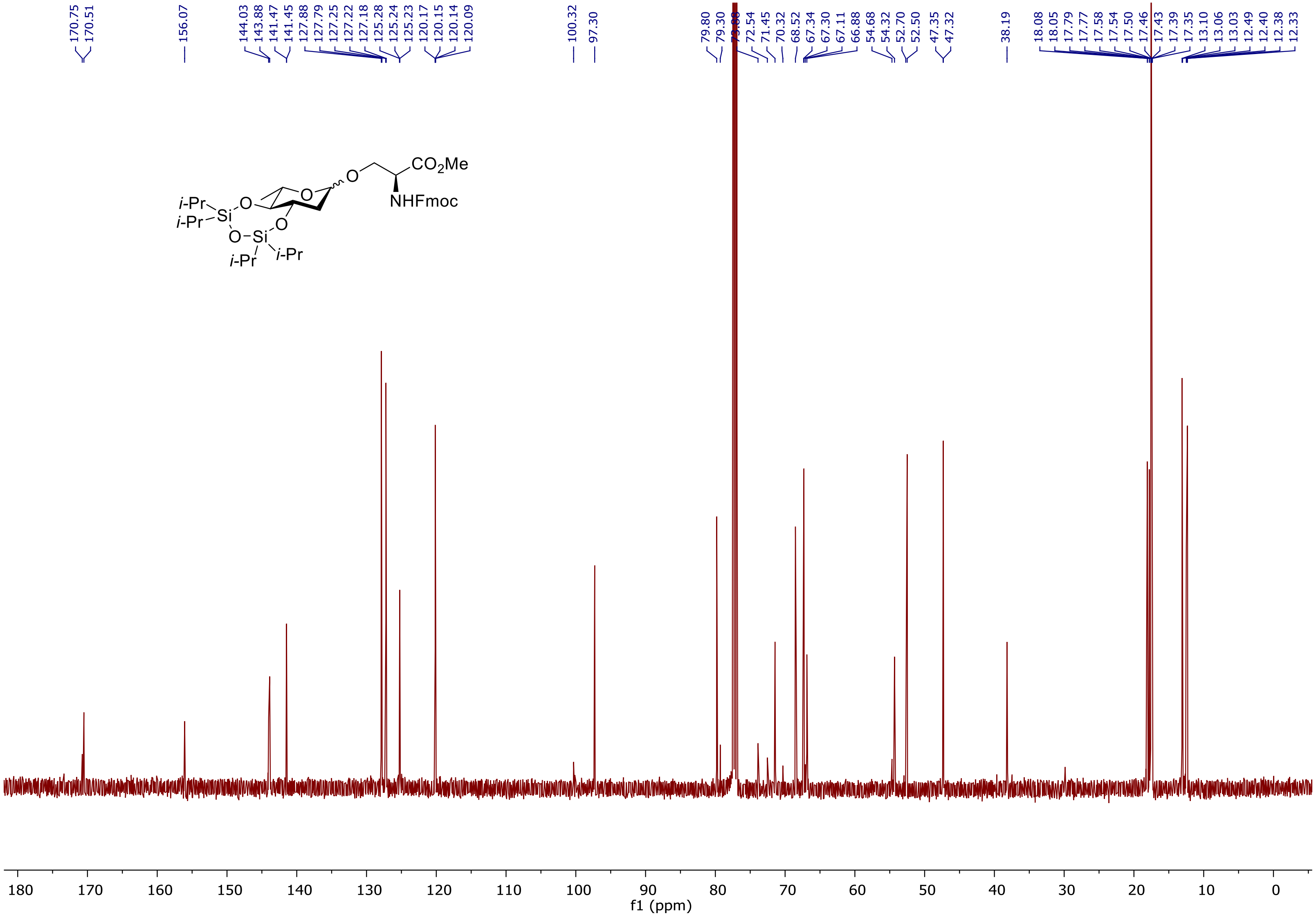

Cholesteryl (2,6-deoxy-3,4-*O*-(1,1,3,3-tetraisopropyldisiloxane-1,3-diyl)-α-L-erythro-hexapyranosyl) (11h-α) ^{1}H NMR (500 MHz; CDCl$_3$)

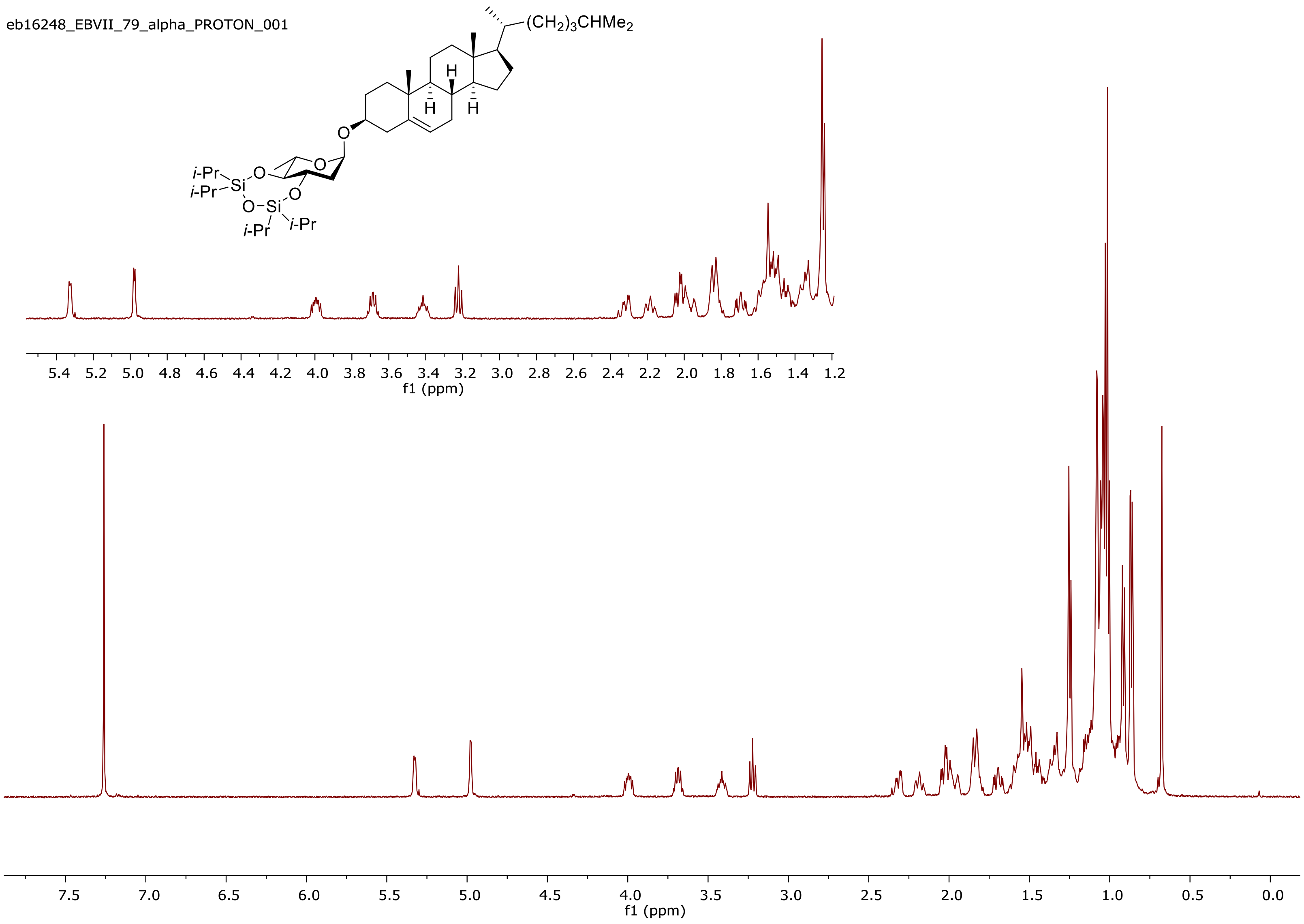

Cholesteryl (2,6-deoxy-3,4-*O*-(1,1,3,3-tetraisopropyldisiloxane-1,3-diyl)-α-L-erythro-hexapyranosyl) (11h-α) ^{13}C NMR (126 MHz; CDCl$_3$). ^{13}C NMR (126 MHz; CDCl$_3$)

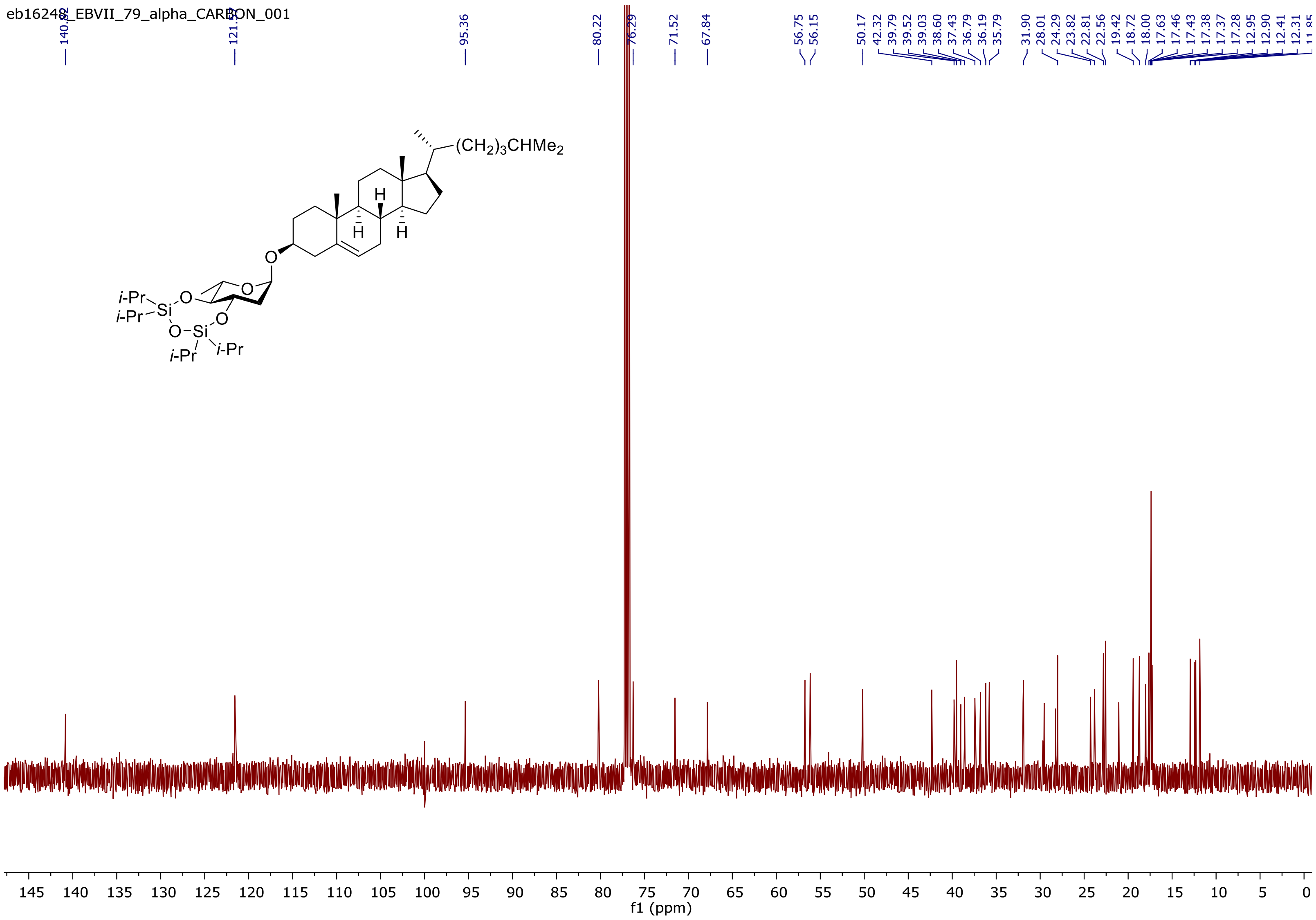

Cholesteryl (2,6-deoxy-3,4-*O*-(1,1,3,3-tetraisopropyldisiloxane-1,3-diyl)-β-L-erythro-hexapyranosyl) (11h-β) ^{1}H NMR (500 MHz; $CDCl_3$)

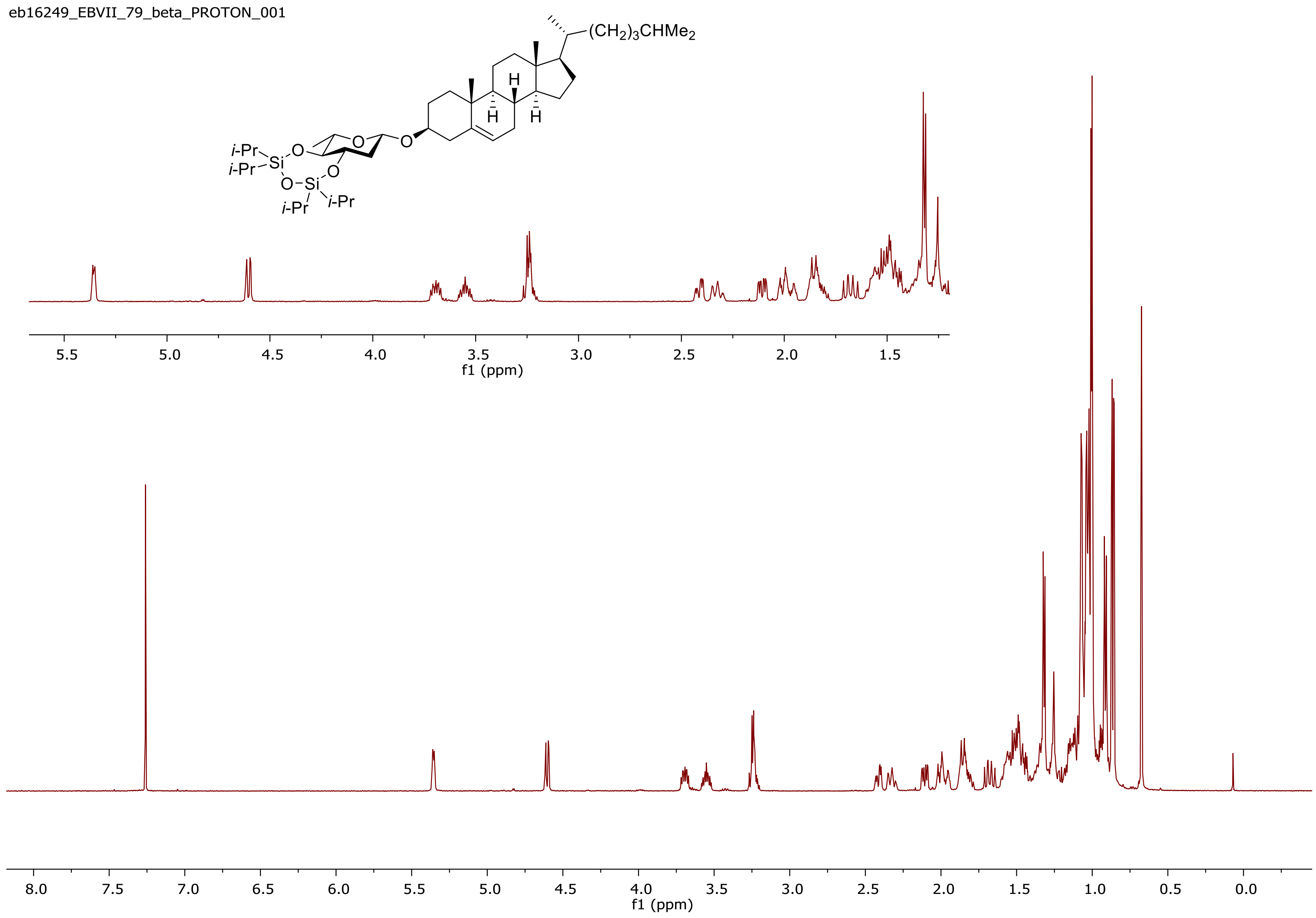

Cholesteryl (2,6-deoxy-3,4-*O*-(1,1,3,3-tetraisopropyldisiloxane-1,3-diyl)-β-L-erythro-hexapyranosyl) (11h-β) ^{13}C NMR (126 MHz; CDCl$_3$)

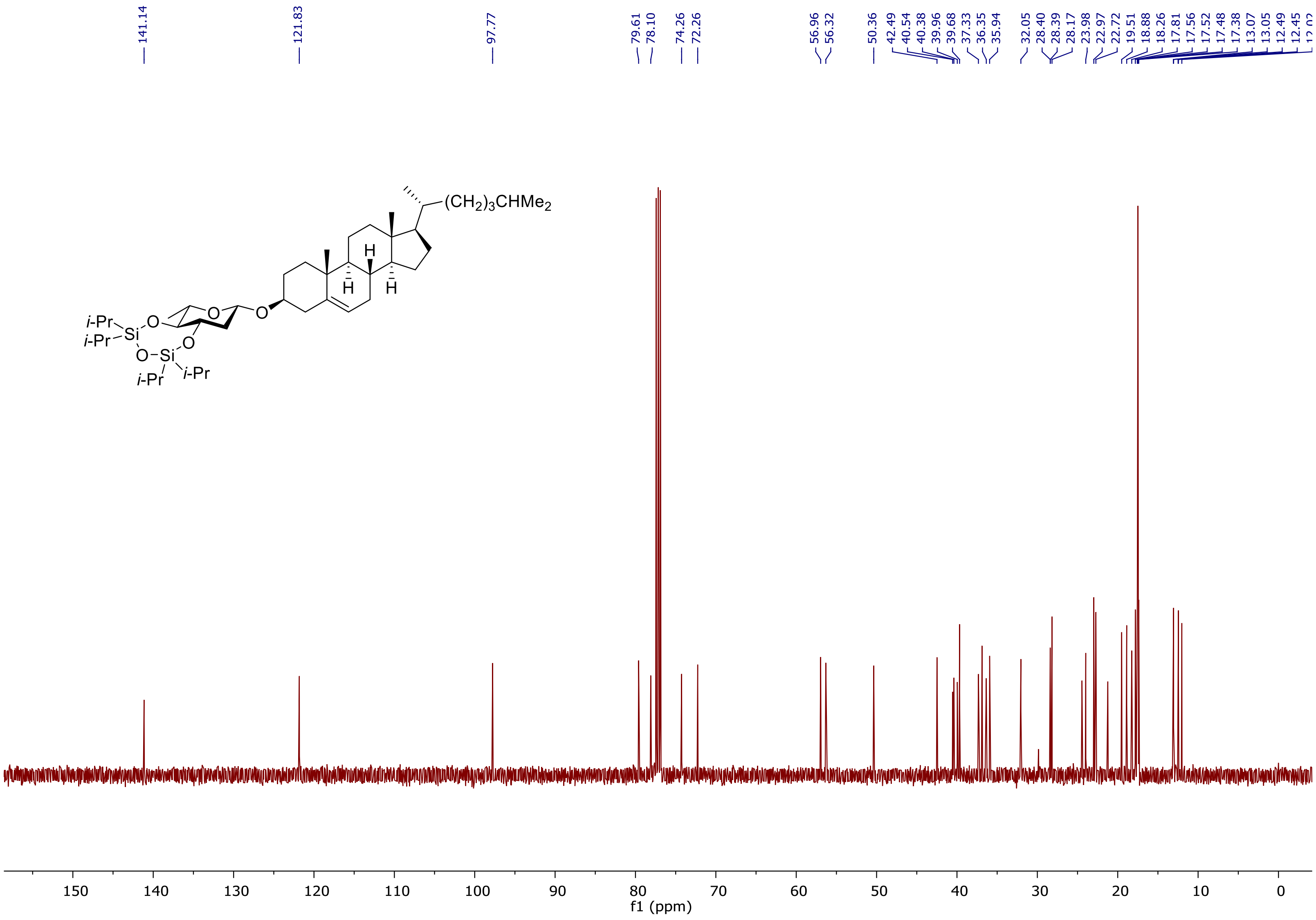

Methyl 2,3,4-tri-*O*-benzyl-6-*O*-(2,6-deoxy-3-*O*-acetyl-4-*O*-(*tert*-butyldimethylsilyl)-α/β-L-*erythro*-hexapyranosyl)-α-D-glucopyranoside (12). ^{1}**H NMR (400 MHz; CDCl$_3$)**

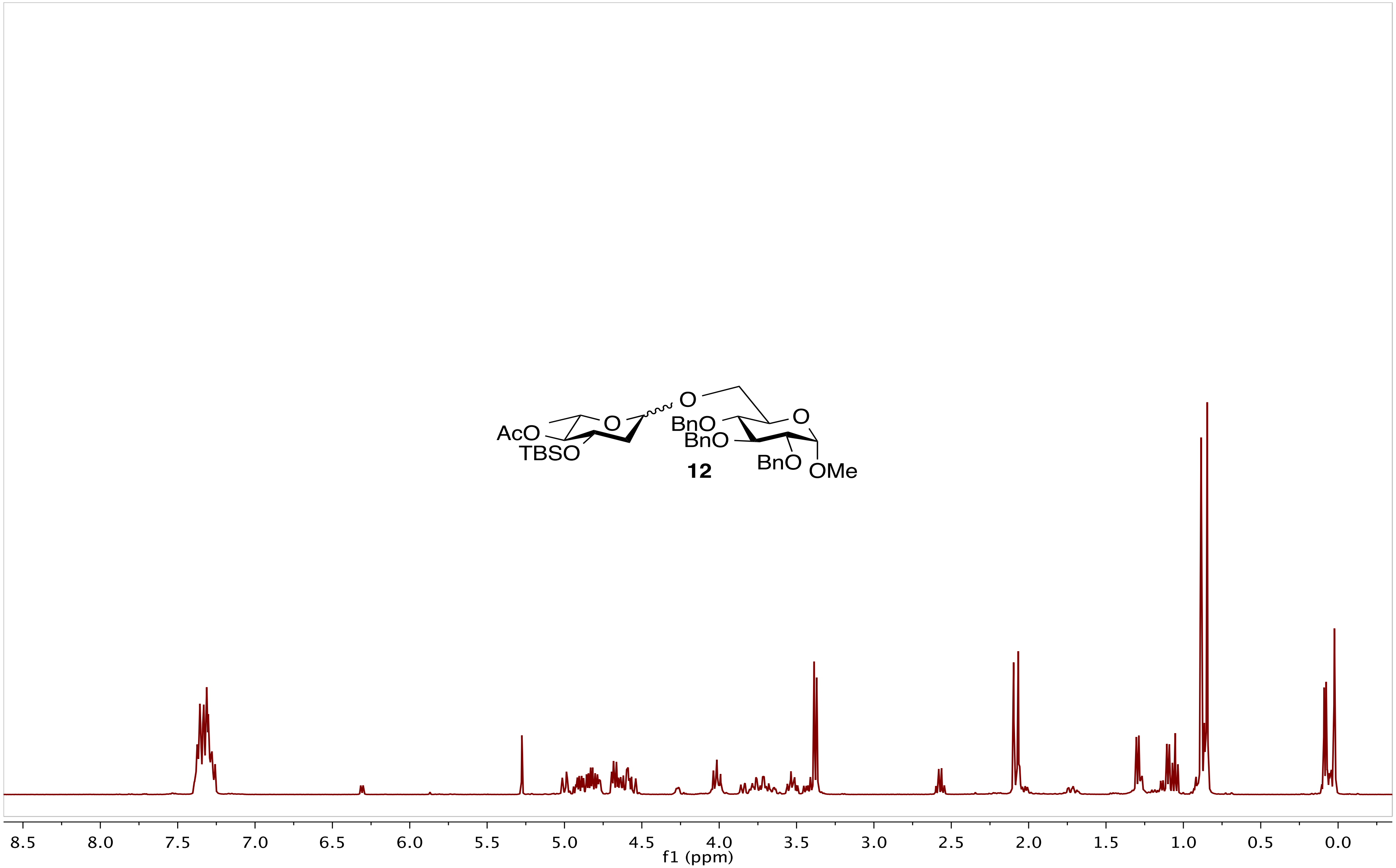

Methyl 2,3,4-tri-*O*-benzyl-6-*O*-(2,6-deoxy-3-*O*-acetyl-4-*O*-(*tert*-butyldimethylsilyl)-α/β-L-*erythro*-hexapyranosyl)-α-D-glucopyranoside (12). ^{13}**C NMR (126 MHz; CDCl$_3$)**

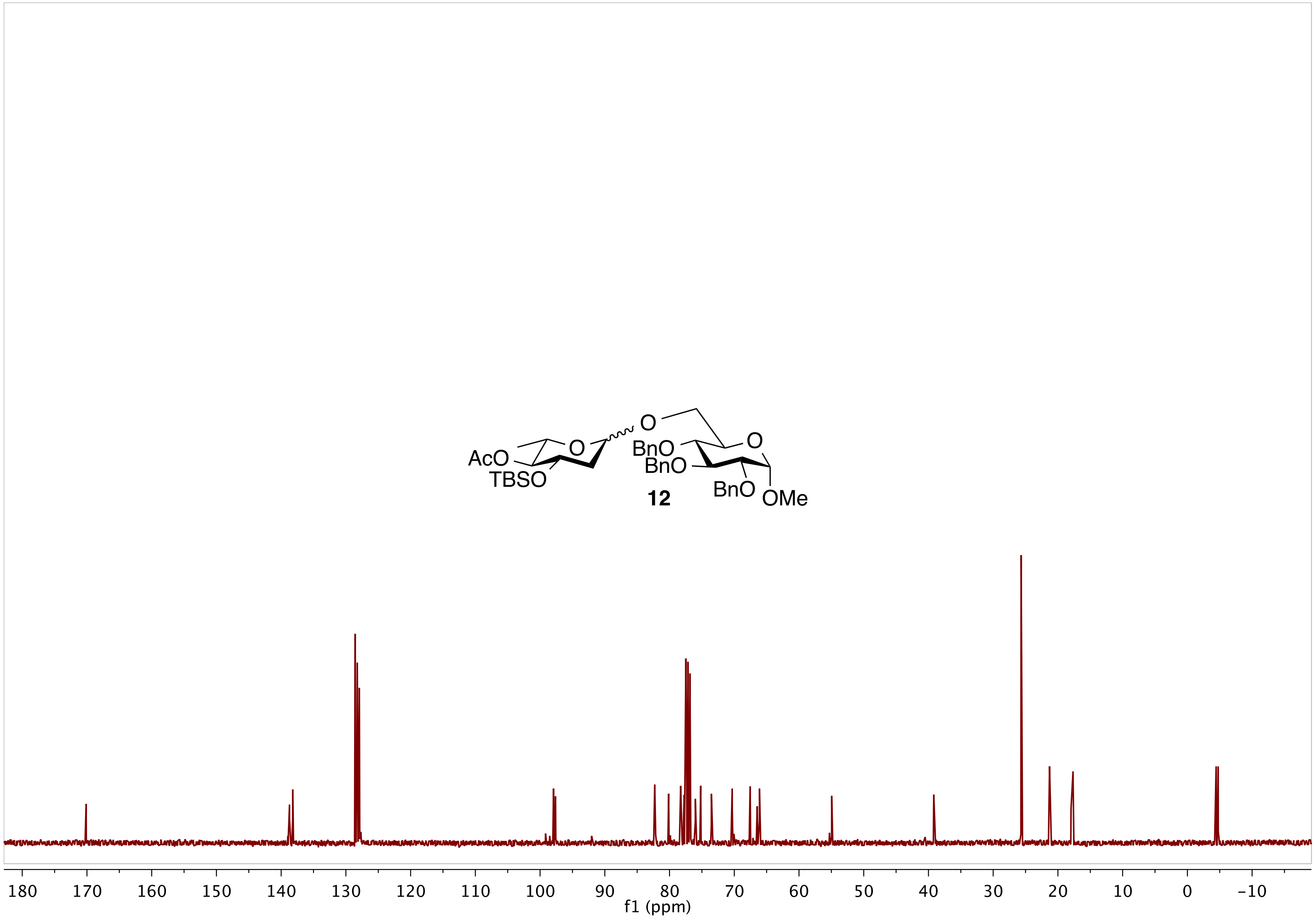

Methyl 2,3,4-tri-*O*-benzyl-6-*O*-((2*S*)-2-deoxy-(2-^{2}H)-3,4-*O*-(1,1,3,3-tetraisopropyldisiloxane-1,3-diyl)-6-*O*-triisopropylsilyl-α-D-*erythro*-hexapyranosyl)-α-D-glucopyranoside (12a) and Methyl 2,3,4-tri-*O*-benzyl-6-*O*-((2*R*)-2-deoxy-(2-^{2}H)-3,4-*O*-(1,1,3,3-tetraisopropyldisiloxane-1,3-diyl)-6-*O*-triisopropylsilyl-α-D-*erythro*-hexapyranosyl)-α-D-glucopyranoside (14b) ^{1}H NMR (400 MHz; $CDCl_3$)

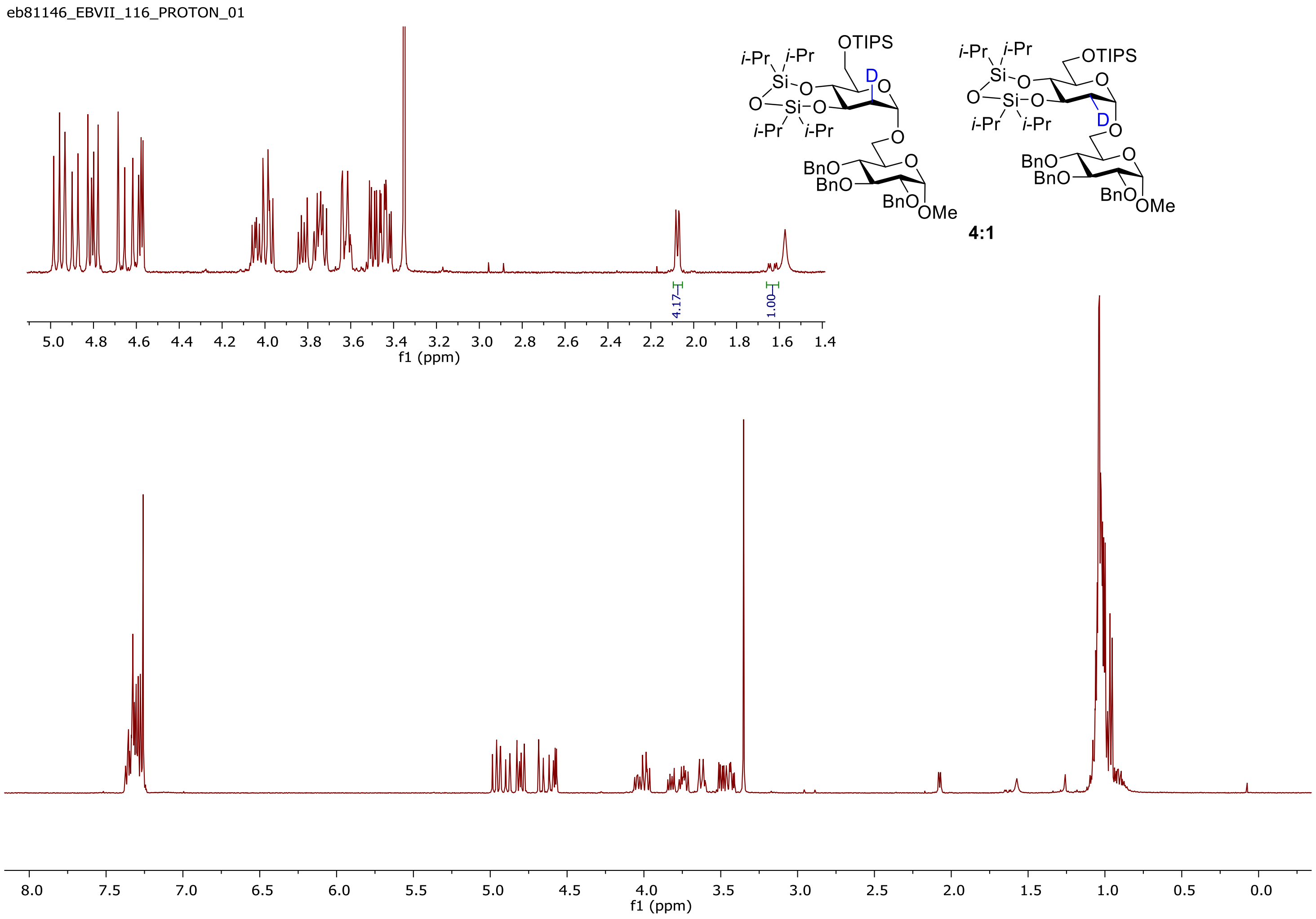

Methyl 2,3,4-tri-*O*-benzyl-6-*O*-((2*S*)-2-deoxy-(2-^{2}H)-3,4-*O*-(1,1,3,3-tetraisopropyldisiloxane-1,3-diyl)-6-*O*-triisopropylsilyl-α-D-*erythro*-hexapyranosyl)-α-D-glucopyranoside (12a) and Methyl 2,3,4-tri-*O*-benzyl-6-*O*-((2*R*)-2-deoxy-(2-^{2}H)-3,4-*O*-(1,1,3,3-tetraisopropyldisiloxane-1,3-diyl)-6-*O*-triisopropylsilyl-α-D-*erythro*-hexapyranosyl)-α-D-glucopyranoside (14b) ^{13}C NMR (101 MHz; $CDCl_3$)

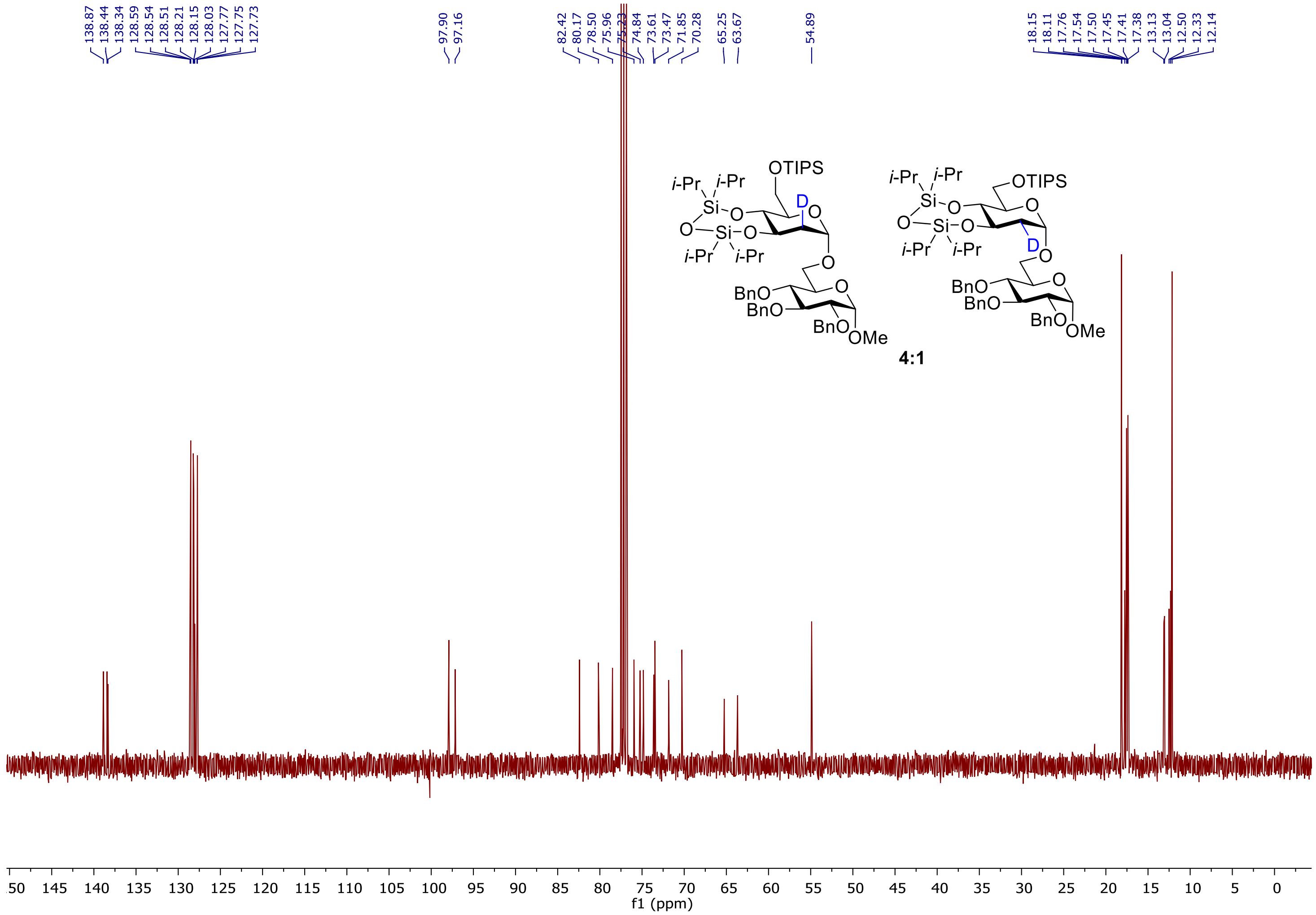

Supplement: Supplementary file 1 [file anie0053-8190-sd1.pdf]
